# Distribution and Evolution of the Lectin Family in Soybean (*Glycine max*)

**DOI:** 10.3390/molecules20022868

**Published:** 2015-02-11

**Authors:** Sofie Van Holle, Els J. M. Van Damme

**Affiliations:** Laboratory of Biochemistry and Glycobiology, Department of Molecular Biotechnology, Ghent University, Coupure links 653, 9000 Ghent, Belgium; E-Mail: Sofie.VanHolle@ugent.be

**Keywords:** lectin, carbohydrate, soybean, evolution, domain architecture

## Abstract

Lectins are a diverse group of proteins that bind specific carbohydrates and are found throughout all kingdoms. In plants, lectins are involved in a range of important processes such as plant defense and stress signaling. Although the genome sequence of *Glycine max* (soybean) has been published, little is known about the abundance and expansion patterns of lectin genes in soybean. Using BLAST and hidden Markov models, a total of 359 putative lectin genes have been identified. Furthermore, these sequences could be classified in nine of the twelve plant lectin families identified today. Analysis of the domain organization demonstrated that most of the identified lectin genes encode chimerolectins, consisting of one or multiple lectin domains combined with other known protein domains. Both tandem and segmental duplication events have contributed to the expansion of the lectin gene family. These data provide a detailed understanding of the domain architecture and molecular evolution of the lectin gene family in soybean.

## 1. Introduction

The legume family is the third largest family within the Angiospermae and represents the second economically most important plant family after the Poaceae. Next to their economic value, grain and forage legumes are also of high nutritional value for human and animal food. Furthermore, most legume species facilitate nitrogen fixation through the formation of a symbiotic relationship with rhizobia resulting in nodule formation on the roots and enabling the plant to survive in soils with poor nitrogen content. Several legumes are considered as high energy crops and are used for biofuel production [1]. These features explain the extensive efforts of many researchers to better understand legume biology and physiology. In the last couple of years, research has mainly concentrated on *Medicago truncatula* (barrel clover), *Lotus japonicus* (Japanese trefoil) and *Glycine max* (soybean) The latter species was the first legume to be sequenced completely at genome level, serving as a reference for other legume species [2].

*Glycine max* is one of the economically most important crops, with a world production of almost 285 million metric ton in 2013 [3]. Soybean seeds are used in particular for food and fodder because of the high protein content. Moreover soybean and peanut together provide more than 35% of the world’s processed vegetable oil [4]. The seed oil content makes soybean an excellent candidate for the production of biofuel. However, if soybean would be grown for energy production, it should be grown on fertile land and would be competing for the land necessary for food production [1].

In 2010 Schmutz and coauthors published the large scale shotgun sequence of *Glycine max* var. Williams 82. Approximately 950 megabases (Mb) are captured in 20 chromosomes, and an additional small amount is present in unmapped scaffolds that mostly consist of repetitive DNA. Similar to all other legume species, the polyploid soybean genome underwent a whole genome duplication (WGD) 59 million years ago (Myr) followed by a specific *Glycine* duplication approximately 13 Myr. These two WGDs were followed by chromosome rearrangements, gene diversification and gene loss [2]. Early 2014, a new assembly (v2.0) of the soybean genome became available from Phytozome (http://phytozome.jgi.doe.gov/). This new release (*Glycine max Wm82.a2.v1*) replaced the first assembly and was constructed using the latest ARACHNE assembler. *Phaseolus* synteny and the available genetic maps for soybean have been used to identify false joins within the previous assembly. The new genome release of *Glycine max* comprises 955 Mb, assembled into 20 chromosomes and 1170 unmapped scaffolds.

Lectins are a diverse group of proteins of non-immune origin found in bacteria, fungi, viruses, plants and animals. They contain at least one non-catalytic domain, which enables them to reversibly bind to specific glycan structures. One class of plant lectins groups all carbohydrate-binding proteins that are constitutively expressed in high amounts, especially in seeds and vegetative storage tissues. Evidence has been presented that these lectins combine a function as storage protein with a role in plant defense against herbivorous insects or animals. Most of these lectins are synthesized with a signal peptide and are directed to the secretory pathway [5,6]. In addition, plants can express specific lectins in response to certain stress conditions such as environmental changes or pathogen attack. In contrast to the abundant lectins, these inducible lectins are expressed in low concentrations, and reside in the nucleus and the cytoplasm of the plant cell. These low abundant lectins most probably interact with glycans inside the plant cell or at the plant cell wall, and as such trigger some signaling pathways in or between plant cells [7,8]. Based on the sequence of their carbohydrate recognition domain (CRD), plant lectins can be classified into twelve families of evolutionary related proteins. These families are in alphabetical order: the *Agaricus bisporus* agglutinin (ABA) family, the amaranthins, the homologs of class V chitinases (CRA), the cyanovirin family, the *Euonymus europaeus* agglutinin family (EUL), the *Galanthus nivalis* agglutinin (GNA) family, the hevein family, the jacalin-related lectins (JRL), the legume lectins, the LysM domain lectin family, the Nictaba-like lectins and the ricin B lectin family [9]. Each CRD is characterized by its amino acid sequence, structure of the binding site and typical folding of the polypeptide. Nevertheless, it has been shown that evolutionary related CRDs of the same family can bind different carbohydrates, which makes it impossible to classify lectins according to their carbohydrate-binding specificity. Moreover, most lectins not only consist of the CRD but also contain one or more unrelated protein domains [10]. This makes other attempts to classify plant lectins based on their carbohydrate-binding specificity [11] or three-dimensional structure [12] less relevant.

The classical soybean agglutinin (SBA) is a tetrameric glycoprotein purified from seeds. It was the first lectin to be cloned in 1983 [13] and is since considered as one of the best characterized plant lectins. SBA is a tetramer consisting of four identical 30 kDa subunits [14,15]. Each subunit carries an N-linked Man_9_(GlcNAc)_2_ chain [16] and possesses one carbohydrate-binding site, specifically recognizing N-acetyl-D-galactosamine (GalNAc) and to a lesser extent D-galactose [17]. The high mannose N-glycan is necessary for the correct folding and assembly of the different polypeptides [18]. The three-dimensional structure of the tetrameric SBA represents a β-sandwich consisting of two curved 12 stranded β-sheets that face each other, creating a large channel in the middle of the tetramer [19]. Next to SBA, three additional highly related isolectins with similar properties have been reported in soybean seeds [20,21]. Furthermore, a vegetative soybean lectin has been described and characterized in detail. Similar to SBA, the soybean vegetative lectin (SVL) is a 119 kDa glycoprotein consisting of four subunits that specifically interact with antibodies raised against SBA. The N-terminal part of the amino acid sequences encoding SBA and SVL shares 63% identity [22] which suggests that these proteins are evolutionary related.

Most plant lectin research focuses on the characterization of one particular lectin family, its distribution and biological properties. For example, studies investigated the omnipresence of EUL, amaranthin and Nictaba-like lectins in different plant species [23,24,25]. To date, little is known about the occurrence of different lectin CRDs within one plant species. Even though the genome was sequenced a couple years ago, few lectins and lectin sequences have been reported in soybean. The new assembly of the soybean genome provides the opportunity to improve the knowledge about the abundance, distribution and expansion of soybean lectins. In this study, we identified proteins belonging to nine different plant lectin families and examined the domain organization, expansion patterns and evolutionary relationship for these lectin genes in soybean.

## 2. Results

### 2.1. Genome-Wide Identification and Distribution of Lectin Genes in Soybean

The abundance and distribution of plant lectin genes within the soybean genome was analyzed using BLAST searches. Hidden Markov models and Clustal Omega alignments confirmed the presence of lectin domains belonging to one of the twelve described plant lectin families. A total of 359 putative lectin genes have been identified in the soybean genome, representing homologs for nine out of twelve lectin families. No homologs for the *Agaricus bisporus* agglutinin, the amaranthins or the cyanovirin family were detected. The abundance of the lectin genes within each family varied greatly. The GNA family is by far the largest lectin family with 166 identified genes, representing approximately 46% of all lectin genes. Not surprisingly, the legume family comes second with 94 genes (26%) and the lectins containing a LysM domain represent the third largest family (13%). All the other lectin families comprise significantly less lectin genes (Table 1). The gene names, chromosome positions, the corresponding amino acid sequences and protein domains for all lectin family members, downloaded through the BioMart tool at Phytozome v10, are shown in Table S1.

**Table 1:** Predicted lectin genes and chromosome localization in soybean.

Lectin Domain	Predicted Genes	Percentage	Chromosome Localization
ABA domain	0	0.0	/
Amaranthin domain	0	0.0	/
CRA domain	6	1.7	13, 15, 17
Cyanovirin domain	0	0.0	/
EUL domain	3	0.8	16, 19
GNA domain	166	46.2	all except chr 5
Hevein domain	6	1.7	2, 12, 13, 16, 19
Jacalin domain	5	1.4	2, 11, 13, 15, 18
Legume domain	94	26.2	all except chr 4 and chr 19
LysM domain	47	13.1	all except chr 12
Nictaba domain	22	6.1	3, 5, 6, 7, 10, 14, 17, 19, 20
Ricin B domain	10	2.8	5, 8, 11, 18

**Figure 1:**
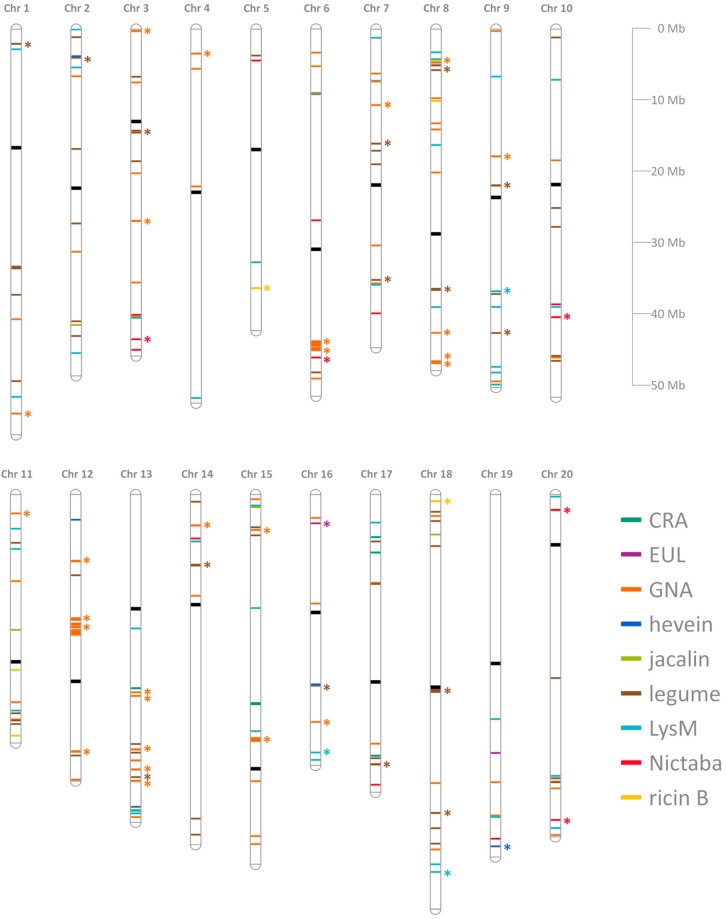
Chromosomal distribution of soybean lectin genes. All the genes for the different lectin families are shown in distinct colours and the centromere positions are indicated in black. Tandem duplicated genes are indicated by an asterisk and segmental duplications are not represented. The chromosome map was generated using the MapChart software and drawn to scale [26].

The transcript start positions were downloaded and used to map all the lectin genes to their corresponding position on the chromosomes (Figure 1). However, two lectin genes could not be mapped to a certain chromosome because they are found in one of the 1170 unmapped scaffolds. It concerns Glyma.U042800 and Glyma.U032400, a GNA and a legume homolog, respectively. Overall, the lectin genes are widely distributed in the genome and are spread over all 20 chromosomes, though the distribution and abundance is not uniform between the different chromosomes or within the same chromosome (Table 1). Chromosomes 6 and 8 carry most lectin genes (10.3 and 10.9%, respectively) while chromosomes 4 and 5 hold the lowest amount of lectin genes (each 1.7%). Genes of the GNA family mainly occur in condensed hotspots, e.g., on chromosomes 6, 12 and 13, and result from numerous tandem duplications.

### 2.2. Domain Organization/Architecture of Putative Soybean Lectins

Since the length of most lectin sequences exceeded that of the lectin domain, the amino acid sequences of all predicted lectins were also searched for the presence of other conserved protein domains. Moreover, the SignalP server provided information about the presence of a signal peptide, necessary to guide proteins to the secretory pathway and the TMHMM Server was used to predict transmembrane domains in the protein sequences (Table 2). The majority of the identified lectin genes encode proteins containing one lectin domain linked to at least one additional protein domain. The different lectins and chimerolectins retrieved from the soybean genome are discussed below. A schematic overview of the lectin domain architecture for the lectin genes within each family is represented and all figures are drawn to scale.

#### 2.2.1. Homologs of Class V Chitinases (CRA)

The *Robinia pseudoacacia* chitinase-related agglutinin specifically recognizes high mannose N-glycans and represents a lectin family with high sequence identity to class V chitinases. However these proteins are essentially devoid of chitinase activity [27]. Plant chitinases can be divided in five classes based on their sequence similarity [28,29].

**Table 2:** Overview of the different lectin families in soybean and their predicted localization in the plant cell.

Lectin Family	Predicted Localization
CRA family	vacuole, membrane bound
*Euonymus europaeus* lectin family	nucleus, cytoplasm
*Galanthus nivalis* lectin family	vacuole, nucleus, cytoplasm or membrane bound
Hevein family	vacuole
Jacalin family	nucleus, cytoplasm
Legume family	vacuole, nucleus, cytoplasm or membrane bound
LysM family	vacuole, nucleus, cytoplasm or membrane bound
*Nicotiana tabacum* lectin family	nucleus, cytoplasm
Ricin B family	vacuole, nucleus, cytoplasm

**Figure 2:**
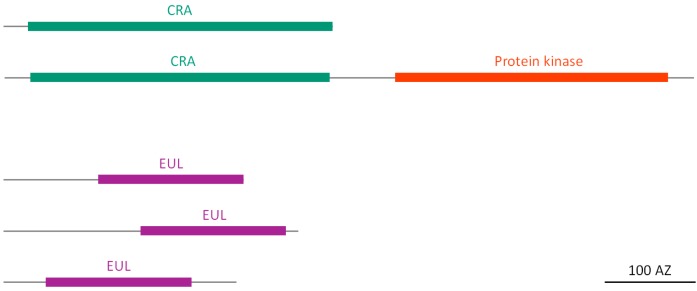
Domain architecture of soybean CRA and EUL homologs.

According to the CAZy database (www.cazy.org), classes I, II and IV belong to the glycosyl hydrolase (GH) family 19, whereas classes III and V are classified in the GH family 18 [30]. In the soybean genome, a total of 45 genes encode for GH 18 proteins, and six of them have been identified as possible chitinase-related agglutinins (CRA). It needs to be investigated whether the six identified CRA homologs from soybean are true lectins and if they have retained their chitinase activity. According to the transcriptome data, each of the six soybean CRAs is expressed. All sequences consist of a signal peptide and one chitinase-related domain of 330–342 amino acids. One of the CRA homologs also contains an additional transmembrane domain and a C-terminal protein kinase domain (Figure 2), thus it can be considered as lectin receptor-like kinase (LecRLK).

#### 2.2.2. EUL Homologs

The EUL family groups all proteins containing at least one domain homologous to the *Euonymus europaeus* lectin (EUL), and was shown to be ubiquitous in land plants [23]. The soybean genome comprises three orthologs of the EUL family and EST data confirm that these genes are expressed. All sequences encode EUL proteins with one EUL domain and variable N- and C-terminal regions (Figure 2). According to the classification system elaborated by Fouquaert *et al.*, the EUL sequences from soybean belong to different groups, one of them being the EULS3 group, a type of proteins that is found in most dicot plant genomes for which sequence information is available [23]. None of the identified EUL homologs contains a signal peptide, suggesting a nucleocytoplasmic localization for these proteins.

#### 2.2.3. GNA Homologs

**Figure 3:**
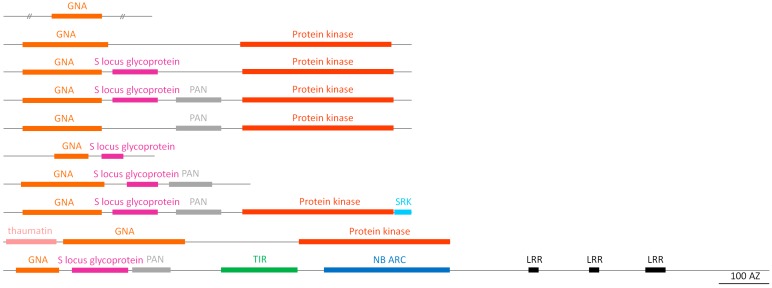
Domain architecture of the GNA family.

GNA homologs are named after a mannose-binding lectin that was first isolated from snowdrop (*Galanthus nivalis*) bulbs [31]. GNA-related lectin sequences have been reported in plants, bacteria, fungi and animals [9]. Within the soybean genome, the GNA-like lectins represent the most abundant lectin family with 166 predicted lectin genes (Table 1). This lectin family also shows most variation with regard to domain architecture. In total, ten different domain combinations of the GNA domain with other protein domains are found in soybean (Figure 3). The largest group comprises all proteins consisting of the GNA domain in combination with/without an S-locus glycoprotein domain and/or a PAN domain and/or a transmembrane domain and/or a protein kinase domain. A small group of chimeric proteins contained all of the above protein domains and an additional S-locus receptor kinase (SRK). One sequence of the GNA homologs is unique and consists of a signal peptide, a GNA domain, an S-locus glycoprotein, a PAN domain, a TIR domain, an NB-ARC domain and three C-terminal LRR domains. Another chimeric protein consists of an N-terminal signal peptide, a thaumatin domain, a GNA domain, a transmembrane domain and a C-terminal protein kinase domain. Next to the chimeric proteins, a few proteins were predicted to have a truncated (64–111 amino acids) GNA domain and variable unrelated N- and C-terminal amino acid sequences. Most of GNA-related sequences from soybean contain a signal peptide and for the majority of these sequences, EST data are available.

#### 2.2.4. Hevein Homologs

**Figure 4:**
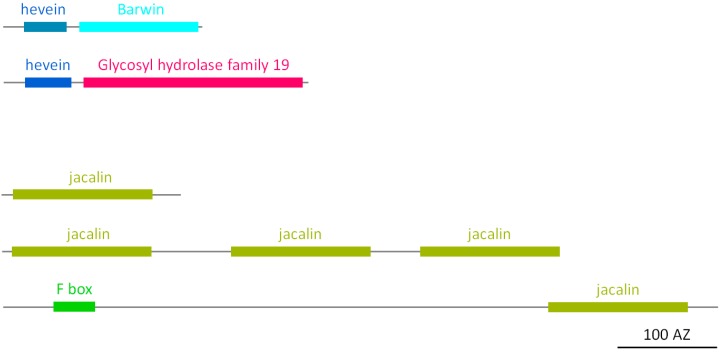
Domain architecture of hevein and jacalin homologs.

Proteins with hevein domains are ubiquitous in plants [6]. Hevein is a small chitin-binding protein with antifungal properties, first identified in the latex of the rubber tree (*Hevea brasiliensis*) [32]. The hevein domain refers to a structural unit of about 40 amino acid residues with sequence similarity to hevein and typically contains eight cysteine residues that are all involved in interchain disulfide bridges that determine the tertiary fold of the lectin domain [6]. The soybean genome comprises two types of hevein orthologs (Figure 4). The first type is a class I pathogenesis-related 4 (PR) protein [33], comprising an N-terminal signal peptide and a hevein domain linked to a C-terminal Barwin domain. The second type is a protein with an N-terminal hevein domain, preceded by a signal peptide and linked to a long C-terminal chitinase domain (GH 19 family). In 2001, the first class I chitinase from soybean was described in the seed coat. The 32 kDa protein contained an N-terminal signal peptide, a hevein domain, a proline rich hinge domain and the catalytic chitinase domain. Aside from its high expression in the seed coat, this gene was also expressed in late developmental stages of pods, leaves and embryos, and transcript levels were increased in response to pathogen (*Phytophthora sojae*) infection [34]. The amino acid sequence of this protein corresponds to one of four genes containing a hevein domain identified in our study.

#### 2.2.5. Jacalin Homologs

BLAST searches revealed that the soybean genome encodes five proteins, containing at least one protein domain with homology to the T-antigen specific lectin (jacalin) that was originally isolated from the seeds from jack fruit [35,36]. Jacalin-related sequences are not only ubiquitous in plants, but are also present in fungi, bacteria, vertebrates and invertebrates [9,37]. One of the soybean homologs comprises the jacalin domain alone, and another one contains an additional F-box domain at its N-terminus (Figure 4). The three other putative jacalin-related lectin genes encode proteins containing three tandem arrayed jacalin units. ESTs confirm the expression of all jacalin-related sequences from soybean. In contrast to jacalin, none of these sequences contains a signal peptide, suggesting the proteins will reside in the nucleocytoplasmic compartment.

#### 2.2.6. Legume Homologs

**Figure 5:**
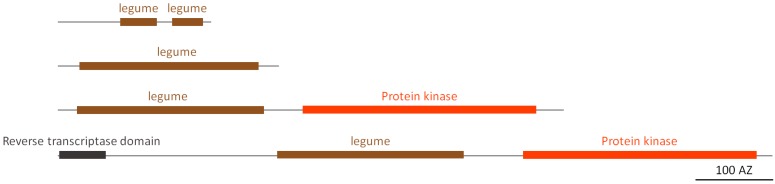
Domain architecture of legume lectin homologs.

Legume lectins are a large family of homologous proteins originally found in the seeds of most legume species. However, this type of lectins has also been identified in a few other plant families, and some evidence for sequences related to the legume lectin domain has also been reported in bacteria and animals [9]. The soybean agglutinin, the prototype of soybean legume lectins, comprises a single legume domain and represents the second largest type of legume homologs in soybean (Figure 5). The largest group of sequences encode L-type (legume) LecRLKs, proteins containing an N-terminal legume domain and a C-terminal protein kinase domain. In most protein sequences of this type, a transmembrane domain was identified that assigns an extracellular localization of the lectin domain. Next to these two groups, there are two predicted proteins with different domain architectures: one protein consists of two short in tandem arrayed legume domains, and another protein sequence is similar to that of the L-type LecRLKs but has an additional N-terminal reverse transcriptase domain. Though most of these sequences contain a signal peptide, there are also sequences which lack a signal peptide (e.g., the legume homolog containing the reverse transcriptase domain) suggesting that these legume lectin homologs will be distributed among different plant compartments.

#### 2.2.7. LysM Homologs

**Figure 6:**
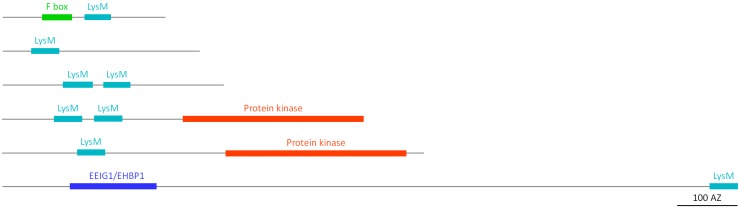
Domain architecture of LysM homologs.

The LysM motif was originally identified in enzymes involved in bacterial cell wall degradation [38]. However, LysM domains are widespread in nature [9] and have been identified first in plants as part of the legume Nod factor receptor-like kinases (RLKs) [39,40]. Of all lectin families in soybean, the LysM domain containing lectins represent the third largest group. In total six different domain architectures containing at least one LysM domain are present in soybean (Figure 6). The two largest groups are the proteins consisting of one LysM domain, and the LysM LecRLK proteins. In addition some LysM sequences encode proteins with two tandem arrayed LysM domains. For all LysM LecRLK sequences, a transmembrane domain was detected between the LysM domain and the protein kinase domain. Two small groups of sequences encode LysM lectins with an N-terminal F-box domain or the LysM domain linked to the EEIG1/EHBP1 domain at its N-terminus. The majority of the identified LysM-related genes in soybean is fully covered by assembled EST contigs and most of the translated amino acid sequences possess a signal peptide. Recently, the LysM domain containing orthologs from *Lotus japonicus* and *Medicago truncatula* Nod factor receptor kinases have been reported (GmNFR1α-GmNFR5α/β) in soybean [41]. These proteins belong to the LysM LecRLK group and overexpression of GmNFR1α resulted in increased nodulation, unlike overexpression of GmNFR5. However, GmNFR5 is generally more transcribed than GmNFR1 [41,42]. GmNFR5α and GmNFR1α form functional complexes that efficiently recognize Nod factors. Mutation of GmNFR1β, another homolog does not affect nodulation, probably due to the formation of dysfunctional receptor complexes with GmNFR1α [41].

#### 2.2.8. Nictaba Homologs

**Figure 7:**
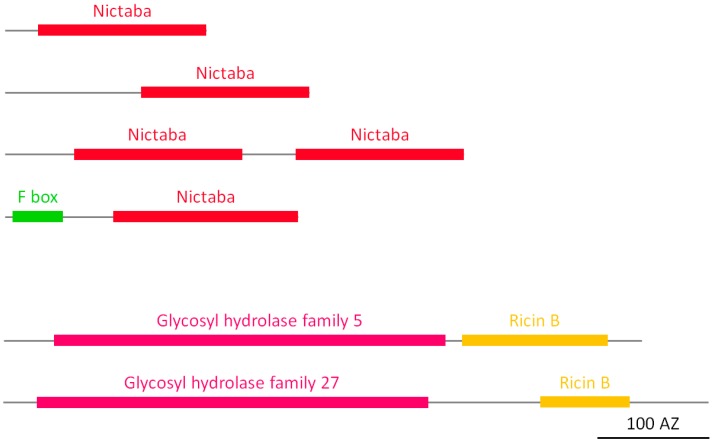
Domain architecture of Nictaba homologs and ricin B lectins.

The nucleocytoplasmic protein Nictaba is a jasmonate inducible lectin that was first identified in the leaves of tobacco plants [43]. Extensive searches revealed that Nictaba-like sequences are widespread in the plant kingdom [25]. Analysis of the soybean genome resulted in the identification of four different types of Nictaba-like lectins. All sequences lack a signal peptide. Hence these proteins are synthesized on the free ribosomes and most probably reside in the cytosol or the nucleus, similar to Nictaba. An important group of Nictaba-related sequences encode so-called F-box Nictaba proteins, chimeric proteins in which an F-box domain is C-terminally linked to a lectin domain homologous to Nictaba. Other Nictaba-like lectins contain one or two Nictaba domains, preceded by variable unrelated N-terminal sequences (Figure 7). EST data confirmed the expression of most of these Nictaba-related genes.

#### 2.2.9. Ricin B Homologs

Ricin B homologs are named after the lectin domain of a toxic protein (called ricin) characterized from *Ricinus communis*. Ricin is a ribosome-inactivating protein (RIP), a chimeric lectin composed of an N-terminal A domain with RNA N-glycosidase activity [44] and a C-terminal B domain with carbohydrate-binding activity. The ricin B lectin family is widespread in nature and homologs have been reported in bacteria, fungi, animal and plant species [9]. The soybean genome comprises two types of ricin B homologs and according to EST data, all the genes except for one are expressed. In both types, the ricin B lectin domain is preceded by an enzymatic domain of the glycoside hydrolase family, in particular the glycoside hydrolase family 5 (GH 5) or family 27 (GH 27) (Figure 7). The ricin B domain linked to the GH 27 domain is apparently shorter (about 80 amino acids) than the lectin domain (120 amino acids) in the homolog containing the GH 5 domain. A signal peptide was detected in all but one of the ricin B homologs in soybean, thus suggesting that most of these proteins are synthesized on the ER. Interestingly, RIPs containing a domain with N-glycosidase activity and a lectin domain have not been identified in the soybean genome.

### 2.3. Tandem and Segmental Duplication Largely Contributed to the Expansion of Lectin Genes in Soybean

The observed variation in the number of homologs between the different lectin families and the distinct chromosomal distribution is probably the result of a series of evolutionary processes. The polyploid soybean genome has undergone two polyploidy events that resulted in a genome in which 75% of the genes are present in multiple copies. The fate of duplicated genes in soybean has recently been studied and the majority of the duplicated genes showed a differential expression and had undergone sub-functionalization [45]. Of the different types of gene duplication (whole-genome, tandem, segmental, transposition), tandem and segmental duplications were studied to gain more insight in the differential expansion of the soybean lectin genes. Mapping the lectin genes to their physical positions on the chromosomes (Figure 1) revealed that many lectin genes are clustered together, suggesting that they might be the result of tandem duplication events. Tandem duplicated genes were defined as one or more members of the same family occurring within a certain intergenic region. A total of 53 tandem duplication blocks have been identified involving 188 genes of seven different lectin families (Table 3). The CRA family and the jacalin family are the only lectin families for which no tandem duplications were detected.

**Table 3:** Tandem and segmental duplication in soybean.

Lectin Family (Number of Genes)	Tandem Duplication		Segmental Duplication
	Duplication Clusters	Genes Involved	Genes Involved
CRA (6)	0	0	4
EUL (3)	1	2	0
GNA (166)	26	114	69
Hevein (6)	1	2	3
Jacalin (5)	0	0	5
Legume (94)	16	49	51
LysM (47)	2	4	38
Nictaba (22)	5	13	11
Ricin B (10)	2	4	7

**Figure 8:**
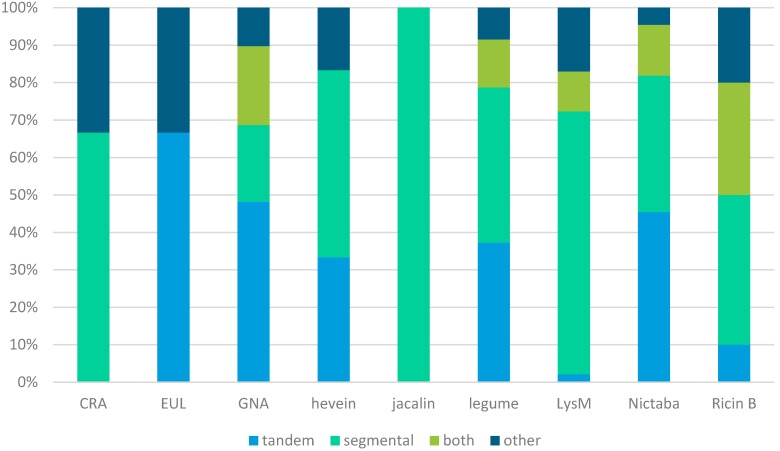
Evolutionary mechanisms responsible for the expansion of the lectin families.

In addition, the contribution of duplications of chromosomal regions (segmental duplications) was investigated. The soybean McScan output data, available from the Plant Genome Duplication Database (PGDD—http://chibba.agtec.uga.edu/duplication/) [46], was searched for collinear blocks containing lectin genes. A total of 166 lectin genes were shown to be involved in segmentally duplicated events belonging to all lectin families, except for the EUL family (Table 3). These 166 genes were found in 121 different collinear blocks, containing one or more lectin genes belonging to different families. These data suggest that lectin gene expansion is mainly the result of segmental duplication, especially for the jacalins for which the increase in family size is completely due to segmental gene duplication. Though helpful, these data are not completely representative since genes of some lectin families (GNA, legume, LysM, Nictaba and ricin B) are involved in both segmental and tandem duplications. The evolutionary mechanisms responsible for the expansion of the different lectin families are represented in Figure 8 (Table S2).

In general, tandem and segmental duplication contributed equally (36.5% and 36.2%) to lectin gene expansion in soybean. About 16.2% of the genes was involved in both segmental and tandem duplication and the remaining 11.1% of the expansion is due to other mechanisms such as retrotransposition. However, there are important differences between the different lectin families. For the LysM, CRA and jacalin families, tandem duplication had (almost) no influence on the expansion of these families, while for all the other families, tandem duplication contributed 33%–67% to gene expansion.

## 3. Discussion

Since the discovery of the soybean agglutinin in 1952 [47], our knowledge about lectins and putative lectin genes and their distribution in plants has increased enormously. With the advent of proteomics and genomics, a vast amount of sequences has become available, which allows whole genomes to be screened for the presence of particular protein domains. In our analysis, a total of 359 lectin genes has been identified in the soybean genome, and grouped into nine distinct lectin families. Gene duplications on various levels (tandem, segmental and whole genome duplications) have been recognized as one of the primary forces in the evolution of eukaryotic genomes [48]. Being a paleopolyploid, the soybean genome has undergone two rounds of duplication 59 and 13 Myr [2] which resulted in a genome with nearly 75% of its genes present in multiple copies [45]. The success of ancient duplicated genomes is due to the facilitated plant response under specific conditions, thereby increasing their chances of survival compared to diploids [49]. Our current results reveal that the whole lectin gene family in soybean has expanded through both tandem and segmental duplication (or a combination of both mechanisms). The data also show that the different lectin families evolved and expanded differentially which lead to the great variation in number of genes per lectin family. The fact that a lot of lectin genes have been retained after WGDs, suggests that lectin sequences are associated with biological needs or advantages for the plant to adapt to changing environmental stresses. Duplicate gene preservation by means of subfunctionalization or neofunctionalization illustrates potential biological benefits for retention of these genes [49,50].

Analysis of the domain architecture of the identified soybean lectins revealed that most of them are chimerolectins, consisting of one or more carbohydrate-binding domains tandemly arrayed to other protein domains. The occurrence of an F-box domain in combination with a single lectin domain was found in three different lectin families: the jacalin family, the LysM-related lectins and the Nictaba-like lectins. The F-box domain is part of the SCF complex, involved in ubiquitination of proteins destined for proteasomal degradation [51,52]. It has been suggested that protein-carbohydrate interactions through the C-terminal sugar-binding lectin domain could facilitate degradation of glycoproteins in plants, similar to mammalian F-box proteins [9,53,54,55]. Combinations of an F-box and lectin domain are not unique to soybean and can be found throughout the plant kingdom, suggesting a general role in protein degradation. Domain combinations of F-box Nictaba and F-box LysM are highly conserved in plants, in contrast to the F-box jacalin combination, which has only been reported in *Arabidopsis* [9,25,54,56].

Another striking observation is that different families of glycoside hydrolases are linked to lectin domains. In the ricin B family, the lectin domain is preceded by either a GH 5 or GH 27 domain while for members of the hevein lectin family, the GH 19 domain is C-terminally linked to the lectin domain. Glycoside hydrolases are a diverse group of enzymes. GH 5 represents one of the largest glycoside hydrolase families and is formally known as the “cellulose family A” [57]. The glycoside hydrolases of family 19 are chitinases comprising class I, II and IV chitinases and GH family 27 together with GH family 31 and 36 form the GH-D clan, a superfamily of α-galactosidases, α-N-acetyl-galactosaminidases and isomaltodextranases [30]. Plants do not contain chitin but it has been assumed that plant chitinases play a role in the defense against fungal pathogens as they can hydrolyse chitin from the fungal cell wall [58].

In addition, other plant defense related domains were also identified in combination with lectin domains. The Barwin domain was identified in the soybean genome in combination with an N-terminal hevein domain (class I PR-4), and was first identified in a wound induced barley seed protein. Like hevein, it is cysteine rich and has the ability to bind carbohydrates [59,60]. Class I PR-4 proteins have also been identified in potato (WIN2), Arabidopsis (HEL), tobacco (CBP20) and jelly fig (FaPR-4) [61,62,63,64], and were induced upon wounding or viral/fungal infection. This suggests that the two tandem arrayed soybean class I PR-4 genes also play a role in plant defense and might be upregulated upon pathogen attack or wounding. Another defense-related protein architecture is the thaumatin domain fused to a GNA and protein kinase domain. Thaumatin related proteins are classified as PR-5 proteins and transcript levels for osmotin, a tobacco PR-5 protein are enhanced after pathogen attack and osmotic stress [65,66]. Similar to the thaumatin domain, combinations of the GNA domain with the NB-ARC, TIR and LRR domains are related to plant defense since these domains are known to be involved in disease resistance [67,68].

A more common example of domain architecture are the lectin receptor-like kinases. These are mainly plasma membrane localized proteins and contain an intracellular kinase domain, a transmembrane region and an extracellular lectin domain. Although a lot of LecRLKs have been reported, only a limited number of proteins have been functionally characterized, hence little information is available with respect to the carbohydrate-binding activity of the lectin domain. In soybean, combinations of the protein kinase domain are found with the CRA domain, the GNA domain, the LysM type lectin domain and with legume lectin domains. Some soybean LysM LecRLKs are involved in the symbiotic relationship with rhizobia as they can recognize Nod factors [41]. *Medicago* legume LecRLKs were also shown to be involved in symbiosis [69] while Arabidopsis L-type LecRLKs are induced upon treatment with elicitors and pathogen attack [70]. GNA (G-) type LecRLKs represent the largest group of lectin receptor like kinases in soybean. In most proteins, the GNA domain is accompanied by an S-locus glycoprotein, known for its role in self-incompatibility [71] and a PAN domain, which is believed to play a role in protein-protein/carbohydrate interactions [72]. The origin of G-type LecRLKs containing an S-locus glycoprotein and a PAN domain was analyzed in Brassicaceae, where this type of protein architecture is abundant and well-studied. It seems that two gene fusion events in the common ancestor of land plants most likely resulted in an ancient precursor [73]. Variations on this protein architecture that lack the PAN and/or S-locus glycoprotein domain are not restricted to soybean and almost all the architectures found in the soybean genome, have also been identified in either *Physcomitrella patens*, *Selaginella moellendorffii*, *Oryza sativa*, *Arabidopsis thaliana* or *Populus trichocarpa*, confirming the wide distribution of these types of proteins [73]. So far, no G-type LecRLKs from soybean have been studied in detail. However, a protein with a GNA domain, S-locus glycoprotein and protein kinase domain from wild soybean (*Glycine soja*) was shown to be involved in abiotic stress. Transcript levels largely increased upon abscisic acid, salt and drought treatment [74]. All identified types of LecRLK could be considered as plant defense related proteins as they might act as a receptor at the level of cell wall/plasma membrane of the plant cell during pathogen attack. However, the functionality of the lectin domains has to be investigated in more detail [8].

Overall the high diversity of domain architectures within the lectin family in soybean could be explained by the high rate of retention of duplicated genes after WGD events. Proteins containing multiple protein domains are generally a combination of preexisting domains by fusion, fission or terminal loss, rather than a creation of novel domains [75,76]. Single-domain proteins are therefore more likely to be shared by different plant species. The longer a domain arrangement, the more likely it is species-specific [76].

Strikingly, in some families there are tandem arrayed lectin domains. The occurrence of two Nictaba domains was already observed in rice [25] while the presence of two tandem arrayed LysM domains can be found across kingdoms (including prokaryotes, green algae, mosses, gymnosperms and angiosperms) [56]. The protein containing two legume domains is probably an exception since it concerns two incomplete legume domains. Jacalin-like lectin sequences containing three jacalin motifs have also been identified in the seed of *Parkia platycephala*, the most primitive subfamily of the Leguminosae [77].

**Figure 9:**
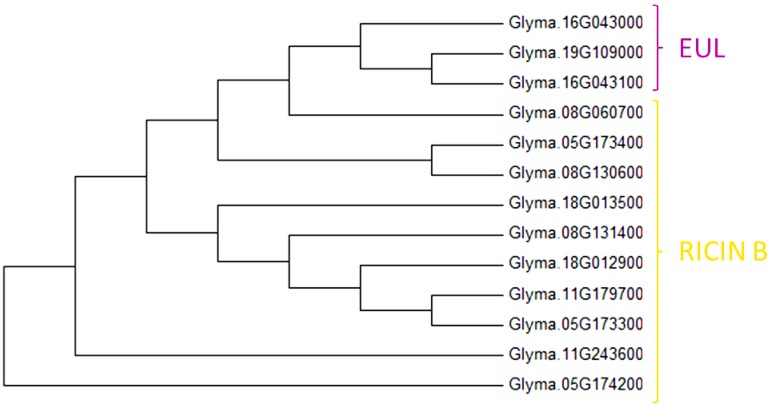
Evolutionary relationship between EUL and ricin B homologs from soybean.

The classification of lectins in twelve different families has been the subject of a continuous debate. Especially the evolutionary relationship between EUL and ricin B lectins is striking. During BLAST searches and in the Interpro database, EUL lectins are typically annotated as ricin B domain containing lectins, based on the shared Q-X-W motif in their amino acid sequences. Recent molecular docking studies of the *Euonymus europaeus* lectin also revealed that this lectin adopts the ricin B fold [78]. However, BLASTp searches show no significant sequence similarity between EUL and ricin B lectins. The phylogenic relationship between EUL and ricin B lectins from soybean was investigated (Figure 9) and showed that the three EUL homologs clearly cluster together in a specific branch of the dendrogram, separated from the ricin B homologs. These data justify that EUL and ricin B lectins are classified in distinct lectin families.

Another point of discussion concerns the lectin homologs of class V chitinases. This family was named after the first identified member (RobpsCRA) which shared 50% sequence identity with plant class V chitinases [27]. Class III and V plant chitinases are grouped together in the GH family 18 and other chitinase-like lectins belonging to the GH 18 family have been reported. For instance, a GlcNAc binding lectin from *Parkia platycephala* is homologous to the class III chitinases and does retain its chitinase activity [79]. In contrast, TCLL is a recently identified class III chitinase-like lectin from *Tamarindus indica* without chitinase activity [80]. It is clear that these chitinase like proteins should be categorized in the same lectin family of chitinase-related lectins and subdivisions can be made to address the chitinase activity. This would make it easier to study the relationship of related lectins and their physiological role.

With respect to the sugar-binding specificity of the different lectins, conclusions should be drawn thoughtfully. Several studies highlighted the promiscuity of the carbohydrate-binding site for different homologs of the Nictaba, EUL, legume, jacalin and GNA family [81,82,83,84,85]. The diverse carbohydrate specificities within different lectin families make it difficult to predict the biological properties of the lectins. Therefore even proteins with homologous lectin domains can have different functions due to carbohydrate-binding specificity and the presence of additional protein domains.

Despite the identification of 359 putative soybean lectin genes, only a few of them have been studied in detail. However, transcriptome data is available for the majority of the identified lectin genes in soybean, indicating that the genes are expressed during soybean development. In the early days, lectin research mainly focused on lectins that were abundant in seeds or vegetative storage tissues, mainly because biochemical research involving the purification and characterization of lectins was limited by the experimental tools available at that time. Only recently evidence became available to show that there are also weakly expressed lectins in non-storage tissues of the plant. Furthermore, some lectins can only be detected after the plant has been subjected to certain stress conditions, which makes them even more difficult to discover. The presence of 359 lectin genes in the soybean genome, belonging to nine different lectin families urges to adapt the idea on the occurrence of lectins and confirms that multiple lectins are present in the same species.

It can be concluded that the whole group of lectins in soybean is highly diverse (size of the protein, domain architecture, and sugar-binding specificity) and mainly expanded through tandem and segmental duplications. Furthermore it can be envisaged that the soybean plant succeeded in evolving a complex set of lectin genes encoding proteins with different localizations in the plant cell and biological function. It can be hypothesized that the concerted action of all these lectins can help the plant to protect itself against different environmental stresses, including the attack from different pathogens and predators [86,87,88].

## 4. Materials and Methods

### 4.1. Identification of Lectin Genes in the Soybean Genome

Protein sequences encoding *Agaricus bisporus* agglutinin (UniProtKB/Swiss-Prot: Q00022.3—ABA), *Amaranthus caudatus* agglutinin (GenBank: AAL05954.1—amaranthin), *Robinia pseudoacacia* chitinase-related agglutinin (GenBank: ABL98074.1—CRA), *Nostoc ellipsosporum* agglutinin (UniProtKB/Swiss-Prot: P81180.2—cyanovirin), *Euonymus europaeus* agglutinin (GenBank: ABW73993.1—EUL), *Galanthus nivalis* agglutinin (UniProtKB/Swiss-Prot: P30617.1—GNA), *Hevea brasiliensis* agglutinin (GenBank: ABW34946.1—hevein), *Artocarpus integer* agglutinin (GenBank: AAA32680.1—JRL), *Glycine max* agglutinin (UniProtKB/Swiss-Prot: P05046.1—legume lectin), *Brassica juncea* LysM domain (GenBank: BAN83772.1—LysM), *Nicotiana tabacum* agglutinin (GenBank: AAK84134.1—Nictaba) and the lectin chain of the *Ricinus communis* agglutinin (GenBank: PDB: 2AAI_B—ricin B), representing one member of each lectin family, were used individually to perform BLASTp (E value < 0.0001, comparison matrix: BLOSUM62, word length: default) searches [89] against the soybean genome (assembly *Wm82.a2.v1*) available from the Phytozome v10 website (http://phytozome.jgi.doe.gov/) [90]. All retrieved sequences were selected as candidate lectin genes and the top hit was used for a second BLASTp search to obtain more candidate sequences. The amino acid sequences from the candidate lectin genes were downloaded with the BioMart tool from Phytozome v10. All these protein sequences were scanned for the presence of conserved lectin domains using InterproScan5 (http://www.ebi.ac.uk/interpro/download.html) [91] with default settings. The program was locally installed and combines the following databases: PROSITE, HAMAP, Pfam, PRINTS, ProDom, SMART, TIGRFAM, PIRSF, SUPERFAMILY, CATH-Gene3D and PANTHER. The lectin domains corresponding to the EUL, the Nictaba and the CRA family were identified by sequence alignment using Clustal Omega (http://www.ebi.ac.uk/Tools/msa/clustalo/) [92] since no Pfam ID is available for these carbohydrate-binding lectin domains. Only those sequences containing at least one lectin domain were retained. The SignalP 4.1 server (http://www.cbs.dtu.dk/services/SignalP/) [93] was used to check the presence of signal peptides [94] and the TMHMM Server v.2.0 (http://www.cbs.dtu.dk/services/TMHMM/) provided information about possible transmembrane regions [95]. Predicted transmembrane regions at the N-terminus of a sequence were double-checked manually since these could give false positive results due to the presence of a signal peptide.

### 4.2. Construction of Chromosome Map

The MapChart software [26] was used to map all the putative lectin genes on the different chromosomes. The data file containing the gene name and transcript start position were downloaded from the Phytozome v10 website (http://phytozome.jgi.doe.gov/) and used for the construction of the chromosome map. The position of the centromers was retrieved from the Soybase website [96].

### 4.3. Analysis of Lectin Gene Expansion

Segmental and tandem duplication were traced to define the degree of lectin gene expansion within the soybean genome. Tandem duplicated genes were assigned as one or more surrounding genes, (1) belonging to the same lectin family, (2) with no more than ten intervening genes and (3) present on the same chromosome. Identification of segmentally duplicated chromosome blocks was possible through the Plant Genome Duplication Database (PGDD) [46]. Collinear blocks within the soybean genome were determined by McScan version 0.8 (http://chibba.agtec.uga.edu/duplication/index/files) and the output data was downloaded and searched for the presence of lectin genes. Two duplicated genes with a Ks (synonymous substitution) value higher than 1.0 were excluded because of the risk of saturation [97]. For this reason, 32 duplicated genes were excluded from the dataset.

### 4.4. Sequence Alignment and Phylogenetic Analysis

Sequence alignment and phylogenetic tree construction was conducted using the Molecular Evolutionary Genetics Analysis (MEGA) 6.0 program [94]. The amino acid sequences of the lectin domains were aligned using Clustal Omega [92] and used for constructing a Maximum Parsimony phylogenetic tree and dendrogram using the Max-mini branch-and-bound algorithm.

## Supplementary Materials

**Table S1**: Lectin gene names, chromosome positions, annotated protein domains and protein sequences of the identified lectin homologs in soybean.

**Table S2**: Contribution of tandem and segmental duplication events to the expansion of the lectin families.

Supplementary materials can be accessed at: http://www.mdpi.com/1420-3049/20/02/2868/s1.
