# Supplementary material for: Distribution and Evolution of the Lectin Family in Soybean (Glycine max)
Source: Molecules. 2015 Feb 11;20(2):2868–91. doi: 10.3390/molecules20022868 (PMC6272470; doi:10.3390/molecules20022868)
Supplement: Supplementary file 1 [file molecules-20-02868-s001.pdf]

Supplementary Materials

Table S1. Lectin gene names, chromosome positions, annotated protein domains and protein sequences of the identified lectin homologs in soybean.

Lectin family

```
>gene name|transcript name|chromosome position|protein domain(s)
amino acid sequence
```

CRA

```
>Glyma.13G155800|Glyma.13G155800.1|27091569|CRA
MASLSMLRFLFLAIFFVGITATTSTNDIKAIYWLEQPLFPPSAINTSLFTHVYYAFLAPN
NVTYELDVVSNSTGTNLATFTNTLRSNVATLISIGGANSNSTLFSLIAANAAARATFINS
TITVARTFGFNGIDLDWEFPRTVNEMNDLGLLFKEWRAAVSAEAAATGREPLLLTAAVYF
SVDYFLSETTSLRYPVDSINENLDWVNVMSYDLNGPWSNHTGPPAGLFDPKNNASVSYGL
GSWIRGGVIPKKVVMGLPLYGRTWQLLEPNVHGIGAPASGPGPGSNGAMALFQVLEFNNE
TGANVVCDKETASVYSYSGSYWVGYDDPKTVAVKVGFAQALSLHGYFFWAAGLDTSDWKI
STQALNAWMLCIEETGGMN*

>Glyma.15G206400|Glyma.15G206400.1|29177838|CRA/protein kinase
MVSRITIALVLFEFLLCQEFEPLKAQTWLQAGYWYSGSGFPVSDINSALYTHLICAFAEL
NSSTYELYVSPEDEQSFSSFTTTVKQKNPSITTLLSIAGGNGNDTVLSLMVSKDSSRKYF
IQSSIRIARLYGFQGLDLSWVPETISDMNNMGRLFEEWRAAAKSEAANDSTQVLILTAAV
HFRPGLDSASYPVESIQNNLNWVHILTYDYHMPQLANFTAAHAALYDPSSSVNTDNGIKE
WIGSGVTASKLVLGLPFYGYAWNLRNPEDNAIGASATGPAIGKSGAMNYKDIKAYIQRYG
GHVKYNATYVVNYFSNGSTWIGYDDVEVVKMKVSYARENKLLGYAVWQVPYDDNWVLSSA
AAEHVDQNGRNSWRLLVIILIITAMSVILLGILIYYLRRRFPKSTAVILSTLNNVNKDAS
RLFHSNAPDLQVFSFSDIEQATNRFSIENKVGQGGYGPVYKGILSNRQEVAVKKLSKAST
QGFEEFKNEVMLTARLQHVNLVRLLGFYIDGEQQMLVYEYMPNKSLDSYLFDPIRRYLLD
WRKRIYIIEGITQGLLYLQEYSRLTIIHRDIKASNILLDNEMKPKISDFGMARIFRKDEL
EANTSKIVGTYGYVSPEYAMKGLYSTKSDVYSFGVLLLQIVSGRRTACFYGEHENLNLME
YAYELWKEGKGMEFADPSLDDSHSTCKLLRCMQIALLCVQEDANDRPTVKEISSMLKSDT
ILIIPQKPAFSINRDEKKPNKFIMHEEKCSINDATISQVVAR*

>Glyma.15G206800|Glyma.15G206800.1|29340004|CRA
MANSKNNLILVSTLLMLLQLHFTPSKAAIKGGYWYSESGLAVSNINPSHFTHLFCAFAHL
DPNTNKVTISSSDSSQFSTFTQTLQAKNPSVKTLLSIGGGFGPSLAANFSRMARQANTRK
SFIDSSIQQARSNNFLGLDLDWEYPSSDTDKTNFASLIKEWKEAVTKESRTSGKAPLFLS
AAVAGSDQITPLKYYPGKDVANNLDFVNVMAYDLFTSEGYPTVTQPPAPWNNPRGQFSAE
QGVTEWNKTLGVPLNKLNLGLPFYGYKWSLSDSNKNGLFAPAKQGLGAVKYKDIKNVAAQ
VVFDSTYVTNYCFKGTDWFGYDDTQSISAKVVNAKQKGLVGYFAWHIEQDSNWALSQAAS
QAWGM*

>Glyma.17G076100|Glyma.17G076100.1|5959648|CRA
MPMAYSKSFSFLISILLFIFHNFHVSTAQKIKGGYWFPGSSFAVSDINSTLYTHLFCAFA
DLNASTYQVTISSSNAPQFSTFTQTVQKKNPSVKTLLSIGGGASNPSTFSAMASQAGRRK
TFIDSSIQLARSNNFHGLDMDWEYPSTSTDMTNYGFLIREWRTALVNEARNSGKQILLLV
GAVFYSSNYYSLNYTIQDVINAFDFVNVMAYDFYGPNWYPNFTAPPAALYALNHPAANRV
SGDMGIRDWIGSGMPASKLVLGVPFYGYAWRLLNSNNRGLFDRANGSAFGGDGSMGYSQI
REFVSQNRAACTFNSTVVSDYCSSGTTWIGYDDVQSVSAKVAYAKTNNLGGHFVWHVGAD
YNWVLSQAASRTWG*

>Glyma.17G103500|Glyma.17G103500.1|8127907|CRA
MASLSMLRFLFLATLFVITATSSTNDVKAIYWLEQPLFPPSAINTSLFTHVYYAFLAPNN
VTYKLHVSNSTATNLAKFTTTLGSTVATLISIGGANSNATLFSLIAANSAARATFINSTI
TLARTFGFHGVDLDWEFPRTASEMKNLGLLFKKWRAAIAAEAAATGREPLLLTAAVYFSV
DYFLSETSSLRYPVGSINENLDWVNVMSYDLNGPWSNHTGPPAGLFNPKSNASVGYGLGS
WIRGGVIPKKVVMGLPLYGRTWQLRDRSVHGIGAPTIGPVPGSDGVMALFQVLQFNKETG
AKVVYDRETASVYSYSGSYWIGYDDPISVAVKVGFAQAFSLRGYFFWAAGLDTSDWKIST
QALNAWMLCIKETGRMN*

>Glyma.17G217000|Glyma.17G217000.1|36553695|CRA
MINMALQRLSITLLLVLFMSLHSQASVESSNWKYAPNIGSVRAAYWPAGDDLSPSSIDTK
YFTHIYYAFIQQDPQLFHLSVTEFDEKWIPKFINGLRYRYPPVKTLLSIGGGGSNSTAFS
LMASNKHTRQVFINSTIHVARQYGFNGLDLDWEFPDDEHDMSNLGVLFQEWHQALVVEAK
ISRKPRLLLTAAVYYASTIKLIGDGPRSYPAQAIRNYLDWASPMCFDYHGTWDNFTGFNA
```

ALYDPKSNISTQYGIGSWIESGVPTQKLVMGLPLYGRAWALRDPNVNGVEAEAVGKATDT
DGTMDYDEILVFNKENGATVVYDDVAVAFYSYAGTTWIGYDDGPSIKKKVQFARFTGLKG
YFFWAVGKDKDWTISRQASNAWRH*

EUL

>Glyma.16G043000|Glyma.16G043000.1|4024940|EUL
MGGSFKNYDNQSHEVSEEESENYPYHHHAPLVMHVNVNNNSHSNIATTTSALSNKRSTVR
ILCKAAPNFSLSIRDGKVILAPADPSDAYQHWYKDERYSSMVKDEEGYRAFCLVNKATNE
VMKHSIADSHPVRLVPYNPEYLDESIMWTEASDLGNGYRAIRMANNIHLNMDALKGIKEL
GGVQDGTIVALWKWNDGDNQKWKVVPYGDEDSAIDADHSLMRRLDRLELNMQAMFDAQQK
YLEGLSKRFGNEKLSGY*

>Glyma.16G043100|Glyma.16G043100.1|4028980|EUL
MSFPFNHSHRRDDDDDDDDKRPAVYPPPGNTFNNPPPPFYGVHQPETHVSHVFNNSNYSA
PPPPPPSHNHHHDSFTHGGGSTVHHTPHNNAPPTTSVHHLSHQVNTGVLSSKPTVRVFTK
ANPNFSLTIRGGQVILAPSDPTNEYQHWYKDEKYSTRVKDEEGCPAFSLINKATGEALKH
SIGATHPVRLIPYKPDYLDESILWTESRDLGDGHRAIRMVNNVHLNVDAFHGDKNSGGVR
DGTTIVLWDWNKGDNQQWKILPY*

>Glyma.19G109000|Glyma.19G109000.1|36170931|EUL
MSFPFNHSHATHTHHRRDDDDDDERPTVYPPPGNSFSNPPPPFYGVPQPPPPQPETHVFHS
AHVTPGHVSHDFNNYSAPPPPPPPSHHHHDRHHDSFTHAYPPAPAAADYPAHGGSSTTVHH
VAHESHHHTPHSAFSSHNNAPPTTVHHVSHQVNTGGLSTKPTVRVFTKANPNFSLTIRRG
QVILAPSDPTNEYQHWYKDEKYSTRVKDEEGCPAFSLINKATGEALKHSIGATHPVRLIP
YKPDYLDESILWTESRDLGDGHRAIRMVNNVHLNVDAFHGDKNSGGVRDGTTIVLWDWNK
GDNQRWKILPYLKLTGFVSGFEDALS*

GNA

>Glyma.01G117900|Glyma.01G117900.1|40636182|GNA/S-locus glycoprotein/PAN/protein
kinase
MKFILFLISMIVYILFFPFLIVFTAAETSSITQSQSLSYRKTLVSPSGIFELGFFNLGNP
NKIYLGIWYKNIPLQNIVWVANGGSPIKDSSSILKLDSSGNLVLTHNNTVVWSTSSPEKA
QNPVAELLDSGNLVIRDENGGNEDAYMWQSFDYPSNTMLQGMKVGWDLKRNFSTRLIAWK
SDDDPTQGDLSWGIILHPYPEIYMMKGTKKYHRLGPWNGLRFSGFPLMKPNNHIYYSEFV
CNQEEVYFRWSLKQTSSISKVVLNQTTLERQRYVWSGKSWILYAALPEDYCDHYGVCGAN
TYCTTSALPMCQCLKGFKPKSPEEWNSMNWSEGCVRKHPLSCKNKLSDGFVLVEGLKVPD
TKDTFVDETIDLKQCRTKCLNKCSCMAYTNSNISGAGSGCVMWFGDLFDIKLYPENGQSL
YIRLPASELEFIRHKRNSIIIIVTSVAATLVVMVVTLAIYFIRRRKIADKSKTEENIERQ
LDDMDVPLFDLLTVTTATNNFSLNNKIGQGGFGPVYKGELVDGREIAVKRLSTSSGQGIN
EFTAEVKLIAKLQHRNLVKLLGCCFQGQEKLLIYEYMVNGSLDTFIFDKVKGKLLDWPRR
FHIILGIARGLLYLHQDSRLRIIHRDLKASNVLLDEKFNPKISDFGTAKAFGGDQIEGNT
KRVVGTYGYMAPEYAVAGLFSIKSDVFSFGILLLEIVCGIKNKALCDGNQIHSLAWTLWK
EKNALQLIDSSIKDSCVISEVLRCIHVSLLCLQQYPGDRPTMTSVIQMLGSEMELVEPKE
LSFFQSRILDEGKLSFNLNLMTSNDELTITSLNGR*

>Glyma.01G206300|Glyma.01G206300.1|53862540|GNA/protein kinase
MVVMFVHGNITLSSTLSTNDYDAWLSPSGEFAFGFHQLNGTNLFMTIAWNTKANEILATA
TTGSQVQLTSEGLTLTSAMLDIGNFVLVNKNSTFEWESFKNPTDTLLPNQSLELDGKYYY
RIDASHSASRLVFDELGNIYVETANGTRIQPQGPTWGYSTLAPKGSTNAHQGWTIMRYVP
DDICTTIFNENGSGSCGYNSYCSMKHDRPTCKCPYGYSMVDPSNEFVKAQTEELYEMHEF
RNFNFPLGDYEKKQPYSQQECRQSCLHSGNTCWMKRLPLGNGRQVAVSDEHFPKLTRLVP
ATPSLLETNLHSFTYETLEKATRGFSEEIGRGSSGVVYKGQLEAASCNLMIAIKRLDRLA
QERDKEFRTELSAIGKTSHKNLVRLIGFCDEGIHRLLMYEFMRWLVYLHEECDAPIIHCD
IKPKKVLLLADQSKTDTMIRRGYVPPEWFKNVPVMVKVDVYSFGVMLEIIWCRSVLMMDS
GEEEKAILTDWAYDCYIEGRNVDALVKNDEEAFSDSCRLHKWIKIALMRDSYL*

>Glyma.01G206400|Glyma.01G206400.1|53864760|GNA/S-locus glycoprotein//protein
kinase
MVAIWYNMIPDDQTVVWSARKDNKLATAPAGSKLQITQEGLSLTNPKGDFIWTASSKDFV
SEGAMLDSGNFVLLNGSSANVWQSFEHPTDTLLPNQSLQLGGMLTSRLTDTNYTTGRFQL
YFDGGNLLLSPLAWPSQLRYKSYPVIDASGNASRLLFNISGDIYVETTNGNRIQPQGQKW
VSNSSSSLDLNPEMNFYRATLDPSGVFTQYAHPRNNTARQGWIIMRYVPDDICNIIFDRF
GSGSCGYNSYCDMENERPTCNCLDGYSLVDPSNQFGGCQPNFTLACGADVQAPPEQLYHM
LQSSRYNFPEADYEKIQPYTQQECLQFCLHDCMCAVAIFGLDTCWMKRLPLSNGRVTDVN
DHHFVYIKIRNSRDFYPGVNEELPPGADSNKEDGAKPILMGSLIGSLVVNGILLATVALL
VLLKPKLKVAVPVAAASLLETNLHSFSYEALKEATWGFSEELGRGSCGIVYKGKLEAEDS
CNVIAVKRLDRLAQEREKEFRTELSAIGKTSHKNLVRLIGFCDQGINRLLVYEFMSNGTL

ADILFGHSKPNWNTRVGFALGIARGLVYLHEECDTPIIHCDIKPQNILIDEHFNTKISDF
GLAKLLLSDQSRTNTMIRGTRGYVAPEWFKNVAVTVKVDVYSFGIMLLEIICCRRSVVME
EPGEEEKAVLADWACDCYMEGRIDALVENEEEALSDKERLQKWIKIAIWCIHENPEMRPT
IGMVVQMLEGFVQVSNPPPTFTMHSVS*

>Glyma.01G206500|Glyma.01G206500.1|53866538|GNA/protein kinase
MVAIWYDKIPDKTVVWSAKTEYKLATAPTGSHVQITKEGLSLTSPEGDSIWRAKPEATVS
EGAMLNNGNFVLLNGGSEYENMWQSFDNPTDTLLPNQSLQLGLGGVLTSRFTDTNYTTGR
FQLYFQDFNVMLSPLAFPSQLRYNPYYHAINDASVGNASRLVFDKSGEIYVETTGGTRNR
ILPQVDNTLDTEVNYYRATLDFSGVFTLYAHPRNTSGQPRWRIMNYVPDNICDAIFNDYG
SGSCGYNSYCSMENDRPTCNCPYGYSLVDPSNESGGCQPNFTLACGADVQQPPEELYEMH
VAKNFNFPLGDYEKVEPYSQQECQQACLHDCMCAVAILEVDTCWMKRLPLGNGRQLPIRD
QHFVYIKTRLSPDFYPGLANRELPAAPDSKKENRAKSIILGSLIASLVVNSILLAAVALF
FLLKPKLKKVIQASALLETNLHSFSFEALKEATEDFCKELGRGSCGIVYKGKLETADSCN
VIAVKRLDRLAQEREKEFRTELSAIGKTSHKNLVRLIGFCDQGINRLLVYEFMSNGTLAD
ILFGHSKPIWNLRVGFVLGIARGLVYLHEECDSAIIHCDIKPQNILIDEHFNAKISDFGL
AKLLLFDQSRTNTMIRGTRGYVAPEWFKNVAVTVKVDVYSFGVMLLENICCRRSVMTMEP
EEEEKAILTDWAYDCCVEGRLHALVENDREALSDIGRLQRWVKIAIWCIQEDPEMRPTMG
KVNQMLEGLVEVANPPSPNPDI*

>Glyma.02G075600|Glyma.02G075600.1|6616676|GNA/S-locus glycoprotein/protein kinase
MFLKTQFLFLTLALATTTVTTAIDPGSTLAASSSNQTWSSPSGTFSLRFISVQPPTTPPS
FIAAIVFSGGAPVVWSAGNGAAVDSAGSLQFLRSGHLRLFNGSGATVWDTGTAGASSATL
EDSGNLVISNSTGSLWSSFDHPTDTLVPSQNFTVGKVLNSESYSFGLSSIGNLTLKWNNS
IVYWTQGLNSSVNVSLDSPSLGLLSIGLLQLSDANLSPSIDVAYSSDYAEGNSDVMRVLK
LDSDGNLRIYSTAKGSGVATARWAAVLDQCEVYAYCGNYGVCSYNDSTPVCGCPSENFEM
VDPNDSRKGCRRKASLNSCQGSATMLTLDHAVILSYPPEAASQSFFSGISACRGNCLSGS
RACFASTSLSDGTGQCVMRSEDFVSAYHNPSLPSTSYVKVCPPLEPNPPPSMGGVREKRS
RVPAWVVVVVVLGTLLGLIALEGGLWMWCCRNSTRFGGLSAHYALLEYASGAPVQFSHKE
LQQATKGFKEKLGAGGFGTVYRGTLVNKTVIAVKQLEGIEQGEKQFRMEVATISSTHHLN
LVRLIGFCSEGRHRLLVYEFMKNGSLDNFLFLTELHSGNFLNWEYRYNIALGTARGITYL
HEECRDCIVHCDIKPENILLDENYVAKVSDFGLAKLINPKDHRHRTLTSVRGTRGYLAPE
WLANLPITSKSDVYSYGMVLLEIVSGRRNFDVSEDTNRKKFSIWAYEEFEKGNISGILDK
RLAEQEVEMEQVRRAIQASFWCIQEQPSQRPTMSRVLQMLEGVTELERPPAPKSVMEGAV
SGTSTYFSSNASAFSTVGVSPAGPSSSSSFQTSVVSTFTLGRNPEKATSSLLQSDT*

>Glyma.02G182200|Glyma.02G182200.1|31198198|GNA/S-locus glycoprotein/protein
kinase
MLGQENCVLRSPFLQCIFIGFLMHSVVGAEIPLGSKLSVVENDCWVSSNGDFAFGFYNIS
DQPNQFSVGIRFNSKSIPYNQQTVVWVAGGDVKVGNKSYFELTQEGELVLFDSLGEVSVW
TVKTGNRSVASASLLDNGNLVLMDKEQRIIWQSFDTPSDTLLPGQSLFANEMLRAATASK
NSKASYYTLHMNASGHLELHWESGVIYWTSENPSASNLSAFLTAGGALELRDRSLKPVWS
AFGDDHNDSVKYRYLRLDVDGNLRLYSWVESLESWRSVWQAVENQCKVFATCGQIGVCVF
NASGSAECKCPFEVTGGNKCLVPYEGECESGSNMIAYKNTYLYAFYPPDNSFTTTSMQHC
EQLCLNDTQCTVATFSNDGTPQCSIKKTGYVTGYSDPSVSSISFVKRCSGPFAVNPGITK
SPPPSEPPPRLCVPCVIGASTGTFFTLVILQLGIGLFIYRRKNTTRKKSTLAFTGTNSKG
LIVLSFSEIKSLTGDFKNQIGPKVFKGLLPNNHLIAVKDLNASIEERKFRSAVMKMGCIH
HKNLVKLEGYCCEFDHRCLVYEYCKKGSVDKYIDDDALGRMLTWRKRVEICSSVAKAICY
LHSGCREFISHGNLKCKNVMLDENLVAKVTEFGFAIADGKATYCGFSAEKDIEDFGKLVL
TLLTGCCDHDHVKLCKWAYKEWMEERVANVVDKRMEGGYNSEELERTLRIAFWCLQMDER
RRPSMGEVVRVLDGSLSVDPPPSPFVFQRPLQVDYSEENVSELEG*

>Glyma.03G002700|Glyma.03G002700.1|257834|GNA/S-locus glycoprotein/PAN/protein
kinase
MASLLLPFLVSLIIFHNFQHTSSFSLSVEKDVIVSSPEGTFTAGFHPVGENAYCFAIWYT
QPPRTVVWMANRDQPVNGKRSTLSLLGVGNLVLTDADQFQVWSTNTLTSSKQVQLRLYDT
GNLVLLNNSNGFVLWQSFDFPTDTLLPNQPLRKTTNLVSSISGTNYSSGYYRLFFDFENV
LRLMYQGPRVTSVYWPFAWLQNNNFGNNGNGRSTFNDTRVVLLDDFGRVVSSDNFTFTTS
DYGTVLRRRLTLDHDGNVRLYSIKDGEDNWKVSGQFRPQPCFIHGICGPNSYCTNQPTSG
RKCICLPGHRWVDSEDWSQGCIPNFQPWCSNNSTEQESHFLQLPEMDFYGYDYALYQNHT
YQRCVNLCSRLCECKGFQHSYSKEGGDIGQCYLKTQLLNGHRSGGFSGAFFLRLPLSLQD
YDDRAILNNSNVLVCEGEVKVLERPYVEEKENAFVKFMLWFAIALGGIEFVIFFLVWCLL
FKNDADKEAYVLAVETGFRKFSYSELKQATKGFSDEIGRGGGGTVYKGLLSDNRVVAIKR
LHEVANQGESEFLAEVSIIGRLNHMNLIGMLGYCAEGKYRLLVYEYMENGSLAQNLSSSS
NVLDWSKRYNIALGTARGLAYLHEECLEWILHCDIKPQNILLDSDYQPKVADFGLSKLLN
RNNLDNSTFSTIRGTRGYMAPEWVFNLPITSKVDVYSYGIVVLEMITGRSPTTGVQITEI
EAKSPHHERLVTWVREKRKKGSEMGSSWVNQIVDPALGSDYDMNKMEMLATMALECVEEE
KDVRPTMSHVAERLQSHEHDS*

>Glyma.03G002900|Glyma.03G002900.1|270078|GNA/S-locus glycoprotein/protein kinase

MQKLRMATSPLSQLLLSLILLQNFHHSHSFSLSVENHNEDVIVSSPNATFTAGFYPVGEN
AFCFAIWYTRPPRTVVWMANRDQPVNGKRSTLSLLGTGNLELTDAGQFIVWSTNTATPSK
QNPRLHLYDTGNLVLIAILDNSEDHVLWQSFDFPTDTLLPNQPLSKSTNLVSSRSGTNYS
SGHYKLFFDFENVLRLMYQGPRVSSVYWPYAWLQSNNFGNGNGRSTFNDSRVVVLDDFGK
LVSSDNFTFTTIDSGTVVLRRRLTLDHDGNARVYSIRDGEDNWKVTGIFRPQPCFIHGIC
GPNSYCSNKPTTGRTCSCLPGYRWVDSQDWSQGCESSFQLWCNNTEKESHFLRLPEFDFY
GYDYGYYPNHTYEQCVNLCLELCECKGFQHSFSEKSDSTSQCYLKTQLLNGHHSPGFKGS
FSLRLPLSHDYDEKAILNNDNGLVCEGNSGGAKELERPYVEEKENGSVKFMLWFATALGG
IEIVCFFLVWCFLFRNNADKQAYVLAAETGFRKFSYSELKQATKGFSQEIGRGAGGIVYK
GVLSDDQVVAIKRLHEVVNQGESEFLAEVSIIGRLNHMNLIGMLGYCAEGKYRLLVYEYM
ENGSLAQNLSSSSNVLDWNKRYNIALGTARGLAYLHEECLEWVLHCDIKPQNILLDSDYQ
PKVADFGLSKLLNRNNLDNSTFSRIRGTRGYMAPEWVFNLPITSKVDVYSYGIVVLEMIT
GRSPTTGVQITELEAKSPHHGRLVTWVREKRKKGSEMGSSWVDQIVDPALGSDYDMNKME
MLATMALECVEEEKDVRPSMSHVAERLQSHEHDS*

>Glyma.03G003000|Glyma.03G003000.1|281581|GNA/S-locus glycoprotein/protein kinase
MASSTLLLALVILHNFQHSTQYSFSLSVENLKEDVIVSSPKATFTAGFYPVGENAYCFAI
WYTQQPHTLVWMANRDQPVNGKLSTLSLLKTGNLALTDAGQSIVWSTNTITSSKQVQLHL
YDTGNLVLLDNQQNRSSNIVVLWQSFDFPTNTLLPGQILTKNTNLVSSRSETNYSSGFYK
LFFDFENVLRLMYQGPRVSSVYWPDPWLQNNNFGNGGTGNGRSTYNDSRVAVLDDFGYFV
SSDNFTFRTSDYGTLLQRRLTLDHDGSVRVFSFNDGHDKWTMSGEFHLHPCYVHGICGPN
SYCSYEPSSGRKCSCLPGHTWVDSQDWSQGCTPNFQHLCNSNTKYESRFLRIPDIDFYGY
DYGYFGNYTYQQCENLCSQLCECKGFQHSFSEANAFFQCYPKTHLLNGNSQPGFMGSFFL
RLPLSSHDEYENPVQNNRSGLVCGGDVGNVKMLERSYVQGEENGSLKFMLWFAGALGGIE
VMCIFLVWCLLFRNNRTLPSSADRQGYVLAAAAGFQKFSYSELKQATKGFSEEIGRGAGG
IVYKGVLSDDQVVAIKRLHEVANQGESEFLAEVSIIGRLNHMNLIGMLGYCAEGKHRLLV
YEYMENGSLAQNLSSNSNVLEWSKRYNIALGTARGLAYLHEECLEWILHCDIKPQNILLD
SEYQPKVADFGLSKLLNRNNVNNSSFSRIRGTRGYMAPEWVYNLSITSKVDVYSYGIVVL
EMITGRSPTTGVRITELEAESDHRERLVTWVREKKMKGSEAGSSWVDQIIDPALGSNYAK
NEMEILARVALECVEEEKNVRPNMSQVVEKLQNHSMHTIVDGLNKE*

>Glyma.03G003100|Glyma.03G003100.1|288563|GNA/S-locus glycoprotein/protein kinase
MASSIFLLLHLLFPLIFHNFENSYSFSLSVENFKEKVIVSSPKATFTAGFYPIGDNAYCF
AIWYTTPPHTLVWMANRDRPVNGKRSMLSLLKTGNLVLTDAGQSIVWSTNTITSSKQVQL
HFYDTGNLVLLDNSIAVVLWQSFDFPTDTLLPGQTLSKNTNLVSSRSQTNYSSGFYKLFF
DSENVLRLMYQGPRVSSLYWPDPWLQSNDFGSGNGRLSYNDTRVAVLDHLGYMVSSDNFT
FRTSDYGTVLQRRLTLDHDGNVRVYSKKDVEEKWSMSGQFNSQPCFIHGICGPNSICSYD
PKSGRKCYCIKGYSWVDSQDWSQGCILNFQIFGNRTYEECENLCLGLSQCKGFQHRFWQP
DGVFICFPKTQLLNGYHTPGFTGSIFLRLPRNSPLSLSDSENPINYNNGFVCGGSNGGLK
LLDRPYVEEEENESVKLLLCFVTALGGIEVACIFLVWCFLFRNKNRKLHSGVDKPGYVLA
AATVFRKFSYSELKKATKGFSEAIGRGGGGTVYKGVLSDSRVVAIKRLHQVANQGESEFL
AEVSIIGRLNHMNLIDMLGYCAEGKYRLLVYEYMENGSLAQNLSSSSNALDWSKTYNIAV
GTAKGLAYLHEECLEWILHCDIKPQNILLDSDYKPKVADFGLSKLLNRNSNLDNSSFSRI
RGTRGYMAPEWVFNLPITSKVDVYSYGIVVLEMITGRSATAGTQITELEAESYHHERLVT
WVREKRKKGSEVGSCWVDQIVDPALGSNYERNEMEILATVALECVEEDKNARPSMSQVAE
KLQRYAHTS*

>Glyma.03G003300|Glyma.03G003300.1|300704|GNA/S-locus glycoprotein/protein kinase
MASSIFLILHLLLPLIFNNFENSYSFSLSVENFKEEVIVSSPKATFSAGFYPVGDNAYGF
AIWYTTTPHTLVWMANRDRPVNGKRSMLSLLKTGNLVLTDAGQSIVWSTNTITSSKQVQL
HFYDTGNLVLLDNSNAVVLWQSFDFPTDTLLPGQTLSKNTNLVSSRSQTNYSSGFYKLFF
DSENVLRLMYQGPRVSSLYWPDPWLQSNDFGSGNGRLSYNDTRVAVLDHLGYMVSSDNFT
FRTSDYGTVLQRRLTLDHDGNVRVYSKKDLEEKWSMSGQFKSQPCFIHGICGPNSICSYD
PKSGRKCSCIKGYSWVDSEDWSQGCVPNFQLRYNNNTEKESRFLHLPGVDFYGYDYSIFR
NRTYKECENLCLGLSQCKGFQHKFWQPDGVFICFPKTQLLNGHHTPGFTGSIFLRLPRNS
PLSLSDSENPINYNNGFVCGGSNGGPKLLDRPYVEEEENDSVKLLLCFVTALGGIEVACI
FLVWCFSFRNKNRKLHSGVDEPGYVLAAATVFRKFSYSELKKATKGFSEAIGRGGGGTVY
KGVLSDSRVVAIKRLHQVANQGESEFLAEVSIIGRLNHMNLIDMLGYCAEGKYRLLVYEY
MDNGSLAQNLSSSLNALDWSKRYNIALGTAKGLAYLHEECLEWILHCDIKPQNILLDSDY
KPKVADFGLCKLLNRNSNLDNSSFSRIRGTRGYMAPEWVFNLPITSKVDVYSYGIVVLEM
ITGRSATAGTQITELEAESYHHERLVTWVREKRKKGSEVGSCWVDQIVDPALGSNYERNE
MEILATVALECVEEDKNARPSMGQVAEKLQRYAHNS*

>Glyma.03G054900|Glyma.03G054900.1|7467093|GNA/S-locus glycoprotein/PAN/protein
kinase
MKFILFLMSIIVYILFFSSLIVFTAGETSSITQSQSLSYGKTLVSPSGIFELGFFNLGNP
NKIYLGIWYKNIPLQNMVWVANSSIPIKDSSPILKLDSSGNLVLTHNNTIVWSTSSPERV
WNPVAELLDSGNLVIRDENGAKEDAYLWQSFDYPSNTMLPGMKIGWDLKRNLSTCLVAWK
SDDDPTQGDLSLGITLHPYPEVYMMNGTKKYHRLGPWNGLRFSGMPLMKPNNPIYHYEFV
SNQEEVYYYRWSLKQTGSISKVVLNQATLERRLYVWSGKSWILYSTMPQDNCDHYGFCGAN

TYCTTSALPMCQCLNGFKPKSPEEWNSMDWSEGCVQKHPLSCRDKLSDGFVPVDGLKVPD
TKDTFVDETIDLKQCRTKCLNNCSCMAYTNSNISGAGSGCVMWFGDLFDIKLYPVPENGQ
SLYIRLPASELESIRHKRNSKIIIVTSVAATLVVTLAIYFVCRRKFADKSKTKENIESHI
DDMDVPLFDLLTIITATNNFSLNNKIGQGGFGPVYKGELVDRRQIAVKRLSTSSGQGINE
FTTEVKLIAKLQHRNLVKLLGCCFQEQEKLLIYEYMVNGSLDTFIFDQVKGKLLDWPRRF
HVIFGIARGLLYLHQDSRLRIIHRDLKASNVLLDENLNPKISDFGTARAFGGDQTEGNTK
RVVGTYGYMAPEYAVAGLFSIKSDVFSFGILLLEIVCGIKNKALCDGNQTNSLVGYAWTL
WKEKNALQLIDSSIKDSCVIPEVLRCIHVSLLCLQQYPGDRPTMTSVIQMLGSEMELVEP
KELGFFQSRTLDEGKLSFNLDLMTSNDELTITSLNGR*

>Glyma.03G079500|Glyma.03G079500.1|20189237|GNA/S-locus glycoprotein/PAN/protein kinase
MGSLTQVNYLIFLLILSSFYFGIISVNDTITSTRFIRDPEAIISSNGDFKLGFFSPEKST
NRYVAIWYLSETYIIWIANRDQPLNDSSGVFQIHKDGNLVVMNPQNRIIWSTNVSIIATN
TSAQLDDSGNLILRDVSDGKILWDSFTHPADVAVPSMKIAANRLTGEKIAYVSWKSSSDP
SSGYFTGSLERLDAPEVFFWFNKTKPYWRTGPWNGRVFLGSPRMLTEYLYGWRFEPNDNG
TAYLTYNFENPSMFGVLTITPHGTLKLVEFLNKKIFLELEVDQNKCDFYGTCGPYGSCDN
STLPICSCFEGFKPSNLDEWNRENWTSGCVRNMQLNCDKLNNGSDVQQDGFLEYHNMKVP
DFAERSINGDQDKCRADCLANCSCLAYAYDSYIGCMFWSRDLIDLQKFPNGGVDLFIRVP
AQLLVVAGGKKEKDYKGLIIGITLAIGALITAVTAYLLWRKFTPKHTGNQPQNLITGDQK
QIKLEELPLFEFEMLATATNNFHLANMLGKGGFGPVYKGQLDNGQEIAVKRLSKASGQGL
EEFMNEVVVISKLQHRNLVRLLGCCIERDEQMLVYEFMPNKSLDSFLFDPLQRKILDWKK
RFNIIEGIARGVLYLHRDSRLRIIHRDLKASNILLDDEMNPKISDFGLARIVRGGDDDEA
NTKRVVGTYGYMPPEYAMEGIFSEKSDVYSFGVLLLEIVSGRRNTSFYNNEQSLSLVGYA
WKLWNEDNIMSIIDPEIHDPMFEKSILRCIHIGLLCVQELTKERPTISTVVLMLISEITH
LPPPRQVAFVQKQNCQSSESSQKSQFNSNNDVTISEIQGR*

>Glyma.03G090200|Glyma.03G090200.1|26849526|GNA/protein kinase
MASSLLLFFLFCSVILLPFVVAQTKTNIAIGDSHTAGKSTTPWLVSSPSGDFAFGFLPLE
ATPDHFILCIWYANIQDRTIVWFANRDNKPAPKGSKVELSADDGLVLTAPNGDKLWNTGG
FTARVSSGVFNDTGNLVLLDGASSSTWESFDDYRDTLLPSQTMERGQKLSSKLRRNDFNI
GRFELFFQNDGNLVMHSINLPSEYVNANYYASGTIESNTSSAGTQLVFDRSGDVYILRDN
KEKYNLSDGGSISTTQFYLRATLDFDGVFTLYQHPKGSSGSVGWTPVWSHPDNICKDYLS
AASSGVCGYNSICSLGDYKRPICKCPKWYSLVDPNDPNGSCKPDFVQSCSEDELSQREDL
YDFEVLIDTDWPLSDYVLQKPFTEEQCRQSCMEDCLCSVAIFRLGDSCWKKKLPLSNGRV
DATLNGAKAFMKVRKDNSSLVVPPIIVKKNSRNTLIVLLSGSACLNLILVGAICLSSFYV
FWCKKKLRRVGKSGTNVETNLRCFTYEELEEATNGFEKVLGKGAFGIVYEGVINMGSLTL
VAVKRLNTFLLEEVQKEFKNELNVIGLTHHKNLVRLLGFCETQDERLLVYEYMSNGTLAS
LVFNVEKPSWKLRLQIATGVARGLLYLHEECSTQIIHCDIKPQNILLDDYYNARISDFGL
AKILNMNQSRTNTAIRGTKGYVALEWFKNMPITAKVDVYSYGVLLLEIVSCRKSVEFEAD
EEKAILTEWAFDCYTEGVLHDLVENDKEALDDMKTLEKLVMIALWCVQEDPGLRPTMRNV
TQMLEGVVEVQIPPCPSSQLSIQCSLD*

>Glyma.03G090300|Glyma.03G090300.1|26855486|GNA/protein kinase
MVSHQLATWPCSMVLLQIFVVAQTRSNKIIGHSLLKKTSKASWMVSPHGQSRRNIVVGDS
LLAKTTSNNNNPSSWVVSPLGDFAFGFVLLEDTNNFLVFIWYAKIPEKIVVWYTNGETPA
SKGSKVELTADDGLVLTALNGELLWKNDNHNGKDNGNFVLVDENHQGVLWETLKDPRDTL
LPSQSLEKGEKLSSRFLESNFSKGRFELLLQMDGILSIHALNSPFEYASENYYETRIEES
NTSSPGTRLVFEPLGYVYILRKNNERYNLSTWSRASTNESSFRETLNFDGIFTLYQHAKS
SSESDAWSPIWCFFCTLGGDQRSSCQCPKWYSLLDPNHPYGSCKPDFIQGCAEDELIGRK
EDVAEYDFEVLINTDWPLLDYVLLKPFTEEQCKQSCLEDWMCPVTIFRSVDVVGLNCAKT
FMKVRKDNSSLVVPQAKVNENSKSTLSYKKKHRGISKSEKALETNLHCFSYKELQRATNG
FQKELGRGSFGVVYERVINIGSAIPIAVKKLNNLLFQQVEKEFKNELHVIGLTHHKNLVR
LIGYL*

>Glyma.03G090500|Glyma.03G090500.1|26893241|GNA/protein kinase
MASSLLLFFLFCSVILLPFVVAQTKTNIAIGDSHTAGKSTTPWLVSSPSGDFAFGFLPLE
ATPDHFILCIWYANIQDRTIVWFANRDNKPAPKGSKVELSADDGLVLTAPNGDKLWNTGG
FTARVSSGVFNDTGNLVLLDGASSSTWESFDDYRDTLLPSQTMERGQKLSSKLRRNDFNI
GRFELFFQNDGNLVMHSINLPSEYVNANYYASGTIESNTSSAGTQLVFDRSGDVYILRDN
KEKYNLSDGGSISTTQFYLRATLDFDGVFTLYQHPKGSSGSVGWTPVWSHPDNICKDYLS
ATSSGVCGYNSICSLGDYKRPICKCPKWYSLVDPNDPNGSCKPDFVQSCSEDELSQREDL
YDFEVLIDTDWPSSDYVLQKPFTEEQCRQSCMEDCLCSVAIFRLGDSCWKKKLPLSNGRV
DATLNGAKAFMKVRKDNSSLVVPPIIVKKNSRNTLIVLLSGSACLNLILVGAICLSSFYV
FWCKKKLRRVGKSGTNVETNLRCFTYEELEEATNGFEKVLGKGAFGIVYEGVINMGSLTL
VAVKRLNTFLLEEVQKEFKNELNAIGLTHHKNLVRLLGFCETQDERLLVYEYMSNGTLAS
LVFNVEKPSWKLRLQIATGVARGLLYLHEECSTQIIHCDIKPQNILLDDYYNARISDFGL
AKILNMNQSRTNTAIRGTKGYVALEWFKNMPITAKVDVYSYGVLLLEIVSCRKSVEFEAD
EEKAILTEWAFDCYTEGVLHDLVENDKEALDDMKTLEKLVMIALWCVQEDPGLRPTMRNV
TQMLEGVVEVQIPPCPSSQLSIQCSLD*

>Glyma.03G090600|Glyma.03G090600.1|26899208|GNA/protein kinase
MVSPQLATWPCSMVLLQIFVVAQTRSNKIIGHSLLKKTSKASWMVSPHGQSRRNIVVGDS
LLAETTSNNNNLSSWVVSPLGDFAFGFVLLEDTNNFLVFIWYAKIPEKIVVWYTNGETPA
SKGSKVELTADDESVLTVPNGELLWKNDNHNGKDNGNFVLVDENHQGVLWETLKDPRDTL
LPSQPLEKGEKLLSRFLESNFSKGRFELLLQMDGILSIHALNSPSEYANENYCETRTEES
NTSSPGTRLVFEPLGYVYILRKNNERYNLSTWSGASTNESSFRETLNFDGIFTLYQHAKS
SSESDAWSPIWCFFCTLGGDQRSSCQCPKWYSLLDPNHPYGSSKLDFIQGCAEDELIGRK
EDVAEYDFEVLINTDWPLSDYVLLKPFTEEQCKQSCLEDWMCAVTIFRSVDVVGLNCAKT
FMKVRKDNSSLVVPPPNLSQKNDDLQLIHRWIIVKFEHHIHNPIPSILTVGNLDIMSDLR
EIPLYKKKLRGISKSEKALETNLHCFNYKELQRATNGFQKELGRGSFGVVYERVINIGSA
IPIAVKKLNNLLFQQVEKEFKNELQVIGLTHHKNLVRLIGYL*

>Glyma.03G138400|Glyma.03G138400.1|35488023|GNA/S-locus glycoprotein/PAN/protein
kinase
MVLITVQFQVGLTSLWCYIVNDARGNSLCLLIEFLSFGLFFKVFGQVGIQHFSLVNVTWA
IGFVDEPNQFSAGIRFNSKSIPYDQQTVVWVAGAHDKVSNMSYFQLTPEGELILFDSLKG
FIAWRSGTGNRAVASAALRDNGNLVLIDTKQNIIWQSFDTPSDTLLPGQSLSVYETLRAT
TKNPMSSSYTLYMNPSGQLQLRWDSHVIYWTSESPSSASNLTAFLTNGGALQLQDQSLKA
VWSVFGEDHNDSVNYRFLRLDVDGNLRLYSWIEASQSWRSVWQAVENQCKVFATCSQRGV
CIFTASGSTDCWCPFEVTESNQCLVPYEQECESGSNMLMYKNTYLYGIYPPDDSVVISSL
QQCEQLCLNDTQCTVATFSNNGRPQCSIKKTKYVTGYAVPSLNSISFVKRCSGPFAVNPG
LTKSPPPKLPRRLCVPCLMGAASGTFFIFAILQLGIIFIIFRRKNSTMRNVAIAFTSPNA
KGLNVFSFSEIKSLTGDLKDQIGPNMFKGVLPNNNHLIAVKDLNASIEERKFRSAVMKLGN
IHHKNLVKLEGYCCEFNHRFLVYEYVKIGSLHKYINDCTLCKRLTWRKRIEICSSVAKAI
CYLHTGCREFVSHGNLKCENVMLDENSVAKVCEYGFAIADGEATYRGFSAEKDVGDFGKL
ALTLFTGCLVHEQLYEWAYTEWMEGRAVNVVDKRLDGVVNSEELERALRISFWCLQMDER
RRPSMEEVVRVLDGTLNVDPPPPPFVLHRPLQEDDPQENGSD*

>Glyma.03G191300|Glyma.03G191300.1|40211543|GNA/S-locus glycoprotein/PAN
MLQTRHPQFLAIIPFLFLYTLTCSETATTTSIPQELHIGFSATAESSTTPFQAVLSDHSG
NFSLGFLRVNQNQLALAVLHVASSEPFWVANPTHAASWSDTTRLFFNGSLVLSDPETRVS
WSTATNNGDRVVLLNTSNLQVHDKGGTPLWQSFHFPANTLVQDQNFTSNMTLLSSNGIYS
MRLGNDFMGLYENHDSLYWKRTPLGAKAEVKEGQGPIYARVNPEGYLGMYQTSDEKPADV
QKFNTFQLTSSFLLLRLEPDGNLKGYYWDGSRWMLNYQAITEACELPRSCGSYGLCTPGG
SGCSCLDNRTRFEPGGCFNDASSGDADLCSSEGIGGKSSYWVLRRTGVEAAHKELLRHVT
TSSLAECEGLCQNNCSCWGALYSNETGFCYLLEYTIQTLLGTGDGSKVGYFKVKKEERRT
KRVWIRVGVVVTVLVGVGVIIIGVGFCVTRWKKKRGVKEEDWGSPGPYKNLESASFRSIE
MSNSNSANE*

>Glyma.04G042400|Glyma.04G042400.1|3423548|GNA/S-locus glycoprotein/protein kinase
MHLSTSREVPNQPQLIVHVSLYLFIPVLYAMRMVLLFLLLLFVPTSFGTIERLPEGSSLS
VEKQNDTIVSSNGDFSAGFFQVGDNAFCFSVWFTRSERPTVLWMANRDKPVNGRGSHLSL
WKDGNVVLTDAGGTIIWATATLSSSQQLHLKLRNNGNLVLLASKSTNTTIIWQSFDSPTD
TLLTLQPLTEQASLVSSRSTTNHSSGFYKLYFDNDNVLRLLYKGPTLSSVYFPEPWRLPM
DIGRSTYNVTKTAVLDSFGRFTSSDGFQFRSTDHPKKLFRRLTMDPDGNLRLYSFDEKLK
TWQVTWQLIPQPCTVHGICGANSACNYDRVVGRTCYCLKGFKVKDPNDWTQGCEPEFDPS
VFSCNSGESMGFLHYPTTELYGYDWNITVVNSLEECLNLCLELCDKCVAVQFKFNDVAKY
NCYPKTMVFNGRYTPNFDGEMYLKLPQAILGSSATPLNKHSTMNCTAGLSQQLERFYEAP
SRNSTLSFLVWFACGMGVFELSTIFLVWFFLFRTSKNSETVDQQRHLLSATGFQRFTYAE
LKSATKGFKEEIGRGAGGVVYKGVLYDDRVAAIKRLGEATQGEAEFLAEISTIGMLNHMN
LIDMWGYCVEGKHRMLVYEYMEHGSLAGNLFSNTLDWKKRFNVAVGTAKGLAYLHEECLE
WILHCDVKPQNILLDSDFQPKVADFGLSKLLNRDERGNSTFSRIRGTRGYMAPEWVYNLP
ITSKVDVYSYGIVVLEMVTGRSPMEIHSLENSRGIEQRRLVMWVTDKINDAPTSGFWIEE
ILDPNLEGQCQVSQVEVLVKVALQCVQDDMNQRPSMSQVVEMLLSHENNVLPR*

>Glyma.04G042500|Glyma.04G042500.1|3429721|GNA/S-locus glycoprotein/PAN/protein
kinase
MHQGSSLSVEEPKDVMLSPNAMFSAGFYAVGENAYSFAVWYSEPNGRPPNPTFVWMANRD
QPVNGKRSKFSLLGNGNLVLNDADGSVVWSTDIVSSSSAVHLSLDNTGNLVLREANDRRD
VVLWQSFDSPTDTLLPQQVFTRHSKLVSSRSETNMSSGFYTLFFDNDNVLRLLYDGPDVS
GPYWPDPWLAPWDAGRSSYNNSRVAVMDTLGSFNSSDDFHFMTSDYGKVVQRRLIMDHDG
NIRVYSRRHGGEKWSVTWQAKSTPCSIHGICGPNSLCSYHQNSGLKCSCLPGYKRKNDSD
WSYGCEPKVHPSCKKTESRFLYVPNVKLFGFDYGVKENYTLKECKELCLQLCNCKGIQYT
FYDTKGTYTCYPKLQLRHASSIQYFTDDLYLKLPASSSYSNEGSTDEQGLNCSSRTIKIE
RTYDKGHENRYVKFLVWFATGVGGLELLCAFVVWFFLVRTTGKQDSGADGRVYALAGFRK
FSYSELKQATKGFSQEIGRGAAGVVYKGVLLDDQRVAAVKRLKDANQGEEEFLAEVSCIGR
LNHMNLIEMWGYCAEGKHRLLVYEYMEHGSLAKNIESNALDWTKRFDIALGTARCLAYLH
EECLEWILHCDVKPQNILLDSNYHPKVADFGLSKLRNRNETTYSSFSTIRGTRGYMAPEW
IFNLPITSKVDVYSYGIVVLEMVTGRSITKDIEATDNGVVNQHLSMVTWLKERQKNGFTC
VSEILDPTVEGVYDEGKMETLARVALQCIEEEKDKRPTMSQVVEMLQESSRETHNR*

>Glyma.04G042600|Glyma.04G042600.1|3433939|GNA
MSCSHKKNGMFSAGFYAVGQNAYSFAVWFSEPYGQTRNATVVWMANRDQPVNGKDSKISL
LRNGNLALNDVDESLVWYTNTASLSSSVRLFFDNTGNLLLHETQATGVVLWQSFDFPTDT
LLPQQVFTRHSKLVSSRSETNMSSGFYALFFDNDNIFRLLYDGPEVSGLYWPDPCRVAVM
DTLDNFSSSDDLHFLTSDYGTLIQRRLVLDHDGNIRVYSQRHGGVGGLEVFCIFVIWFFL
VRTRGQKYSGVDGRVYNLAMSGFRKFSYSQLKQATKGFSQEIGRDAGGVVYKGVC*

>Glyma.04G066700|Glyma.04G066700.1|5567751|GNA/protein kinase
MEIHWPFFHITGTLFLLCKVCLAGIQYSGSVSPGIINGSQMNWIDRDGKFLVSKEGQFAF
AFVATANDSTKFLLAIVHVATERVIWTANRAVPVANSDNFVFDEKGNAFLEKDGTLVWST
NTSNKGVSSMELLDTGNLVLLGSDNSTVIWQSFNHPTDTLLPTQEFTEGMKLISDPSTNN
LTHFLEIKSGNVVLTAGFRTLQPYWTMQKDNRKVINKDGDAVASANISGNSWRFYGKSKS
LLWQFIFSTDQGTNATWIAVLGSDGFITFSNLNGGESNAASQRIPQDSCATPEPCDAYTI
CTGNQRCSCPSVIPSCKPGFDSPCGGDSEKSIQLVKADDGLDYFALQFLQPFSITDLAGC
QSSCRGNCSCLALFFHISSGDCFLLNSVGSFQKPDSDSGYVSYIKVSTVGGAGTGSGGSG
GGNKHTIVVVVIVIITLLVICGLVFGGVRYHRRKQRLPESPRDGSEEDNFLENLTGMPIR
YSYKDLETATNNFSVKLGQGGFGSVYKGALPDGTQLAVKKLEGIGQGKKEFRAEVSIIGS
IHHLHLVRLRGFCADGTHRLLAYEYLSNGSLDKWIFKKNKGEFLLDWDTRFNIALGTAKG
LAYLHEDCDSKIVHCDIKPENVLLDDHFMAKVSDFGLAKLMNREQSHVFTTLRGTRGYLA
PEWITNYAISEKSDVYSYGMVLLEIIGGRKNYDPRESSEKSHFPTYAFKMMEEGKLRDIF
DSELEIDENDDRFQCAIKVALWCIQEDMSMRPSMTRVVQMLEGICIVPKPPTSSSLGSRL
YATMFKSSSEEGATSSAPSDCNSDAYLSAVRLSGPR*
>Glyma.04G139100|Glyma.04G139100.1|22051371|GNA/S-locus glycoprotein/PAN/protein kinase/SRK
MESQKVLMLMMYTTLFCFMATFSSQDTVTITLNQSLQFSDTLVSLDGTFEAGFFNFENSR
HQYFGIWYKRISARTVVWVANRDVPVQNSTAVLKLTDQGNIVILDGSRGRVWSSNSSRIA
VKPVMQLLKTGNLVVKDGEGTKNILWQSFDYPGNTFLPGMKLKSNLVTGPYNYLTSWRDT
EDPAQGEFSYRIDIRGLPQLVTAKGATIWYRAGSWNGYLFTGVSWQRMHRFLNFSFESTD
KEVSYEYETWNSSILTRTVLYPTGSSERSLWSDEKQRWLTIATRPVDECEYYAVCGVNSN
CNINDFPICKCLQGFIPKFQAKWDSSDWSGGCVRRIKLSCHGGDGFVKYSGMKLPDTSSS
WFNKSLSLEECKTLCLRNCSCTAYANLDIRDGGSGCLLWFDNIVDMRNHTDRGQEIYIRL
DISELYQRRNKNMNRKKLAGILAGLIAFVIGLTILVWATSSFIKRMNLGKPEIIKKLIHW
KHMKETEENDIQTIFDFSTIDIATNHFSDRNKLGEGGFGPVYKGILEDGQEIAVKRLSKT
SRQGTEEFKNEVKLMATLQHRNLVKLLGCSIQQDEKLLIYEFMPNRSLDYFIFDTMRGKL
LDWTRCFQIIEGIARGLLYLHQDSTLRIIHRDLKTSNILLDINMIPKISDFGLARTFGGD
QAEANTNRVMGTYGYMPPEYVVHGSFSTKSDVFSYGVIVLEIISGRKNRGFRDPHHNHLN
LLGHVWRLWTEERPLELIDEMLDDDTTISSEILRRIHVGLLCVQENPENRPNMSSVVLML
NGGTLLPKPRQPGFYTGKDNTIDTGSCSKHHERCSVNEISISLLEAR*

>Glyma.06G043200|Glyma.06G043200.1|3296539|GNA/S-locus glycoprotein/PAN/protein kinase
MHQGSSLSVEEPKDFMLSPNGMFSSGFFAVGENAYSFAVWYSEPYGQTRNATVVWMANRD
QPVNGKGSKFSLLHNGNLALNDADESHVWSTNTVSLSSSVLLFLDNTGNLVLRQTESTGV
VLWQSFDFPTDTLLPQQVFTRHAKLVSSRSKTNKSSGFYTLFFDNDNILRLLYDGPEVSG
LYWPDPWLASWNAGRSTYNNSRVAVMDTLGNFSSSDDLHFLTSDYGKVVQRRLTMDNDGN
IRVYSRRHGGEKWSITWQAKARPCNIHGICGPNSLCSYHQNSGIECSCLPGYKWKNVADW
SSGCEPKFSMLCNKTVSRFLYISNVELYGYDYAIMTNFTLNQCQELCLQLCNCKGIQYTY
VFESGTYTCYPKLQLRNAYRTPYFNADLYLKLPANSSYSYEGSTEQHGLDCSSSRTIQLE
RAYDMGHESRYIKFLFWFVGGVGGIEVFCIFVICLFLVKTSGQKYSGVDGRVYNLSMNGF
RKFSYSELKQATKGFRQEIGRGAGGVVYKGVLLDQRVVAVKRLKDANQGEEEFLAEVSSI
GRLNHMNLIEMWGYCAERKHRLLVYEYMENGSLAQNIKSNALDWTKRFDIALGTARGLAY
IHEECLECILHCDVKPQNILLDSNYHPKVADFGMSKLIMRNRNDTSTYSNISRIRGTRGY
VAPEWVFNLSITSKVDVYSYGMVVLEMVTGKSVTKDVDATDNGVENLHLSMVAWLKEKDK
NGSGCVSEILDPTVEGGYDEGKMKALARVALQCVKEEKDKRPTMSQVVEILQKSSRENDH
Q*

>Glyma.06G068100|Glyma.06G068100.1|5213049|GNA/protein kinase
MGKHWSFFHITGTLFLLCKVCLAGSQYSGRVLPGVLNGSQMNWIDRDGKFLVSKKVQFAF
GFVTTTNDTTKFLLAIIHVATTRVIWTANRAVPVANSDNFVFDEKGNAFLQKDGTLVWST
STSNKGVSSMELLDTGNLVLLGIDNSTVIWQSFSHPTDTLLPTQEFTEGMKLISDPSSNN
LTHVLEIKSGNVVLTAGFRTPQPYWTMQKDNRRVINKGGDAVASANISGNSWRFYDKSKS
LLWQFIFSADQGTNATWIAVLGSDGFITFSNLNDGGSNAASPTTIPQDSCATPEPCDAYT
ICTGDQRRCSCPSVIPSCKPGFDSPCGGDSEKSIQLVKADDGLDYFALQFLQPFSKTDLA
GCQSSCRGNCSCLALFFHRSSGDCFLLDSVGSFQKPDSDSGYVSYIKVSTDGGAGTGSGG
GGGVHKHTIVVVVIVIIALVVICGLVFGGVRYHRRKQRLPESPREGSEEDNFLENLTGMP
IRYSYKDLEAATNNFSVKLGQGGFGSVYKGVLPDGTQLAVKKLEGIGQGKKEFRAEVSII
GSIHHLHLVRLKGFCADGTHRLLAYEYLSNGSLDKWIFKKNKGEFQLDWDTRFNIALGTA
KGLAYLHEDCDSKIVHCDIKPENVLLDDHFMAKVSDFGLAKLMNREQSHVFTTLRGTRGY
LAPEWITNYAISEKSDVYSYGMVLLEIIGGRKNYDPSKSSEKSHFPTYAYKMMEEGKLRD
IFDSELKIDENDDRFQCAIKVALWCIQEDMSMRPSMTRVVQMLEGICIVPNPPTSSSLGS

RLYATVFKSSSEGATSSGPSDCNSDAYLSAVRLSGPR*

>Glyma.06G110700|Glyma.06G110700.1|8945501|GNA/S-locus glycoprotein/protein kinase
MSQICTSSNYTMKPIITFSCILLFFTTISARSFSDYITPNFTASYLEFIDNFGTFLFSHN
RTFKAAIFNPGGQQTSFYLCVIHAASNTIIWSGNRDAPISDSGKMLLSFKGITILDEHGN
TKWSTPSLKSQVNRLQLTEMGNLVLLDKSNGSLWESFQNPTDTIVIGQRLPVGASLSSAA
SNSDLSKGNYKLTITSSDAVLQWYGQTYWKLSTDTRVYKNSNDMLEYMAINNTGFYLFGD
GGTVFQLGLPLANFRIAKLGTSGQFIVNSFSGTNNLKQEFVGPEDGCQTPLACGRAGLCT
ENTVSSSPVCSCPPNFHVGSGTFGGCEPSNGSYSLPLACKNSSAFSFLNIGYVEYFGNFY
SDPVLYKVNLSACQSLCSSNCSCLGIFYKSTSGSCYMIENELGSIQSSNGGDERDILGFI
KAITVASTTSSNDGNDDKENSQNGEFPVAVAVLLPIIGFIILMALIFLVWRRLTLMSKMQ
EVKLGKNSPSSGDLDAFYIPGLPARFDYEELEEATENFKTLIGSGGFGTVYKGVLPDKSV
VAVKKIGNIGIQGKKDFCTEIAVIGNIHHVNLVKLKGFCAQGRHRLLVYEYMNRGSLDRN
LFGGEPVLEWQERFDVALGTARGLAYLHSGCVQKIIHCDIKPENILLQDQFQAKISDFGL
SKLLSAEQSGLFTTMRGTRGYLAPEWLTNSAITEKTDVYSFGMVLLELVSGRKNCYYRSR
SHSMDDSNSGGGNSSTSSTTGLVYFPLFALEMHEQRSYLELADSRLEGRVTCEEVEKLVR
IALCCAHEEPALRPNMVTVVGMLEGGTPLPHPRIESLNFLRFYGRRYTEASTIAEENEYG
SVMLQQARSSTTSMPSDSSTRGFSYMSSQNISGPR*

>Glyma.06G255900|Glyma.06G255900.1|43736673|GNA/S-locus glycoprotein/PAN/protein kinase
MAFTSALTSLTTLVCLCMFCVNATTHKEILQTGQSLGTSDTLLSYGGNFELGFFSKDNST
KYYVGIWYKRVPNDKIVWVANRDSPVQTSSAVLIIQPDGNFMIIDGQTTYRVNKASNNFN
TYATLLDSGNLVLLNTSNRAILWQSFDDPTDTLIPGMNLGYNSGNFRSLRSWTSADDPAP
GEFSLNYGSGAASLIIYNGTDVFWRDDNYNDTYNGMEDYFTWSVDNDSRLVLEVSGELIK
ESWSEEAKRWVSIRSSKCGTENSCGVFSICNPQAHDPCDCLHGFQPLHADSWRNGNTSAG
CVRKIELSCSNRSSNNVKSNDGFFQFNKVQLPQTSNGYIKLKIDRARECESACSRNCSCV
AYAYYLNSSICQLWHGQVLSLKNISTYLDNSDNTNPIFYLRLDASELVTADSNPTNATEL
ATDFRKHENLLRNLLLIVILILLLAFLILGLLVYWTRRQRRKGEDLLRFHVSMSMKVEDS
ELAEAHRGAKVKKKEVKLPLFSFVSVAAATNNFSDANKLGEGGFGPVYKGILLNGDEVAV
KRLSRRSGQGWEELRNEALLIAKLQHNNLVRLLGCCIDRDEKMLIYELMPNKSLDVFLFD
ATKRRMLDWGTRVRIIDGIAQGILYLHQYSRFRIIHRDLKASNILLDTNMNPKISDFGMA
RIFGDNELQANTNRIVGTYGYMSPEYAMEGLFSIKSDVFSFGVLLLEILSGKKNTGFYQT
NSFNLLGYAWDLWTNNSGMDLMDPALDDSDTTSSSMHTVPRYVNIGLLCVQESPADRPTM
SDVVSMIGNDTVALPSPKPPAFLNVRGNQNSILPASMPESFSLNLITDTMVEAR*

>Glyma.06G256400|Glyma.06G256400.1|43823445|GNA/S-locus glycoprotein/PAN/protein kinase/SRK
MLFIWFCLFSYMTSTSTSTSLDSLAVSQSIRDGETLVSAGGITELGFFIPGNSARRYLGIWF
RNVSPFTVVWVANRNTPLDNKSGVLKLNENGILVLLNATNSTIWSSSNISSKTENDPIAR
LLDSGNFVVKNGEQTNENGVLWQSFDHPCDISMPEMKIGWNLETGVERYVSSWTSDDDPA
EGEYALKMDLRGYPQLIVFKGPDIKSRAGPFNGFSLVANPVPSHDTLPKFVFNEKEVYYE
FELLDKSAFFLYKLSPSGTGQSLFWTSQLRTRQVASIGDQDQCETYAFCGANSLCNYDGN
HPTCECLRGYVPKSPDQWNISIWVNGCVPMNKSNCENNDTDGFFKYTHMKLPDTSSSWFN
ATMNLDECHKSCLKNCSCTAYANLDVRDGGSGCLLWLNNLVDLRSFSEWGQDFYIRVSAS
ELDHAGHGNVKRKIVGITVGVTIFGLIISCVCIFIIKNQGTARKIYNKHYQNRLLRKEDI
DLPTFDLSVLANATENFSTRNKLGEGGFGPVYKGTLIDGKELAVKRLSKKSEQGLDEFKN
EVALISKLQHRNLVKLLGCCIDGDEKMLIYEFMPNHSLDYFVFDETKRKFLDWPKRFNII
NGIARGLLYLHQDSRLRIIHRDLKTSNVLLDANLHPKISDFGLARSFIGDQVEANTNRVA
GTYGYIPPEYAARGHFSLKSDVFSYGVIVLEIVSGKKNREFSDPEHYNNLLGHAWRLWTE
ERVLELLDELLGEQCAPFEVIRCIQVGLLCVQQRPEDRPDMSSVVLMLNSDTSLPKPKVP
GFYTEIDVTSDANSSSANQKLHSVNELSITILDAR*

>Glyma.06G256600|Glyma.06G256600.1|43863765|GNA/S-locus glycoprotein/PAN/protein kinase/SRK
MVQNFRMLFIWFLLLWYLRNSTSLDSLAVSQSIHDGETLVSEEGTFEVGFFSPGTSTRRY
VGIWYRNLSPLTVVWVANRENALQNNAGVLKLDERGLLVILNGTNSTIWWSNNTSSKVVK
NPIAQLLDSGNLVVRNERDINEDNFLWQSFDYPCDKFLPGMKLGWNLVTGLDRTITSWKN
EDDPSKGEYSMKLDLRGYPQVIGYKGDVVRFRSGSWNGQALVGYPIRPFTQYVHELVFNE
KEVYYEYKTLDRSTFFIVALTPSGIGNYLLWTNQTRRIKVLLFGESEPCEKYAMCGANSI
CNMDNSSRTCDCIKGHVPKFPEQWNVSHWYNGCVPRNKSDCKTNNTDGFLRYTDMKIPDT
SSSWFDKTMNLDECQKYCLKNCSCKAYANLDIRDGGSGCLLWFDDLIDMRHFSNGGQDLY
LRVVSLEIDFTAVNDKGKNMKKMFGITIGTIILGLTASVCTIMILRKQGVARIIYRNHFK
RKLRKEGIDLSTFDFPIIERATENFTESNKLGEGGFGPVYKGRLKDGQEFAVKRLSKKSG
QGLEEFKNEVVLIAKLQHRNLVKLIGCCTEGKERMLIYEYMQNKSLDYFIFDETRRNLVD
WPKRFNIICGIARGLLYLHEDSRLRIVHRDLKTSNILLDENFNPKISDFGLARAFLGDQV
EANTNRVAGTYGYMPPEYAACGHFSMKSDVFSYGVIVLEIVCGQRNREFSDPKHYLNLLG
HAWRLWTKESALELMDGVLKERFTPSEVIRCIQVGLLCVQQRPEDRPNMSSVVLMLNGEK
LILPNPKVPGFYTKGDVTPESDIKPANRFSSNQISITLLEAR*

>Glyma.06G256700|Glyma.06G256700.1|43889014|GNA/S-locus glycoprotein/PAN/protein kinase
MADNFRMLFIWLFLLLSYLRNSTSLDSLLPGQSIRDGETLVSEEETFEVGFFSPGTSTGR
YLGIWYRNVSPLIVVWVANRETPLQNKSGVLKLDERGVLVILNGTNSTIWWSYNTSSKVI
KNPIAQLLDSGNIVVRNEHDINEDNFLWQSFDYPCDKLLPGMKIGWNLVTGLDRTISSWK
KEDDPAKGEYSLKLDPKGFPQLFGYKGNAIRFRVGSWNGQALVGYPIRPLTEYVHELVFN
EKEVYYEYKTLDRSIFFIVTLNSSGIGNVLLWTNQTRGIQVFSLWSDLCENYAMCGANSI
CSMDGNSQTCDCIKGYVPKFPEQWNVSKWYNGCVPRTTPDCRNSNTDGFLRYTDLKLPDT
SSSWFNTTINLEECKKYCLKNCSCKAYANLDIRNGGSGCLLWFDDLIDMRKFSIGGQDIY
FRIQASSVLDHVPVNGHGKNKRMIGITVGANILGLTACVCIIMILKKLGVARIIYRNHFK
RKLRKEGIDLSTFDFPIIARATENFATSNKLGEGGFGPVYKGRLKDGQEFAVKRLSKKSG
QGLEEFENEVVLIAKLQHRNLVKLIGCCIEGNERMLIYEYMPNKSLDCFIFDETRRHLVD
WHIRFNIICGIARGVLYLHQDSRLRIIHRDLKTSNILLDANMDPKISDFGLARTFCGDQV
GANTNKVAGTYGYMPPEYATRGHFSMKSDVFSYGVIVLEIVSGKRNREFSDPTHSLNLLG
HAWRLWTEERALELLDGVLRERFIASEVIRCIQVGLLCVQQTPEDRPDMSPVVLMLNGEK
LLPNPKVPGFYTEGDVHLNQSKLKNPFSSNQISITMLEAR*

>Glyma.06G257000|Glyma.06G257000.1|43941660|GNA/S-locus glycoprotein/PAN/protein kinase/SRK
MLFIWFFLFSFMTRTSTSLDRLEVNQSIRDGETLVSAGGIIEVGFFSPGNSTRRYFGVWY
KNVSPLTVVWVANRNTPLENKSGVLKLNEKGIIVLLNATNSTLWSSSNISSKARNNATAH
LLDSGNFVVKHGHKTNSVLWQSFDYPGNTLMQGMKLGWDLETGLERSISSWKSVEDPAEG
EYVIRIDLRGYPQMIEFKGFDIIFRSGSWNGLSTVGYPAPVNLSLPKFVFNEKEVYYEFE
ILDSSVFAIFTLAPSGAGQRIFWTTQTTTRQVISTQAQDQCEIYAFCGANSICSYVDNQA
TCECLRGYVPKSPDQWNIAIWLGGCVQKNISNCEIRYTDGFLKYRHMKLPDTSSSWFNKT
MNLGECQKSCLKNCSCTAYANLDIRNGGSGCLLWFNILVDMRNFSLWGQDFYIRVPASEL
DDTGNRKIKKKIVGITVGVTTFGLIITCLCIFMVKNPGAVRKFYNKHYNNIKRMQDLDLP
TFNLSVLTKATRNFSSENKLGEGGFGPVYKGTLIDGKEIAVKRLSKKSVQGLDEFKNEVA
LIAKLQHRNLVKLLGCCIEGEEKMLIYEYMPNQSLDYFVFDETKRKFLDWGKRLNIIIGI
ARGLLYLHQDSRLRIIHRDLKTSNILLDENLDPKISDFGLARSFLGDQVEANTNRVAGTY
GYMPPEYAARGHFSVKSDVFSYGVIVLEIVSGKKNREFSDPEHYNNLLGHAWRLWTEQRS
LDLLDEVLGEPCTPFEVIRCIQVGLLCVQQRPEDRPDMSSVVLMLNCDKELPKPKVPGFY
TETDAKPDANSSFANHKPYSVNELSITMLDAR*

>Glyma.06G257100|Glyma.06G257100.1|43996701|GNA/S-locus glycoprotein/PAN/protein kinase
MLCIWFFLFSYMLGKCTLLDRLEMSQYIPDDGETLVSAGEITEMGFFSPGNSTRRYLGIW
YKNVSPFTVVWVANQNTPLENNFGVLKLNEKGILELLNPTNNTIWSSSNNISSKARTNPI
VRLLNSENLVKNGQGTKDDSFLWQSFDHPCDTYMPGMKVGWNLDTDLEWFLSSWKSVDDH
AKGEYALKIDLRGYLQIIKFKGIVIITRAGSWNGLSAVGYPGPTLGISPIFVFNKKEMSY
RYNSLDKSMFSIFYWTSQARNQQVVSIGEVDKCKNYAFCGANSVCNYNGNHPNCECLRGY
DPKSPGQWNVGIWFYGCVPRNKASCGNSYVDGFLKYMDMKLPDTSSSWFSKTMNLDKCQK
SCLNNCSCTAYANLDMRHGGNHDSHRNTKRKIVEIMVGVTIFGLIITWAARKFYIKHYKN
KQRTEDGDLPIFYFSVIANATENFSTKNKLGEGGFGPVYKATLIDGKELAVKRLSKKSGL
VCNSYAKTQAYMQCGTMSVKNLVRRLGVHDKTNHTLARQGLDEFKNEVALIVKLRHPNLV
KLVGCCIEEEKMLIYEYMSNRSLDYFIFDEAKRKLLDWRKLFNIICGSARGLLYLHQDSR
LRIIHRDLKTSNILLDTNLDPKISDFGLARSFLGDQVEANTNTVAGTYGYMPPGYAVSGQ
FSVKSDVFSYGVILLEIVSAKKNREFSDPESYNNLLGHGTELLDDVLGEQCTFREVIRCI
QIGLLCVQQRPGDRPEMSSVVLMLKGDKLLPKPKVPGFYTEKDVKFESNHNLCSVNELSI
TTLDGRQEGKVRKC*

>Glyma.06G257300|Glyma.06G257300.1|44030457|GNA/S-locus glycoprotein/PAN/protein kinase
MLFICFFIFFYMTTTSTSVDRLAVTQSIRDGETLASAGGIIEAGFFSPGNSIRRYLGIWY
RNVSPFIVVWVANRNTPLENKSGVLKLNEKGVLELLNATNNTIWSSNIVSSNAVNNPIAC
LFDSGNFVVKNSEDGVLWQSFDYPGDTLMPGIKLGWNLETGLERSISSWKSDDDPAEGEY
AIKIDLRGLPQMIEFKGSDIRMRTGSWNGLTTVGYPSPTPLLIRKFVVNEKEVYYEYEII
KKSMFIVSKLTPSGITQSFSWTNQTSTPQVVQNGEKDQCENYAFCGANSICIYDDNYLTC
ECLRGYVPKSPDEWNIRIWFDGCIRRNKSDCKISYTDGFLKYSHLKLPDTSSSWFSNTMN
LDECQKSCLENCSCKAYANLDIRNGGSGCLLWFNT

>Glyma.06G257500|Glyma.06G257500.1|44050091|GNA/S-locus glycoprotein/PAN/protein kinase/SRK
MLLIWFLLISYTTTTSTSTLRSVNHLAVSQSIRDGETLVSAGGITELGFFSPGNSTRRYL
AIWYTNVSPYTVVWVANRNTPLQNNSGVLKLNEKGILELLSPTNGTIWSSNISSKAVNNP
VAYLLDSGNFVVKNGHETNENSFLWQSFDYPTDTLMSGMKLGWNIETGLERYLTSWKSVE
DPAEGEYTSKIELTGYPQLVRFKGPDIRTRIGSWNGLYLVGYPGPIHETSQKFVINEKEV
YYEYDVVARWAFSVYKLTPSGTGQSLYWSSERTTRKIASTGEEDQCENYAFCGANSICNF
DGNRPTCECLRGYVPKSPDQWNMSVWSDGCVPRNKSNCKNSYTDGFFTYKHLKLPDTSAS
RYNKTMNLDECQRSCLTTCSCTAYTNLDIRDGGSGCLLWSNDLVDMRKFSDWGQDLFVRV

PASELDHAGHGNIKKKIVEIIVGVIIFGFLICASVFIIRNPWTARKLYNKHFKSKPRKED
GDLPTFNLSVLANATENFSTKNKLGEGGFGPVYKGKLIDGQVLAVKRLSKESGQGLEEFK
NEVALIAKLQHRNLVKLLGCCIEGEEKMLIYEYMPNQSLDYFIFDETKRKLLDWHKRFNI
ISGIARGLLYLHQDSRLRIIHRDLKTSNILLDANFDPKISDFGLARSFLGDQFDAKTNRV
AGTYGYIPPEYAARGHFSVKSDVFSYGVILLEIVSGKKNREFSDPQHYNNLLGHAWRLWT
EGRALELLDEVLGEQCTLSEIIRCIQIGLLCVQQRPEDRPDMSSVGLFLNGDKLLSKPKV
PGFYTEKDVTSEANSSSANHKLCSVNELSITILDAR*

>Glyma.06G257700|Glyma.06G257700.1|44160331|GNA/S-locus glycoprotein/PAN/protein kinase
MLSLLCICLLLFSYFSGNCTSLDSLAVNQSIQDGGNETLVSAGGITEVGFFSPAKTTRRY
LGIWFRNVTPLIVVWVANRNTPLENNSGVLKLNQKGILVLLNDKSSTIWSSKISSKAGNN
PIAHPLDSGNFVVKIGQQPNKGTVLWQSFDYPGDTHIPGMKIGWNIETGLERSISSWKSD
EDPAKGEYVVKVDLRGYPQVFVFKGSLIKFRVGPWNGHFWRAQTNTRQVLTIEDQDQCEN
YAFCGENSICSYDGNRPTCECLRGYFPKSPDQWNMSISPNGCVPRNKSNCQNSYTDGFFK
YAHTKMPDTSSSWFNTTMNLDECRKSCLKNCSCTAYANLDIRGGGSGCLLWFNNTVDMRY
FPKFGQDIYIRVPASELDNGGPGIKKKIVVITAGVTVFGLIITCFCILIVKNPEYFILRR
EDMDLPTFELSAIAKATDKFSSRNKLGEGGFGPVYKGTLIDGQEVAVKRHSEMSDQGLEE
FKNEVVLIAKLQHRNLVKLLGCCIHGEEKLLIYECMANKSLDCFIFDETRSKLLSWNQHL
KTSNILLDAHMNPKISDFGMARTFGWDQSQAKTRKVVGTYGYMPPEYAVHGYYSVKSDVF
GFGVIVLEIVSGNKNRGFSDPEHSLNLLGHAWRLWTEDRPLELIDINLSERCIPFEVLRC
IHVGLLCVQQKPQDRPDMSSVIPMLNGEKLLPLPKAPGFYTGNCTPELVSSSKTCNPLSQ
NEISLTIFEAR*

>Glyma.06G257900|Glyma.06G257900.1|44201533|GNA/S-locus glycoprotein/PAN
MSSTLDMVTPTHPIRDGFFNLANSNNRYLGVWYKNIFPRTTVWVANKETPLKDNTGILEV
GTNQGILSIKDGGGAKIWSSSASHTPNKSIVVKLLESGNMVMKDGHNNLLWQSFDYPSDT
LLPGMKIGVNFKTGQHRALRSWKSLSDLTLVIIKENANSSNDIAYRQGSWNGLSVTELPG
EINDQLTKSLFVMNENDVFYEILLLNSSTILRRNLLPEKGYQVRFIWLNKNKRWVGQLPK
PYDVCQTYSLCGANTICNFNGKDKHCECLNCNKGDIDKFQKYDGMKLSDTSSSWYDKTIS
LQECEKYTLSNCSCTAYAQLNISGNGSGCLHWFYDIVDIRTLPMGGQDFYLRMAIVSNLD
LQLQDKSKKDDIDLPIFHFLTISNATNHFSKSNNLGQGGFGPMYKQDEKLLVYEFMPNRS
LCESCRQTLANLLRVHPGTSICIIHSKEEKSFCLSQKVSCNQETICLLNYSTRRTLLDWA
KRFEIICGIA*

>Glyma.06G258100|Glyma.06G258100.1|44232261|GNA/S-locus glycoprotein/PAN
MLGTCTSLDSLAVSQSIQDGETLVSTGGITELGFFSPGNSTRRYLGIWFRNASPLTIVWV
ANRNIPLKNNSGVLKLSEKGILQLLSATNSTIWSSNILSKAANNPIAYLLDSGNFVVKYG
QGTNEDAILWQSFDYPCDTLMAGMKLGWNLKTGLERSLSSWRGVDDPAEGEYTIKIDLRG
YPQIIKFKGPDTISRYGSWNGLTTVGNPDQTRSQNFVLNEKEVFYEFDLPDISTFGVLKL
TPSGMPQTMFWTTQRSTLQVVLLNADDQCENYAFCGANSVCTYDGYLLPTCECLRGYIPK
NPDQWNIAIWSDGCVPRNKSDCENSYTDGFLKYTRMKLPDTSSSWFSKIMNLHECQNSCL
KNCSCSAYANLDIRDGGSGCLLWFNTLVDLRKFTESGQDLYIRLPASELEKRVEYVSMRS
FKDDGGQKKINKKIVAIAVGVTIFGLIITCVCILVIKNPGSLRKFYNKRYKNIPRKEDID
LPTFSFSVLANATENFSTKNKLGEGGYGPVYKGKLLDGKELAVKRLSKKSGQG*

>Glyma.06G258200|Glyma.06G258200.1|44277958|GNA/S-locus glycoprotein/PAN/protein kinase/SRK
MALFLAMLVFSNPLVFFSQISYATDTITQSQPLLDGSTLVSKEGTFELGFFTPGNSPNHY
VGIWFKNIPMRTVVWVANRDNPAKDKSNMLSLSKDGNLILLGKNRSLIWSTNATIAVSNP
VVQLLDNGNLVIREEKDDNMDNEENFVWQSFDYPCDTQLQGMKLGWNLKTGLNRYLTAWK
NWEDPSSGDFTSGLKLGTNPELVISKGSNEYYRSGPWNGIFSSGVFGFSPNPLFEYKYVQ
NEDEVYVVRYTLKNSSVISIIVLNQTLFLRQRITWIPHTRTWSVYQSLPQDSCDVYNVCGA
YGNCMINASPVCQCLEGFKPKSPQDWNQMDWTKGCVRSEPWSCGVKNKDGFRLIAGMKMP
DTTHSWINRSMTLEDCKAKCLKNCSCTAFANMDTGGGGSGCSIWFGDLVDLRISESGQDL
YVRMAISGTGKDNVNADAKHKHLKKVVLVVAITVSLVLLMLLAFSYIYMTKTKYKENGTW
TEEKDDGGQENLELPFFDLATIINATNNFSIDNKLGEGGFGPVYKGTMLDGHEIAVKRLS
KSSGQGLKEFKNEVILCAKLQHRNLVKVLGCCVEGEEKMLLYEYMPNRSLDSFIFDPAQS
KLLDWPTRFNILCAIARGLLYLHQDSRLRIIHRDLKASNILLDNNMNPKISDFGLAKMCG
GDQVEGNTNRIVGTYGYMAPEYAIDGLFSIKSDVFSFGVLLLEIISGKKNRTVTYEEHSD
NLIGHAWRLWKEGIPEQLIDASLVDSCNISELVRCIQVGLLCLQHHPEDRPNMTTVVVML
SSENSLSQPKVPGFLIKNISIEGEQPCGRQESCSTNEVTVSLLNAR*

>Glyma.06G258400|Glyma.06G258400.1|44338609|GNA/S-locus glycoprotein/PAN/protein kinase
MHIYFNSCALTYKKMAIPLSLMLVIAMLFLFSSKISSESDTLTQLQPLHDGATLVSKEGT
FELGFFSPGSSTNRYLGIWFKNIPLKTVIWVANRNYPIINKNTSTYTNTNTKLTITKDGN
LTLLTANNTHHWSTNATTKSVNAVAQLLDSGNLILREEKDNTNSQNYLWQSFDYPSDTLL
PGMKLGWEVTTEALNLNRYLTAWNNWEDPSSGQFAYGVARSSIPEMQLWNGSSVFYRSGP
WNGFRFSATPIPKHRSLVNLNFVDTTKESYYQIFPRNRSLLIRTVVNQTVSTLQRFFWDE

ESQNWKLELVIPRDDFCSYNHCGSFGYCAVKDNSSVCECLPGFEPKSPWTQGCVHSRKTW
MCKEKNNDGFIKISNMKVPDTKTSCMNRSMTIEECKAKCWENCSCTAYANSDITESGSSY
SGCIIWFGDLLDLRQIPDAGQDLYVRIDIFKVDKYGSKKVMVVVASIVSSIIAMLVVLKF
VYWRNKTKFRSEVIIKTKGKTNESEDEDLELPLFDFDFDTIVCATSDFSSDNMLGQGGFG
PVYRGTLPDGQDIAVKRLSDTSVQGLNEFKNEVILCSKLQHRNLVKVLGYCIEEQEKLLI
YEYMSNKSLNFFLFDTSQSKLLDWPRRLDIIGSIARGLLYLHQDSRLRIIHRDLKSSNIL
LDDDMNPKISDFGLARMCRGDQIEGTTRRVVGTYGYMSPEYAIGGVFSIKSDVFSFGVIL
LEVLSGKRNKEFSYSSQNYNLIGHAWRCWKECIPMEFIDACLGDSYIQSEALRCIHIGLL
CVQHQPTDRPDTTSVVTMLSSESVLPQPKKPVFLMERVLVEEDFRQNMNSPTNEVTISEL
EPR*

>Glyma.06G258500|Glyma.06G258500.1|44347886|GNA/S-locus glycoprotein/PAN/protein kinase
MAIQLPLMLLIAILLLLFSSKISSETDTLTQFQPLSDGTTLVSKEGTFELGFFSPGSSTN
RYLGIWFKNIPVKTIVWVANRDNPIKSNTNNTNTKLTITKDGNLVLLTVNDTVHWTTNAT
EKSFNAVAQLLDTGNLVLIDEKDNNSQNYLWQSFDYPTDTLLPGMKIGWEVATGLNRYLT
SWNNWEDPSSGHFAYGVARSNIPEMQIWNGSSVFYRSGPWSGFRFSATPTLKRRSLVNIN
FVDTTEESYYQLFPRNRSLVIRTVVNQTVFALQRFIWDEVTQNWKLDLLIPRDDFCGYNQ
CGSFGFCTEKDNSSVCGCLRGFEPKSPQNRGAKNSTHQGCVQSSKSWMCREKNIDGFVKM
SNMKVADTNTSWMNRSMTIEECKEKCWENCSCTAYANSDITESGSGFSGCILWFSDLLDL
RQFPDGGQDLYVRVDISQIDKDAKDGSKIAVVVVASIVPSIIAILVFTFFYRRSKTKFRS
KVIIKTKGKINESEEEDLELPLFDFETIAFATSDFSSDNMLGQGGFGPVYKGTLPDGHNI
AVKRLSDTSAQGLDEFKNEVIFCSKLQHRNLVKVLGYCIEEQEKLLIYEYMHNKSLNFFL
FDTSQSKLLDWSKRLNIISGIARGLLYLHQDSRLRIIHRDLKSSNILLDDDMNPKISDFG
IARVCRGDIIEGNTSRVVGTYGYMAPEYAIGGLFSIKSDVYSFGVILLEVLSGKKNKGFS
FSSQNYNLIAHAWWCWKECSPMEFIDTCLRDSYIQSEALRYIHIGLLCVQHQPNDRPNMT
AVVTMLTSESALPHPKKPIFFLERVLVEEDFGQNMYNQTNEVTMSEMQPR*

>Glyma.06G258900|Glyma.06G258900.1|44382191|GNA/S-locus glycoprotein/PAN/protein kinase
MTIFTLILAIANLLLFLSKASSIDTLTLSQSLPDGTTLVSKDETFELGFFSLRNSTNRYL
GIWFKNIPVKTVVWVANRDYPLKDNSTKLIITNDGNLVLLTKNNKVQWSTNTTTKASRPI
LQLLNTGNLVLRNDNEDNKNNNKSSNNNNEDRFLWQSFDYPSDTLLPGMKLGWYRKTGLN
RRVIAWKNWDDPSPGNFSWGITFDSNPEMVLWKGSFKYHRSGPWNGIRFSGAFGGSNRLS
THPLFVYKLINNDDEVYYSYSLTNKSVISIVVMNQTLLRRQRNIWIPENGTWRLFQTAPR
DICDTYNPCGSYANCMVDSSPVCQCLEGFKPKSLDTMEQGCVRSEPWSCKVEGRDGFRKF
VGLKFPDTTHSWINKSMTLEECKVKCWENCSCTAYANLDIRGAGSGCSIWFGDLIDLKVV
SQSGQYLYIRMADSQTDAKDAHKKKELLLIGTIVPPIVLVILLAIFYSYKRKRKYEEENV
SVVKKDEAGGQEHSMELPLFDLATLVNATNNFSTDNKLGQGGFGPVYKGVLAGGQEIAVK
RLSRSSGQGLTEFKNEVILCAKLQHRNLVKVLGCCIEEEEKMLLYEYMPNKSLDSFLFDS
TKSKILDWSKRFHILCATARGLLYLHQDSRLRIIHRDLKASNILLDNNLNPKISDFGLAR
MCGGDQIEGNTNRVVGTYGYMAPEYVIHGLFSTKSDVFSFGILLLEIISGKKNREITYPY
HSHNLIGHAWKLWKEGIPGELIDNCLQDSCIISEALRCIHIGLLCLQRQPNDRPNMASVV
VMLSSDNELTQPKEPGFLIDRVLIEEESQFRSQTSSSTNGVTISILDAR*

>Glyma.06G260900|Glyma.06G260900.1|44662225|GNA/S-locus glycoprotein/PAN/protein kinase
MSNNRELLYSMKILSFMMLVICIVVPSLRICVANDSVNVLQSMSDGERLVSKGGNFELGF
FSPGSSQKRYVGIWYKNIPTQTVVWVANGANPINDSSGILTLNTTGNLVLTQNGSIVWYT
NNSHKQVQNPVVELLDSGNLVIRNDGEPNPEAYLWQSFDYPSHALLPGMKFGRDLRTGLE
RRYTAWKSPEDPSPGDVYGVLKPYNYPEFYMMKGEKKLLRQGPWNGLYFSGFPDLQNNTI
FGINFVSNKDEIYYTFSLVKSSVVTINVINQTGRTYRYVWVEGDQNWRIYISQPKDFCDT
YGLCGAYGSCMISQTQVCQCLKGFSPKSPQAWASSDWTQGCVRNNPLSCHGEDKDGFVKF
EGFKVPDSTHTWVDESIGLEECRVKCLSNCSCMAYTNSDIRGEGSGCVMWFGDLIDMKQL
QTGGQDLYIRMPASELEHKKNTKTIVASTVAAIGGVLLLLSTYFICRIRRNNAEKDKTEK
DGVNLTTFDFSSISYATNHFSENNKLGQGGFGSVYKGILLDGQEIAVKRLSETSRQGLNE
FQNEVKLIAKLQHRNLVKLLGCSIQKDEKLLIYELMPNRSLDHFIFDSTRRTLLDWVKRF
EIIDGIARGLLYLHQDSRLKIIHRDLKTSNVLLDSNMNPKISDFGMARTFGLDQDEANTN
RIMGTYGYMPPEYAVHGFFSVKSDVFSFGVIVLEIISGRKIRGFCDPYHNLNLLGHAWRL
WTEKRSMEFIDDLLDNSARLSEIIRYIHIGLLCVQQRPEDRPNMSSVILMLNGEKLLPEP
SQPGFYTGKVHSTMTESSPRNTDAYSFNEISNSLLEAR*

>Glyma.06G261000|Glyma.06G261000.1|44680166|GNA/S-locus glycoprotein/PAN/protein kinase
MEILSFMIIFACIFVPSLKISLAIDSINLLQSVRDGETLVSKGGKFELGFFSPGSSQKRY
LGIWYKNIPNKTVVWVANGANPINDSSGIITLNNTGNLVLTQKTSLVWYTNNSHKQAQNP
VLALLDSGNLVIKNEEETDPEAYLWQSFDYPSDTLLPGMKLGWDLRTGLDRRYTSWKSPD
DPSPGDVYRALVLHNYPELYMMKGTQKLYRYGPWNGLYFSGQPDLSNNTLFNLHFVSNKD
EIYYTYTLLNDSDITRTITNQTGQIDRYVWDENGQTWRLYRYYPKEFCDSYGLCGPNGNC
VITQTQACQCLKGFSPKSPQAWFSSSDWTGGCVRNKGLSCNGTDKDKFFKFKSLKVPDTT

YTFVDESIGLEECRVKCLNNCSCMAFTNSDINGEGSGCVMWFHDLFDMRQFESVGQDLYI
RMAASESDSQEPVSRHKNNTPKIVASSIAAICGVLFLSTYFICRIRRNRSPRNSAANLLP
EDNSKNDLDDLEVQLFDLLTIATATNDFSTENKIGEGGFGPVYKGILMDGREIAVKTLSK
STWQGVAEFINEVNLIAKLQHRNLVKFLGCCIQRQERMLIYEYMPNGSLDSLIFDDKRSK
LLEWPQRFNIICGIARGLMYIHQDSRLRIIHRDLKPSNILLDENLSPKISDFGVARTFGG
DESEGMTRRVVGTYGYMAPEYAVDGSFSVKSDVFSFGILALEIVSGTRNKGLYQTDKSHN
LVGHAWTLWKAGRELDLIDSNMKLSSCVISEVQRCIHVSLLCVQQFPDDRPPMKSVIPML
EGHMEMVEPKEHGFISVNVLGELDLHSNPQNTSSSNYVTITMLEGR*

>Glyma.06G261100|Glyma.06G261100.1|44721147|GNA/S-locus glycoprotein/PAN/protein kinase
MHIIIIQISEYHKSSDPMHILSFIILFTCILVPFPKISVANDSINLRQSMRDGDTLVSKT
RKFELGFFSPGSSQKRYLGIWYKNIPIQTVVWVANRENPINDSSGILTLNNTGNFVLAQN
ESLVWYTNNSHKQAQNPVAVLLDSGNLVIRNDGETNPEAYLWQSFDYPSDTLLPGMKLGW
DLRTGLDRRLTAWKSPDDPSPGDVYRDLELYSYPEFYIMKGTKKVYRFGPWNGLYFSGVP
DLRNNTIFGFNFFSNKEESYYIFSPTNDVMSRIVMNESTTIYRYVWVEDDQNWRIYTSLP
KDFCDTYGLCGVYGNCMTTQTQVCQCLKGFSPKSPEAWVSSGWSQGCVRNKPLSCKDKLT
DGFVKYEGLKVPDTRHTWLDESIGLEECKVKCLNNCSCMAYTNSDIRGAGSGCVMWFGDL
IDIKQLQTAGQDLYIRMPASELESVYRHKKKTTTIAASTTAAICGVLLLSSYFICRIRRN
NAGKSLTEYDSEKDMDDLDIQLFDLPTITTATNDFSMENKIGEGGFGPVYKGILVDGQEI
AVKTLSRSSWQGVTEFINEVKLIAKLQHRNLVKLLGCCIQGQEKMLIYEYMANGSLDSFI
FDDKKRKLLKWPQQFHIICGIARGLMYLHQDSRLRIIHRDLKASNVLLDENSSPKISDFG
MARTFGGDQFEGNTSRVVGTCGYMAPEYAVDGSFSVKSDVFSFGILVLEIVCGKRNKGLY
QTDKSLNLVGHAWTLWKEGRALDLIDDSNMKESCVISEVLRCIHVGLLCVQQYPEDRPTM
ASVILMLESHMELVEPKEHGFISRNFLGEGDLRSNRKDTSSSNDVTITLLEAR*

>Glyma.06G261200|Glyma.06G261200.1|44748559|GNA/S-locus glycoprotein/PAN/protein kinase
MTDGESLVSKGGKFELGFFSPGNSQKRYLGIWYKNVPNQTVVWVANREDPINDSSGILTL
NTTGNLVLTQNKSLVWYTNNSHKQAPNPVAVLLDSGNLVIRNEGETNPEAYLWQSFDYPS
DTFLPGMKLGWNLRTGHEWKLTAWKSPDDPSPGDVYRVFKLYNYPELYVMKKTKKLYRFG
PWNGLYFSGMSDLQNNTVHSFYYVSNKDEIYYAYSLANDSVIVRSVTDQTTSTVYRYKWV
VGEQNWRLSRSFPTEFCDTYSVCGAYGNCVSSTQPQACNCLKGFSPNSPQAWKSSYWSGG
CVRNKPLICEEKLSDGFVKFKGLKVPDTTHTWLNESIGLEECRVKCLSNCSCMAFANSDI
RGEGSGCVMWFGDLIDMKQLQTDGQDLYIRMHASELATNCWKDKSEKDDNIDLQAFDFPS
ISNATNQFSESNKLGQGGFGPVYKGMLPNGQEIAVKRLSNICGQGLDEFKNEVMLIAKLQ
HRNLVTLVGCSIQQDEKLLIYEFMPNRSLDYFIFDSARRALLGWAKRLEIIGGIARGLLY
LHQDSKLKIIHRDLKTSNVLLDSNMNPKISDFGMARTFELDQDEENTTRIMGTYGYMSPE
YAVHGSFSVKSDVYSFGVIILEIISGRKIKEFIDPHHDLNLLGHAWRLWIQQRPMQLMDD
LADNSAGLSEILRHIHIGLLCVQQRPEDRPNMSSVVLMLNGEKLLPQPSQPGFYTGNNHP
PMRESSPRNLEAFSFSEMSNSVLVAR*

>Glyma.06G261400|Glyma.06G261400.1|44785849|GNA/S-locus glycoprotein/PAN/TIR/NB ARC/LRR/LRR/LRR
MSDGETLVSKGGKFEFGFFSPGNSHKRNLVLTKNESLVWYTNNSHNQAQNPVAELLDSGN
LVIRNDGETNPEAYLWQSFDYPSDTFLPGMKLGWNLRIGHEWKQTAWKSPDDPSPGDVYR
VLELYNYPEFYVMKGTKKAYRFGPWNGLYFSGLSDFENGTMYSFCYVSNKHEISFTYSIA
NDSFIARSVANQTAITIYRYMWVVGEQDWKMSRSFPQEFCDTYSLCGAYGNCVSSTQRQA
CQCLKGFSPKSPEAWNSSDWSGGCLKVPDTTHTWWDESIGLEECRVKCLNSCSCMAYSNS
DIRGEGSGCVMWFGDLIDMKQLQTEAGRPMLVLILEVQQQTQHVTSCMVTKSSLKNIIFI
LTNGSPLNEEKLENLAKEEWQLHISVEYFHACIPLRSLCLLKRFDQQLNFKIIYNFNKYF
AIEMASTSNAIIQCTSSSSSSSFEYDVFVSFRGEDTRNSFTGFLFEALKKQGIEAFKDDK
DIRKGESIAPELIRAIEGSHVFLVVFSKDYASSTWCLRELAHIWDCIQKSPRHLLPIFYD
VDPSQVRKQSGDYEKAFAQHQQSSRFEDKEIKTWREVLNDVGNLSGWDIKNKQQHAVIEE
IVQQIKNILGCKFSTLPYDNLVGMESHFATLSKLICLGLVNDDVRVVGITGMGGIGKSTL
GQALYERISHQFNSRCYIDDVSKLYQGYGTLGVQKELLSQSLNEKNLKICNVSNGTLLVW
ERLSNAKALIIILDNVDQDKQLDMFTGGRNDLLRKCLGKGSIVIIISRDQQILKAHGVDVI
YRVEPLNDNDALGLFCKKAFKNNYMMSDFEKLTSDVLSHCQGHPLAIEVLGSSLFDKDVL
HWRSALALLRENKSKSIMNVLRISFDQLEDTHKEIFLDIACFFNHYPVKYVKEVLDFRGF
NPEYGLQVLVDKSLITMDSRQIQMHDLLCDLGKYIVREKSPRKPWKWSRLWDVKDILKVM
SDNKAADNVEAIFLIEKSDILRTISTMRVDVLSTMSCLKLLKLDHLDFNVKINFFSGTLV
KLSNELGYLGWEKYPFECLPPSFEPDKLVELILPKSNIKQLWEGTKPLPNLRRLDLSGSK
NLIKMPYIGDALYLESLDLEGCIQLEEIGLSIVLSPKLTSLNLRNCKSLIKLPQFGEDLI
LEKLLLGGCQKLRHIDPSIGLLKKLRRLNLKNCKNLVSLPNSILGLNSLEDLNLSGCSKL
YNTELLYELRDAEQLKKIDIDGAPIHFQSTSSYSREHKKSVSCLMPSSPIFPCMLKLDLS
FCNLVEIPDAIGIMCCLQRLDLSGNNFATLPNLKKLSKLVCLKLQHCKQLKSLPELPSRI
YNFDRLRQAGLYIFNCPELVDRERCTDMAFSWTMQSCQVLYLCPFYHVSRVVSPGSEIPR
WFNNEHEGNCVSLDASPVMHDHNWIGVAFCAIFVVPHETLSAMSFSETEGNYPDYNDIPV
DFYEDVDLELVLDKSDHMWLFFVGRGRFIEYFHLKHKYLGRLLLKCDNEGIRFKESYAEV
KKYGYRWVYKGDIEWSARKHWGN*

>Glyma.06G261800|Glyma.06G261800.1|44863206|GNA/S-locus glycoprotein/PAN/protein kinase
MLNPYYRYPKNLVLGVGKKIMMNRTNISLPNPIYHFMIIVAYMLVPSLKISAAILSVSQF
ITESQTLVSHRGVFELGFFSPGNSKNRYLGIWYKTITIDRVVWVANWANPINDSAGILTF
SSTGNLELRQHDSVAWSTTYRKQAQNPVAELLDNGNLVVRNEGDTDPEAYLWQSFDYPSD
TLLPGMKLGWDLRTALEWKITAWKSPEDPSPGDFSFRLNLYNYPEFYLMKGRVKYHRLGP
WNGLYFSGATNQNPNQLYEIKYVVKNDSMYVMNEVEKFCFLTVKNSSAAAIVRVKITETS
LQIQVWEEERQYWSIYTTIPGDRCDEYAVCGAYGNCRISQSPVCQCLEGFTPRSQQEWST
MDWSQGCVVNKSSSCEGDRFVKHPGLKVPETDHVDLYENIDLEECREKCLNNCYCVAYTN
SDIRGGGKGCVHWYFELNDIRQFETGGQDLYIRMPALESVNQEEQHGHTTSVKIKIATPI
AAISGILLFCIFVMYRVRRSSADKSKTKDNLKKQLEDLDLRLFDLLTITTATNNFSLNNK
IGQGGFGPVYKGKLADGRDVAVKRLSSSSGQGITEFMTEVKLIAKLQHRNLVKLLGCCIR
GQEKILVYEYMVNGSLDSFVFDQIKGKFLDWPQRLDIIFGIARGLLYLHQDSRLRIIHRD
LKASNILLDEKLNPKISDFGMARAFGGDQTEGNTNRVVGTYGYMAPEYAVDGLFSIKSDV
FSFGILLLEIICGNKNRALCHGNQTLNLVGYAWTLWKEQNVLQLIDSNIMDSCVIQEVLR
CIHVSLLCVQQYPEDRPTMTSVIQMLGSEMELVEPKEPGFFPRRISNEGKLLANLNQMTS
NNELTITLLNAR*

>Glyma.06G261900|Glyma.06G261900.1|44875369|GNA/S-locus glycoprotein/PAN/protein kinase
MKFILSLKSFIYILFFPSLVVSIVPDRSSISQFQSLSYGKTIVSSPHGMFELGFFNLGYP
NRIYLGIRYKNIPVDNVVWVANGGNPINDSSADLKLHSSGNLVLTHNNMVAWCTRSSKAA
QNPVAELLDSGNLVIRDLNSANQESYLWQSFDYPSNTMLSGMKVGWDLKRNLNIRLIAWK
SGDDPTPGDLSWSIVRHPYPEIYMMKGNKKYHRLGPWNGLRFTGMPEMKPNPVYHYEFVS
NKEEVYYTWTLKQTSLITKAVLNQTALARPRYVWSELDESWMFYSTLPSDYCDHYGVCGA
NAYCSTSASPMCECLKGFKPKYLEKWNSMDWSQGCVLQHPLNCKHDGFVLLEGLKVPDTK
ATFVNDSIDIEKCRTKCLNNCSCMAYTNSNISGAGSGCVMWFGDLFDIKQYSVAENGQGL
YIRLPASELEAIRQRNFKIKHVTIVTAASGMLVLGIYFIYRIWRNINEKSKAENNYEGFV
DDLDLPLLDLSIILAATDNFSEVNKIGEGGFGPVYWGKLASGLEIAAKRLSQNSGQGISE
FVNEVKLIAKLQHRNLVKLLGCCIHKQEKILVYEYMANGSLDYFIFDLLRNTDHTKGKSL
DWPKRLSIICGIARGLMYLHQDSRLRIIHRDLKGSNVLLDEDFNPKISDFGMAKTVGREE
IEGNTNKIVGTFGYMAPEYAVDGQFSVKSDVFSFGILLMEIICGKRNRGRYSGKRYNLID
HVWTHWKLSRTSEIIDSNIEDSCIESEIIRCIHVGLLCVQQYPEDRPTMTSVVLMLGSEM
ELDEPKKPGVFTKKESIEAISSSSTNTLTITLSAR*

>Glyma.06G262000|Glyma.06G262000.1|44888315|GNA/S-locus glycoprotein/PAN/protein kinase
MRFVHFLMNIIIYTLFDTFLLVFEAAGTSSFIAQYQSLSYGKSIVSSPRGTYELCFFNLG
NPNKIYLGIRYKNIPTQNVVWVANGGNPINDSSTILELNSSGNLVLTHNNMVVWSTSYRK
AAQNPVAELLDSGNLVIREKNEAKPEEEEYLWQSFDYPSNTMLAGMKVGWDLKRNFSIRL
VAWKSFDDPTPGDLSWGVTLHPYPEFYMMKGTKKYHRLGPWNGLRFSGRPEMAGSDPIYH
FDFVSNKEEVYYTWTLKQTNLLSKLVLNQTTQERPRYVWSETEKSWMFYTTMPEDYCDHY
GVCGANSYCSTSAYPMCECLKGFKPKSPEKWNSMGWTEGCVLKHPLSCMNDGFFLVEGLK
VPDTKHTFVDESIDLEQCKTKCLNDCSCMAYTNSNISGAGSGCVMWFGDLIDIKLYPVPE
KGQDLYIRLPSSELESSRDKKDSKIIIIATSIGATLGVILAIYFVYRRNIAEMSNAENNH
EEPLPQHGHNRWNIADKSKTKENIKRQLKDLDVPLFDLLTITTATNNFSSNNKIGQGGFG
PVYKGKLVDGRDIAVKRLSSGSGQGIVEFITEVKLIAKLQHRNLVKLLGCSFPKQEKLLL
YEYMVNGSLDSFIFDQQKGKLLDWPQRFHIIFGIARGLLYLHEDSRLRIIHRDLKASNVL
LDEKLNPKISDFGMARAFGGDQTEGNTNRVVGTYGYMAPEYAVDGVFSIKSDVFSFGILL
LEIICGNKNRSLCHGNQTLNLVGYAWTLWKEQNTSQLIDSNIKDSCVIPEVLRCIHVSLL
CVQQYPEDRPTMTSVIQMLGSEMELVEPKEPGFFPRRISDERNLSSNLNQTISNDEITIT
TLKGR*

>Glyma.06G262100|Glyma.06G262100.1|44913295|GNA/S-locus glycoprotein/PAN/protein kinase
MKFILTLTSFILYILFVSSLVVSIAADTSSISQSQSLSFGRTIVSPNGVFELGFFNLGNP
NKSYLGIWFKNIPSQNIVWVANGGNPINDSFAILSLNSSGHLVLTHNNTVVWSTSSLRET
QNPVAKLLDSGNLVIRDENEVIQEAYLWQSFDYPSNTGLSGMKIGWYLKRNLSIHLTAWK
SDDDPTPGDFTWGIVLHPYPEIYLMKGTKKYYRVGPWNGLSFGNGSPELNNSIYYHEFVS
DEEEVSYTWNLKNASFLSKVVVNQTTEERPRYVWSETESWMLYSTRPEDYCDHYGVCGAN
AYCSTTASPICECLKGYTPKSPEKWKSMDRTQGCVLKHPLSCKYDGFAQVDDLKVPDTKR
THVDQTLDIEQCRTKCLNDCSCMAYTNSNISGAGSGCVMWFGDLLDIKLYSVAESGRRLH
IRLPPSELESIKSKKSSKIIIGTSVAAPLGVVLAICFIYRRNIADKSKTKKSIDRQLQDV
DVPLFDMLTITAATDNFLLNNKIGEGGFGPVYKGKLVGGQEIAVKRLSSLSGQGITEFIT
EVKLIAKLQHRNLVKLLGCCIKGQEKLLVYEYVVNGSLNSFIFDQIKSKLLDWPRRFNII
LGIARGLLYLHQDSRLRIIHRDLKASNVLLDEKLNPKISDFGMARAFGGDQTEGNTNRVV
GTYGYMAPEYAFDGNFSIKSDVFSFGILLLEIVCGIKNKSFCHENLTLNLVGYAWALWKE
QNALQLIDSGIKDSCVIPEVLRCIHVSLLCVQQYPEDRPTMTSVIQMLGSEMDMVEPKEP
GFFPRRILKEGNLKEMTSNDELTISLFSGR*

>Glyma.06G262300|Glyma.06G262300.1|44955111|GNA/S-locus glycoprotein/PAN/protein kinase
MKFILTVTSFILYILFVSSLVVSIAADTSSISQSQSLSFGRTIVSPNGVFELGFFNLGNP
NKSYLGIWFKNIPSQNIVWVANGGNPINDSFALLSLNSSGHLVLTHNNTVVWSTSSLRET
QNPVAKLLDSGNLVIRDENEVIQEAYLWQSFDYPSNTGLSGMKIGWYLKRNLSIHLTAWK
SDDDPTPGDFTWGIILHPYPEIYLMKGTKKYYRVGPWNGSPGLINSIYYHEFVSDEEELS
FTWNLKNASFLSKVVVNQTTQERPRYVWSETESWMLYSTRPEDYCDHYGVCGANAYCSST
ASPICECLKGYTPKSPEKWKSMDRTQGCVLKHPLSCKYDGFAQVDGLKVPDTKRTHVDQT
LDIEKCRTKCLNDCSCMAYTNYNISGAGSGCVMWFGDLLDIKLYSVAESGRRLHIRLPPS
ELESIKSKKNSKIIIGTSVAAALGVVLAICFIHRRNIADKSKTKKSNDRQLQDVDVPLFD
LLTITAATDNFLLNNKIGEGGFGPVYKGKLEGGQEIAVKRLSSRSGQGITEFITEVKLIA
KLQHRNLVKLLGCCIKGQEELLVYEYVVNGSLNSFIFDQIKSKLLDWPRRFNIILGIARG
LLYLHQDSRLRIIHRDLKASNVLLDEKLNPKISDFGMARAFGGDQTEGNTNRVVGTYGYM
APEYAFDGNFSIKSDVFSFGILLLEIVCGIKNKSLCHENQTLNLVGYAWALWKEQNALQL
IDSGIKDSCVIPEVLRCIHVSLLCVQQYPEDRPTMTSVIQMLGSEMDMVEPKEPGFFPRR
ILKEGNLKEMTSNDELTISLFSGR*

>Glyma.06G262400|Glyma.06G262400.1|44964196|GNA/S-locus glycoprotein/PAN/protein kinase
MKFILTMMSSIPYILFVSSLVVSIAADTSSISQSQSLSFGKTIVSPSGTFELGFFHLGNP
NKSYLGIWFKNIPSRDIVWVLPINNSSALLSLKSSGHLVLTHNNTVVWSTSSLKEAINPV
ANLLDSGNLVIRDENAANQEAYLWQSFDYPSDTMVSGMKIGWDLKRNLSIHLSAWKSADD
PTPGDFTWGIILHPYPEMYLMKGNKKYQRVGPWNGLQFSGGRPKINNPVYLYKFVSNKEE
IYYEWTLKNASLLSKLVVNQTAQDRSRYVWSETTKSWGFYSTRPEDPCDHYGICGANEYC
SPSVLPMCECLKGYKPESPEKWNSMDRTQGCVLKHPLSCKDDGFAPLDRLKVPDTKRTYV
DESIDLEQCKTKCLKDCSCMAYTNTNISGAGSGCVMWFGELFDIKLFPDRESGQRLYIRL
PPSELESNWHKKISKIVNIITFVAATLGGILAIFFIYRRNVADKSKTKESIERQLEDVDV
PLFNLLTITIATNNFLLKNKIGQGGFGPVYKGKLEGGQEIAVKRLSSRSGQGLTEFITEV
KLIAKLQHRNLVKLLGCCIKGKEKLLVYEYMVNGSLDSFIFDKIKSKLLDWPQRFHIILG
IVRGLLYLHQDSRLRIIHRDLKASNILLDEKLNPKISDFGLARAFGGDQTEGNTDRVVGT
YGYMAPEYAVDGQFSIKSDVFSFGILLLEIVCGNKNKALCHENQTLNLVGHAWTLWKEQN
ALQLIDSSIKDSCVISEVLRCIHVSLLCVQQYPEDRPTMTSVIQMLGSEMDMVEPKEPGF
FPRRILKEGNLCTNLNQVTSNDELSITSLSGR*
>Glyma.06G262600|Glyma.06G262600.1|44984109|GNA/PAN/protein kinase
MSFILYTLFVSSVVFSIAADNTSSISQSQSLSFGKTMVSPRGIFELGFFNLGLPNKSYLG
IWFKNNPSQNVVWVANGGNPINDSSAILRLNSSGNLVLTHNNTVVWSTNCPKEAHNPVAE
LLDFGNLVIRDENAANQEAYLWQSFDYPSDTMLPGDFTWGIILHPYPEIYIMKGTKKYHR
VGPWNGLCFSGGRPKTNNPIYHYEFVSNKEEILENHGCFIQQGPKTTVTIMGFVEAMRIA
ALLHHQCECLKGFKPKSPEKLNSMDWFQGCVLKHPLSCKYDGFAPVDGLKVPDTKRTYVD
ETIDLEQCRRRCLKDCSCMAYTNTNISETGTGSACVIWFGDLFDLTSYYFQFRKRAASIY
KVAWSEVVTGVVVVLGVVVMVKKNMDKSKTKESIERQLKDVDVPLFDLLTIATATNNFLL
NNKIGQGGFGPVYKGKLVGGQEIAVKGLSSRSGQGITEFITEVKPIAKLQHRNLVKLLGC
CIKGHEKLLVYEYMVNGSLDFFIFDFGSNILLDEKLNKKISDFGMTRAFGGDQTQGNTNR
VVGT*

>Glyma.06G262700|Glyma.06G262700.1|44993233|GNA/S-locus glycoprotein/PAN/protein kinase
MKFILSLMSIILYTLFISSLVVSIAADKSSNSQFQSLSHEETIVSPNGVFELGFFPLGNS
NKSYLAIRYKNYSDETFVWVANGSYPINDSSAKLTLHSSGSFVLTHNSNQVWSTSSLKVA
QNPLAELLDSGNLVIREKSEANSEDKEEYLWQSFDYPSNTMLAGMKIGWDHKRKLNRRLI
AWKSDDDPTPGELSWEVVLHPYPEIYMMRGKEKHHRLGPWNGLRFSGMPEMKPNPVFHYK
FVSNEEEVTYMWTLQTSLITKVVLNQTSLERPRFVWSEATASWNFYSTMPGEYCDYYGVC
GGNSFCSSTASPMCECLKGFTPKSPEKWNSMVRTQGCGLKSPLTCKSDGFAQVDGLKVPD
TTNTSVYESIDLEKCRTKCLKDCSCMAYTNSNISGAGSGCVMWFGDLLDIKLYPDPESGQ
RLYIRLPPSELDSIRPQVSKIMYVISVAATIGVILAIYFLYRRKIYEKSMTEKNYESYVN
DLDLPLLDLSIIIAATNKFSEGNKIGEGGFGSVYWGKLPSGLEIAVKRLSKNSDQGMSEF
VNEVKLIAKVQHRNLVKLLGCCIKKQEIMLVYEYMVNGSLDYFIFDSTKGKLLDWPKRFH
IICGIARGLMYLHQDSRLRIIHRDLKASNVLLDDTLNPKISDFGVAKTFGGENIEGNTTR
IVGTYGYMAPEYAIDGQFSIKSDVFSFGVLLLEIICGKRSRCSSGNQIVHLVDHVWTLWK
KDMALQIVDPNMEDSCIASEVLRCIHIGLLCVQQYPEDRPTMTSVVLLLGSEVELDEAKE
PGDFPKKESIEANSSSFSSTNAMSTTLLTAR*

>Glyma.06G300600|Glyma.06G300600.1|48955701|GNA/S-locus glycoprotein/PAN/protein kinase
MRNNKPQLWLSLSLFITCFSFHTSLAALTTISANQSLSGDETLVSQGGEFELGFFNTGNN
SNKFYIGMWYKKISQRTYVWVANRDQPVSDKNSAKLTILDGDLVLLDQYQNLVWSTNLNS
PSSGSVVAVLLDSGNLVLSNRANASASDAMWQSFDHPTDTWLPGGKIKLDNKTKKPQYLT
SWKNREDPAQGLFSLELDPAGRNAYLILWNKSEQYWTSGAWNGHIFSLVPEMRLNYIYNF
TFQSNENESYFTYSVYNSSIITRFVMDGSGQIKQLSWLDNAQQWNLFWSQPRQQCEVYAF
CGGFGSCTENAMPYCNCLNGYKPKSQSDWNLNDYSGGCVKKTNFQCENPNSSNKDKDRFL

PILNMKLPNHSQSIGAGTSGECEATCLSNCSCTAYAYDNSGCSIWNGDLLNLQQLTQDDS
SGQTLFLRLAASEFHDSKSNKGTVIGAAGAAAGVVVLLIVFVFVMLRRRRRHVGTGTSVE
GSLMAFSYRDLQNATKNFSDKLGGGGFGSVFKGTLADSSIIAVKKLESISQGEKQFRTEV
STIGTVQHVNLVRLRGFCSEGTKKLLVYDYMPNGSLESKMFYEDSSKVLDWKVRYQIALG
TARGLNYLHEKCRDCIIHCDVKPENILLDADFVPKVADFGLAKLVGRDFSRVLTTMRGTR
GYLAPEWISGVAITAKADVYSYGMMLFEFVSGRRNSEASEDGQVRFFPTYAANMVHQGGN
VLSLLDPRLEGNADLEEVTRVIKVASWCVQDDESHRPSMGQVVQILEGFLDLTLPPIPRT
LQAFVDNHENIVFFDDSSSTQSSQVKSNASSASSQAKSNISSSKSST*

>Glyma.07G069000|Glyma.07G069000.1|6232846|GNA/S-locus glycoprotein/protein kinase
MSLFILILILILILHSPTCSTTIILQGNATLQSPNNTFRLGLFSFSPNSSFYLAIRHTSL
PFPNTIWLANRLHPSPSQTASSLQLTQTGQLLLTHSNTTLWTTTISNIHPSNFSSLSLKL
LDSGNLILTAPNGVVLWQSFDSPTDTWLPGMNLTRLNSLLSWRTETDPSPGLYSLRLKPP
FYGEFELVFNDTVPYWSTGNWTNGSFLNIPEMSIPYLYNFHFLSPFSPAAAFGFSERAES
EAGNRPPTMFRVEPFGQIQQYTWNSQAGSWNMFWSKPEPLCLVRGLCGRFGVCIGETSKP
CECISGFQPVDGDGWGSGDYSRGCYRGDSGCDGSDGFRDLGNVRFGFGNVSLIKGKSRSF
CERECLGDCGCVGLSFDEGSGVCKNFYGSLSDFQNLTGGGESGGFYVRVPRGGSGGRKGL
DRKVLAGVVIGVVVVSGVVVVTLLMMVKKKRDGGRKGLLEEDGFVPVLNLKVFSYKELQL
ATRGFSEKVGHGGFGTVFQGELSDASVVAVKRLERPGGGEKEFRAEVSTIGNIQHVNLVR
LRGFCSENSHRLLVYEYMQNGALSVYLRKEGPCLSWDVRFRVAVGTAKGIAYLHEECRCC
IIHCDIKPENILLDGDFTAKVSDFGLAKLIGRDFSRVLATMRGTWGYVAPEWISGVAITT
KADVYSYGMTLLELVGGRRNVEAPPSAGGGGGGRESGSETGTKWFFPPWAAQQIIEGNVS
DVVDKRLGNGYNIDEARRVALVAVWCIQDDEAMRPTMGMVVKMLEGLVEVSVPPPPKLLQ
ALVTGDSFHGVKADSGNGVSTGGSLSDGNLEVSTADSESYTGNVFSPLDVNVHVSVR*

>Glyma.07G080700|Glyma.07G080700.1|7341451|GNA/S-locus glycoprotein/protein kinase
MNANMASSPTLIPFLLIFFLFHFHRSSSLPLSVENPEDDVIVSSPKGTFTAGFSPVGENA
YSFAIWFSTQATTKTVVWMANRDQPVNGKRSTLSLLKTGNLVLTDAGQFDVWSTNTLSSK
TLELHLFDTGNLVLREQSNQSAVLWQSFGFPTDTLLPGQIFTRFTKLVSSRSEGNHSSGF
YNLYFDNDNVFRILYDGPQVSSVYWPDPWLVSDNVGFGNGRSTYNSSRVAVLDNLGEFSA
SDHFSFKTIDYGLLLQRRLTLDHDGNVRVYSRKNGEENWSITGQFKSQPCFIHGICGPNS
ICSHEQVIGRKCSCLEGYSWIDSQDWTLGCKPNFQPTCDNKTEYRFVPYYEVDFYGYDYG
SSFSNYTYKQCEKLCSGLCECMGFQYSFARENGLFWCYPKRQLLNGHHSPGFTGQIFLRL
PKNDVQENRVQNSDDLACSRNAEKVLERPYVKGKENGSVKFMLWFAIGLGGFEVLCIFMV
WCFLFRSSNHLVSADQQGYVLAAATGFRRYTYSELKQATKGFSEEIGRGAGGTVYKGVLS
DKRIAAIKKLHEFADQGESEFLTEVSIIGRLNHMNLIGMWGYCVEGKHRMLVYEYMENGS
LAHNLPSNALDWSKRYNIAVGMAKGLAYLHEECLEWILHCDIKPQNILLDSDYQPKVADF
GLSKPLNRNNVNNSSFSRIRGTRGYMAPEWVFNLQITSKVDVYSYGIVVLEMITGRSPMI
GVQVTELGADQSHNERLATWVRERRRKAREGECWVEQIVDPTLGSDYDVEQMEILTTVAL
ECVEEEKDVRPSMSQVVERLQSHDS*

>Glyma.07G108200|Glyma.07G108200.1|10636080|GNA/S-locus glycoprotein/protein kinase
MASSTFLLPLFVSLIFHNFQHSSSFSLSVEKFKEDVIVSSPKGKFTAGFYPVGDNAYCFA
IWYTQPPHTLVWMANRDQPVNGKRSTLSLLTTGNLVLTDAAQFMVWSTNTATSSKQVQLH
FYDTGNLVLLDNSDNVALLWQSFDFPTDTLLPNQPLRKSTNLISSRSGTNYSSGYYKLFF
DFENVLRLMYQGPQVSSVYWPYDWLRSNNIDYGIGNGRYTFNDSRVVVLDDFGYLVSSDN
FTSKTSDYGMIIQRRLTLDHDGNVRVYSIKDGQDKWSVSGIFRRQPCFIHGICGPSSICS
YEPASGRKCSCLPGYRWLDSEDWSQGCVPKFQLWCRNNNTEQDSRFLQLPEVDFYGYDYG
FFLNHTYQQCVNLCLRLCECKGFQHSSSGQGGVNGQCYLKTQLLNGHRTPGYSRSFILRL
PSSMHDYDENTINIGLVCGGNRGVQVLERPYVEEKENGSVKLMMWFASALGGIEVVCIFM
VWCFLFRKNNADKQIYVLAAETGFRKFSYSELKQATKNFSEEIGRGGGGTVYKGVLSDNR
VAAIKRLHEVANQGESEFLAETSIIGRLNHMNLIGMLGYCAEGKHRLLVYDYMENGSLAQ
NLDSSSNVLDWSKRYNIALGTARGLAYLHEECLEWILHCDIKPQNVLLDSDYQPKVADFG
LSKLLNRNSNLNNSNFSRIRGTRGYMAPEWVFNLPITSKVDVYSYGIVVLEMITGRSPTT
GVRVTELEAESHHDERLVTWVREKKMKASEVGSTWVDRIVDPALGSNYDMNQMEILATVA
LECVDEDKDVRPSMSQVAERLQNHENDS*

>Glyma.07G108400|Glyma.07G108400.1|10652106|GNA/S-locus glycoprotein/protein kinase
MGFSTFLLPLLVSLIFHNFQHSSSFSLSVEKFKEDVIVSSPKGKFTAGFYPVGDNAYCFA
IWYTQPPHTLVWMANRDQPVNGKRSTLSLLTTGNLVLTDAAQFMVWSTNTATSSKQVQLH
FYDTGNLVLLDNSDNVVVLWQSFDYPTDTLLPDQTLTKNSNLISSRSGTNYSSGFYKLFF
DSDNVLRLMYQGPRVSSVYWPDPWLLNNNLGIGGTGNGRTSYNDSRVAVLDEFGHFVSSD
NFTFKTSDYRTVLQRRLTLDPDGSVRVYSKNDGEDKWSMSGEFKLHPCYAHGICGSNSYC
RYEPTTGRKCLCLPDHTLVDNQDWSQGCTPNFQHLCNDNNTKYESRFLGMSLVSFYGYDY
GYFANYTYKQCENLCSRLCQCKGFLHIFSEENAFFECYPKTQLLNGNRQMDFKGSFFLRL
PLSHEEYENPVQNNDNGGLVCGGEGGGAKLLERQYAEEKENGSVKLMLWFASALGGIEVV
CIFLVWCFLFRNKNRKLHSGADKQGYVIATAAGFRKFSYSELKQATKGFSEEIGRGGGGT
VYKGVLSDNRVVAIKRLHEVANQGESEFLAEVRIIGRLNHMNLIGMLGYCAEGKHRLLVY

EHMENGSLAQNLSSSSNVLDWSKRYSIALGTAKGLAYLHEECLEWILHCDIKPQNILLDS
DYQPKVADFGLSKLLNRNSNLNNSSFSRIRGTRGYMAPEWVFNLPITSKVDVYSYGIVVL
EMITGRSPTAGIQITELEAASHHHERLVTWVRDKRRTRSKMGSSWVDQIVDPALGSKNYD
RNEMEILATVALECVEDEKDARPSMSQVAERLQNHEPYT*

>Glyma.07G173000|Glyma.07G173000.1|30309381|GNA/S-locus glycoprotein/protein kinase
MALCTSSFFFTFFFTFFLFLFNLQPSVSQQQFTSFNISHSPWLPAQNKTLLSPNKNFTAG
FFPLPNSSNVFTFSIWYSKVPPSANPFVWNATVQVNTSGSLEITPKGELLLNGSPFQSAE
NATTNSTSNSTQLLLQNDGNLVFGEWSSFKNPTSTVLPNQNFSTGFELHSNNGKFRFIKS
QNLVLSSTSDQYYNTPSQLLNMDDNGKMSMQGNSFLTSDYGDPRFRKLVLDDDGNLRIYS
FYPEQKNQWVEVWKGIWEMCRIKGKCGPNAICVPKEDLSTSTYCVCPSGFTPAIQNDPEK
GCRRKIPLSQNTQFLRLDYVNCSSDGHLNEIKADNFAMCEANCSREKTCLGFGFKYDGSG
YCMLVNGTNLQYGFWSPGTEAALFVKVDKSESSVSNFIGMTEVMQTTCPVNISLPLPPKD
SNATARNIAIICTLFAAELIAGVAFFWSFLKRYIKYRDMATTLGLELLPAGGPKRFTYSE
IKAATKDFSNLIGKGGFGDVYKGELPDHRVVAVKCLKNVTGGDAEFWAEVTIIARMHHLN
LVRLWGFCAEKGQRILVYEHIPGGSLDKYLFRVNKSHNNNHLKEQSSSLNPNTPQQERHV
LDWSMRYRIALGMARAIAYLHEECLEWVLHCDIKPENILLGDDFCPKISDFGLAKLRKKE
DMVTMSRRRGTPGYMAPEWITADPITSKADVYSFGMVLLELVSGIRNFEIQGSVVRSEEW
YFPGWAFDKMFKEMRVEEILDGQIRDAYDSRAHFEMVNRMVKTAMWCLQDRPELRPTMGK
VAKMLEGTVEITEPKKPTVFFLGEE*

>Glyma.07G188800|Glyma.07G188800.1|35624655|GNA/S-locus glycoprotein/PAN/protein kinase
MTTKRKHANFLLPLFFFFFFCSHTLFSHAADSITGDTVIRDNDGGDTLVSKDLTFEMGFF
SFDNSSRYVGIWYHEIPVKTFIWVANREKPIKGREGLIQIKTDGNLVVLDGERNEVWSTN
MSIPRNNTKAVLRDDGNLVLSEHDKDVWQSFEDPVDTFVPGMALPVSAGTSMFRSWKSAT
DPSPGNYSMKVDSDGSTKQILILEGEKRRRWRTGYWDGRVFTGVSDVTGSSLFGFGVTTN
VEGEEYFTYKWNSPEKVRFQITWDGFEKKFVWDEDGKQWNRTQFEPFNDCEHYNFCGSFA
VCDMGNSPVCSCMQGFQPVHWEEWNNRNWSRGCGRKTPLKAETERAANSSSSGAEVSVGE
DGFLEQRCTKLPDFARLENFVGYADCQSYCLQNSSCTAYSYTIGIGCMIWYGELVDVQHT
KNNLGSLLNIRLADADLGEGEKKTKIWIILAVVVGLICLGIVIFLIWRFKRKPKAISSAS
GYNNNSEIPVFDLTRSTGLSEISGELGLEGNQLSGAELPLFNFSYILAATNNFSDENKLG
QGGFGPVYKGKFPGGEEVAVKRLSRKSSQGLEEFKNEMVLIAKLQHRNLVRLLGCCIQGE
EKILVYEYLPNKSLDCFLFDPVKQTQLDWARRFEIIEGIARGLLYLHQDSRLRIIHRDLK
ASNILLDESMNPKISDFGLARIFGGNQNEANTNRVVGTYGYMSPEYAMEGLFSIKSDVYS
FGVLLLEIMSGRKNTSFRDTEDSSLIGYAWHLWSEQRVMELVDPSVRDSIPESKALRFIH
IGMLCVQDSASRRPNMSSVLLMLGSEAIALPLPKQPLLTTSMRKLDDGESYSEGLDVSND
VTVTMVTGR*

>Glyma.08G060500|Glyma.08G060500.1|4664282|GNA/S-locus glycoprotein/PAN/protein kinase
MHDASTRKEKIPKFRLGQIRAVRRLQSCSSSLVFITDSLFIIFFISKTPHLCNLNKDTGM
TTKRQHAILLLLLLFFCSHTLFSHAADSITEDTVIRDNDGGDNLVSKDLTFEMGFFGLDNN
NSSRYVGIWYHEIPVKTFIWVANREKPIKGREGSILIQKSNGNLIVLDGENNEVWSTNMS
VPRNNTKAVLRDDGNLVLSEHDKDVWQSFEDPVDTFVPGMALPVSAGTNIFRSWKSETDP
SPGNYSMKVDSEGSTKQILILEGEKRRKWRSGYWDGRVFTGVSDVTGSSLFGFTVITDTK
GEEYFTYKWNSPEKVRFQITWDGFEKKFVLDADGKQWNRTQFEPFDDCEKYNFCGSFAVC
DTGNSPFCSCMEGFEPMHWEEWNNRNWTRGCGRRTPLKAEAERSANNSSSGADREVSVGE
DGFLEQRCTKFPDFARLENFVGDADCQRYCLQNTSCTAYSYTIGIGCMIWYGELVDVQHS
QNNLGSLLHIRLADADLGDGGKKTKIWIILAVVVGLICIGIVVLLVWRFKRKPKAVSSAS
GFNNNSEIPAFDLTRSTDLSEISGELGLEGNQLSGAELPLFHFSCILAATNNFSDENKLG
QGGFGPVYKGKIPGGEEVAVKRLSRKSSQGLEEFKNEMVLIAKLQHRNLVRLLGCCIQGE
EKILVYEYLPNKSLDCFLFDPVKQTQLDWAKRFEIIEGIARGLLYLHRDSRLRIIHRDLK
ASNILLDESMNPKISDFGLARIFGGNQNEANTNRVVGTYGYMSPEYAMEGLFSIKSDVYS
FGVLLLEIMSGRKNTSFRDTDDSSLIGYAWHLWSEQRVMELVDPSLGDSIPKTKALRFIQ
IGMLCVQDSASRRPNMSSVLLMLGSESTALPLPKQPLLTTSMRILDDGESYSEGLDVSND
LTVSMVTTGR*

>Glyma.08G060800|Glyma.08G060800.1|4681172|GNA/S-locus glycoprotein/PAN/protein kinase
MGGSTNPLSLFLLCFTTFLTLFEVSISTDTLTSSQSLRTNQTLLSPNAIFELGFFSYTNS
TWYLGIWYKTIHDRDRTVVWVANRDIPLQTSLGFLKINDQGNLVIINQSQKPIWSSNQTT
TTPSNLILQLFDSGNLVLKEPNENDPKKILWQSFDYPTDTLLPGMKLGWNFDTGIEKHIT
SWSATNEDPSSGDFSFKLDPRGLPEIFLWNKNQRIYRSGPWNGERFSGVPEMQPNTDSIK
FTFFVDQHEAYYTFSIVNVSLFSRLSVNSIGELQRLTWIQSTQVWNKFWYAPKDQCDNYK
ECGAYGVCDTNASPVCQCIKGFRPRNPQAWNLRDGSDGCVRNTELKCGSDGFLRMQNVKL
PETTLVFVNRSMGIVECGELCKKNCSCSGYANVEIVNGGSGCVMWVGELLDVRKYPSGGQ
DLYVRLAASDVDDIGIEGGSHKTSDTIKAVGIIVGVAAFILLALAIFILWKKRKLQCILK
WKTDKRGFSERSQDLLMNEGVFSSNREQTGESNMDDLELPLFDFNTITMATNNFSDENKL

GQGGFGIVYKGRLMEGQNIAVKRLSKNSGQGIDEFKNEVKLIVKLQHRNLVRLLGCSIQM
DEKMLVYEYMENRSLDAILFDKTKRSSLDWQRRFNIICGIARGLLYLHQDSRFRIIHRDL
KASNILLDKEMNPKISDFGMARIFGTDQTEANTMRVVGTYGYMSPEYAMDGIFSVKSDVF
SFGVLVLEIISGKKNRGFYSANKELNLLGHAWKLWKEENALELIDPSIDNSYSESEVLRC
IQVGLLCVQERAEDRPTMASVVLMLSSDTASMSQPKNPGFCLGRNPMETDSSSSKQEESC
TVNQVTVTMLDAR*
>Glyma.08G061000|Glyma.08G061000.1|4694260|GNA/S-locus glycoprotein/PAN/protein kinase/SRK
MKFFSAIEFLSSFLVLMFFYPFCHSLDNTITINHPIRDGDVLVSNGLGNFALGFFSPRNS
TNRYVGIWYNKISEQTVVWVANRDTPLNDTSGVLKISNNGNLVLHDNSTRSLNPVWSSNV
SIESTNNISAKLLDTGNLVLIQTNNNNILWQSFDYPGNTMLPFMKLGLNRKTGLDRFLVS
WKSPNDPGTGNMTYKIDPTGFPQLFLYKDKIPLWRVGSWTGQRWSGVPEMTPNFIFTVNY
VNNESEVSIMYGVKDPSVFSRMVLDESGHVARSTWQAHEHRWFQIWDAPKEECDNFRRCG
SNANCDPYHADKFECECLPGFEPKFEREWFLRDGSGGCVRKSNVSTCRSGEGFVEVTRVK
VPDTSKARVAATIGMRECKERCLRDCSCVAYTSANESSGSGCVTWHGNMEDTRTYMQVGQ
SLFVRVDKLELAKYAKHPYGSLGKKGMVAVLTAAIFLFLLLAITFVYWFVKTRRQGIRRD
RKYSFRLTFDDSTDLQEFDTTKNSDLPFFELSSIAAATDNFSDANKLGQGGFGSVYKGLL
INGMEIAVKRLSKYSGQGIEEFKNEVVLISKLQHRNLVRILGCCIQGEEKMLIYEYLPNK
SLDSLIFDESKRSQLDWKKRFDIICGVARGMLYLHQDSRLRIIHRDLKASNVLMDSSLNP
KIADFGMARIFGGDQIAANTNRVVGTYGYMSPEYAMEGQFSVKSDVYSFGVLLLEIVTGR
KNSGLYEDITATNLVGHIWDLWREGKTMEIVDQSLGESCSDHEVQRCIQIGLLCVQDYAA
DRPSMSAVVFMLGNDSTLPDPKQPAFVFKKTNYESSNPSTSEGIYSVNDVSITMIEAR*

>Glyma.08G125800|Glyma.08G125800.1|9686176|GNA/protein kinase
MVICYKQLGEYHFFLVLLLISVQCVIAANNILKPGDTLNTRSQLCSENNIYCMDFSPLNT
NPIVNYTHLSISDNRKDDNSAVWVANRNQPVDKHSAVLMLNHSGVLKIESSKDAKPIILF
SSPQPLNNNNTEAKLLDTGNFVVQQLHPNGTNTVLWQSFDYPTDTLLPGMKLGVNHKTGH
NWSLVSWLAVSDPRIGAFRFEWEPIRRELIIKERGRLSWTSGELRNNNGSIHNTKYTIVS
NDDESYFTITTTSSNEQELIMWEVLETGRLIDRNKEAIARADMCYGYNTDGGCQKWEEIP
TCRHSGDAFETREVYVSMNMLNNLGNSSYGPSDCRDICWENCACNGYRNYYDGGTGCTFL
HWNSTEEANFASGGETFHILVNNTHHKGTKKWIWITVAVVVPFVICAFILFLALKKRKHL
FEEKKRNRMETGMLDSAIKDLEDEFKKRQNLKVFKYTSVLSATNDFSPENKLGQGGFGPV
YKGILPTGQEAAIKRLSKTSRQGVVEFKNELMLICELQHMNLVQLLGCCIHEEERILIYE
YMPNKSLDFYLFDCTRSKLLDWKKRFNIIEGISQGLLYLHKYSRLKVIHRDLKASNILLD
ENMNPKISDFGLARMFEEQESTTTTSRIIGTYGYMSPEYAMEGIVSVKSDVYSFGVLVLE
IISGRRNTSFNDDRPMNLIGHAWELWNQGVPLQLMDPSLNDLFDLNEVTRCIHIGLICVE
KYANDRPTMSQIISMLTNESVVVPLPRKPAFYVEREILLRKASSKELCTNSTDEITIT*

>Glyma.08G167500|Glyma.08G167500.1|13202201|GNA/protein kinase
MKLSPFLIFAWLCLRTTTHATTARDSLRPGEMLNNSSILTSAQKKFSLKFATIEIPNTSL
NTYLVIDRANTTGNVDWIGNRNDPLAYNSCALTLNHSGALIITRHNGDSIVLYSPAEATN
RTIATLLDSGNFVLKEIDGNGSTKNVLWQSFDHPEFVLLPGMKLGVNKKSGMSWLVKASI
SRAKPASGSFTLEWEPREGQLVIKRQGQVYWASGLLRNKRFEHIPEEVQLMYEYNIVSNE
EEESFSYTTPNGVSQWVLFHNGQLKDTTRGSEIARADTCYGYNTDGGCQRWEQPTCRKKS
DQRVKKRSDSRVFSYASIIAMTNRFSVENKLGEGGFGLVYKGKLPTGEDVAIKRLSKGSR
QGVIEFKNELNLISQLQHMNVIQILGCCIHGEERMLIYEKMLLDWKRRFNIIEGIAQGLL
YLHKYSRLKVVHRDLKASNILLDENMNPKISDFGTARIFSPQESEINTERIVGTYGYMSP
EYVTRGIFSIKSDVYSFGVLILEIVSGGRTNSFYSGERQCNLIGHAWELWQQGKGLELVD
PTIRDSCIEDQALRCIHVGLLCAEDNAVDRPTISDIINMLTSEYAPFPLPRRPAFYSRRM
PNEECRCTKGSECYSVNGLSISNVVAR*

>Glyma.08G176200|Glyma.08G176200.1|14059990|GNA/S-locus glycoprotein/protein kinase
MASTLLPFLFLSMVLLPFQTIAQTKSNIAIGESHTAGASTSPWLVSSPSGDFAFGFLPLE
DTPDHFMLCIWYAKIQDKTIVWFANRDQPAPKGSKVVLTADDGLVLITAPNGHMLWKTGG
LTLRVSSGVLNDTGNFVLQDGHSKTVWESFKDYRDTLLPYQTMEKGHKLSSKLGRNYFNK
GRFVLFFQNDGSLVMHSINMPSGYANENYYQSGTIESNTNTSTSAGTQLVFDGTGDMYVL
RKNNEKYNLSKGGSRASSTTQFYYLRATLDFDGVFTLYQHPKGSSGSGGWSQVWSHPDNI
CKDYVASAGSGVCGYNSICSLRDDKRPNCRCPKWYSLVDPNDPNGSCKPDFVQACAVDKL
SNRQDLYDFEVLIDTDWPQSDYVLQRPFNEEQCRQSCMEDCMCSVAIFRLGDSCWKKKLP
LSNGRVDATLNGAKAFMKVRKDNSSLIVPPIIVNKNNKNTSILVGSVLLGSSAFLNLILV
GAICLSTSYVFRYKKKLRSIGRSDTIVETNLRRFTYEELKKATNDFDKVLGKGAFGIVYE
GVINMCSDTRVAVKRLNTFLMEDVHKEFKNELNAIGLTHHKNLVRLLGFCETEEKRLLVY
EYMSNGTLASLLFNIVEKPSWKLRLQIAIGIARGLLYLHEECSTQIIHCDIKPQNILLDD
YYNARISDFGLAKLLNMNQSRTNTAIRGTKGYVALEWFKNMPITAKVDVYSYGVLLLEIV
SCRKSVEFEAEDEEKAILAEWAYDCYIEGTLHALVEGDKEALDDMKTFEKLVMIALWCVQ
EDPSLRPTMRNVTQMLEGVVEVKMPPCPSQFSVQYS*

>Glyma.08G236900|Glyma.08G236900.1|20084182|GNA/PAN/protein kinase
MTCSEQEKPHHVLLFLIYMWLWWSTTCIHVEANDSLKPGDTLNATVPGAELCSKKGKYCM

SFDPITHDNQEAVYLTICAQKKDDWEVWVANRNQPVDSNSAVLSLDHKGVLKIESQDGKK
KVKKSPIILYSPPQPINNTLATLLDTGNFVLQQLHPNGSKIRVLWESFDFPTDTLLPGMK
LGLNHKTGGTNWSLVSWLSGQVPTAGPFKLEWEPKTRELLIIKRGGSSSSGGKRVLWASG
NKLEHIPSEIRREIVPSETGDYFTLKSSDSEEEPTKWTLLSTGQLINRKGVDVARADMCH
GYNTDGGCQKWDAILPSCRRPGDAFELKYGYPKWDTEVKRDEENSSYGISDCQEICWRNC
SCVGFALNHRNETGCVFFLWDLVKGTNIANEGYKFYVLVRSNHQNRIKQWIWAMVATVAT
ILIICLCILRRVLKKRKHVLKENKRNGMEIENQDLAASGRSSSTDILEVYLKEEHDLKLF
SYASIIEATNDFSSENKLGQGGFGVVYKGILSTRQEVAVKKLSRSSGQGLIEFKNELTLI
SKLQHTNLVQLLGYCIHEEERILIYEYMSNKSLDFILFDSTQSHLLDWNKRFNIIEGIAQ
GLLYLHKYSRLRIIHRDLKASNILLDENMNPKISDFGIAKMFTQQDSEANTTRIFGTYGY
MSPEYAMEGIFSTKSDVYSFGVLLFEIVSGKRNNSFYTEERQLNLVGHAWELWKKGEALK
LVDPALNNDSFSEDEVLRCVHAGLLCVEENADDRPSMSNIVSMLSNKSKVTNLPKKPAYY
VRTKLLGEELETSTKEYGLDFLFENSLYVCSV*

>Glyma.08G307300|Glyma.08G307300.1|42551411|GNA/S-locus glycoprotein/protein kinase
MSSAIFLPFILSLSLSLSLLNGLDVIQLNTNITAGSNSTWKSPSGDFEFGFYDLRTGLFL
VGIWFGKIPDRTLAWYFQSPPVEANSQIQFTSAGNLVVAYPNQTIAQTIYSGGAATSSYM
QDDGNFVMKDSNSESVWQSFNSPSNTMLPGQTLQSTKVLYSKERGDSNYSLGKFMLQMQD
DGNLVLKAYQWSGPAYWYNSTNTPNVNLEFNATSALMHFVSGSRSIYTLTKSTSTPVEDY
YHRATIDENGNFQQYAYPRRNENDTTGWRRVWRAVEDPCRVNLVCGVYGLCTSPDNESVK
CECIPGYIPLDHQDVSKGCHPPDTINYCAEKKFKVEVFGDTDFQFDNNFVRVYDVDLEGC
KKSLMDDCNVIAATYNTSTRTCAKKRLPLLNARNSSSSKGQKALLKVPNSVDDGRSNKAT
NKKSFNVRIFLKVMLAVTATLACFFGALVVYYHPFTQRLARRKRYLNASAIGINFREFTF
QELHEATDGFTRILGRGSSGKVYHGTLIIDDAVIGIAVKKLEKKIEKSESEFMTELKIIG
RTHHRNLVRLLGFCIESSHRVLVYELMTNGALSSFLFGEGERPQWGQRIEMALGVARGLL
YLHEECHTQIIHCDIKPQNVLLDSNHTAKIADFGLSKLLLKDQTRTSTNLRGTIGYMAPE
WLKSAPITAKVDIYSFGVMLLEIICCRRHFESPHDANDSEDDDLVLSNLVLRSVVSRKLE
VVVRHDSEVLNDFKRFEEMALVGLWCVHPNPALRPSMKHVMQMLNGTVEVGIPPLVYDQM
MEDQGL*

>Glyma.08G307400|Glyma.08G307400.1|42556212|GNA/S-locus glycoprotein/protein kinase
MVTKPLVLFVSFFLCSSVLRNANSIELGSSIVAGTNNSSWRSSNGDYAFGFYHLLSGHYL
VGIWFDKVPNKTLVWSANRDNPVEIGSTINLTSSGEFLLQPVKGATFQIYKGTNTPAATA
KMEDNGNLVLRNSLSEFIWQSFDSPTDTLLLGQTLKMGQKLYSNANGSVDYSKGQYSLEI
QQSDGNIVLKAFRFTDAGYWSSGTNQNTDVRIVFNSTTAFLYAVNGTNQTIHNMTVDPLT
GAIEDYYHRVLIDDRGNLQKLIHPKENGSDWTSVWNAIELPCRVTALCGVYGFCNSSDNQ
SYSCECLPGYTHLDPNVPSKGCYLSTEANGLCAANSSKVEVKAIQDADIPNNDYFYFDLQ
VINNMDLESCKRELMDDCLCMAAVFYGSDCHKKTWPVINAIKIFPDTSNRVMLIKVPLLD
NDMENEKDSQSLVVLIVALVSCSLLAVLFAATFIYHHPIICQHLIHKGEPPKPKPMDINL
KAFSFQQLREATNGFKDKLGRGAYGTVYSGVLNLEGQQVEVAVKQLEQVEEQGEKEFVTE
VQVIAHTHHRNLVGLLGYCNEQNHRLLVYEKMENGTLSNFLFGEGNHRPSWESRVRIVIE
IARGLLYLHEECDQQIIHCDIKPQNVLLDSSYTAKISDFGLAKLLMKDKTRTSTNARGTV
GYMAPEWLKNAPVTTKVDIYSFGVVLLETIFCRRHIELHRINDETTGGDDMILIDWVLYL
AKENSLRAAVVDDLEVESDFKRFERMVMVGLWCVYPNSTLRPSMKVVAQMLEGNIEVGVP
PLN*

>Glyma.08G352100|Glyma.08G352100.1|46524724|GNA/S-locus glycoprotein/PAN/protein kinase
MGSSSCVKFFFVFILCCHVLDVGTAIDTITSSQSIKDTETLTSTDGNFTLGFFTPQNSTN
RYVGIWWKSQSTVIWVANRNQPLNDSSGIVTISEDGNLVVLNGHKQVIWSTNVSKTSFNT
SSQFSDSGKLVLAETTTGNILWDSFQQPSNTLLPGMKLSINKSTGKKVELTSWESPYNPS
VGSFSSSLVQRKNIVELFIFNGTQLYWRSGPWNGGIFTGIAYMSTYLNGFKGGDDGEGNI
NIYYTVSSELGPLGFLIYMLNSQGRLEEKWWDDEKQEMGLMWASRKSDCDIYAICGSFAI
CNAQSSPICSCLKGFEPRNKEEWNRQHWTSGCVRNTGLLCERVKDQNTSIDTNEDGFLEL
QMVKVPDFPERSPVDPDKCRSQCLENCSCVAYSHEEMIGCMSWTGNLLDIQQFSSNGLDL
YVRGAYTELEHDEGTNTTIIIIITVTIGTVFIVICACAYVMWRTSNHPAKIWHSIKSGRK
RGNKYLARFNNGVPSEHTSNKVIEELSQVKLQELLLFDFERVVAATNNFHLSNKLGQGGF
GPVYKGKLPDGQEIAVKRLSRASGQGLEEFMNEVVVISKLQHRNLVKLFGCCAEGDEKML
IYEYMLNKSLDVFIFDPSKSKLLDWRKRCGIIEGIGRGLLYLHRDSRLKIIHRDLKASNV
LLDEALNPKISDFGMARIFGGTEDQANTNRVVGTYGYMSPEYAMQGLFSEKSDVFSFGVL
VIEIVSGRRNSRFYDDDNALSLLGFAWIQWREGNILSVIDPEIYDVTHHKDILRCIHIGL
LCVQERAVDRPTMAAVISMLNSEVAFLPPPDQPAFVQSQNMLNLVSVSSEERQKLCSINV
LSQYGGQKHKPVGGKH*

>Glyma.08G352200|Glyma.08G352200.1|46533648|GNA/S-locus glycoprotein/PAN/protein kinase
MGFSSRANLFFVLLMLCCCVLDVGIAIDTITSSQSIKDPEVLTSKDGNFTLGFFTPQNST
NRYVGIWWKSQSTIIWVANRNQPLNDSSGIVTIHEDGNLVLLKGQKQVIWTTNLSNSSSN

RTSQFSDYGKLVLTEATTGNILWDSFQQPSNTLLPGMKLSTNNSTGKKVELTSWKSPSNP
SVGSFSSGVVQGINIVEVFIWNETQPYWRSGPWNGRLFTGIQSMATLYRTGFQGGNDGEG
YANIYYTIPSSSEFLIYMLNLQGQLLLTEWDDERKEMEVTWTSQDSDCDVYGICGSFAIC
NAQSSPICSCLKGFEARNKEEWNRQNWTGGCVRRTQLQCERVKDHNTSTDTKEDGFLKLQ
MVKVPYFAEGSPVEPDICRSQCLENCSCVAYSHDDGIGCMSWTGNLLDIQQFSDAGLDLY
VRIAHTELDKGKNTKIIIIITVIIGAVVIITCSCAYVMRRTSNHPAKIWHLIKLRKGNRN
GFVQSKFDETPEHPSHRVIEELTQVQQQEMFVFDFKRVATATNNFHQSNKLGQGGFGPVY
KGKLQDGQEIAVKRLSRASGQGLEEFMNEVVVISKLQHRNLVRLFGSCIEGEEKMLLYEY
MPNKSLDVFIFDPSKSKLLDWRKRISIIEGIARGLLYLHRDSRLRIIHRDLKASNILLDE
ELNPKISDFGMARIFGGTEDQANTLRVVGTYGYMSPEYAMQGLFSEKSDVFSFGVLVLEI
VSGRRNSSFYDNENFLSLLGFAWIQWKEGNILSLVDPGTYDPSYHKEILRCIHIGFLCVQ
ELAVERPTMATVISMLNSDDVFLPPPSQPAFILRQNMLNSVSSEEIHNFVSINTVSITDI
HGR*

>Glyma.08G352300|Glyma.08G352300.1|46539457|GNA/S-locus glycoprotein/PAN/protein kinase
MCFSSCANLFFVLFILFCYVLDVAIAVDTITSSQPVKDPETLRSKDGNFTLGFFSPQNSK
NRYVGIWWKSQSTVVWVANRNQPLNDSSGIITISEDGNLVVLNGQKQVVWSSNVSNTSSN
TTSQFSDYGKLVLTETTTGNILWDSFQQPSDTLLPGMKLSSNSTSMRVKLASWKSPSNPS
VGSFSSGVVERINILEVFVWNETQPYWRSGPWNGGIFTGIPSMSPYRNGFKGGDDGEANT
EIYYTVPSALTFTIYMLNSQGQYEEKWWYDEKKEMQLVWTSQESDCDVYGMCGPFTSCNA
QSSPICSCLKGFEPRNKEEWNRQNWTGGCVRRTQLQCERVKDHNTSRDTKEDGFLKLQMV
KVPDFPEGSPVEPDICRSQCLENCSCVAYTHDDGIGCMSWTGNLLDIQQFSEGGLDLYIR
VAHTELDKGTNTKIIITITVIIGTVMIVTCAYVMWRTSNHPGRIWNLIKSARKGNNRAFV
RFNNDETPNHPSHKVIEELSQVTLPELLLFNFERVATATNSFDLSNKLGQGGFGPVYKGK
LQDGQEIAVKRLSRASGQGLEEFMNEVVVISKLQHRNLVRLFGCCAEGDEKMLIYEYMPN
KSLDVFIFDQSRSKLLDWRKRSSIIEGIARGLLYLHRDSRLRIIHRDLKASNILLDEELN
PKISDFGMARIFGGTEDQANTNRIVGTYGYMSPEYAMQGLFSEKSDVFSFGVLVLEIVSG
RRNSSFYDNVHALSLLGFAWIQWREGNTLSLMMDQEIHDPSHHEDILRYIHIGLLCVQEH
AVDRPTMAAVISMLSSELALPPPSQPAFILQQNMLNLASSEETLRCCSINIVSVTDIQGR
*

>Glyma.08G352400|Glyma.08G352400.1|46545324|GNA/S-locus glycoprotein/protein kinase
TPSQSIKDPETLRSKDGKFTLGFFTPQNSTNRYVGIWWMSQSTIIWVADRNQPLNDSSGK
VTISEDGNLVVLNGTKQVIWSSNMSNITSNIQAQSFQIFHPSNTLLPGLELTTNIRAGLK
VELTSWKSPSNPSIWSFSSNIVQRINLIELLIWNGTRPYWRSGPWNGRLFTWIPNTDSAY
LNGFQEETRWDDEKKDTEVSWTSPESDCDVYSICGSFSICNAQSSQICNCLKGFEPRIKG
EWDRQNWSSGCVRSTGLQCETTGCMSWTGNLLDIQQFSSAELVLYVRVAYAELEHARKRN
NKASLLFNNDETSEHPSQKVIEELSQVTLPELLQLDFEMVAIATNNFHLPNKLGQGGFGP
VYKGKLQDGQEIAVKRLSRASGQGLEEFMNEVVVISKLQHRNLVKLFGCCIEGDEKMLIY
EYMPNKSLDVLIFDPSKSKLLDWRKRYSIIEGIARGLLYMHRDSRLRIIHRFEDK*

>Glyma.08G354900|Glyma.08G354900.1|46763083|GNA/S-locus glycoprotein/PAN/protein kinase
MALSITLFLLASLLSFQISSSSSSLRKGSSLSVENPQHVLVSPNGMFSAGFLAIGENAYS
FAIWFTEPHFHSPNTVTWMANRDQPVNGKGSKLSLTHAGNIVLVDAGFNTAWSSNTASLA
PAELHLKDDGNLVLRELQGTILWQSFDFPTDTLVPGQPLTRHTLLVSARSESNHSSGFYK
FFFSDDNILRLVYDGPDVSSNYWPNPWQVSWHIGRTLFNSSRIAALNSLGRFRSSDNFTF
VTFDYGMVLQRRLKLDSDGNLRVYGRKSAVEKWYVSWKAIRNDCIIHGVCGPNSTCGYDP
KSGRTCKCLPGYRLRNHSDWSYGCEPMFDLTCNWNETTFLEMRGVEFYGYDNYYVEVSNY
SACENLCLQNCTCQGFQHSYSLRDGLYYRCYTKTKFLNGQRLPRFPGTTYLRIPKSYSLS
VKESAIDSVDDHHVCSVQLQRAYIKTLESRVVRVLLWFAAALGAFEMVCIFVVWCFLIRT
GQKSNADQQGYHLAATGFRKFSYSELKKATKGFSQEIGRGAGGVVYKGILSDQRHAAIKR
LNEAKQGEGEFLAEVSIIGRLNHMNLIEMWGYCAEGKHRLLVYEYMENGSLAQNLSSNTL
DWSKRYNIVLGTARVLAYLHEECLEWILHCDIKPQNILLDSNYQPRLADFGLSKLLNRNN
PNNPSISMIRGTRGYMAPEWVFNLPITSKVDVYSYGIVVLEMVTGKSPTTSIDDINGEET
YDGRLVTWVREKRSNSNTSWVEQIIDPVIGLNYDKSKIEILITVALKCVLEDRDSRPNMS
QVVEMLQCHGSDSH*

>Glyma.08G355000|Glyma.08G355000.1|46767745|GNA/S-locus glycoprotein/PAN/protein kinase
MAFSISLFLLVLLFSFQSSSSSLLSLNKGSSLSVEKHAEDVIVSPNQMFCAGFFQVGENA
FSFAIWFNDPHTHNNNHTVVWMANRETPVNGRLSKLSLLNSGNMVLVGAGQITTWSSNTA
SDAPVKLHLQDDGNLVLLDLQGTILWQSFDTPTDTLLPGQLLTRYTQLVSSRSQTNHSPG
FYKMLFDDDNVLRLIYDGPDVSSTYWPPPWLLSWQAGRFNYNSSRVAVLNSIGNFTSSDN
YDFSTDDHGTVMPRRLKLDSDGNARVYSRNEALKKWYVSWQFIFDACTSHGICGANSTCS
YDPKRGRRCSCLPGYRVKNHSDWSYGCEPMFDLTCSRNESIFLEIQGVELYGYDHNFVQN
STYINCVNLCLQDCNCKGFQYRYDGNQIFSCFTKSQLLNGRRSPSFNGAIYLRLPITNNF
SKEESVSADDHVCSVKLHKDYVRKPENRLVRFFLWLATAVGALEVIFFFLIWGFLIWNLK

TSSADQQGYHLAAVGFRKYSYLELKKATKGFSQEIGRGAGGIVYKGILSDQRHVAIKRLY
DAKQGEGEFLAEVSIIGRLNHMNLIEMWGYCAEGKHRLLVYEYMENGSLAQNLSSNTLDW
SKRYSIALGTARVLAYLHEECLEWILHCDIKPQNILLDASYQPKVADFGLSKLLNRNNLN
NSSFSMIRGTRGYMAPEWVLNLAITSKVDVYSYGIVLLEMITGKSPTTTGVQNIDGEEPY
NGRLVTWVREKRSATSWLEHIIDPAIKTNYDECKMNLLATVALDCVEEDKDVRPTMSHVV
EMLQSHESDPRNIINA*

>Glyma.08G355100|Glyma.08G355100.1|46778500|GNA/S-locus glycoprotein/PAN/protein kinase
MDFPTSLFFLLLLLSFQCSSSLSSLNKGSSLSVEKHTQDSIVSPNQMFCAGFFQVGENAF
SFAIWFNDPHTHNNNHNNRNVVWIANREQPVNGKLSKLSLLNSGSIVLLDADQITTWSSN
TASNAPLELNLQDDGNLVLRELQGTILWQSFDSPTDTLLPGQPLTRYTQLVSSRSKTNHS
SGFYKLLFDNDNLLRLIYDGPDVSSSYWPPQWLLSWDAGRFSFNSSRVAVFNSLGIFNSS
DNYGFSTNDHGKVMPRRLTLDSDGNVRVYSRNEASKKWYVSWQFIFETCTVHGVCGVNST
CNFDPKRGRICSCLPGHTVKNHSDWSYGCEPMFNLSCNGNDSTFLELQGFEFYGYDSNYI
PNSTYMNCVNLCLQDCNCKGFQYRYDGEYSTCFTKRQLLNGRRSTRFEGTIYLRLPKNNN
FSKEESVSAYGHVFSVQLHKEYVRKPENRFVRFFLWLATAVGALEVVCFLIIWVFLIKTR
QKSGADQQGYHQAEMGFRKYSYSELKEATKGFNQEISRGAEGIVYKGILSDQRHVAIKRL
YEAKQGEEEFLAEVSIIGRLNHMNLIEMWGYCAEGKHRLLVYEYMENGSLAQNLSSNTLD
WSKRYSIALGTARVLAYLHEECLEWILHCDIKPQNILLDANYQPKVADFGLSKLLNRNNL
NNNLRFSVIRGTRGYMAPEWVYNSPITSKVDVYSYGIVLLEMITGKNPTTGVHSNAGEES
YNGRLVTWVREKRGDASWLEHIIDPAIKTNFDECKMDLLARVALDCVEVNKDRRPTMSQV

VEMLQSHDCLRC*

>Glyma.08G355200|Glyma.08G355200.1|46783898|GNA/S-locus glycoprotein/PAN/protein kinase
MAFSISLFLLVLLFSFQSSSSSLLSLNKGSSLSVEKHAEDVIVSPNQMFCAGFFQVGENA
FSFAIWFNDPHTHNNNHTVVWMANREQPVNGRLSKLSLLNSGNMVLVDAGQITKWSSNTA
SHAPVKLHLQDDGNLVLLDLQGTILWQSFDTPTDTLLPGQLLTRHTQLVSSRSQTNHSPG
FYKMLFDDDNVLRLIYDGPDVSSTYWPPPWLLSWQAGRFNYNSSRVAVLNSIGNFTSSDN
YDFSTDDHGTVMPRRLKLDSDGNARVYSRNEALKKWHVSWQFIFDTCTIHGICGANSTCS
YDPKRGRRCSCLPGYRVKNHSDWSYGCEPMFDLACSGNESIFLEIQGVELYGYDHKFVQN
STYINCVNLCLQDCNCKGFQYRYDGNQIFSCYTKLQLWNGRRSPSFNGTINLRLPNSNNF
SKEESESADDHVCSVQLHKDYVRKAANRFERFSLWLATAVGALEMICLLMIWGFLIRSQQ
KSSANKLGYHLAAVGIRKYSYSELKKATEGFSQEIGRGAGGVVYKGILSDQRHAAIKRLY
DAKQGEGEFLAEVSIIGRLNHMNLIEMWGYCAEGNHRLLVCEYMGNGSLEENLSSNTLDW
SKRYNIALGVARVLAYLHEECLEWILHCDIKPQNILLDASYQPKVADFGLSKLLNRDNLH
SNSTVSMIRGTRGYMAPEWVYNLPITSKVDVYSYGIVLLQMITGKSPTTGVQSIDGEESH
NGRLVTWVREKRSATSWLEQIMDPAIKTNYDERKMDLLARVALDCVEEKKDSRPTMSQVV
EMLQSHETN*

>Glyma.09G003100|Glyma.09G003100.1|263802|GNA/protein kinase
MLLESTKMASSTFLRSYFLLLLLIIILPFLPSVFSATSSNCNVDLNSSLVTNGTWNSPSGH
FAFGFQSVLFDNKEFMSVLAVWFAKDPNRTIVWYAKQKQSPAFPSGSTVNLTNKGIVVND
PKGHEMWHRPENNTTIALVSCASMLDNGSFVLLDESGKQVWESFEEPTDTILPGQNLAKP
KTFRARESDTSFYNGGFELSWQNDSNLVLYYSPQSSDDQASQSPTGEAYWATGTFKTESQ
LFFDESGRMYIKNDTGTVISEITYSGPEEFFYMARIDPDGVFRLYRHPKGENTVADSCSS
GWWSVVQQYPQDICLSFTKQTGNVICGYNSYCITINGKPECECPDHYSSFEHDNLTGCRP
DFPLPSCNKDGWEQNKDLVDFKEYTNLDWPLSDYDKLVATAMDKDMCKQKCLEDCFCAVA
IYGEGQCWKKKYPFSNGRKHPNVTRIALVKVPKRDLDRGGREQTTLVLVISILLGSSVFL
NVLLFVALFVAFFIFYHKRLLNNPKLSAATIRSFTYKELEEATTGFKQMLGRGAFGTVYK
GVLTSDTSRYVAVKRLDKVVQEGEKEFKTEVSVIGQTHHRNLVRLLGYCDEGEHRLLVYE
HMSNGSLASFLFGISRPHWNQRVQIALGIARGLTYLHEECSTQIIHCDIKPQNILLDELF
TPRIADFGLAKLLLAEQSKAAKTGLRGTIGYFAPEWFRKASITTKIDVYSFGVVLLEIIC
CKSSVAFAMANDEEALIDWAYRCYSQGKVAKLVENDEEAKNDIKRVEKHVMVAIWCIQED
PSLRPSMKKVTQMLEGVTTVSVPPRPSIFSSSSFEISFTS*

>Glyma.09G099100|Glyma.09G099100.1|17829353|GNA/S-locus glycoprotein/PAN/protein kinase/SRK
MAIPPLTLICKLLWLLFSQICYATDTITQDQQLSDDGSTLVSNGGTFELGFFNPGSSNNR
YVGIWYKKISIKTVVWVANRDNPIVRHNSSKLVIRQEGNLVLLSNNNQSLLWTTNVTKKA
SSSSPIVQLLDTGNLVIKDGINEESVFLWQSFDHPCDTLLSGMKLGWDLRTGLNRRLTSW
KSWDDPSSGDIVWEVVIGNNPELVMWKSKVDYFRTGPYTGNMFSGVYAPRNNPLYNWKFV
SNKDEVYFQYTLSNSFVVSIIVLNQTLNLRQRLTWIPDTKTWTVYQSLPLDSCDVYNTCG
PNGNCIIAGSPICQCLDGFKPKSPQQWNAMDWRQGCVRSEEWSCGVKNKDGFQRLASMKL
PNTTFSWVNESITLEECRAKCLENCSCTAYSNLDTRGGGSGCSIWVGELVDMRDVKSGQD
LYVRIATSDPDGKHERQKKVILVVAITVSLVLVMLLAFCVYMIKKKYKGKTEIRMSIEQK
DQGGQEDLELPFFDLATIIITATNNFSINNKLGEGGFGPVYKGLLVDEQEIAIKRLSRSSG
QGLKEFRNEVILCAKLQHRNLVKVLGYCIEGEEKMLVYEYMPNKSLDLILFNSVESKFLD

WPMRFNILNAIARGLLYLHHDSRLRIIHRDLKASNILLDNDMNPKISDFGLARLCGSDQV
EGSTSIIAGTHGYMAPEYAIDGLFSIKSDVFSFGVLLLEIVSGKKNKGLTYQDHDHNLIG
HAWRLWKEGTPEQLIDACLANSCSIYEVARCVQISLLCLQHHPDDRPNMTSVVVMLSSEN
VIPEPKELGFLIRRVSNEREQSSNRQSSSINEVTMSLLNAR*

>Glyma.09G099200|Glyma.09G099200.1|17845393|GNA/S-locus glycoprotein/PAN/protein kinase/SRK
MAIPLVILLICKLLSLFSQICYATTDTITKGQPLPDDGNTLLSKDGTFELGFFNPGSSNN
RYVGIWYKNIVVKTVVWIANRDNPIRNNSSKLVISQDGNLVLLSQNESLIWTTNASSSEV
SSSSPIVQLLDTGNLVIKDGNDKESVFLWQSFDYPCDTLLPGMKFGWDLRTGLNRRLTSW
KSWDDPSSGDFTWGVEIGSNPDIVMWKGNVEYFRTGPYTGNMFSGVYGPRNNPLYDYKFV
NNKDEVYYQYTLKNSSVITMIVMNQTLYLRHRLTWIPEAKSWTVYQSLPRDSCDVYNTCG
PNGNCIIAGSPICQCLDGFEPKSPQQWNVMDWRQGCVRSEEWSCGVKNKDGFRRFASMKL
PNTTFSWVNESMTLEECRAKCLENCSCKAYSNLDTRGGGNGCSIWVGDLVDLRVIESGQD
LYVRMATSDMDGKHEHRRKVVLVVSTIASLVLVMLVAFCIYMIKKIYKGKTKTRMSREDK
DEGRQEDLELPFFDLATIVNATNNFSIENKLGEGGFGPVYKGTLVNGQEIAIKRLSRSSG
QGLKEFRNEVILCAKLQHRNLVKVLGYCIQGEEKMLLYEYMPNKSLDLFLFDSEQSKFLN
WPVRFNILNAIARGLLYLHQDSRLRIIHRDLKASNILLDNNMNPKISDFGLARMCGSDQV
EGSTSIIVGTHGYMAPEYAIDGLFSTKSDVFSFGVLLLEIISGKKNRAFTYQDNDHNLID
HAWRLWKEGTPERLTDAHLANSCNISEVIRCIQISLLCLQHHPDDRPNMTSVVVMLTSEN
ALHEPKEPGFLIRRVSNEGEQSSNRQTSSFNEVSISLLNAR*

>Glyma.09G278200|Glyma.09G278200.1|49362681|GNA/PAN/protein kinase
MLLLLLPLMRLFPFLFLLLVLLFNYGFSESGVGAGYQLMVAVPVEYEVNFKGRAFLVETN
QTAPNFRVALSIEAINGKYSCSLEVFLGDVKVWDSGHYSRFYITEKCLLELTMDGDLRLK
GPKERVGWKTGTSGQGVKRLEIQRTGNLVLVDALNNIKWQSFNFATNVMLRGQQLDVATR
LTSSQSNSSLFYFSFEIEDKKVALYLNYGKLRYSYWGFQPTMNRSITYIKLSSRGLLLFD
VKYKKIAQIPSEGIHPLRFLALNNETGNLGLYHYSPEKGKFEASFQALNSTCDLPISCRP
YGICTFSNSCSCIQLLANENKGGADCSGGITGGFCNGKEAEMLEIDNISSVLKNVTRVVN
ISKKTCESLCLQDCKCAAALYFGNASTDEAECYIYRLVLGLKQVKKGTGFSYMVKVPKGT
IKNHERHNVKRWVLIVAGGVDGLIILLLVGGFGYWLVKRRSHSLHSRAGNT*

>Glyma.10G100600|Glyma.10G100600.1|18396083|GNA/S-locus glycoprotein/PAN/protein kinase
MLGQENCVLKSPFLLCIFIGFLMHSVVGAEIPLGSKLSVVDNDYWVSSNGDFAFGFYNIS
DQPNQFSVGIRFNSKSIPYSQQTVAWVAGGDVKVGNKSYFELTQEGELVLFDSIGEGSVW
TVKTGNQSVASASLLDNGNLVLMDKEQKIIWQSFDTPSDTLLPGQSLFANETLRAATASK
NSKASYYTLHMNASGHLELHWESGVIYWTSENPSASNLRAFLTASGALELQDRSLKPVWS
AFGDDHNDSVKYRYLRLDVDGNLRLYSWVESLGSWRSVWQAVENQCKVFATCRQLGVCVF
NASGSAECKCPFEVTGGNECLVPYEEECESGSNMIAYKNTYLYAFYPPDNSFITSSLQQC
EQLCLNDTQCTVATFSNDGTPQCSIKKTEYITGYSDPSVSSISFVKRCSGPFAVNPGTFF
ILVIFQMGIVLFIYRRKNSTRKRSTLTFTGTNSKGLIVLSFSEIKSLTGDFKNQIGPKVF
KGLLPNNHPIAVTDLNASLEERKFRSAVMKMGCIHHKNLVKLEGYCCEFDHRFLVYEYCK
KGSVDKYIDDDALCKVLTWRKRVEICSSVAKAICYLHSGCREFISHGNLKCENVMLDENL
GAKVTEFGFAIADGKATYCGFSAEKDIEDFGKLVLTLLTGCRNHDHIELCEWAYKEWMEE
RVANVVDKRMEGGYKSEELEHVLRIAFWCLQMDERRRPSMGEVVRVLDGTLSVDPPPPPF
AFQRPLQVDDSEENVSELEV*

>Glyma.10G228900|Glyma.10G228900.1|45897641|GNA/S-locus glycoprotein/protein kinase
MASAASIFPCNWVVLALCLCCFSGCISAQIGLGSQLLASKAQTWVSENGTFALGFTPAET
DNRLLVIGIWFAQLPGDPTLVWSPNRDTPVSQEAALELDTTGNLVLMDGDTTVWTSNTSG
ADVQTATMSETGNFILHSTNNHSVWQSFSQPSDTLLPNQLLTVSSELTSSKSSSHGGYYA
LKMLQQPTSLSLALTYNLPETYQASDESYTNYSYWQGPDISNVTGEVIVVLDQAGSFGIV
YGDSSDGAVYVYKNDGGDDAGLSSAVHQSAPLTVLRRLTLEKNGNLRLYRWDEVNGSRQW
VPQWAAVSNPCDIAGVCGNGVCNLDRSKTKATCTCLPGTAKVGRDGQCYENSSLVGKCNG
KHENLTSQLRISTVQQTNYYFSEFSVIANYSDISNVSKCGDACLLDCDCVASVYGLNEER
PYCWVLRSLSFGGFEDTSSTLFVKVRANGSWTLEGQEGGSNSSSDGMGSAKEKAVIIPTV
LSMVVLIVLLSLLLYYTVHRKRTLKREMESSLILSGAPMNFTYRDLQIRTCNFSQLLGTG
GFGSVYKGSLGDGTLVAVKKLDRVLPHGEKEFITEVNTIGSMHHMNLVRLCGYCSEGSHR
LLVYEFMKNGSLDKWIFPSYQARDRLLDWTTRFNIAIATAQGIAYFHEQCRDRIIHCDIK
PENILVDENFCPKVSDFGLAKLMGREHSHVVTMVRGTRGYLAPEWVSNRPITVKADVYSY
GMLLLEIIGGRRNLDMSFGAEDFFYPGWAYKEMTNGSIIKVADKRLNGAVDEEEVTRALK
VAFWCIQDEVSMRPTMGEVVRLLEDSIDINMPPMPQTVLELIEEGLDQVYKAMKREYNQS
SSFTITSHLTSHATCSNSTMSPR*

>Glyma.11G036600|Glyma.11G036600.1|2635652|GNA/protein kinase
MLSGTNRIILYIIQVLTSRVFAKVELNKPLTTDDNNAWRSASGEFAFGFRQLNNDTKLFM
VAIWYNMMPGDQTVVWSAKRGYKLATAPTGSRIQITSEGLVLTGPKGDSIWIANSKDIVS
EGAMLDSGNFVLLNGNSEHVWQSFDYPTDTLLPNQSLQLGGVLTSRLTDTNFTTGRFQLY

FHKGDSHVLLCPLGWPSQLRYESYHTIDDSGNASQLVFDKSGDIYVETTNGTRIQPQGST
WGNSNLDLDRNYYRATLDFTGVFTQYAYPRNSTIAQPGWKIIRYVPGNICEAVNQYGSGC
CGYNSYCSMENQRPTCTCLYGYSLVDPSNPFGGCQLNFTLTCGADVQAPPDELYEMHEFK
NFNFPLADYEKIQPYTRQECQQACLQDCMCALAISGDFCWKKRLPLVNGREPKLKKVVQA
SSLLETNLHSFSYEALKEATWGFSEELGRGSCGIVYKGKLEAATSCNLIAVKRLDRLTQE
REKEFRTELSAIGKTCHKNLLLVYEFMSNGTLADILFGQSKAPIWNTRVRLALGIARGLL
YLHEECDSAIIHCDIKPQNILIDEHFNAKISDFGLAKLLLFDQSRTNTMIRGTRGYVAPE
SFKNVAVTVKVDVYSFGVMLLEMICCRRSVMTMEAGEEEKAILTDWAYDCCVEGKLHDLV
ENDKEALSDIGRLEKWIKIAIWCIQEHPEMRPTMGKVNQMMEGLVEVPNPPSPNPDV*

>Glyma.11G036700|Glyma.11G036700.1|2642437|GNA/protein kinase
MASPTLFFIFSLVFLHVMFVLGNITLSSTLSTNDNDAWLSPSGEFAFGFRQLNSTNLFVV
AIWYDKIPAKTIVWNAKANETLATAPAGSQVQLTLEGLTLTSPKGESIWKAQPSVPLSYG
AMLDTGNFVLVNKNSTFEWESFKNPTDTLLPNQFLELDGKLTSRLQDTNYTTGRFQLYFQ
NGVLLLSPLAWPTQLRYRYYYRIDASHSASRLVFDELGNIYVERVNGTRIRPQGPTWGNS
SLDPKEYYYRATLEFNGVFTQYAHPRTNNAYQGWTIMRYVPGNICTAIFNEYGSGSCGYN
SYCSMENDRPTCKCPYGYSMVDPSNEFGGCQPNFTLACGVDVKAQPEELYEMHEFRDFNF
PLGDYEKKQPYSQQECRQSCLHDCICAMAVLGGNTCWMKRLPLSNGRVIHVNDQHFVYIK
TRVRRDFYDPGANEELPPGADSKKEDGAKPILLGSLIGSLVFISISMLLCAVSWFILLKP
KLTRLVPAIPSLLETNLHSFTYETLEKATRGFCEEIGRGSFGIVYKGQLEAASCNVIAVK
RLDRLAQEREKEFRAELSAIGKTCHKNLVRLIGFCDEGINRLLVYEFMSNGTLADILFGQ
SKAPIWNTRVGLALGIARGLLYLHEECDSAIIHCDIKPQNILIDEHFNAKISDFGLAKLL
LFDQTRTNTMIRGTRGYVAPEWFKNIAVTVKVDVYSFGVMLLEIICCRRNVLTMEAEEEE
KVILTDWAYDCYIEGRNIDALVENDEEALSDNGRLEKWIKIAFWCINENPEVRPTMGMVM
LMLEGFVEVPNPPPPFSMHSIS*

>Glyma.11G152200|Glyma.11G152200.1|12092389|GNA/S-locus glycoprotein/PAN/protein kinase/SRK
MLIMVRFFFFCFFISTLLIQGTLAIITPNESIQGNRTLVSSAGTFEAGFFNFGNSQGQYF
GIWYKNISPKTIVWVANKDAPVKDSTAFLTLTHQGDPVILDGSRSTTVWFSNSSRIAEKP
IMQLLDSGNLVVKDGNSKKENFLWESFDYPGNTFLAGMKLRTNLVSGPYRSLTSWKNAED
PGSGEFSYHIDAHGFPQLVTTKGEILFSRAGSWTGFVFSGVSWRRMLSLVTFSLAINDKE
VTYQYETLKAGTVTMLVINPSGFVQRLLWSERTGNWEILSTRPMDQCEYYAFCDVNSLCN
VTNSPKTCTCLEGFVPKFYEKWSALDWSGGCVRRINLSCEGDVFQKYAGMKLPDTSSSWY
DKSLNLEKCEKLCLKNCSCTAYANVDVDGRGCLLWFDNIVDLTRHTDQGQDIYIRLAASE
LDHRGNDQSFDNKKLVGIVVGIVAFIMVLGSVTFTYMKRKKLAKREMLKIFHWKYKREKE
DVELSTIFDFSTISNATDQFSPSKKLGEGGFGPVYKGLLKDGQEIAVKRLAKTSEQGAEQ
FKNEVMLMAKLQHRNLVKLLGCSIHQKERLLIYEYMSNRSLDYFIFDSTQSKQLDLTKRL
QIIDGIARGLLYLHQDSRLRIIHRDLKVSNILLDNDMNPKISDFGLARTFGGDQAEANTN
RVMGTYGYMPPEYALHGRFSIKSDVFSFGVIVLEIISGRKNRNFQDSEHHLNLLSHAWRL
WIEEKPLELIDDLLDDPVSPHEILRCIHVGLLCVQQTPENRPNMSSVVLMLNGEKLLPDP
SQPGFYTGTIQYPIQLESSSRSVGACSQNEATVSLLEAR*

>Glyma.11G206200|Glyma.11G206200.1|29046880|GNA
MGFFSPGNSTRRYLAIWYTNASSYTVVWVANRNTPLQNNSGVLKLNEKGIRELLSATNGA
IWSSNISSKAVNNPVAYLLDLGNFVVKSGHDTNKNSFLWQSFDYPTDTLMSGMKLEWNIE
TGLERSLTSWKSVEDPAEGEYASKIELRGYPQLVRFKGPDIKTRIGSWNGLYLVYN*

>Glyma.11G219800|Glyma.11G219800.1|31465079|GNA/PAN/protein kinase
MTLSKRMVSFITFTCFLHLTKPSNLREDTLLQGHQLGSTNRLISPSGLYTLRFFQLDDGS
DANSKFYLGVSANKFHYYVWVANRDNPIHDDPGVLTIDEFSNLKILSSTTTMMLYSVEAE
NTNKSVRATLLDTGNFVLHELNPDGISVKRVLWQSFDYPTDTILPGMKLGYDKNTGHTWS
ITARRSYRTLWSGSFSLSLDPKTNQLVSRWREAIIWSSGEWRNGSFSNLNSSSLYKENFN
FTFFSNESVTYFEYASVSGYFTMEPLGRLNASGAAYSCVDIEIVPGCTMPRPPKCREDDD
LYLPNWNSLGAMSRRGFIFDERENLTISDCWMKCLKNCSCVAYTYAKEDATGCEIWSRDD
TSYFVETNSGVGRPIFFFQTETKAIEKRKKRASLFYDTEISVAYDEGREQWNEKRTGNDA
HIFDLITILEATDNFSFTNKIGEGGFGPVYKGKLSNGQEIAIKRLSKSSGQGLVEFKNEA
MLIVKLQHTNLVRLLGFCSDREERILVYEYMSNKSLNLYLFDSTKRNVLEWKTRYRIIQG
VAQGLVYLHQYSRLKVIHRDLKASNILLDNELNPKISDFGMARIFKLTQSEEKTNRVVGT
YGYMSPEYAMSGVISTKTDVYSFGVLLLEIVSGKKNNCDDYPLNLIGYAWKLWNQGEALK
LVDTMLNGSCPHIQVIRCIHIGLLCTQDQAKDRPTMLDVISFLSNENTQLPPPIQPSLYT
INGVKEAKQHKSCSINEITNSMTSGR*

>Glyma.12G103700|Glyma.12G103700.1|9277653|GNA/S-locus glycoprotein/PAN/protein kinase/SRK
MVNTKGETRRIIILNWCSSHMLSIFILYSFFFTFSFKHCSATDTISITINNFLQDGGGDT
LVSKGENFELGFFTPNGSSSGKRYLGIWYYKLTPLTVVWVANRDKPLLDSCGAFGIAEDG
NLKVLDKSGKFYWGTNLEGSHSQHRIVMLMDNGNLVVSDEVEDQGNHQVKILWQSFANPT
DTFLPGMKMDDNLALTSWRSYEDPAPGNFSFEHDQGENQYIIWKRSIRYWKSSVSGKFVG
TGEISTAISYFLSNFTLKVSPNNTVPFLTSALYTDTRLVMTHWGQLKYMKMDSEKMWLLV

WGEPRDRCSVFNACGNFGSCNSKYDSMCKCLPGFKPNSIESWNAGDFSGGCSRKTNVCSG
DAKGDTFLSLKMMKVGNPDAQFNAKDEEECMSECLNNCQCYAYSYEDTEKGRLGDSGDVV
CWIWSEDLNNLEEEYEDGCDLHVRVAVSDIESTGRNCGTCGTNFIPYPLSTGPSCGDPMY
FSFHCNISTGELDFETPGGTYQVISINPEAQKFLIHRKNVLNCDQSSRDKFLPLNKSFPF
HLTSNCYADPSIFSSNAPMKHGVEIELSWEQPLEPICSSLLDCKEWPNSTCNTSSDGKKR
CLCNTNFLWDGLKLNCTLEGNHSYQPERQLSLPKIIVITLTTVIGLILLSTTSTCVYLRK
RRQAKPQDSRGYVQKNSGINLYDSERYVRDLIESSRFKEDDAQAIDIPYFHLESILDATN
NFANTNKLGQGGFGPVYKGKFPGGQEIAVKRLSSCSGQGLEEFKNEVVLIAKLQHRNLVR
LLGYCVEGDEKMLVYEYMPNRSLDAFIFDRKLCVLLDWDVRFKIILGIARGLLYLHEDSR
LRIIHRDLKTSNILLDEEKNPKISDFGLARIFGGKETVANTERVVGTYGYMSPEYALDGH
FSVKSDVFSFGVVVLEIISGKRNTGFYQADHELSLLGYAWLLWKEGKALEFMDQTLCQTC
NADECLKCVIVGLLCLQEDPNERPTMSNVVFMLGSEFNTLPSPKEPAFVIRRCPSSRAST
SSKLETFSRNELTVTIEHGR*

>Glyma.12G104100|Glyma.12G104100.1|9363764|GNA/S-locus glycoprotein/PAN/protein kinase
MRNNKPQLWLSLSLIITCFSFHTSLAALTTISANQSLSGDETLVSQHGNFELGFFNTGNN
SNKFYIGMWYKKISQRTYVWVANRDQPVSDKNSAKLTILEGNLVLLDQSQNLVWSTNLSS
PSSGSAVAVLLDTGNLILSNRANASVSDAMWQSFDHPTDTWLPGGKIKLDKKTKKPQYLT
SWKNREDPAPGLFSLELDPAGSNAYLILWNKSEQYWTSGAWNGQIFSLVPEMRLNYIYNF
TFQSNENESYFTYSMYNSSIISRFVMDGSGQIKQLSWLENAQQWNLFWSQPRQQCEVYAF
CGGFGSCTENAMPYCNCLNGYEPKSQSDWNLTDYSGGCVKKTKFQCENPNSSDKEKDRFL
PILNMKLPNHSQSIGAGTVGECEAKCLSNCSCTAYAHDNSGCSIWHGDLLNLQQLTQDDN
SGQTLFLRLAASEFDDSNSNKGTVIGAVAGAVGGVVVLLILFVFVMLRRRKRHVGTRTSV
EGSLMAFGYRDLQNATKNFSEKLGGGGFGSVFKGTLPDSSVVAVKKLESISQGEKQFRTE
VSTIGTVQHVNLVRLRGFCSEGTKKLLVYDYMPNGSLESKIFHEDSSKVLLDWKVRYQIA
LGTARGLTYLHEKCRDCIIHCDVKPENILLDADFIPKVADFGLAKLVGRDFSRVLTTMRG
TRGYLAPEWISGVAITAKADVYSYGMMLFEFVSGRRNSEASEDGQVRFFPTIAANMMHQG
GNVLSLLDPRLEENADIEEVTRVIKVASWCVQDDESHRPSMGQVVQILEGFLDVTLPPIP
RTLQAFVDNHENVVFFTDSSSTQTSQVKSNASAASSQAKSNISSSNSST*
>Glyma.12G140200|Glyma.12G140200.1|17278442|GNA/S-locus glycoprotein/PAN/protein kinase
MSIIVYTLFVSSLVVSIAADTPSNSQFQSLSPGETIVSPRGIFELGFFNLGNPNKSYLAI
RYKSYPDQTFVWVANGANPINDSSAILKLNSPGSLVLTHYNNHVWSTSSPKEAMNPVAEL
LDSGNLVIREKNEAKLEGKEYLWQSFDYPSNTMLAGMKIGWDLKRKINRRLIAWKSDDDP
TPGDLSWIIVLHPYPEIYMMSGTKKHHRLGPWNGLRFSGMPEMKPNPVFNYKFVSNKDEV
TYMWTLQTSLITKVVLNQTSQQRPRYVWSEATRSWNFYSTMPGEYCDYYGVCGANSFCSS
TASPMCDCLKGFKPKSPEKWNSMYRTEGCRLKSPLTCMLDGFVHVDGLKVPDTTNTSVDE
SIDLEKCRTKCLNNCSCMAYTNSNISGSGSGCVMWFGDLLDIKLYPAPESGQRLYIRLPP
SELDSIRHKVSKIMYATSVAAAIGVILAIYFLYRRKIYEKSMAEYNNESYVNDLDLPLLD
LSIIIVATNKFSEGNKIGEGGFGSVYWGKLASGLEIAVKRLSKNSDQGMSEFVNEVKLIA
RVQHRNLVKLLGCCIQKKEKMLVYEYMVNGSLDYFIFDSTKGKLLDWPKRFHIICGIARG
LMYLHQDSRLRIVHRDLKASNVLLDDTLNPKISDFGVAKTFGEENIEGNTNRIVGTYGYM
APEYAIDGQFSIKSDVFSFGVLLLEIICGKKSRCSSGKQIVHLVDHVWTLWKKDMALQIV
DPNMEDSCIASEVLRCIHIGLLCVQQYPEDRPTMTSVVLLLGSDEVQLDEPKEPGHFVKK
ESIEANSSSCSSTNAMSITLLTAR*

>Glyma.12G140300|Glyma.12G140300.1|17324801|GNA/S-locus glycoprotein/PAN/protein kinase
MEIFSFLIFIVSYMLVPSLKISAATLSVSQYVTDGETLVSNSGVFELGFFSPGKSTKRYL
GIWYKNITSDRAVWVANRENPINDSSGILTFSTTGNLELRQNDSVVWSTNYKKQAQNPVA
ELLDTGNFVVRNEGDTDPETYSWQSFDYPSDTLLPGMKLGWDLRTGLERKLTSWKSPDDP
SAGDFSWGLMLHNYPEFYLMIGTHKYYRTGPWNGLHFSGSSNRTLNPLYEFKYVTTNDLI
YASNKVEMFYSFSLIKNSSIVMIVNINETMSDIRTQVWSEVRQKLLIYETTPRDYCDVYA
VCGAYANCRITDAPACNCLEGFKPKSPQEWSSMDWSQGCVRPKPLSCQEIDYMDHFVKYV
GLKVPDTTYTWLDENINLEECRLKCLNNCSCMAFANSDIRGGGSGCVLWFGDLIDIRQYP
TGEQDLYIRMPAKESINQEEHGHNSVKIIIATTIAGISGILSFCIFVIYRVRRSIADNFK
TKENIERQLKDLDLPLFDLLTITTATYNFSSNSKIGHGGFGPVYKGKLADGQQIAVKRLS
SSSGQGITEFVTEVKLIAKLQHRNLVKLLGFCIKRQEKILVYEYMVNGSLDSFIFDKIKG
KFLDWPRRFHIIFGIARGLLYLHQDSRLRIIHRDLKASNVLLDEKLNPKISDFGMARAFG
GDQTEGNTNRVVGTYGYMAPEYAVDGLFSIKSDVFSFGILLLEIICGNKNRALCHGNQTL
NLVGYAWTLWKEQNVLQLIDSSIKDSCVIPEVLRCIHVSLLCVQQYPEDRPSMTLVIQML
GSETDLIEPKEPGFFPRRFSDEGNLSTIPNHMSSNEELTITALNGR*

>Glyma.12G140500|Glyma.12G140500.1|17341802|GNA/S-locus glycoprotein
MIEEHGERTLCNHPIVLKDDVEKFALDWNYRSNEPENFDKSTSSDITWSISDGKTLESTT
GVIELGFFIPGNSNKRYLGIWYKNIPTDRVVWVANGANPINDSSAIKKQAQNPVAELLDN
GNFVVRNEEETDPDAYVWQSFDYPSDTLLTGMKLGWDLRTGMEQKITSWRSPDNPSPGDF
SWDLMLYNYPEFYLMNGTQKFYRIGPWNELHFSGVSNQNSNWLYELKHVSNKDDMFYSYT
LKNNSEPITDSRVCQCLKGFNPKSPQESSQGCVRNKPLTCKDNYKDGLCRPENTRYYTNL

AG*

>Glyma.12G140800|Glyma.12G140800.1|17522247|GNA/S-locus glycoprotein/PAN/protein kinase
MICKQVGHRTQTLRLKFMSLILIYNIRNPFGYSHKRYVGIWYKNIPIQTVVWVANKANPI
NDSSGIITLNNTGNLVLTQNAYLVWYTNNSHKQAQNPVVVLLDSGNLVIKNEEETDPEVC
LWQSFDYPSDTLLPGMKLERNIRTGHEWKLTSWKNPNDPSPGDIYRVLELYNYPELYVMK
GKKKVYRSGPWNGLYFSGLPYLQNNTIFGYNFVSNKDEIYFTFNLLNNCIVYRYVWLEGD
HNWTMHRSYPKEFCDNYGLCGAYGNCIINQAQGCQCLKGFSPKSPQAWASSDWSQGCVRN
KPLSCNGEHKDGFVKFEGLKVPDTTQTWLDKTIGLEECRVKCLNNCSCMAYSNSDIRGAG
SGCVMWYGDLIDIRQFETGGQGLHIRMSASESVTNYSKDKSEKDIDLPTFDFSFISNATN
DFSQSEKLGQGGFGSVYKGILPDGQEIAVKRLSKTSGQGLDEFKNEVMLIAKLQHRNLVK
LLGCSIQQDEKLLIYEFMPNRSLDYFIFDSTRHTLLGWTKRFEIIGGIARGLLYLHQDSR
LKIIHRDLKTSNVLLDSNMNPKISDFGMARTFGLDQDEANTNRVMGTYGYMPPEYVVHGS
FSVKSDVFSFGVIVLEIISGKKNRAFYDPHHHLNLLGHAWRLWIEKRPTELMDDLVDNSA
CPSEIIRYIHIGLLCVQQRPEDRPNMSSVTLFLNGEKLLPEPNQPGFYTGKAHPTKPNSS
SRNIDVYSFNEMSNSLLEPR*

>Glyma.12G142100|Glyma.12G142100.1|18033116|GNA/S-locus glycoprotein/PAN/protein kinase
MDILSSLIFVASILIPCFKFCIAADTILLSQSISDGMTLVSRGETFELGFFSPENSNKRY
LGIWYKNIPQTVVWVSNRAINDSSGILTVNSTGNLVLRQHDKVVWYTTSEKQAQNPVAQL
LDSGNLVVRDEGEADSEGYLWQSFDYPSDTILPGMKLGLNLRTGIEWRMTSWKNPNDPSP
GDFYWGLLLYNYPEFYLMMGTEKFVRVGPWNGLHFSGIPDQKPNPIYAFNYISNKDEKYY
TYSLQNAAVISRLVMNQTSSMSIRYVWMENEQYWKVYKSLPKDNCDYYGTCGAYGTCLIT
GSQICQCLAGFSPKSPQAWNSSDWTQGCTRNQPLNCTNKLNDGFMKVEGVKVPDTTHTWL
DETIGLGECRMKCLNNCSCMAYTNSDIRGEGSGCVMWFGDLIDIRQFENDGQDLYIRMDS
SELGEQEEHKKVVIIIVSTTIATILVILCIGCYRIYIVRHSITEYSDIVRDQNRGGSEEN
IDLPLLDLSTIVIATDNFSINNKIGEGGFGPVYKGRLVSGQEIAVKRLSRGSGQGMTEFK
NEVKLIAKLQHRNLVKLLGCCVQEQDRMLVYEYMTNRSLDWLIFDDTKSKLLDWPKRFNI
ICGIARGLLYLHQDSRLRIIHRDLKASNVLLDDQMIPKISDFGIARIFGGEQTEGNTNRV
VGTYGYMAPEYAADGIFSVKTDVFSFGILLLEILSGKRNRGFYLENQSANLVTHAWNLWK
GGRAIEMVDSNIEDSCVLSEVLRCIHVCLLCVQQHAEDRPLMPSVVLMLGSESELAEPKE
PGFYIKNDEGEKISISGQSDLFSTNEITITLLEAR*

>Glyma.12G142200|Glyma.12G142200.1|18094703|GNA/S-locus glycoprotein/PAN/protein kinase/SRK
MAMLTILLLSKLLSLFSKFAVATDTITQSEFLEDNTTLVSNNGTFELGFFTPGSSSSPNR
YVGIWYKNIPIRTLVWVANRDNPIKDNSSKLSINTQGNLVLVNQNNTVIWSTNTTAKASL
VVAQLLDSGNLVLRDEKDTNPENYLWQSFDYPSDTFLPGMKLGWDLKKGLNWFLTAWKNW
DDPSPGDFTRSTLHTNNPEEVMWKGTTQYYRSGPWDGIGFSGIPSVSSDSNTNYTIVSNK
DEFYITYSLIDKSLISRVVMNQTRYARQRLAWNIDSQTWRVSSELPTDFCDQYNICGAFG
ICVIGQAPACKCLDGFKPKSPRNWTQMSWNQGCVHNQTWSCRKKGRDGFNKFSNVKVPDT
RRSWVNANMTLDECKNKCWENCSCTAYANSDIKGGGSGCAIWFSDLLDIRLMPNAGQDLY
IRLAMSETAQQYQEAKHSSKKKVVVIASTVSSVIAILLIFIFIYWSYKNKNKEIITGIEG
KNNKSQQEDFELPLFDLASIAHATNNFSNDNKLGEGGFGPVYKGILPYGQEVAVKRLSET
SRQGLKEFKNEVMLCAELQHRNLVKVLGCCIQDDEKLLIYEYMANKSLDVFLFDSSQGKL
LDWPKRFCIINGIARGLLYLHQDSRLRIIHRDLKASNVLLDNEMNPKISDFGLARMCGGD
QIEGKTSRVVGTYGYMAPEYAFDGIFSIKSDVFSFGVLLLEIVSGKKNRLFSPNDYNNLI
GHAWRLSKEGKPMQFIDTSLKDSYNLHEALRCIHIGLLCVQHHPNDRPNMASVVVSLSNE
NALPLPKNPSYLLNDIPTERESSSNTSLSVNDVTTSMLSGR*

>Glyma.12G142300|Glyma.12G142300.1|18108745|GNA/S-locus glycoprotein/PAN/protein kinase/SRK
MAMLTILLLSKLLSLFSKFAVATDTITQSEFLEDNTTLVSNNGTFELGFFTPGSSSSPNL
YVGIWYKNIPIRTVVWVANRDNPIKDNSSKLSINTKGYLVLINQNNTVIWSTNTTTKASL
VVAQLLDSGNLVLRDEKDTNPENYLWQSFDYPSDTFLPGMKLGWDLKKGLNRVLTAWKNW
DDPSPGDFTLSILHTNNPEVVMWKGTTQYYGSGPWDGTVFSGSPSVSSDSNVNYAIVSNK
DEFYITYSLIDKSLISRVVINQTKYVRQRLLWNIDSQMWRVSSELPTDFCDQYNTCGAFG
ICVIGQVPACKCLDGFKPKSPRNWTQMSWNQGCVHNQTWSCRKKGRDGFNKFNSVKAPDT
RRSWVNASMTLDECKNKCWENCSCTAYANSDIKGGGSGCAIWFSDLLNIRLMPNAGQDLY
IRLAVSETAQQNQDEKHSSKKKVVVIASTVSSVIAILLIFIFIYWSYKNKNKEIITGIEG
KNNKSQQEDFELPLFDLASIAHATNNFSHDNKLGEGGFGPVYKGILPDGQEVAVKRLSRT
SRQGLKEFKNEVMLCAELQHRNLVKVLGCCIQDDEKLLIYEYMANKSLDVFLFDSSQGKL
LDWPKRFCIINGIARGLLYLHQDSRLRIIHRDLKASNVLLDNEMNPKISDFGLARMCGGD
QIEGKTNRVVGTYGYMAPEYAFDGIFSIKSDVFSFGVLLLEIVSGKKNRLFYPNDYNNLI
GHAWRLWKEGNPMQFIDTSLKDSYNLHEALRCIHIGLLCVQHHPNDRSNMASVVVSLSNE
NALPLPKNPSYLLNDIPTERESSSNTSFSVNDVTTSMLSGR*

>Glyma.12G142400|Glyma.12G142400.1|18209733|GNA/S-locus glycoprotein/PAN/protein kinase
MAMLTIFLLVSKLIFFFSKFAAATDTINQFESLEDNTTLVSNDGTFELGFFIPGSTSPNR
YLGIWYKNIPIRTVVWVANRETPIKDNSSKLNITPEGSLVLLNQNKTVIWSANPTTKGVV
VVAQLLDSGNLVLRDEKDTNPENYLWQSFDNPTDTFLPGMKLGWDLKKGLNTVLTAWKNW
DDPSPGDFTDITLRTNYPEEVMWKGTTKYWRSGPWDGTKFSGNPSVPSNAIVNYTIVSNK
DEFYATYSMTDKSIISRIVMNQSLYVRQRLTWNTDSQTWRVSSELPGDLCDHYNTCGAFG
ICVAGQAPVCKCLDGFKPKSPRNWNQMNWNQGCVHNQTWSCREKNKDGFTKFSNVKAPDT
ERSWVNASMTLGECRVKCWENCSCMAYANSNIRGEGSGCAIWIGDLLDIRLMPNAGQDLY
IRLAVSETAQQSHDQKDNSNKKVVVIASTISSVIAMILIFIFIYWSYRNKNKEIITGIEG
KSNESQQEDFELPLFDLVLIAQATDHFSDHKKLGEGGFGPVYKGTLPDGQEVAVKRLSQT
SRQGLKEFKNEVMLCAELQHRNLVKVLGCCFQDDEKLLIYEYMSNKSLDVFLFDSSRSKL
LDWPKRFCIINGIARGLLYLHQDSRLRIIHRDLKASNVLLDNEMNPKISDFGLARMCGGD
QIEGETSRIVGTYGYMAPEYAFDGLFSIKSDVFSFGVLLLEIVSGKKNSRLFYPNDYNNL
IGHAWRLWKEGNPMQFIDSSLEDSCILYEALRCIHIGLLCVQHHPNDRPNMASVVVLLSN
ENALPLPKDPSYLSKDISTERESSSENFTSVSINDVTISMLSDR*

>Glyma.12G142800|Glyma.12G142800.1|18489474|GNA/S-locus glycoprotein/PAN/protein kinase
MLCIWFFLLLGTSTSLDSLAVGQSLRDVENESLVSAGGITELGFFSLGDFSRRYLGVWFR
NINPSTKVWVANRNTPLKKNSGVLKLNERGVLELLNDKNSTIWSSNISSIALNNPIAHLL
DSGNFVVKYGQETNDDSLLWQSFDYPGNILLPGMKLGWNLETGLERFLSSWTSSNDPAEG
DYAAKIDLRGYPQIIKFQRSIVVSRGGSWNGMSTFGNPGPTSEASQKLVLNEKEVYYEYE
LLDRSVFTILKLTHSGNSMTLVWTTQSSTQQVVSTGEIDPCENYAFCGVNSICNYDGNVT
ICKCSRGYVPSSPDRWNIGVSSDGCVPKNKSNDSNSYGDSFFKYTNLKLPDTKTSWFNKT
MDLDECQKSCLKNRSCTAYANLDIRDGGSGCLLWFHGLFDMRKYSQGGQDLYVRVPASEL
DHVGHGNMKKKIVGIIVGVTTFGLIITCVCILVIKNPGSARKFYSNNYKNIQRKEDVDLP
VFSLSVLANVTENFSTKNKLGEGGFGPVYKGTMIDGKVLAVKRLSKKSGQGLEEFKNEVT
LISKLQHRNLVKLLGCCIEGEEKMLIYEYMPNHSLDYFVFDETKRKLLDWHKRFNVITGI
ARGLLYLHQDSRLRIIHRDLKTSNILLDANLDPKISDFGLARSFLGDQVEANTNRVAGTY
GYMPPEYAARGHFSVKSDVFSYGVIVLEIVSGKKNRDFSDPEHYNNLLGHAWRLWTEERA
LELLDKLSGECSPSEVVRCIQVGLLCVQQRPQDRPHMSSVVLMLNGDKLLPKPKVPGFYT
GTDVTSEALGNHRLCSVNELSITMLDAR*

>Glyma.12G143000|Glyma.12G143000.1|18512606|GNA/S-locus glycoprotein/PAN/protein kinase
MKENVPKTMENFDILGVCLLFLSLITMSSTLDMVTTIQPIRDGKNENETLVSTNGTFEAG
FFSPENFDSRYLGIWYTNIFPRTVVWVANKEKPLKDHSGVLEVDTDQGILSIKDGTGAKI
WFSSASHTPNKPVAAELLESGNMVLKDGDNNFLWQSFDYPGDTLLPGMKIGVNFKTGQHR
ALRSWRSFTDPTPGNFSLGVDTRGLPQLVITNENTNSNDIAYRPGSWNGLSITGLPGEIT
DQLTKSLFVMNQDEVFYEIQLLNSSTKLMRSRLLPEGYQVRFIWSDEKKIWDSQFPKPFD
VCQTYALCGANAICDFNGKAKHCGCLSGFKANSAGSICARTTRLDCNKGGIDKFQKYKGM
KLPDTSSSWYDRTITLLECEKLCLSNCSCTAYAQLNISGEGSGCLHWFSDIVDIRTLPE
GGQNFYLRMATVTASELQLQDHRFSRKKLAGIVVGCTIFIIAVTVFGLIFCIRRKKLKQS
EANYWKDKSKEDDIDLPIFHFLSISNATNQFSESNKLGQGGFGPVYKGILPDGQEIAVKR
LSKTSGQGLDEFKNEVMLVAKLQHRNLVKLLGCSIQQDEKLLVYEFMPNRSLDYFIFDST
RRTLLGWAKRFEIIGGIARGLLYLHQDSRLKIIHRDLKTGNVLLDSNMNPKISDFGMART
FGLDQDEANTNRVMGTYGYMPPEYAVHGSFSVKSDVFSFGVIVLEIISGRKNRGFCDPHN
HLNLLGHAWRLWIEKRPLELMDDSADNLVAPSEILRYIHIGLLCVQQRPEDRPNMSSVVL
MLNGEKLLPEPSQPGFYTGGRDHSTVTNSSSRNCEAYSLNEMSDSLLKPR*

>Glyma.12G143200|Glyma.12G143200.1|18578467|GNA/S-locus glycoprotein/PAN/protein kinase
MLLIWFFLFSYMTRTSTSVDHLAASRSIRDSQILVSAGNITALGFFSPGNSTRRYLGIWF
RKVHPFTVVWVANRNTPLENESGVLKLNKRGILELLNGKNSTIWSSSSNKSSKAAKKPIA
QLRDLGNLVVINGPKRNTKKHKTNNGDILWQSFDYPGDTLMPGMKLGWTLENGLERSLSS
WKNWSDPAEGEYTLKVDRRGYPQIILFRGPDIKRRLGSWNGLPIVGYPTSTHLVSQKFVF
HEKEVYYEYKVKEKVNRSVFNLYNLNSFGTVRDLFWSTQNRNRRGFQILEQNQCEDYAFC
GVNSICNYIGKKATCKCVKGYSPKSPSWNSSTWSRGCVPPIPMNKSNCKNSYTEEFWKNQ
HMKFPDTSSSLFIETMDYTACKIRCRDNCSCVAYANISTGGGTGCLLWFNELVDLSSNGG
QDLYTKIPAPVPPNNNTIVHPASDPADHRNLKIKTVAITVGVTTFGLIIIYVWIWIIKNP
GAARKFYKQNFRKVKRMKEIDLPTFDLSVLANATENFSSKHKLGEGGFGPVYKIIFQGTL
IDGKVIAVKRLSKKSKQGLDELKNEVALIAKLQHRNLVKLLGCCIEGEEKMLIYEYMPNL
SLDCFLFDETKKKLLDWPKRFNIISGITRGLVYLHQDSRLRIIHRDLKTSNILLDDNLDP
KISDFGLARSFLEDQVEANTNRVAGTCGYMPPEYAAGGRFSVKSDVFSYGVIVLEIVSGK
RNTEFANSENYNNILGHAWTLWTEDRALELLDDVVGEQCKPYEVIRCIQVGLLCVQQRPQ
DRPHMSSVLSMLSGDKLLPKPMAPGFYSGTNVTSEATSSSANHKLWSVNEASITELDAR*

>Glyma.12G143900|Glyma.12G143900.1|18958348|GNA/S-locus glycoprotein/PAN/protein kinase

MMHSTLMLCIWFLLLSYLSETCTSLHSLEVNQSIRDGETLVSARGITEVGFFSPGNSTRR
YLGIWYTNVSPFTVVWVANRNTPLENKSGVLKLNEKGVLMIFDAANSTIWSSSIPSKARN
NPIAHLLDSANFVVKNGRETNSVLWQSFDYPSDTLIPGMKIGGNLETGEERLITSWKSAD
DPAVGEYTTKIDLRGYPQYVVLKGSEIMVRAGPWNGESWVGYPLQTPNTSQTFWFNGKEG
YSEIQLLDRSVFSIYTLTPSGTTRNLFWTTQTRTRPVLSSGEVDQCGKYAMCGTNSICNF
DGNYATCECLKGYVPKSPDQWNIASWSDGCVPRNKSNCENSYTDGFFKYTHLKIPDTSSS
WFSKTMNLDECRKSCLENCFCTAYANLDIRDGGSGCLLWFNTLVDMMQFSQWGQDLYIRV
PASELDHVGHGNKKKIAGITVGVTIVGLIITSICILMIKNPRVARKFSNKHYKNKQGIED
IELPTFDLSVLANATENYSTKNKLGEGGFGPVYKGTLKDGQELAVKRLSNNSGQGLEEFK
NEVALIAKLQHRNLVKLLGCCIEREEKMLVYEYMSNKSLNYFVFDETKGKLLDWCKRFNI
ICGIARGLLYLHQDSRLRIIHRDLKTSNILVDSNWDPKISDFGLARSFLEDQFEAKTNRV
VGTYGYMPPEYAVRGNFSVKSDVFSFGVIILEIVSGKKNREFSDPEHCHNLLGHAWRLWV
EERALDLLDKVLEEQCRPFEVIRCIQVGLLCVQRRPEHRPDMSSVVPMLNGEKLLPEPTV
PAFYNETIITEVNCKLCSSNELSITQLDAR*

>Glyma.12G144100|Glyma.12G144100.1|18975569|GNA/S-locus glycoprotein/PAN/protein
kinase
MLCIWFFLFSYFSGTCTSLHSLAVNQSIRDAENETLVSAGGIIEVGFFSPGKSTRRYLGI
WFKNVNPLKVVWVANRNAPLEKNSGVLKLDEKGILVLLNHKNSTIWSSNISSKAGNNPIA
HPLDSGNFVVKNGQQPGKDAILWQSFDYPGDTHTPGMKFGWSFGLERSISSWKSVDDPAE
GEYVVKMDLRGYPQVIMFKGSKIKVRVGPWNGLSLVGYPVEIPYCSQKFVYNEKEVYYEY
NLLHSLDFSLLKLSPSGRAQRMYWRTQTSTRQVLTIEEIDQCEYYDFCGENSICNYDGNR
PTCECLRGYVPKSPDQWNMPIFQSGCAPRNKSDCKNSYTDGFLKYARMKLPDTSSSWFSK
TMNLNECQKSCLKNCSCTAYANLDIRNGGSGCLLWFNNIVDMRYFSKSGQDIYIRVPASE
LDHAGPGNIKKKILGIAVGVTIFGLIITCVCILISKNPMARRLYCHIPRFQWRQEYLILR
KEDMDLSTFELSTIAKATNNFSIRNKLGEGGFGPVYKGTLIDGQEVAIKRHSQMSDQGPG
EFKNEVVLIAKLQHRNLVKLLGCCVQGGEKLLIYEYMPNKSLDYFIFDKARSKILAWNQR
FHIIGGIARGLLYLHQDSRLRIIHRDLKTSNILLDANMNPKISDFGLARTFGCEQIQAKT
RKVVGTYGYMPPEYAVHGHYSVKSDVFGFGVIVLEIVSGSKNRGFSDPEHSLNLLGHAWR
LWTEDRPLELIDINLHERCIPFEVLRCIHVGLLCVQQKPGDRPDMSSVIPMLNGEKLLPQ
PKAPGFYTGKCIPEFSSPKTCKFLSQNEISLTIFEAR*

>Glyma.12G144400|Glyma.12G144400.1|19069606|GNA/S-locus glycoprotein/PAN/protein
kinase/SRK
MLFIWFFLFSYFSGTCTSLHSLAVNQSIRDGENETLVSAGGIIEVGFFSPGKSTRRYLGI
WFKNVNPLTVVWVANRNAPLEKNSGVLKLDEKGILVILNHKNSTIWSSNISSKAGNNPIA
HPLDSGNFVVKNGQQPGKDAILWQSFDYPGDTHTPGIKFGWNFQIGLERSLSSWKSVDDP
AEGEYVAKMDLRGYPQVIVFKGSEIKVRVGPWNGLSLVGYPVEIPYCSQKFVLNEKEVYY
EYNLLDSLDFSLFKLSPSGRSQRMYWRTQTNTRQVLTVEERDQCENYGFCGENSICNYDG
SRATCECLRGYVPKSPDQWNMPIFQSGCVPGNKSDCKNSYSDGFLKYARMKLPDTSSSWF
SKTMNLDECQKSCLKNCSCTAYANLDIRNGGSGCLLWFNNIVDMRCFSKSGQDVYIRVPA
SELDHGGPGNIKKKILGIAVGVTIFGLIITCVCILISKNPIARRLYRHFRQFQWRQEYLI
LRKEDMDLSTFELSTIAEATNNFSSRNKLGEGGFGPVYKGTLIDGQDVAIKRHSQMSDQG
LGEFKNEVVLIAKLQHRNLVKLLGCCVQGGEKLLIYEYMSNKSLDYFIFDEARSKLLAWN
QRFHIIGGIARGLLYLHQDSRLRIIHRDLKTSNILLDADMNPKISDFGLAQSFGCDQIQA
KTRKVVGTYGYMPPEYAVHGHYSVKSDVFGFGVIVLEIVSGSKNRGFSDPKHSLNLLGHA
WRLWTEDRPLELIDINLHERCIPFEVLRCIHLGLLCVQQKPGDRPDMSSVIPMLNGEKLL
PQPKAPGFYTGKCTPESVSSSKTCKFLSQNEISLTIFEAR*

>Glyma.12G144500|Glyma.12G144500.1|19113078|GNA/S-locus glycoprotein/PAN/protein
kinase
MVHIFRMLFIWFLLLSYLRNSTSSDNLAVSQYIRDGETLVSEEGTFEVGFFSPGASTGRY
LGIWYRNLSPLTVVWVANRENALQNKSGVLKLDEKGVLVILNGTNNTIWWSNNTSSKAAK
NPIAQILDSGNIVVRNERDINEDNFFWQSFDYPCDTFLPGMKIGWKTGLDRTLSSWKNED
DPAKGEYSMKLDLRGYPQFFGYKGDVITFRGGSWNGQALVGYPIRPPTQQYVYDFVFNEK
EVYVEYKTPDRSIFIIITLTPSGSGFGNVLLWTKQTRNIEVLRLGESDQCENYAICGANS
ICNMDGNSQTCDCIKGYVPKFPEQRNVSYLHNGCVPRNKFDCKSSNTNGFLRYTDLKLPD
TSSSWLNKTMNLDECQKSCLKNCSCKAYANADIRNGGSGCLLWFDDLIDMRKFSLGGQDI
YFRVPASELDHVAFNGHGKNMKKMLGITVGTIILGLTACACIIMILKMQGRIIYRKHFKH
KLRKEGIDLSTFDFLIIARATENFAESNKLGEGGFGPVYKGRLKNGQEFAVKRLSKKSGQ
GLEEFKNEVVLIAKLQHRNLVKLIGCCIEGNERMLIYEYMPNKSLDNFIFHETQRNLVDW
PKRFNIICGIARGLLYLHQDSRLRIVHRDLKTSNILLDANLDPKISDFGLARTLWGDQVE
ANTNRVAGTYGYMPPEYAARGHFSMKSDVFSYGVILLEIVSGQRNREFSDPKHNLNLLGY
AWRLWTEERALELLEGVLRERLTPSEVIRCIQVGLLCVQQRPEDRPDMSSVVLMLNGEKL
LPNPNVPGFYTERAVTPESDIKPSSNQLSITLLEAR*

>Glyma.12G144800|Glyma.12G144800.1|19170258|GNA/S-locus glycoprotein/PAN/protein
kinase
MVDNFRMLFIWLFILLSYLKNSTSMDSLSPSQSIRDGETLVSDEETFEVGFFSPGTSTRR
YLGIWYRNVSPLTVVWVANRENALQNKLGVMKLDENGVIVILSGNNSKIWWSSSTSSKVV

KNPIAQLLDYGNLVVRDERDINEDKFLWQSFDNPCDKFLPGMKIGWNLVTGLDRIISSWK
NEDDPAKGEYSFKLDLKGYPQLFGYKGNVIRFRVGSWNGQALVGYPIRPVTQYVHELVFN
EKEVYYEYKILDRSIFFIVTLNSSGIGNVLLWTNQTRRIKVISLRSDLCENYAMCGINST
CSMDGNSQTCDCIKGYVPKFPEQWNVSKWYNGCVPRNKPDCTNINIDGLLRYTDLKLPDT
SSSWFNTTMSLEECKKSCLKNFSCKAYANLDIRNGGSGCLLWFDDLIDTRKFSIGGQDIY
FRIQASSLLDHVAVNGHGKNTRRMIGITVGANILGLTAWAAKIIYRNHFKRKLRKEGIGL
STFDFPIIARATENIAESNKLGEGGFGPVYKGRLKDGLEFAVKKLSKNSAQGLEELKNEV
VLIAKLQHRNLVKLIGCCIEGNERMLIYEYMPNKSLDCFIFDETRRHLVDWPIRFNIICG
IARGLLYLHQDSRLRIVHRDLKTCNILLDASLDPKISDFGLARTLCGDQVEANTNKVAGT
YGYMPPVYVTRGHFSMKSDVFSYGVVVLEIVSGKRNREFSDPKHFLNLVGHAWRLWTEER
ALELLDGVLRERFTPSEVIRCIQVGLLCVQQRPKDRPDMSSVVLMLNGEKLLPNPKVPGF
YTEGDVTPESDIKLKNYFSSNQISITMLEAR*

>Glyma.12G145300|Glyma.12G145300.1|19403597|GNA/S-locus glycoprotein/PAN/protein
kinase
MVDNFRMLFIWFLLFSYLRNSTSLDSLAPSQSIRDSERLVSKEGTFEAGFFSPGTSTRRY
LGIWYRDVSPLTVVWVANREKPVYNKSGVLKLEERGVLMILNSTNSTIWRSNNISSTVKN
PIAQLLDSGNLVVRNERDINEDNFLWQSFDYPCDTFLPGMKLGWNLVTGQDRFLSSWKSE
DDPAKGDYSLKLDLRGYPEFFGYEGDAIKFRGGSWNGEALVGYPIHQLVQQLVYEFVFNK
KDVYYEYKILDRSIIYIFTLTPSGFGQRFLWTNQTSSKKVLSGGADPCENYAICGANSIC
NMNGNAQTCDCIKGYVPKFPGQWNVSYWSNGCVPRNKSDCKTSNTDGLLRYTDMKIPDTS
SSWFNKTMNLEECQKSCLKNCSCKACANLDIRNGGSGCLLWFDDLVDMRQFSKGGQDLYF
RAPASELVNSHGKNLKKLLGITIGAIMLGLTVCVCMILILKKQGLARIIDRNHFKHKLRK
EDDDLSTFDFAIIARATGNFAKSNKLGEGGFGPVYKVIWETCHRCFFLKMFLTIVDVALQ
ARLLDGQEFAVKRLSNKSGQGLEEFKNEVMLIAKLQHRNLVKLIGCSIEGKERMLIYEYM
PNKSLDYFIFDETRRTMVDWPKHFNIICGIARGILYLHQDSRLRIVHRDLKTSNILLDGN
FDPKISDFGLARTFWGDQVEANTNRLAGTYGYMAPEYAARGQFSMKSDVFSYGVIVLEIV
SGKKNREFSDPKHYLNLLGHTWRLWAEERALELLDGVLKERFTPSEVIRCIQVGLLCVQQ
RPEDRPDMSSVVLMLNGEKLLPNPKVPGFYTEGDVKPESDFSPTNRFSTNQISITMLEAR
*

>Glyma.12G145400|Glyma.12G145400.1|19598643|GNA/S-locus glycoprotein/PAN/protein
kinase
MTLTFRLTSLTTLLFLCMFCVNATTYKEYLWMDQSLGTSDTILSRSGNFELGFFPAVREN
STNYYIGIWNKKGGSDKNKIMWVANRDYAVQASSAALTIQETEGNIIIIDRQMTYHVSQI
SNNSITYAKLLDSGNLLLLNNFTQEILWQSFDYPTDTLLPGMNLGYDTDSGYTWSLSSWK
SADDPAPGAFSLKYDFGRATLIINNGSNVFWIDDRSNDTIDNVISRSGVHKERYRRYFTW
PVDYNSMLVLEVSGELNQKYWSEEEKGWISIQSSKCGTNNLCGAFSICNPQALDPCDCLH
GFKPFDANSWSKGIKSAGCVRKKELSCRNGVHSNDVFMPLNKTQLPSTLKGDSKIKIDTE
RGCESACSRKCSCVAYAYNLNGYCHLWLGQILNLKNISTYVDNSDNTNPIFNLRLDASEL
VPADSNTANAKEPANDFRKHENWLRILLIVILITLLTFLIFGLFVYWIRKQRRKGEDLLH
FNVSMSMKAEDSELTEARRAKVKKKEVKLPLFSFVSVAAATNNFSDDNKLGEGGFGPVYK
GILLNGDEVAVKRLSRRSGQGWEELRNEALLIAKLQHNNLVRLLGCCIDQEEKMLIYEFM
PNRSLDVFLFDATKRRMLDWGSRVRIIDGIAQGVLYLHQYSRFRIIHRDLKASNILLDTN
MNPKISDFGMARIFGENELQASTKRIVGTYGYMSPEYAMEGVFSIKSDVFSFGVLLLEII
SGKKNTSFYQTNSLCLLGYAWDLWTNNSVMDLMDPTLDDSDSTSSRNHTVPRYVNIGLLC
VQESPADRPTMSDAVSMIGNDNVALPSPKPPAFLNVRGNQNSILPNSIPESFSLNVITNT
IVEPR*

>Glyma.12G198000|Glyma.12G198000.1|35923580|GNA/S-locus glycoprotein/protein
kinase
MRTDEVLFSFSLFSLVLCFQLCSTGDTLKAGQKITLNSLENLVSSNRTFELGFFPLSGSS
SVVKSYLGIWYHGLEPQTVVWVANRDKPVLDSSGVFRIAEDGNLVIEGASSESYWSSKIE
ASSSTNRTVKLLESGNLVLMDDNLGRSNYTWQSFQHPTDTFLPGMKMDASVALISWRNST
DPAPGNFTFTMAPEDERGSFAVQKLSQIYWDLDELDRDVNSQVVSNLLGNTTTRGTGSHN
FSDKTIFTSKPYNYKKSRLLMNSSGELQFLKWDEDEGQWEKHWWGPADECDIHDYCGSFG
ICNRNNHIGCKCLPGFAPIPEQSEGELQGHGCVRKSTSCINTDVTFLNLTNIKVGNADHE
IFTETEAECQSFCISKCPLCQAYSYNRSTYSDRSPFTCNIWTQNLSYLVEEYDRGRDLSI
LVKRSDIAPTAKTCEPCGTYEIPYPLSTGPNCGDSMYNKFNCTKSTGQVNFMMPEGISYQ
VTRIEEDTRTFFIQADASYSCSSRRDQNNTPNFPFNVVDCIQDFGIVKFSWQPAPEPPCN
RPMDCMNWPHSTCRETRCHCDLKYRWNNSIMSCTQEEPSREHLTHRLTLILTVTLGSMAI
LACITAFVLVRRKKKAHKLDRASTQIQESLYESEKRVKGLIGLGSLEEKDIEGIEVPCYT
FASILAATDNFTDSNKLGRGGYGPVYKGTFPGGQDIAVKRLSSVSTQGLEEFKNEVILIA
KLQHRNLVRLRGYCIKGDEKILLYEYMPNKSLDSFIFDRTRTLLLDWPIRFEIIVGIARG
MLYLHQDSRLRVIHRDLKTSNILLDEEMNPKISDFGLAKIFGGKETEASTERVVGTYGYM
APEYALDGLFSFKSDVFSFGVVLLEILSGKRNTGFYQSKQISSLLGHAWKLWTENKLLDL
MDPSLGETCNENQFIKCALIGLLCIQDEPGDRPTMSNVLSMLDIEAVTMPIPTPPTFFVN
KRHSSSASSSSKPETSLQFDSSYQEGR*

>Glyma.12G198100|Glyma.12G198100.1|35933643|GNA/S-locus glycoprotein/protein kinase
MRTDEVLFSFSLFSLVLCFQLCSTGDTLKAGQKITLNSFENLVSSNRTFELGFFPLSGSS
SVVKRYLGIWYHGLEPQTVVWVANRDKPVLDSNGVFRIAEDGNLVIEGASSESYWSSKIE
AYSSTNRTVKLLESGNLVLMDDNLGRSNYTWQSFQHPTDTFLPGMKMDASVALISWRNST
DPAPGNFTFTMVPEDERGSFAVQKLSQIYWDLDELDRDVNSQVVSNLLGNTTTRGTRSHN
FSNKTVYTSKPYNYKKSRLLMNSSGELQFLKWDEDEGQWEKRWWGPADECDIHDSCGSFG
ICNRNNHIGCKCLPGFAPIPEGELQGHGCVRKSTSCINTDVTFLNLTNIKVGNPDHEIFT
ETEAECQSFCISKCPLCQAYSYHTSTYGDRSPFTCNIWTQNLSSLVEEYDRGRDLSILVK
RSDIAPTAKTCEPCGTYEIPYPLSTGPNCGDPMYNKFNCTKSTGQVNFMTPKGISYQVTR
IEEDTRTFFIHTNASYSCSSRRDQSNTPNFPFNVAECIPDVGVLFRWQPAPEPPCNRPMD
CMNWPYSTCRETSEGGTRCHCDLKYRWNNSIMSCTQEPPSNRSYKRLELILTIILISTIT
LACIIVLAIVRRKKNAPKPDRASTQIQESLYESERQVKGLIGLGSLEEKDIEGIEVPCYT
YASILAATDNFSDSNKLGRGGYGPVYKGTFPGGQDIAVKRLSSVSTQGLEEFKNEVILIA
KLQHRNLVRLRGYCIEGDEKILLYEYMPNKSLDSFIFDPTRTSLLDWPIRFEIIVGIARG
MLYLHQDSRLRVIHRDLKTSNILLDEEMNPKISDFGLAKIFGGKETEACTGRVMGTFGYM
APEYALDGFFSTKSDVFSFGVVLLEILSGKKNTGFYQSKQISSLLGHAWKLWTENKLLDL
MDPSLCETCNENEFIKCAVIGLLCVQDEPSDRPTMSNVLFMLDIEAASMPIPTQPTFFVK
KHLSSSASSSSKPDIGQYESSYQEGR*

>Glyma.12G198600|Glyma.12G198600.1|35997653|GNA/S-locus glycoprotein/PAN/protein kinase
MHRNPSTISFLISPYNHMRNPWFCISLLTLFFSLLTHNSLAALTNVSSNQTLTGDQTLLS
KGEIFELGFFKPGGNLVLLDGSSNQVWSTNITSPRSDSVVVAVLRDSGNLVLTNRPNDAS
ASDSDSLWQSFDHPTDTWLPGGKIKLDNKTKKPQYLTSWKNNEDPATGLFSLELDPKGST
SYLILWNKSEEYWTSGAWNGHIFSLVPEMRANYIYNFSFVTNENESYFTYSMYNSSIISR
FVMDVSGQVKQFTWLENAQQWNLFWSQPRQQCEVYAFCGAFGSCTENSMPYCNCLPGFEP
KSPSDWNLVDYSGGCERKTMLQCENLNPSNGDKDGFVAIPNIALPKHEQSVGSGNAGECE
SICLNNCSCKAYAFDSNGCSIWFDNLLNLQQLSQDDSSGQTLYVKLAASEFHDDKSKIGM
IIGVVVGVVVGIGILLAILLFFVIRRRKRMVGARKPVEGSLVAFGYRDLQNATKNFSEKL
GGGGFGSVFKGTLGDSSGVAVKKLESISQGEKQFRTEVSTIGTVQHVNLVRLRGFCSEGA
KRLLVYDYMPNGSLDFHLFHNKNSKVLDWKMRYQIALGTARGLTYLHEKCRDCIIHCDVK
PENILLDAEFCPKVADFGLAKLVGRDFSRVLTTMRGTRGYLAPEWISGVAITAKADVYSY
GMMLFEFVSGRRNSEPSEDGKVTFFPSFAANVVVQGDSVAGLLDPSLEGNAEIEEVTRII
KVASWCIQDNEAQRPSMGQVVQILEGILEVNLPPIPRSLQVFVDNQESLVFYTESDSTQS
SQVKSNVSKTSSSHAISNISSASSKSLGGEN*

>Glyma.12G198800|Glyma.12G198800.1|36004701|GNA/S-locus glycoprotein/PAN/protein kinase
MRNPWICISLLTLFFSLFTHNSLAALPTVSSNQTLTGDQTLLSKGGIFELGFFKPGNTSN
YYIGIWYKKVTIQTIVWVANRDNPVSDKNTATLTISGGNLVLLDGSSNQVWSTNITSPRS
DSVVVAVLNDTGNLVLKPNDASASDSDYLWQSFDHQTDTFLPGGKIKLDNKTKKPQYLTS
WKNNQDPATGLFSLELDPKGSNSYLILWNKSEEYWTSGAWNGQIFSLVPEMRLNYIYNFS
FVMNENESYFTYSMYNSSIMSRFVMDVSGQIKQFSWLEKTQQWNLFWSQPRQQCEVYAFC
GVFGSCTENSMPYCNCLPGFEPKSPSDWNLFDYSGGCERKTKLQCENLNSSNGDKDGFVA
IPNMALPKHEQSVGSGNVGECESICLNNCSCKAYAFDGNRCSIWFDNLLNVQQLSQDDSS
GQTLYVKLAASEFHDDKNRIEMIIGVVVGVVVGIGVLLALLLYVKIRPRKRMVGAVEGSL
LVFGYRDLQNATKNFSDKLGEGGFGSVFKGTLGDTSVVAVKKLKSISQGEKQFRTEVNTI
GKVQHVNLVRLRGFCWEGTKKLLVYDYMPNGSLDCHLFQNNNCKVLDWKTRYQIALGTAR
GLAYLHEKCRDCIIHCDVKPGNILLDADFCPKVADFGLAKLVGRDLSRVITAVRGTKNYI
APEWISGVPITAKVDVYSYGMMLFEFVSGRRNSEQCEGGPFASFPIWAANVVTQCDNVLS
LLDPSLEGNADTEEVTRMATVALWCVQENETQRPTMGQVVHILEGILDVNLPPIPRSLQV
FVDN*
>Glyma.12G240300|Glyma.12G240300.1|39899116|GNA/protein kinase
MASSTFLHSYSLLLLIILPFLPSVFSATSSNCSANSIHLNSTLVTNHTWNSPSGLFAFGF
QNVLSNKEFMSVLAVWFPKDPHRTIVWYAKYKQTSDLGTMHAVSSMQKSLAFPSDSTVKL
TNKGIVLYDQNGQEMWHRPKNNSIALVRCASMLDSGNFVLLDETGKHVWESFEEPTDTFL
PGQILAKPKSFRARHSNTSFYDGSFELAWQSDYNFVLYYSPQSSVTREAYWATQTNSYDE
SLLVFNESGHMYIKRSNTGKVIREVLYGGSEEFLYMARIDPDGLFRLYRHRKDDDTIADS
CSSGWWSVVDRYPKDICLSITMQTGNAICGYNSYCITINGNPSCECPDIFSSFDHDNNLK
TCRPDFPLPSCNKDGWEQNKDLVDFKEYQNLDWPLSDYDKLVGTAMDKDMCRQKCLEDCF
CAVAIYGEGQCWKKKYPLSNGRKHPNVTRIALVKIPKTGLNKDGTGSLGNGREQSTIVLV
ISILLGSSVFLNVILLVALFAAFYIFYHKKLLNSPNLSAATIRYYTYKELEEATTGFKQM
LGRGAFGTVYKGVLKSDTSRYVAVKRLDKVVQEGEKEFKTEVSVIGQTHHRNLVRLLGYC
DEEEHRLLVYEYMNNGSLACFLFGISRPHWNQRVQIALGIARGLTYLHEECSTQIIHCDI
KPQNILLDELFTPRIADFGLAKLLLAEQSKATKTGLRGTVGYFAPEWFRKASITTKVDVY
SFGVVLLEIICCKSSVSFAMASEEETLIDWAYRCYSQGKVAKLVENDEEAKKDIKRVEKH
VMVAIWCIQEDPSLRPSMKKVTQMLEGVTTVSLPPRPAIFSSSSFETSFTL*

>Glyma.13G160900|Glyma.13G160900.1|27666836|GNA/S-locus glycoprotein/PAN/protein kinase
MISTSTSVDSLAVDQLIRDGETLVSASGITEVGFLSPGDSKRRYLGIWYRNISPLTVVWV
ANRNTPLQNTSGVLKLNQKGFLVLLNATNSAIWSSNILSTALGFGKFCRMKLGWNLETGL
ERYGLSWKSVENPAEGDYTVKIDLGGYPQMVIFRVPDIKTRIVPWNGLSIVGYPGPNHLS
LQEFVINEKEVYYEYELLDRSVFSLYTLAPSGTGQGLFWTTEISTRKVVSIGEQDQCENY
AFCGTNSICSYEGNYSTCECVKGCVPKFPQYWNLSIWSNGCVPRIKSNCKNGYTYGFLKY
TQMKLPDTSSSWFNKTMKLEDCHKLCLENCSCLAYASLDVRGGGSGCLLWFNNLADLRKF
SQWGQDLYIKVPASDLYHVATGHENIKKKIVGITVGVTTFGLIITCVCILIIKNQRAARK
IYNKHYKSKQIIEDIDLPTFALSALANATENFSTKNKLREGGFGPVYKGTLMDGKVLAVK
RLSKKSIQGLDEFKKEVALIAKPQHRNLVKLLGCCIEGEEKMLIYEYMPNQSLDYFVFGL
LYLHQDSRLRIIHRDLKTSNILLDANLDPNISDFGLARSFFGDQVAGTYGYMPPEYAARG
HFSLKSDVFSYGVILLEIVSGNKNREFADPENYNNLLGNAWRLWTEERTLEILDDVLEEK
CTPHERPIVPGFYTQNDVAFEADHNLCSVNELSITVLDAGLERKAGKC*

>Glyma.13G166900|Glyma.13G166900.1|28169255|GNA/S-locus glycoprotein/protein kinase
MAFIPVVLSLLFMLFAEQGTGKRVIELGSRLSPEGNQSSWASSSGHFAFGFYSQGDGFAV
GIWLVNVSPAENTIVWTANRDSPPLSSNSTLQLTKTGLLFFQDGRQGQVLLSNFVDVTSS
ASMLDSGNFVLYDDTHNTVVWQSFEHPTDTILGGQNLSINAKLVSSVSNSSHSSGRFFLL
MQGDGNLVAYPVNSPETGGDAYWASNTKGSTFQHLSLNVLGFLCLSGPGNLHCFFNHSIS
PGMKLQNKTSIYRSTVDVDGNLRLYEHQLEGNGSSHVQVLWSTPLKKCETKGFCGFNSYC
SIVTGHAMCECFPGFVPSKSNGSVSLDCVLAHSKGSCKSSEDAMISYKITMLENMSFSDS
DDPYWVSQMKKEECEKSFLEDCDCMAVLYLNGNCRKYRLPLTYGRTIQNQVAVALFKVPS
GIVDSSTPNNSTLKPRIIVDNKKRLVMVLAITLGCFLLLSLALAGFIFLIYKRKVYKYTK
LFKSENLGFTKECSLHPFSFDELEISTRSFTEEIERGSFGAVYRGTIGDTNTSIAVKRLE
TIADEGEREFRTEITAIARTHHKNLVKLIGFCINGARKLLVYEYVSNGSLASLLFNDEKH
MSWRDRLKIALDVARGVLYLHEECEVRIIHCIWTAKISDFGLAKLLLKLDHSRMKNEDDET
SKYLAPEWQKDAPISVKFDIYSFGMVLLEIVCRRRSIEMNVSSVEEIHLSSWVYQCFAAG
QLNKLVKEDESTVDWRILERMVKVGLWCVQDSPPLRPSIKNVILMLEGLKDIPIPPPPAE
TSLNKYS*

>Glyma.13G167000|Glyma.13G167000.1|28174907|GNA/S-locus glycoprotein/protein kinase
MKDDAEPSAGDVMLEIYGTRVEMKQIQPGASLVPNTTLAWWPSPSGQFAFGFYPQEQGDA
FVIAIWLVSGENKIVVWTARRDDPPVTSNAKLQLTKDGKFLLIDEHGEEKSIADIIAKAS
SASMLDSGNFVLYNNNSSIIWQSFDYPTDTLLGGQSLPNGHQLVSASSNNSHSTGRYRFK
MQDDGNLVMYPVSTTDTALDAYWASSTTNSGFKTNLYLNQTGLLQILNDSDGSIMKTLYH
HSSFPNDGNRIIYRSTLDFDGFFRLYKHFDNGSFQKAHHWPDENACAVKGFCGFNSYCTF
NDTQPLCTCLPDFELIYPTDSTRGCKRSFQNEDCNGQKDSATFYDMKPMEDTFVGTDNPY
FKAKMPKEDCSSACLADCSCEAVFYDDTEESCMKQRLPLRYLRRPGQDEFGVNQALLFLK
VGNRSLNNGTGNDNPVPEQPSPTPIKTTRNKATVQIVVITSVFSLLLCSTIVISSHYMYK
IRILSYERLMEMGNWGLSEELTLKRFSYSELKRATNNFKQKLGRGSFGAVYKGGLNKGSF
GAVYKGGLNKGRRLIAVKRLEKLVEEGEREFQAEMRAIGKTHHRNLVRLLGFCAEGSKRL
LVYEYMPNGSLENLIFGAQSQRRPGWDERVRIALEIAKGILYLHEECEAPIIHCDIKPQN
ILMDEFWTAKISDFGLAKLLMPDQTRTITGARGTRGYVAPEWDKLNIPISVKVDVYSYGI
VLLEILCCRRNIEVHVSEPEAALLSNWAYKCFVSGQLNKLFLWESVDNKTSVENIVKVAL
WCIQDEPFLRPTMKSVVLMLEGITDIAIPPCPNSSYV*

>Glyma.13G248300|Glyma.13G248300.1|35640626|GNA/S-locus glycoprotein/PAN/protein kinase
MDFTSLILALVIVCCFCQCLSSGNDTITPGQFIRDPHTLTSANSAFKLGFFSPQNSSNRY
LGIWYLSDSNVIWVANRNQPLKKSSSGTVQISEDGNLVVLDSNKRAVWSTNLTHNIATNS
TAKLLETGNLVLLDDASGQTTWESFRHPCHALVPKMKFGSNQKTGEKIRITSWRSASDPS
VGYYSTTLEHPNTPEMFFWLNETRPYHRSGPWNSQIFIGSTEMSPGYLSGWNIMNDVDDE
TVYLSYTLPNQSYFGIMTLNPHGQIVCSWWFNEKLVKRMVMQRTSCDLYGYCGAFGSCSM
QDSPICSCLNGYKPKNVEEWNRKNWTSGCVRSEPLQCGEHTNGSKVSKDGFLRLENIKVP
DFVRRLDYLKDECRAQCLESCSCVAYAYDSGIGCMVWSGDLIDIQKFASGGVDLYIRVPP
SELEKLADKRKHRKFIIPVGVTIGTITLVGCVYLSWKWTTKPTGNVYSLRQRMNRDHNEV
KLHDQLPLFSFEELVNATNNFHSANELGKGGFGSVYKGQLKDGHEIAVKRLSKTSGQGLE
ECMNEVLVISKLQHRNLVRLLGCCIKKKENMLVYEYMPNKSLDVILFDPVKKKDLDWPKR
FNIIEGISRGLLYLHRDSRLKIIHRDLKVSNILLDGELNPKISDFGMARIFGGNDIQTNT
RRVVGTFGYMPPEYAFRGLVSEKLDVFSFGVLLLEIISGRKISSYYDHDQSMSLLGFAWK
LWNEKDIQSVIDPEISNPNHVNDIERCIHIGLLCLQNLATERPIMATVVSMLNSEIVNLP
RPSHPAFVDRQIVSSAESSRQNHRTQSINNVTVTDMQGR*

>Glyma.13G248400|Glyma.13G248400.1|35651954|GNA/S-locus glycoprotein/PAN/protein kinase
MIMDITSLILALFIVYCFCQCLSSANNTITSGQYITDPHTLISPNSVFKLGFFSPQNSSN
RYLGIWYLSDSNVIWVANRNQPLKTSSSGTVQISEDGNLVVLDSNKRVVWSSNVTHNIAT

NSTAKLLETGNLVLIDDATGESMWESFRHPCHALVPKMKLSITQKTYEKVRITSWRSPSD
PSLGYYSATLERPNIPEVFYWINETQPYYRTGPWNGQIFIGSPQMSRGYLYGWNMMNDED
DGTVYLSYNLPSQSYFAVMTLNPQGHPTIEWWRDRKLVWREVLQGNSCDRYGHCGAFGSC
NWQSSPICNCLSGYKPKYVEEWNRKNWTSGCVRSEPLQCGEQTNGSEVSKDGFLRLENMK
VSDFVQRLDCLEDECRAQCLENCSCVAYAYDNGIGCMVWSGDLIDIQKFSSGGIDLYIRV
PPSESELEKHSDKRRHKIILIPVGITIGMVALAGCVCLSRKWTAKSIGKINSQRQGMNED
QKQVKLNDHLPFFSFEELVNATNNFHSANELGKGGFGSVYKGQLKDGHEIAVKRLSKTSG
QGLEECMNEVLVISRLQHRNLVRLLGCCIEQEENMLVYEYMPNKSLDVILFDPAKKQDLD
WPKRFNIIEGISRGLLYLHRDSRIKIIHRDLKVSNILLDGELNPKISDFGMAKIFGGNDM
QANTRRVVGTFGYMPPEYAFQGLVSEKLDVFGFGVLLLEIISGRKISSCFDHDQSLSLLG
FAWKLWNEKDIQSLIDPEISNPNNVNDIVRCIHIGLLCSQELAKERPLMATVVSMLNSEI
VDLPPPLNPAFIKRQIVSCADSSQQNHITQSINNVTVTGIQGR*

>Glyma.13G248500|Glyma.13G248500.1|35658070|GNA/S-locus glycoprotein/PAN/protein kinase
MNHGIDINLVNCTVAWFKKTSSHYIHSSADTVTVLSTNIMGFLNALLIVFPIIFLGLTSA
TDTLTSSQSIRDSETVVTSNDSVFKLGFFSPQNSTHRYVGIWYLSDSNVIWIANRNKPLL
DSSGVLKISKDGNLVLVDGKNHVIWSSNVSNTATITSTAQLSRSGNLVLKDDSTGQTLWE
SFKHPCDSAVPTMRISANRITGEKIRFVSRKSASDPSTGYFSASLERLDAPEVFLWINGT
RPYWRTGPWNGRIFIGTPLMSTGYLYGWNVGYEGNETVYLTYSFADPSSFGILTLIPQGK
LKLVRYYNRKHTLTLDLGISDCDVYGTCGAFGSCNGQNSPICSCLSGYEPRNQEEWSRQN
WTSGCVRKVPLKCERFKNGSEDEQEDQFLKLETMKVPDFAERLDVEEGQCGTQCLQNCSC
LAYAYDAGIGCLYWTRDLIDLQKFQTAGVDLYIRLARSEFQSSNAQEHTNKTRGKRLIIG
ITVATAGTIIFAICAYLAIRRFNSWKGTAKDSENQSQRVTEVQKPAKLDELPLFDFEVVA
NATDNFHLANTLGKGGFGPVYKGLLPDGQEIAVKRLAKASGQGLEEFMNEVGVISKLQHR
NLVKLLGCCVEGDEKMLIYEFMPNKSLDAFIFDPLRQKLLDWTKRFNIIEGVARGLLYLH
RDSRLKIIHRDLKASNILLDAEMNPKISDFGLARIYKGEDEVNTKRVVGTYGYMSPEYAM
EGLFSEKSDIYSFGVLLLEIISGKRNTSFRNDDQSLSLIGYAWNLWNEDNISFLVDPEIS
ASGSENHIFRCIHIAFLCVQEVAKTRPTMTTVLSMLNSEISHLPPPRQVGFVQKQSSSSL
ESSSQENQFNSNNHVTLTEMQGR*

>Glyma.13G248700|Glyma.13G248700.1|35668869|GNA/S-locus glycoprotein/PAN/protein kinase
MGFSTGGNTLPVFLILSNFYMNWVTALDIITVSRLMKDPETVTSNDGAFKLGFFSPGNTS
NRYVGIWYLSESNVIWVANRGQPLEDSSGVVTISDDRNLVVLNGRKQVVWSSNVSNIESN
STAQLLNTGNLVLLDNITGKTIWESFKHPSNTFTPNMIISTNQVTGEKVKVTSWKSLSDP
AIGTFSGSLERLSVPEVFVWNQTQPCSGPWNGQVFIGLATMYTSAYLNGFSVERENSVTV
QITYTLPNDSFFGTIFLSPEGRMVYTSWINRQLVGKRVIQQSNCDIYGFCGAYGSCDSNK
LPIICSCLRGFEPRNREEWNRQNWTSGCVRREALQCKRSGASKEDGFVKLQMSKVPDFAH
QSSVSVDTCRTECLNNCSCTAYAYDAAIGCMSWSGELIDIVRFSRGGVDLYIRQAHSELD
VGRNMTSIIIVTVIVGTLLVATCAYFLWTWTSKSSARMESQPSLVLRETPPENRNAGLSG
DLNQVKIQDLPVFNFENIATATNYFNLANKLGQGGFGSVYKGVLQDGQEVAVKRLSRTSR
QGTEEFMNEVTVISKLQHRNLVRLLGCCIEGEEKMLIFEYMPNKSLDFYLFDPVKKVVLD
WQKRFNIIEGISRGSLYLHRDSRLRIIHRDLKPSNILLDGELNPKISDFGMAKIFGGSED
EANTRRVVGTYGYMSPEYAMEGLFSEKSDVFSFGVLLLEIISGRKNSSFRNHELSLSLLG
YAWKLWNEEEIVSLVDPEIFSPDNVYHTLRCIHIGLLCVQELAKERPTMATVVSMLNSEI
VNFPPPQQPAFIQRQIELRGESSQQSHNSNSINNVTVTNLQGR*

>Glyma.13G248800|Glyma.13G248800.1|35677452|GNA/S-locus glycoprotein/PAN/protein kinase
MAAAMMISFYLFLFSFHNLFSLAASSKIRITQGVTIRDKEHETLVSEELNFAMGFFSFDN
SSSRYVGIWYDNIPGSEVIWVANRDKPINGTVGAITIANDGNLVVLDGAMNHVWSTNVSI
DDNNKNSSATLRDDGNLVLTCERKEVWQSFENPTDTYMPGMKVSVGGLSTSHVFTSWKSA
TDPSKGNYTMGVDPEGLPQIVVWEGEKRRWRSGYWDGRMFQGLSIAASYLYGFTLNGDGK
GGRYFIYNPLNGTDKVRFQIGWDGYEREFRWNEDEKSWNEIQKGPFHECDVYNKCGSFAA
CDVLTLSPEDLVPVCTCIRGFEPKHKDQWDKGNWSGGCTRMTPLKAQRINVTSGTGVSVG
EDGFLDRKSMKLPDFALVVGTNDCDRECFSNDSCTAYANVNGLGCMVWHGDLVDIQHLES
GGNTLYIRLAHSDLDDGGKTNRIVIISTVVAGLICLGIFVWLVWRFKAKLKVLPTVSSVS
CCKSSNVLPVFDENKSREMSAEFSGSADLTLEGNQLSGPEFPVFNFSCISIATNNFSEEN
KLGQGGFGPVYKGKLPGGEQIAVKRLSRRSGQGLEEFKNEMMLIAKLQHRNLVRLMGCSI
QGEEKLLVYEYMPNKSLDCFLFDPVKQTQLPWTRRFEIIESIARALLYLHRDSRLRIIHR
DLKASNILLDENMNPKISDFGLARIFGGNQNEANTNRVVGTYGYMAPEYAMEGLFSVKSD
VYSFGVLLLEILSGRRNTSFRHSDDSSLIGYAWHLWNEHRAMELLDPCIRDSSPRNKALR
CIHIGMLCVQDSAAHRPNMSAVVLMLESEATTLPMPTQPLITSMRRTEDRQFYMDGLDVS
NDLTVTMVVGR*

>Glyma.13G248900|Glyma.13G248900.1|35685233|GNA/S-locus glycoprotein/PAN/protein kinase/SRK
MPGFLFFLFCFFLASSQHITISFSADTLTSTQILLTNQTLISPSQVFALGFFPGTNSTWY
LGTWYNNINDRTIVWVANRDNPLENSNGFLTIAENGNIVLTNPSMKKYPVWSSNATTKAN

NNNRVLQLLDTGNLVLREANITDPTKYLWQSFDYPTDTLLPGMKMGWNLDTGVEKHLTSW
KATGSDPSSGDYSFKIDTRGIPEIFLRDDQNITYRSGPWNGERFSGVPEMQPNTDTITFD
FSYDKDGVYYLFSIGSRSILSRLVLTSGGELQRLTWVPSRNTWTKFWYARKDQCDGYREC
GPYGLCDSNASPVCTCVGGFRPRNLQAWNLRDGSDGCVRNTDLDCGRDKFLHLENVKLPE
TTYVFANRTMNLRECEDLCRKNCSCTAYANIEITNGGSGCVTWTGELIDMRLYPAGGQDL
YVRLAASDVDDIGSGGGSHKKNHIGEVVGITISAAVIILGLVVIFWKKRKLLSISNVKAG
PRGSFQRSRDLLTTVQRKFSTNRKNSGERNMDDIELPMFDFNTITMATDNFSEANKLGQG
GFGIVYRGRLMEGQDIAVKRLSKSSMQGVEEFKNEIKLIVRLQHRNLVRLFGCCIEMHER
LLVYEYMENRSLDSILFDKAKKPILDWKRRFNIICGIARGLLYLHHDSRFRIIHRDLKAS
NILLDSEMNPKISDFGMARLFGSNQTEANTSRVVGTYGYMSPEYAMDGNFSVKSDVFSFG
VLVLEIITGKKNRGFYYSNEDMNLLGNAWRQWRDGSALELIDSSTGDSYSPSEVLRCIHV
GLLCVQERAEDRPTMSSVLLMLSSESVLMPQPRNPGFSIGKNPAETDSSSSKKDESWSVN
QVTVTLLDAR*

>Glyma.13G249000|Glyma.13G249000.1|35692094|GNA/S-locus glycoprotein/PAN/protein kinase
MNKVVTIIFALVCQPILQKASYAGAALTQTSSITDGQELISARQIFSLGFFTPRRSSSRY
IGIWYKNVKPQTVVWVANRDNPLNDISGNLTIAADGNIVLFDGAGNRIWSTNIYRSIERP
IAKLLDSGNLVLMDAKHCDSDTYIWQSFDYPTDTMLPGMKLGWDKTSDLNRCLTSWKTAK
DPSPGSFTYSFLHIEFPEFLIRQGMDITFRSGIWDGTRFNSDDWLFNEITAFRPHISVSS
NEVVYWDEPGDRLSRFVMRGDGLLQRYIWDNKTLMWIEMYEIRKDFCDNYGVCGVNGVCN
IEDVPVYCDCLKGFIPCSQEEWDSFNRSGGCIRRTPLNCTQDDGFQKLSWVKLPMPLQFC
TNNSMSIEECRVECLKNCSCTAYANSAMNGGPHGCLLWFGDLIDIRQLINEKGEQLDLYV
RLAASEIEAIAKASKRRKIALIISASSLALLLLCIIFYLCKYIKPRTATDLGCRNHIEDQ
ALHLFDIDIILAATNNFSIENKIGEGGFGPVYRGKLSSRQEIAVKRLSKTSKQGISEFMN
EVGLVAKFQHRNLVSVLGGCTQGDERMLVYEYMANSSLDHFIFDAVHRKLLKWRKRYEII
LGVARGLLYLHQDSNLTIIHRDLKTSNILLDKEFNPKISDFGLAHIFEGDHSTVTTKRIV
GTVGYMSPEYAVNGLLSLKSDVFSFGVIVLEILSGIKNNNFNHPDDSNLLGQAWRLWIEG
RAVEFMDVNLNLAAIPSEILRCLHVGLLCVQKLPKDRPTMSSVVFMLSNESITLAQPKQP
GFFEEVLQSQGCNNKESFSNNSLTITQLEGRT*

>Glyma.13G249200|Glyma.13G249200.1|35701949|GNA/S-locus glycoprotein/PAN/protein kinase
MRRRRVRMNKIVIIFACLSMLQKMAYAADALTPTSSINDGQELISAGQNFSLGFFTPGIS
KSRYVGIWYKNIMPQTVVWVANRDYPLNDSSGNLTIVAGNIVLFDGSGNRIWSTNSSRSS
IQEPMAKLLDSGNLVLMDGKSSDSDSYIWQSFDYPTDTTLPGLKLGWDKTSGLNRYLTSW
KSANDPSAGSFTYGFHHNEITEFVLRQGMKITFRSGIWDGTRLNSDDWIFNEITAFRPII
SVTSTEALYWDEPGDRLSRFVMKDDGMLQRYIWDNKVLKWIEMYEARKDFCDDYGACGVN
GICNIKDVPVYCDCLKGFKPKSQEEWNSFNRSGGCIRRTPLNCTQGDRFQKLSAIKLPKL
LQFWTNNSMNLEECKVECLKNCSCTAYANSAMNEGPHGCFLWFGDLIDIRKLINEEAGQL
DLYIKLAASEIESTANAIKRRKIALIISASLVALLLLCIILYLSKKYIKERTTTDLVAGN
RNHNEHQASPLFHIDTILAATNNFSTANKIGEGGFGPVYRGKLADGQEIAVKRLSKTSKQ
GISEFMNEVGLVAKLQHRNLVSILGGCTQGDERMLVYEYMANSSLDHFIFDPTQRKFLNW
RKRYEIIMGISRGLLYLHQDSKLTIIHRDLKTSNILLDSELNPKISDFGLAHIFEGDHST
VTTKRIVGTVGYMSPEYAANGLLSLKSDVFSFGVIVLEILSGIRNNNFYHSDHERNLLVQ
AWRLWKEGRAVEFMDANLDLATIRSELLRCLQVGLLCVQKLPKDRPTMSSVVFMLSNESI
TLAQPKKPEFIEEGLEFPGYSNNSMTITLLEARN*

>Glyma.13G249300|Glyma.13G249300.1|35712072|GNA/S-locus glycoprotein/PAN/protein kinase/SRK
MERTEFTLLFLVTCCYLLSLFPTALEAEDAITPPQTISGYQTLVSPSQNFELGFFSPGNS
THIYLGIWYKHIPKQTVIWVANRDKPLVNSGGSLTFSNNGKLILLSHTGSVVWSSNSSGP
ARNPVAHLLDSGNFVLKDYGNEGHLWESFDYPSDTLIPGMKLGWNFKTGLNRHLTSWKSS
SNPSSGEYTYGVDPRGIPQLFLHKGNKKVFRSGPWYGQQFKGDPVLSANPVFKPIFVFDS
DEVSYSYETKDTIVSRFVLSQSGLIQHFSWNDHHSSWFSEFSVQGDRCDDYGLCGAYGSC
NIKSSPVCKCLKGFDPKLPQEWEKNEWSGGCVRKNSQVFSNGDTFKQFTGMKLPDAAEFH
TNYTISSDHCEAECSMNCSCVAYAKLDVNASGKGCIVWFGDLFDIREVSVNGEDFYVRVP
ASEVGKKIKGICSRSMSCFSPILAVFRNMAHLFLSPNVDGNKRKKLILFPVTAFVSSTII
VSALWLIIKKCRRKRAKETDSQFSVGRARSERNEFKLPLFEIAIIEAATENFSLYNKIGE
GGFGHVYKGQLPSGQEIAVKRLSENSGQGLQEFKNEVILISQLQHRNLVKLLGCCIHGED
KMLVYEYMPNRSLDSLLFDETKRSVLSWQKRLDIIIGIARGLLYLHRDSRLRIIHRDLKA
SNVLLDGEMNPKISDFGMARMFGGDQTEAKTKRIVGTYGYMSPEYAIDGHFSFKSDVYSF
GVLLLELLSGKKNKGFIHPDHKLNLLGHAWKLWNEDRALELMDALLENQFPTSEALRCIQ
VGLSCIQQHPEDRPTMSSVLLMFDSESVLVPQPGRPGLYSERFFSGTNSSSRGGLNSGSN
DITVTLVEGR*

>Glyma.13G270100|Glyma.13G270100.1|37204783|GNA
MASVPQPKISLLTLFFFSSFTIIAHAIVPQNETFKFENSGELGPYIVEYGADYRMISIFN
SPFQVGFYNTTPNAFTLALRVGLQRSEQLFRWVWEANRANPVGENATFSLGTDGNLVLAD
ADGRIAWQTNTANKGVVAFRLLSNGNMVLLDAQGGFVWQSFDHPTDTLLVGQYLRAKGPS

KLVSRLSEKENVDGPYSLVLEPKGLALYYKSKNSPKPILYWFSSDWFTIQRGSLENVTFT
SDPETFELGFDYHVANSSSGGNRILGRPVNNSTITYLRLGIDGNIRFYTYFLDVRDGVWQ
VTYTLFDRDSDESECQLPERCGKFGLCEDNQCVACPLENGLLGWSNNCTAKAVTSCKASD
FHYYKIEGVEHYMSKYTTGDRVSESTCGNKCTKDCKCVGYFYHKENSRCWVAYDLQTLTR
GANSSHVGYIKVPNK*

>Glyma.13G270200|Glyma.13G270200.1|37209460|GNA
MASLPQPKTSPLTLFFFSSFTIIAHAIVPQNETFKFENSGELGPYIVEYGVDYRMISIFN
SPFHRMGLRRSEQLFRWVWEANRGNPVGENATFSLGTDGNLVLAEADGRIAWQTNTANKG
VVAFRLLPNGNMVLLDAQGKFLWQSFDHPTDTLLNDQYLRPKGPSKLISRLSEKENVDGP
YSLVLEPKRLALYYKSKNSPKPILYWYKLFTQQGSSSSTNEFRTMATGFGNIPVGVMGMP
VNNSTLTYLRLGIDGNIRLHTYFLGVRSGVWQVTYTLFNRDSHDEFECQWPEKCGKLGNN
CTAKPVKSCKARDFHCYKVEGVRHYLSKYTEGGKVRESTCGNKCTKDCKCVGCFYHGEVK
VLDSL*

>Glyma.13G283100|Glyma.13G283100.1|38451885|GNA/S-locus glycoprotein/PAN/protein kinase
MQNLRTQWFWFFLFCCISRTSTSLDSIAPNQSISDGETLISHEKTFELGFFSPGSSKSRY
LGIWYYNINPRTMVWVANREAPLNTTSGVLKLSDQGLVLVNGTNNIVWSSNMSTTAETEN
TIAQLLDSGNLVVKDGNSEYEHYLWQSFDHPCDTLLPGMKLGWNLEKGEELFLSSWKSAD
DPSHGEYSFKIDPRGCPQAVLWKGTNLSNRFGPWNGLYFSGSLIDSQSPGVKVDFVLNKK
EIYYQFQVLNKSLSYRFWVTPNRNALVSLWESQISDWLILYSQPSFPCEYYGRCGANSIC
NAGNPRCTCLDGFFRHMNSSKDCVRTIRLTCNKDRFRKYTGMVLPDTSSSWYNKNMVLEE
CAEMCLQNCSCTAYANLDISGGGSGCLLWYHDLIDLRHYPQAQGGQDIYIRYSDSELDHS
QKNGLSKSKIASIVTGSTTFVVSMILGLVIWLWKRKVEMEEMKKQLYQSHHNYNLRKEEP
DLPAFDLPVIAKATDNFSDTNKLGEGGFGPVYKGTLIGGQDIAVKRLSNNSGQGLKEFKN
EVALIAKLQHRNLVKLHGYCIQEEEKMLIYEYMPNMSLDYFIFDEIRTKLLDWSKRFHII
GGIARGLVYLHEDSRLRVIHRDLKTSNILLDENMNPKISDFGLARTLWGDQVDANTNKIA
GTYGYMPPEYAVHGHFSMKSDVFSFGVMVLEIVSGKKNRDFSDPNHCLNLLGHAWRLWTE
GRPTNLMDAFLGERCTSSEVIRCIHVGLLCVQQRPNDRPDMSAVVLMLNGEKSLPQPKAP
GFYNGRDKADLFGPFSNNDASLTVLEAR*

>Glyma.13G283200|Glyma.13G283200.1|38458232|GNA/S-locus glycoprotein/PAN/protein kinase
MDSYEVMIVWFSLLFSLIPSISTRLSSITLDQPLHHNETLVSASGTFEAGFFSTGSSQRQ
YFCICYKNISPRTIVWVANRNTPLDNNFTGVFKVSDEGNLVVLDGIGASVWSSNASTTSQ
KPIVQLLDSGNLVVKDGGTNSPEKVVWQSFDFPGDTLLPGMKLRSSLVTGAHSSLTSWRD
TEDPALGEYSMYIDPRGFPQRVTTKGGTWLYRAGSWNGYQFSGVPWQLLHNFFNYYFVLT
PKEVYYEYELLEPSVVTRFVINQEGLGQRFTWSERTQSWELFASGPRDQCENYGLCGANS
VCKINSYPICECLEGFLPKFEEKWRSLDWSDGCVRGTKLGCDDGDGFVKYEGMRLPDTSS
SWFDTSMSLDECESVCLKNCSCTAYTSLDIRGDGSGCLLWFGNIVDMGKHVSQGQEIYIR
MAASELDQTWHKRHASTKLVVILATIATFIVGLILGSVIYIRRKLGKPGKTNIIDQMHHS
IKHEKKDIDLPTLDLSTIDNATSNFSASNILGEGGFGPVYKGVLANGQEIAVKRLSKNSG
QGLDEFRNEVVLIANLQHRNLVKILGCCIQDDERILIYEFMPNRSLDLYIFDRTRKKLLD
WNKRFQIISGIARGLLYLHHDSRLRIIHRDIKTSNILLDNDMNPKISDFGLARMLVGDHT
KANTKRVVGTHGYMPPEYAVYGSFSVKSDVFSFGVIVLEIVSGRKNTKFLDPLNQLNLIG
HAWRLWSEGRTLELIDESLDDSIIESEVLKIVHVGLLCVQERPEDRPNMSSVVLMLNGDR
PLPRPKLPAFYPHQEDFSSSSKCEFSSNELSITLEAR*

>Glyma.13G283400|Glyma.13G283400.1|38473895|GNA/S-locus glycoprotein/PAN/protein kinase/SRK
MEGFTLLLFCLALLNSIAAATVRETISTLQSINDDQIIVSPGKTYALGFFSPGNSKNRYV
GIWYNEIPTQTVVWVANRDNPLADSSGVLKLNETGALVLLNHNKSVVWSSNASKPARYPV
AKLLDSGNLVVQDGNDTSETKDLLWQSFDYPGDTILPGQKFGRNLVTGLNRFMSSWNSTD
DPSQGEYSYQIDISGYPQLVLREGAFKRYRFGSWNGIQFSGAPQLKQNNFTRFSFVSDEE
ELYFRFEQTNKFVFHRMQLSTDGYILGDYWNTEEKVWSLHGKIPVDDCDYYDKCGAYASC
NINNVPPCNCLDGFVSKTDDIYGGCVRRTSLSCHGDGFLKLSGLKLPDTERSWFNRSISL
EDCRTLCMNNCSCTAYAALDVSKGPTGCLLWFDDLVDIRDFTDVDEDIYIRVAGTEIEAI
EGKQLHKSSIRKPQTVFISCVLFIAILILCLTFIIYRRWKTRQKGGKMKDKLERDASVIY
EHEKDDLELPMFEWSTITCATNNFSPDNKLGEGGFGSVYKGILDDGGEIAVKRLSKNSSQ
GLQEFKNEVMHIAKLQHRNLVRLLGYCIQAEERLLVYEFMANKSLDSFIFDENKSMLLDW
PRRSLIINGVARGLLYLHQDSRHRIVHRDLKAGNVLLDSEMNPKISDFGLARSFGGNEIE
ATTKHVVGTYGYLPPEYIIDGAYSTKSDVFSFGVLILEIVSGKRNKGFCHQDNLLAHVWR
LFTEGKCSEIVDATIIDSLNLPEVLRTIHVGLLCVQLSPDDRPNMSSVVLMLSSESELPQ
PNLPGFFTSTSMAGDSSSSSSYKQYTNNDMTVSIMSAR*

>Glyma.13G284100|Glyma.13G284100.1|38509353|GNA/S-locus glycoprotein/PAN/protein kinase/SRK
MKNHIMPFMRIIFLCYHILVYLSGISLALDSISQDLSLSDDGKNTTLVSKDGTFELGFFT
PGNSQKRYLGIWYRKIPIQTVVWVANRLNPINDSSGILRMNPSTGTLVLTHNGTVIWSTA

SIRRPESPVALLLNSGNLVIRDEKDANSEDYLWESFNYPTDTFLPEMKFGWDLRTGLNRK
LIAWKSPDDPSPSDFSFGMVLNNYPEAYMMKGDQKFYRSGPWNGLHSSGSPQVKANPIYD
FKFVSNKDELYYTYSLKNSSMISRLVLNATSYVRKRYVWIESKQRWEVYTSVPLDLCDSY
SLCGANANCVISDSPVCQCLQGFKPKLPEAWSSMDWSHGCIRNKELSCENKNKDGFNKLT
LLKTPDTTHSWLDQTIGLEECKAKCLDNCSCMAYANSDISGQGSGCAMWFGDLIDIRQFA
AGGQDVYVRIDASELEHANEGHKKGGVLVAVTVTLALAAVAGILIILGWCYRKKSRCSVK
ERSDFSIKSNQNSGMQVDDMDLPVFDLSTIAKATSNFTVKNKIGEGGFGPVYRGSLTDGQ
EIAVKRLSASSGQGLTEFKNEVKLIAKLQHRNLVKLLGCCLEGEEKMLVYEYMLNGSLDS
FIFDEQRSGSLDWSKRFNIICGIAKGLLYLHQDSRLRIIHRDLKASNVLLDSELNPKISD
FGMARIFGVDQQEGNTKRIVGTYGYMAPEYATDGLFSVKSDVFSFGVLLLEIISGKRSRG
YYNQNHSQNLIGHAWKLWKEGRPLELIDKSIEDSSSLSQMLHCIHVSLLCVQQNPEDRPG
MSSVLLMLVSELELPEPKQPGFFGKYSGEADSSTSKQQLSSTNEITITLLEAR*

>Glyma.13G303400|Glyma.13G303400.1|40047660|GNA/S-locus glycoprotein/PAN/protein kinase
MVPNSAFTILLGSVPTRVGPVPFIISLLLICLLHCSLQLLIGVRATPMYSMRNQRFCVYL
LTLFFNLFTHNFLAALTTISTNQTLTGDQTLVSEAGVFELGFFKPGNSSNYYIGIWYKRV
TIQTIVWVANRDNPVSDKSTAKLTISGGNLVLLDASSNQVWSTNITSPMSDSVVVAVLLD
SGNLVLTNRPNGASASDSLWQSFDHLTDTFLPGGKIKLDNKTKKPQYLTSWKNNQDPATG
LFSLELDPEGSNAYLISWNKSEEYWTSGAWNGHIFSLVPEMRLNYIFNFSFVSNENESYF
TYSLYNTSIISRLVMDVSGQIKQLSWLENAQQWNLFWSQPRQQCEVYAFCGAFGSCTENV
MPYCNCLTGFEPKSPFDWNLVDYSGGCKRKTKLQCENSNPFNGDKDGFIAIPNLVLPKQE
QSVGSGNEGECESICLNNCSCTAYAFDSNGCSIWFDNLLNVQQLSQDDSSGQTLYVKLAA
SEFHDDNSRIGMIVSVVVGVIVGIGVLLALLLYVKIRKRKRMVRAVEGSLVAFRYRDLQN
ATKNFSEKLGEGGFGSVFKGTLGDTGVVAVKKLESTSHVEKHFQTEITTIGKVQHVNLVR
LRGFCSEGSKKLLVYDYMPNGSLDFHLFQNKNSKVLDWKTRYQIALGTARGLAYLHEKCR
ECIIHCDVKPGNILLDADFCPKLADFGLAKLVGRDLSRVVTAARGTTNYIAPEWISGVPI
TAKVDVYSYGMMLFEFVSGRRNSEQCEGGPFAFFPTWAANIVAHGDNVLSLLDPNLEGNV
DAEEVTRMVTVALWCVQENETQRPTMGQVIHILDGILDVNLPPIPRSLKVFVDN*

>Glyma.13G303800|Glyma.13G303800.1|40087141|GNA/S-locus glycoprotein/protein kinase
MRTGELLLFFSFLVSLALWFQLCFAGDTLIAGQEITQNRTGNLVSSSRRTFELGFFSLSGE
KKYYLGIWYRELEKETQKAVWVANRDKPVEDSSRVFRIAEDGNMVVEGASSKRYWSSKLE
ASSSTNRTVKLLDSGNLVLMDDNLGITSYLWQSFQNPTDTFLPGMKMDANLSLISWKDAT
DPSPGNFSFKLIHGQKFVVEKHLKRYWTLDAIDYRIARLLENATSGKVPYKLSGITLNPG
RAYRYGKSMLLMNYSGEIQFLKWDEDDRQWDKRWSRPADKCDIYNCCGSFGFCNKNNLNL
NLEPCRCLPGFRRRPAGEIQDKGCVRKSTSSCIDKKDVMFLNLTNIKVGDLPDQESFDGT
EAECQSLCLNNNTKCSESQCQAYSYSNSTSYDRDHSSTCKIWRRDLSTLLERYNSDFLEE
FVPGPILSILVKRSDIVPYAKSCEPCGIYVIPYPLSTGPNCGDPMYNNFNCNKSTGQVTF
KILGGTSHQVIWIDEDTRMFYIQPNGSYPCNSSNQNITPNFPFNVTDQCSEADDDGKIKI
TWLPAPEPPCTELIDCHNWPHSTCRETSEGGSRCRCDSNYKWNNTIMSCTLEEHSTNQLE
LILIVILSGMAILACTIAFAIVRRKKKAHELGQANARIQESLYESERHVKGLIGLGSLAE
KDIEGIEVPCYTFASILAATANFSDSNKLGRGGYGPVYKGTFPGGQDIAVKRLSSVSTQG
LQEFKNEVILIAKLQHRNLVRLRGYCIKGDEKILLYEYMPNKSLDSFIFDRTRTLLLDWP
MRFEIILGIARGLLYLHQDSRLRVIHRDLKTSNILLDEDMNPKISDFGLAKIFGGKETEA
STERIVGTYGYMAPEYALDGFFSIKSDVFSFGVVLLEILSGKKNTGFYQSKQISSLLGHA
WKLWTEKKLLDLMDQSLGETCNENQFIKCAVIGLLCIQDEPGDRPTMSNVLYMLDIETAT
MPIPTQPTFFVNKHFSSSASSSSKPEISLQFESSYQEGR*

>Glyma.13G365400|Glyma.13G365400.1|45150280|GNA/protein kinase
MGIFRCGTLFFHVLLLFRTCLAKDQHVSQIYPGFSASQPDWSDHNGFFLLSNSSAFAFGF
FTTLDVSSFVLVVMHLSSYKVVWTANRGLLVGTSDKFVLDHDGNAYLEGGNGVVWATNTR
GQKIRSMELLNSGNLVLLGENGTTIWQSFSHPTDTLLPGQDFVEGMTLKSFHNSLNMCHF
LSYKAGDLVLYAGFETPQVYWSLSGEQAQGSSKNNTGKVHSASLVSNSLSFYDISRALLW
KVVFSEDSDPKSLWAATLDPTGAITFYDLNKGRAPNPEAVKVPQDPCGIPQPCDPYYVCF
FENWCICPKLLRTRYNCKPPNISTCSRSSTELLYVGEELDYFALKYTAPVSKSNLNACKE
TCLGNCSCLVLFFENSTGRCFHFDQTGSFQRYKRGAGAGGYVSFMKVSISSASDDGHGNK
NGRNDMVLVVVIVLTVLVIVGLITGFWYLFKRKKNVAKYPQDDLDEDDDFLDSLSGMPAR
FTFAALCRATKDFSSKIGEGGFGSVYLGVLEDGTQLAVKKLEGVGQGAKEFKAEVSIIGS
IHHVHLVKLKGFCAEGPHRLLVYEYMARGSLDKWIFKNSENTFLLNWDTRYNIAIGTAKG
LAYLHEECDVRIIHCDIKPQNVLLDDNFTAKVSDFGLAKLMSREQSHVFTTLRGTRGYLA
PEWITNYAISEKSDVFSYGMLLLEIIGGRKNYDQWEGAEKAHFPSYVFRMMDEGKLKEVL
DPKIDIDEKDERVESALKIALWCIQDDVSLRPSMTKVAQMLDGLCPVPDPPSLSQSGTYS
AFMKLSSGEATSSGQASFFSNVPMSCVQLSGPR*

>Glyma.14G054300|Glyma.14G054300.1|4312928|GNA/S-locus glycoprotein/PAN/protein kinase
MVRSIIMLCIWFFIFFDLPGTSTLIDSLAAGQSIRDGETLVSAGGITKVGFFSPGNSTRR
YLGIWYTNVSPITVVWVANRNSPLENNSGVLKLNEKGILELLNGKNSTIWSSNISSKAVN

YPIAQLLDSGNFVVKYGQEITNEDSVLWQSFDYPCDSLMPGMKLGWNLETGLERYLSSWR
SVDDPALGEYTVKIDLRGYPQIIKFKGPDIISRAGSWNGLSTVGNPGSTRSQKMVINEKE
VYFEFELPDRSEFGISSLTPSGTSLILYWTTQRSTRQAVLSNADKDQCGSYAFCGANSIC
IYDGNVPTCECLRGYAPKHPDQWNIAIWSDGCVPRNKSNCTNSYTDGFLKYTNMKLPDTS
SSWFSKTMNLDECQKSCLKNCSCTAYANLDIRDGGSGCLLWFNTLVDLRNFSELGQDFYI
RLSASELDHGGQRKINKKIVAIAVGVTIFGLIITCVCILVIKNPGAARKIYNKNYRNILR
KEDIDLPTFSFSVLANATENFSTKNKLGEGGYGPVYKGKLLDGKELAVKRLSKKSGQGLE
EFKNEVALISKLQHRNLVKLLGCCIEGEEKILIYEYMPNHSLDYFVFDESKRKLLDWDKR
FDIISGIARGLLYLHQDSRLRIIHRDLKTSNILLDENLDPKISDFGLARSFLGDQVEANT
NRVAGTYGYMPPEYAARGHFSVKSDVFSYGVIVLEIVTGKKNREFSDPECYNNLLGHAWR
LWTEEMALELLDEVLGEQCTPSEVIRCVQVGLLCVQQRPQDRPNMSSVVLMLNGEKLLPK
PKVPGFYTEAEVTSEANNSLGNPRLCSVNELSITMFDAR*

>Glyma.14G054500|Glyma.14G054500.1|4351031|GNA/S-locus glycoprotein/PAN/protein kinase
MAFLVIVILVSKLIFFSSNFLAATDMINQFQSLEDNTTLVSNDGTFELGFFTPGSTSPNR
YLGIWYKNIPIRTVVWVANRDNPIKDNSSKLSINTAGNFILLNQNNNTVIWSTNTTTKAS
LVVAQLLDSGNLVLRDEKDNNPENYSWQSFDYPSDTFLPGMKAGWDLKKGLNRVLTAWKN
WDDPSSGDFTANSSRTNFPEEVMWKGTSEYYRSGPWDGRKFSGSPSVPTNSIVNYSVVSN
KDEFYATYSMIDKSLISRVVVNQTLYVRQRLTWNEDSQTWRVSSELPGDLCDNYSTCGAF
GICVAGQAPVCNCLDGFKPKSTRNWTQMNWNQGCVHNQTWSCMEKNKDGFKKFSNLKAPD
TERSWVNASMTLDECKNKCRENCSCTAYANFDMRGEGSGCAIWFGDLLDIRLIPNAGQDL
YIRLAVSETDEKDDSKKKVVVIASIVSSVVATLLIFIFIYWSNAKNIKEIILGIEVKNNE
SQQEDFELPLFDLVSIAQATDHFSDHNKLGEGGFGPVYKGTLPDGLEVAVKRLSQTSGQG
LKEFKNEVMLCAKLQHRNLVKVLGCCIQENEKLLIYEYMANKSLDVFLFDSDRSKLLDWP
KRFYIINRIARGLLYLHQDSRLRIIHRDLKASNVLLDNEMNPKISDFGLARMCGGDQIEG
KTRRVVGTYGYMAPEYAFDGLFSIKSDVFSFGVLLLEIVSGKKNNRLFYPNDYNNNLIGH
AWSLWNEGNPMEFIATSLEDSCILYEALRCIHIGLLCVQHHPNDRPNMASVVVLLSNENA
LPLPKYPRYLITDISTERESSSEKFTSYSINDVTISMLSDR*

>Glyma.14G116500|Glyma.14G116500.1|14152829|GNA/protein kinase
MGKQWSLFSVMDTLLLCLLLSSEVVLTSFLNVGKVYPRIEGSQMNWIDRDGILLVSNKGE
FGFGLVTTANDSTLFLLAIVHKYSNKVVWVANRALPVSNSDKFVFDEKGNVILHKGESVV
WSSDTSGKGVSSMELKDTGNLVLLGNDSRVIWQSFRHPTDTLLPMQDFNEGMKLVSEPGP
NNLTYVLEIESGNVILSTGLQTPQPYWSMKKDSRKKIINKNGDVVTSATLNANSWRFYDE
TKSMLWELDFAEESDANATWIAGLGSDGFITFSNLLSGGSIVASSTRIPQDSCSTPESCD
PYNICSGDKKCTCPSVLSSRPNCQPGNVSPCNSKSTTELVKVDDGLNYFALGFVPPSSKT
DLIGCKTSCSANCSCLAMFFNSSSGNCFLLDRIGSFEKSDKDSGLVSYIKVVSSEGDIRD
SSKMPIIVVVIIVIFTLFVISGMLFVAHRCFRKKQDLPESPQEDLEDDSFLESLTGMPIR
YSYNDLDPATSNFSVKLGEGGFGSVYKGVLPDGTQLAVKKLEGIGQGKKEFWVEVSIIGS
IHHHHLVRLKGFCAEGSHRLLAYEYMANGSLDKWIFNKNIEEFVLDWDTRYNIALGTAKG
LAYLHEDCDSKIIHCDIKPENVLLDDNFMVKVSDFGLAKLMTREQSHVFTTLRGTRGYLA
PEWITNCAISEKSDVYSYGMVLLEIIGARKNYDPSETSEKSHFPSFAFRMMEEGNLREIL
DSKVETYENDERVHIAVKVALWCIQEDMSLRPSMTKVVQMLEGLCIVHKPAICSVLGSRF
YSTSEVGTSSGPSDCNSEANLSAVRLSGPR*

>Glyma.15G007800|Glyma.15G007800.1|643762|GNA/protein kinase
MGIFRCGALFFYVLLLFRTCLAKDQHVSQIYPGFSASQPDWSDHNGFFLLSNSSAFAFGF
FTTLDVSSFVLVVMHLSSYKVVWTANRGLLVGTSDKFVLDRDGNAYLEGGNSVVWATNTT
GQKIRSMELLDSGNLVLLGENGTAIWQSFSHPTDTLLPRQDFVDGMTLKSFHNSLNMCHF
LSYKAGDLVLYAGFETPQVYWSLSGEQAQGSSRNNTGKVHSASLVSNSLSFYDINRALLW
KVVFSEHSDPKSLWAATLDPTGAITFYDLNKGRAPNPEAVKVPQDPCGIPQPCDPYYVCF
FENWCICPKLLRTRFNCKPPNISTCSRSSTELLYVGEELDYFALKYTAPVSKSNLNACKE
TCLGNCSCLVLFFENSTGRCFHFDQTGSFQRYKRGAGAGGYVSFMKVSISSASDDGHGNK
NRRNDAVLVVVIVVLTVLVIVGLIMGFWYFYKRKKNVAKYPQDDLDEDDDFLDSLSGMPA
RFTFAALCRATKDFSTKIGEGGFGSVYLGVLEDGIQLAVKKLEGVGQGAKEFKAEVSIIG
SIHHVHLVKLKGFCAEGPHRLLVYEYMARGSLDKWIFKNSDNTFLLNWDTRYNIAIGTAK
GLAYLHEECEVRIIHCDIKPQNVLLDDNFTAKVSDFGLAKLMSREQSHVFTTLRGTRGYL
APEWITNYAISEKSDVFSYGMLLLEIVGGRKNYDQWEGAEKAHFPSYVFRMMDEGKLKEV
LDPKIDIDEKDERVEAALKVALWCIQDDVSLRPSMTKVAQMLDGLCPVPDPPSLSQSGTY
SAFMKLSSGEATSSGQASFFSNVPMSCVQLSGPR*

>Glyma.15G064900|Glyma.15G064900.1|4941726|GNA/S-locus glycoprotein/PAN/protein kinase
MKKVVIIIFALACLSILQKTSYAADVLTPTSSIKGGQELISAGQNFSLGFFTPGTSKSRY
VGIWYKNILPQTIVWVANRDSPLNDTSGNLTVAADGNIVLFDGAGNRIWFTNSSRPIQEP
IAKLLDSGNLVLIDGKNSDSDSYIWQSFDYPTDTMLPGLKLGWDKTSGLNRYLTSWKSAN
DPSPGNFTYRFDQKEFPELVIRQGMNITFRSGIWDGIRFNSDDWLSFNEITAFKPQLSVT
RNEAVYWDEPGDRLSRFVMRDDGLLQRYIWDNKILKWTQMYEARKDFCDTYGACGANGIC
NIKDLPAYCDCLKGFIPNSQEEWDSFNWSGGCIRRTPLNCTEGDRFQKLSWVKLPMLLQF

WTNNSMSLEECHVECLKNCSCTAYANSALNEGPHGCLLWFGNLIDIRLLITEEDAGGQLD
LYVRLAASEIESTANASKRRKIALIISASSLALLLLCIILCLSKKYIKRRRTTTDLGHRN
HNEDQAPPLFEIYTILAATNNFSIENKIGEGGFGPVYRGKLAHGQEIAVKRLSKTSKQGI
SEFMNEVGLVAKLQHRNLVSVLGGCTQGEERMLVYEYMPNSSLDHFIFDPKQGKTLKWRK
RYDIIVGIARGLLYLHQDSKLTIIHRDLKTSNILLDNELNPKISDFGVSRIVEGDHFAVT
TNEIVGTIGYMSPEYAVNGLLSVKSDVFSFGVIVLEILSGTKSNNFKVKHPDHDHNLLGQ
AWRLWNEERAVEFMDVNLDLTTIPSEFMSCLQVGLLCVQNLPEDRPTMSSVVFMLSNESI
ALGQPKKPDFFEEKLEYRGYSEKESFSNNAMTITLLEARN*

>Glyma.15G065100|Glyma.15G065100.1|4949613|GNA/S-locus glycoprotein/PAN/protein kinase/SRK
MPGFLFFLFCFFFASSQHIAISFSTDTLSSTQILLTNQTLVSPSHIFALGFFPGTNSTWY
LGAWYNNITDDKTVVWVANRDNPLENSSGFLTIGENGNIVLRNPSKKNPVWSSDATKANN
PVLQLLDTGNLILREANITDPTKYLWQSFDYPTDTLLPGMKMGWNLDTGAEKHLTSWKNT
GSDPSSGDYSFKIDTRGIPEIFLSDDQNIAYRSGPWNGERFSGVPEMQPDTDSITFDFSY
DKHGVYYSFSIGNRSILSRLVVTSGGELKRLTWVPSSKTWTTFWYAPKDQCDGYRACGPY
GLCDSNASPVCTCVGGFRPRNQQAWNLRDGSDGCERNTDLDCGSDKFLHVKNVKLPETTY
VFANGSMNLRECQDLCLRDCSCTAYANIQITNGGSGCVTWSGELEDMRLYPAGGQHLYVR
LAASDVDDIVGGSHKKNHTGEVVGITISAAVIILGLVVIFWKKRKLFSISNVKTAPRGSF
RRSRDLLTSERMFSTNRENSGERNMDDIELPMFDFNTITMATDNFSEANKLGQGGFGIVY
RGRLMEGQDIAVKRLSKNSVQGVEEFKNEVKLIVRLQHRNLVRLFGCCIEMDEKLLVYEY
MENRSLDSILFDKAKKPILDWKRRFNIICGIARGLLYLHHDSRFRIIHRDLKASNILLDS
EMNPKISDFGMARLFGTNQTEANTLRVVGTYGYMSPEYAMDGNFSVKSDVFSFGVLVLEI
ITGKKNRGFYYSNEDMNLLGNAWRQWRDGSTLELIDSSIGDSCSQSEVLRCIHVGLLCVQ
ERAEDRPTMSSVLLMLSSESAIMPQPRNPGFSIGKNPVETDSSSSKKDQSWSVNQVTVTL
LDAR*

>Glyma.15G065200|Glyma.15G065200.1|4960133|GNA/S-locus glycoprotein/PAN/protein kinase
MAAATIIYFYFFLFSFVSFHHLLFSFAASSKTRITQGVTIRDKEHETLVSEELNFAMGFF
SSDNSSSRYVGIWYDNIPGPEVIWVANRDKPINGTGGAITISNDGNLVVLDGAMNHVWSS
NVSNINSNNKNSSASLHDDGNLVLTCEKKVVWQSFENPTDTYMPGMKVPVGGLSTSHVFT
SWKSATDPSKGNYTMGVDPEGLPQIVVWEGEKRRWRSGYWDGRMFQGLSIAASYLYGFTL
NGDGKGGRYFIYNPLNGTDKVRFQIGWDGYEREFRWNEDEKSWSEIQKGPFHECDVYNKC
GSFAACDLLTLSPSSDLVPVCTCIRGFEPKHRDQWEKGNWSGGCTRMTPLKAQRINVTSS
GTQVSVGEDGFLDRRSMKLPDFARVVGTNDCERECLSNGSCTAYANVGLGCMVWHGDLVD
IQHLESGGNTLHIRLAHSDLDDVKKNRIVIISTTGAGLICLGIFVWLVWRFKGKLKVLPT
VSSVSCCKSSDALPVFDANKSREMSAEFSGSADLSLEGNQLSGPEFPVFNFSCISIATNN
FSEENKLGQGGFGPVYKGKLPGGEQIAVKRLSRRSGQGLEEFKNEMMLIAKLQHRNLVRL
MGCSIQGEEKLLAYEYMPNKSLDCFLFDPVKQKQLAWRRRVEIIEGIARGLLYLHRDSRL
RIIHRDLKASNILLDENMNPKISDFGLARIFGGNQNEANTNRVVGTYGYMAPEYAMEGLF
SVKSDVYSFGVLLLEILSGRRNTSFRHSDDSSLIGYAWHLWNEHKAMELLDPCIRDSSPR
NKALRCIHIGMLCVQDSAAHRPNMSAVVLWLESEATTLPIPTQPLITSMRRTEDREFYMD
GLDVSNDLTVTMVVGR*

>Glyma.15G065500|Glyma.15G065500.1|4976617|GNA/PAN/protein kinase
MVYRISIPHSSTKQKHFPGLWISQVNYLFESNVIWVANRNQPLKKSSSGIIQIYENDNLV
VLDSNKRVVWSSNVAHIATNSTAKLLETGNLVLLDDATGESIWESFQHPCDALVPKLKLS
IKKKHVRNGCVRSEPLQCGEHINGSEVCKDGFLRLENMKVPDFVQRLDCLEDECRAQYLE
NCSCVVYAYDSGIGCMVWNGNLIDIQKFSSGGVDLYIRVPPSESELGMFFFGQLKDGHEI
ALKRLSKTSGQGLEECMNEVLVISKLQHRNLVRLLGCCIEQEENMLVYEFMNEVVVISKL
QHCNLVKLLGCCVEGDEKMLIYEFMPNKSLDAFIFDPLRHKLLDWTKRFNMIEGVARGLL
YLHRDSRLKIIRRDLKASNVLLDAEMNPKISDFGLARIYKGEEEVNTKRVVGTYGYMPPE
HAMEGLFSEKSDVYSLGVLLLEIISGKRNTSFRNDDQSLSLIGYAWNLWNEDNIRYLVDP
EISASGSDSHILRCIHIAFLCVQEVAKTRPTMTTVLSMLNSEISHLLHPRQVGFVQKQSS
SSLDSSSQEN*

>Glyma.15G213600|Glyma.15G213600.1|34069137|GNA/PAN/protein kinase
MVCYKLGESHFLLVLTCLWLWLWWSTRIHVIAAYHSLRPGDTLNSTTELCSENDKYCLGF
SQFSSAHNSTYLRIYAKGKGDWNMWIGNRNQPLDMDSAVLSLSHSGVLKIESKDMEPIIL
YSSTQPSNNTVATLMNTSNFVLQRLQPGGTESTVLWQSFDYPTDKLLPGMKLGVNHKTGR
NWSLVSSMGYANPALGAFRLEWEPRRRELLIKQRGQLCWTSGELGKNIGFMHNTHYMIVS
NDDESYFTITTLNEELTRWELLKTGQLINRNGDDNVARADMCYGYNTDGGCQKWEDIPFC
RNPGDAFELKEVYLNLNLKNFLANSSYSPSDCRDTCWKNCSCDGFTDYYDDGTGCIFVYL
NLTEGADFASGGEKFYILVKNTHHKGTKKWIWISILIVAALFSICAFILYLALKKRKLRF
EDKNRKEMEINKMEDLATSNRFYDARDPEDEFKKRQDLKVFSYTSVLLASNDFSTENKLG
QGGFGPVYKGIQPNGQEVAIKRLSKTSSQGTAEFKNELMLIGELQHMNLVQLLGYCIHGE
ERILIYEYMHNKSLDFYLFDGTRSKLLDWKKRFNIIEGISQGLLYLHKYSRLKVIHRDLK
ASNILLDENMNPKISDFGLARMFTRQESTTNTSRIVGTYGYMSPEYAMEGVFSVKSDVYS
FGVLLLEIVSGRRNTSFYDGDRFLNLIGHAWELWNEGACLKLIDPSLTESPDLDEVQRCI

HIGLLCVEQNANNRPLMSQIISMLSNKNPITLPQRPAFYFGSETFDGIISSTEFCTDSTK
AITTSREIESSEHQWGEEARNQLHFS*

>Glyma.15G213700|Glyma.15G213700.1|34098941|GNA/PAN/protein kinase
MVSCKTQFLLVFVYLWLWWTTCSHVISAKHSLKPGEKLNSKTNICSENGIYCISFSSSTG
DCYLQIVRKRGDSWSVWFANRNEPVDAESAVLLLDHSGVLKIESKHRELPIILYFSPQPA
NNTMATLLDTGNFVLQQLHPNGTKSVLWQSFDYPTDNLLPGMKLGVSYKTSHNWSLVSWL
TSEIPNLGAFSLEWQPRTRELIIKRREQLCWTSGELRNKEGFMHNTHYRIVSNENESYFT
ITTSNEELTRWVLLETGQLINRNGGDDVARADMCYGYNTDGGCQKWDEIPICRHRGDAFE
DRLGYPSTNMESYLANSSYGPSDCQDICWKNCSCIAYSDYDGNNETGCTFYHWNSTKGTN
LASGGMKFRLLVKNTDRKGTKKWIWITILIVATLVVISAFVLFLALKNRKLLFKEERRKG
MKTNKMTDLATANRFYDVKDLEDEFKKRQDLKVLNYTSVLSATDDFSTENKLGQGGFGPV
YKGILPTGQEVAIKRLSKTSTQGIVEFKNELMLISELQHTNLVQLLGFCIHEEERILIYE
YMPNKSLDFYLFDCTRSMLLDWKKRFNIIEGISQGILYLHKYSRLKIIHRDLKASNILLD
ENMNPKISDFGLARMFMQQESTGTTSRIVGTYGYMSPEYAMEGTFSTKSDVYSFGVLLLE
IVSGRKNTSFYDVDHLLNLIGHAWELWNQGESLQLLDPSLNDSFDPDEVKRCIHVGLLCV
EHYANDRPTMSNVISMLTNESAPVTLPRRPAFYVERKNFDGKTSSKELCVDSTDEFTAST
Y*

>Glyma.15G213800|Glyma.15G213800.1|34233827|GNA
MANTCKQGKSHLFLVLGYSWLWWTTCNVVAARTQILKPGDTLNSITKLCSESSKYFMKFS
ETGSGAGAYLFINQPVDADSVVLSLDLSGQLKIESNNTKTIILYSSPPPSNNIVATSLDI
GNFLLQHHRSDVTESELWKSFDYPTYTLLPGMMLGVNFKTGPN*

>Glyma.15G214400|Glyma.15G214400.1|34499742|GNA/PAN/protein kinase
MCLQRPGIFKFAAEFEGGAYRSCEKLVIRFGITLLTSISFSIFEAAGYEAYGNFVLQQLH
PNGTNTLLWQSFDYPSTLIPTMKLGVNHKTGHQWVLVSSLTDLVLNPGAFSLEWEPKGQE
LVIRRRGKVCWQSGKLRNNRFEYIPEEAQRMLKYTIVSNGDEDSFSFNSTNDKLTPRWSF
SRSGRLSCNEGYVKADLCYGYNNTGGCQRWQDLPKCRNPGDLFVKKTLFPDYENVTFEMN
PAFGYSDCEASCWSNCSCDGFSALWVNETGCTFYHWNSSKNFVDTSVAGVELYMLENTGN
ITPHNGTKRWIWLSAVIATTLLIIFLSILCLLKKRKYLGQEKKRKEMVMKMPHSTICDGL
SSIEDFGNVFKKGHELNVFDYTLVMMATNGFSSENKLGQGGFGPVYKGILPTGQEVAVKR
LSKTSTQGIMEFKNELTLICELQHMNLVQLLGCCIHEEEKILIYEYMPNKSLDFYLFDSS
RSKLLDWNKRFNIIEGIAQGLLYLHKYSRLKVVHRDLKASVIP*

>Glyma.15G221800|Glyma.15G221800.1|40112860|GNA/S-locus glycoprotein/PAN/protein kinase/SRK
MVTTLFIWFFLFSHMTRASTSVDSLAVDESIRDGETLVSAGGIIEAGFFSPEKSTRRYLG
LWYRNVSPLTVVWVANRNTPLENKSGVLKLNEKGILVLLNATNTTIWSSSNNTVSSKARN
NPIAQLLDSGNFVVKNGQSNKDDSGDVLWQSFDYPGDTLLPGMKIGWNLETGLERFLTSW
KSVDDPAEGEYIVKMDVRGYPQLMKLKGTDIRFRAGSWNGLSLVGYPATASDMSPEIVFN
EKEVYYDFKILDSSAFIIDSLTPSGNLQTLFWTTQTRIPKIISTGEQDQCENYASCGVNS
ICNYVDNRPTCECLRGYVPKSPNQWNIGIRLDGCVPRNKSDCKSSYTDGFWRYTYMKLPD
TSSSWFNKTMNLDECRKLCLQNCSCTAYANLDIRDGGSGCLLWFSTLVDLRKFSQWGQDL
FIRVPSSELDHGHGNTKKMIVGITVGVTIFGLIILCPCIYIIKNPGAARKFYNRNYQHIL
KKEDIDLPTFDLSVLVNATENFSTGNKLGEGGFGPVYKGTLMDGKVIAVKRLSKKSGQGV
DEFKNEVALIAKLQHRNLVKLFGCCIEGEEIMLIYEYMPNQSLDYFVFDETKRKFLEWHK
RFKIISGIARGLLYLHQDSRLRIVHRDLKPSNILLDDNLDPKISDFGLARPFLGDQVEAN
TDRVAGTYGYMPPEYAARGHFSVKSDVFSYGVIVLEIVTGKKNWEFSDPKHYNNLLGHAW
KLWTEERVLELLDELLEEQCEPFEVIRCIQVGLLCVQQRPQDRPDMSSVVLMLNGDKLLP
KPKVPGFYTETDNKSEANSSLENYKLYSVNDISITMLDAR*

>Glyma.15G250200|Glyma.15G250200.1|47824041|GNA/S-locus glycoprotein/protein kinase
MASPLLPFLFLSMVLLPFQHINVMAQTKSNIAIGDSHTAGASTSPWLVSSPSGDFAFGFL
PLEDTPDHFMLCIWYAKIQDKTIVWFANRDKPAPKGSKVVLTADDGLVLITAPNGNQLWK
TGGLTVRVSSGVLNNTGNFVLQDGDSNTVWESFKDYRDTLLPYQTMERGQKLSSKLRRNY
FNKGRFVLFFQNDGNLVMHSINLPSGYANEHYYESGTVESNISSAGTQLVFDGSGDMYVL
RENNEKYNLSRGGSGASSTTQFFYLRATLDFDGVFTLYQHPKGSSGTGGWTPVWSHPDNI
CKDYVASAGSGVCGYNSICSLRDDKRPNCKCPKWYSLVDPNDPNGSCKPDFVQACAVDEL
SNRKDLYDFEVLIDTDWPQSDYVLQRPFNEEQCRQSCMEDCMCSVAIFRLGDSCWKKKLP
LSNGRVDATLNGAKAFMKVRKDNSSLIVPTIIVNKNRNTSILVGSVLLGSSAFLNLILLG
AICLSTSYVFRYKKKLRSIGRSDTIVETNLRCFTYKELEKATDGFDKVLGKGAFGIVYEG
VINMGSDTRVAVKRLNTFLLEDVHKEFKNELNAIGLTHHKNLVRILGFCETEEKRLLVYE
YMSNGTLASLLFNILEKPSWELRLQIAIGVARGLLYLHEECSTQIIHCDIKPQNILLDDY
YNARISDFGLAKLLNMNQSRTNTAIRGTKGYVALEWFKNMPITAKVDVYSYGVLLLEIVS
CRKSVEFETEDKEKAILAEWAYDCYTERTLHALVEGDKEALDDMKNLEKLVMIALWCVQE
DPDLRPTMRNVTQMLEGVVEVKVPPCPSQISDQYS*

>Glyma.15G258400|Glyma.15G258400.1|48923351|Thaumatin/GNA/protein kinase

MCRSIIVPISSSSKCNSTGCAVDMNRVCPTELKTIQNGQVVGCQNPCGRRSQERYFCCIG
GDARSNCKPSVYYSKIFKTSCPQAYSHPKGDQTSMFSCYDPVDYNIVFCPTSSNSRSVGD
SLMAGNGTSRWLSPSGDFAFGFYQLPNEFFLLAVWYDKMPNKTIIWFANGDNPAPIGSRL
ELNDSGLVLNNPQGLELWRSNFASGTIFNGLMNDDGNFQLLDQNAVSLWETFTHPTDTLV
PNQVMELNGKLFSRRGEFNFSHGRFKLHLQEDVNLVLSLINLPSNYSYEPYYDTGTADAN
NQTNIGMKLIFDKSGFLYILKKSGEKFYITKPNETISTNDFYYKATINYDGVFTVSYYPK
DLRKGQGWVTTKTIPENICLSSTFTDGEGVCGFNSICNLKADQRPICNCPERYSLIDSNN
MYGGCVPNFQVVCQGGGYMVSQDDYIMKELRNTDWPTSDYETLSPYSLKECTKSCLQDCL
CVLVTFSGSSCWKKKLPLTNGRRDKGVNATSVIKLMKNDYLLTSFPNPNGKKDHDVLIVV
LSVLLAGSVLIILMLVGALYFGFSCNRKKIESSRTNKSVAKKNLHDFTFKELVEATNNFR
EELGRGSFSIVYKGTIEMTSVAVKKLDKLFQDNDREFQTEVNVIGQTHHRNLVRLLGYCN
EGQHRILVYEFMSNGTLASFLFSSLKSNWGQRFDIALGIARGLVYLHEECCTQIIHCDIK
PQNILLDDQYNARISDFGLAKLLLINQSRTETGIRGTKGYVAPDWFRSAPITAKVDTYSF
GVLLLEIICCRKNVEKELVNEEKGILTDWAYDCYKTRRLEILLENDDEAINDIKSFEKLV
MIAIWCIQEHPSLRPTMKKVLLMLEGNVEVLTPPSPYLHGSISEN*

>Glyma.16G034900|Glyma.16G034900.1|3273872|GNA/S-locus glycoprotein/protein kinase
MSLSLFFLLILIFSSSTSSSTTIILQGNTTLKSPNNTFQLGLFSFSFSFYLAIRHTSLPF
PNTTWVANRLHPSPTQTGSILHLTQTGSLILTHSNTTLWSTAPTFNTSSNLSLKLLDSGN
LILSAPNGLVLWQSFDSPTDTWLPGMNLTRFNSLTSWRTQTDPTPGLYSLRLKPPFFGEF
ELVFNDTVSYWSTGNWTDGKFLNIPEMSIPYLYSFHFLSPFSPAAEFGFSERASETGTQP
PTMFRVEPFGQIRQYTWNNQAGSWKMFWSMPEPVCQVRGLCGRFGVCIGETSKLCECVSG
FEPLDGDGWGSGDYSKGCYRGDAGCDGSDGFRDLGDVRFGFGNVSLIKGKSRSFCEGECL
RDCGCVGLSFDEGSGVCRNFYGLLSDFQNLTGGGESGGFYVRVPKGGSGGRKKVFDRKVL
SGVVIGVVVVLGVVVMALLVMVKKKRGGGRKGLEEEEEDGFVPVLNLKVFSYKELQLATR
GFSEKVGHGGFGTVFQGELSDASVVAVKRLERPGGGEKEFRAEVSTIGNIQHVNLVRLRG
FCSENSHRLLVYEYMQNGALNVYLRKEGPCLSWDVRFRVAVGTAKGIAYLHEECRCCIIH
CDIKPENILLDGDFTAKVSDFGLAKLIGRDFSRVLVTMRGTWGYVAPEWISGVAITTKAD
VYSYGMTLLELIGGRRNVEAPLSAGGGGGGGESGDEMGGKWFFPPWAAQRIIEGNVSDVM
DKRLGNAYNIEEARRVALVAVWCIQDDEAMRPTMGMVVKMLEGLVEVSVPPPPKLLQALV
TGDSFHGVKADSGNGASSTGGSLSDGDLEVSTADSESYTGNVFSPLDVNVNVSVR*

>Glyma.16G093900|Glyma.16G093900.1|15240450|GNA/S-locus glycoprotein/PAN/protein kinase
MVTILVRSTTIEHQLQREFWWQTWNQTPQTQSCCISIHKESTIIQRTSRFSTFKTIRFMG
FLTLTNYLIFLLIFSSFYMGVISVNDTITSTRFIRDPETIISSNGDFKLGFFSPEKSTHR
YVAIWYLAETYIIWIANRDQPLSDLSGPGVFKIHKDGNLVVLNAQNRVIWSTNVSITATN
TTAQLDDSGNLILRDVTNGKTLWDSFTHPADAAVPSMKIAANRLTGKKIEYVSWKSSSDP
SSGYFTGSLERLDAPEVYFWYNKTKPYWRTGPWNGRVFLGSPRMSTEYLYGWRFEPNDSG
TAYLTYNFENPSMFGVLTISPHGTLKLVEFLNKKIFLELEVDQNKCDLYGTCGPFGSCDN
STLPICSCFEGFEPRNPEEWNRENWTSGCVRNVQLNCGKLNNTSDVQQDRFRVYQNMKVP
DFAKRLLGSDQDRCGTSCLGNCSCLAYAYDPYIGCMYWNSDLIDLQKFPNGGVDLFIRVP
ANLLVAAAGGKKRRENMGLLIGITGAIGALIIAVGAYLLWRKFTPKHTGNQPQNMITGDQ
KQIKLEELPLFEFEKLSTATNNFHLANMLGKGGFGPVYKGQLDNGQEIAVKRLSKASGQG
LEEFMNEVVVISKLQHRNLVRLLGCCIERDEQMLVYEFMPNKSLDSFLFDPLQRKILDWK
KRFNIIEGIARGILYLHRDSRLRIIHRDLKASNILLDDEMHPKISDFGLARIVRSGDDDE
ANTKRVVGTYGYMPPEYAMEGIFSEKSDVYSFGVLLLEIVSGRRNTSFYNNEQSLSLVGY
AWKLWNEGNIKSIIDLEIQDPMFEKSILRCIHIGLLCVQELTKERPTISTVVLMLISEIT
HLPPPRQVAFVQKQNCQSSESSQKSQFNSNNNVTISEIQGR*

>Glyma.16G157400|Glyma.16G157400.1|31817018|GNA/S-locus glycoprotein/protein kinase
MFLKTQFLFLLTLVLATSTVTTAIDPGSTLSASSSNQTWSSPSGTFSLLFIPVQPPTTPP
SFIAAIAYTGGNPVVWSAGNGAAVDSGGSLQFLRSGDLRLVNGSGSAVWDAGTAGATSAT
LEDSGNLVISNGTGTLWSSFDHPTDTLVPSQNFSVGKVLTSERYSFSLSSIGNLTLTWNN
SIVYWNQGNSSVNASSDSPVLALSPIGLLQLSYAKLSTVALVAYSSDYDKNDGVFRVLKL
DNDGNLRIYSSSLGGGTPSVRWTAVSDQCEVYAYCGNYGVCSYNDSSPVCGCPSQNFEMV
DPNDSRRGCRRKVSLDSCQRNVTVLTLDHTVVLSYPPEAASQSFFIGLSACSTNCLSNSG
ACFAATSLSDGSGQCVIKSEDFVSGYHDPSLPSTSYIKVCPPLAPNPPPSIGDSVREKRS
RVPAWVVVVIILGTLLGLIALEGGLWMWCCRHSTRLGVLSAQYALLEYASGAPVQFSYKE
LQQATKGFKEKLGAGGFGAVYRGTLVNKTVVAVKQLEGIEQGEKQFRMEVATISSTHHLN
LVRLIGFCSEGRHRLLVYEFMKNGSLDDFLFLTEQHSGKLLNWEYRFNIALGTARGITYL
HEECRDCIVHCDIKPENILLDENYVAKVSDFGLAKLINPKDHRHRTLTSVRGTRGYLAPE
WLANLPITSKSDVYGYGMVLLEIVSGRRNFDVSEETNRKKFSIWAYEEFEKGNISGILDK
RLANQEVDMEQVRRAIQASFWCIQEQPSHRPTMSRVLQMLEGVTEPERPPAPKSVMEGAV
SGTSTYLSSNASAFSVGVSPPGPSSSSSFQTSAVSTFTSGRNLEKTTSSLLQSDT*

>Glyma.16G157500|Glyma.16G157500.1|31824629|GNA
MPIFHSISLLPSNTLFKSQFLPLLHMQNKKHKPSTKITMLKIKDLLLSLTLFIISVVAIS
PGSTLYASNTNQAWSSPNNTFSLNFLQVQPPISPPSFMVGIVHSGGVGGGTLVDSRGSFQ

LLSIGSLQLVDGSGAILWNSGTSHFCVFSTFLDEQGNFVLSNGTSTVWSSFDHPTDTPLC
HLRFSLLT*

>Glyma.17G148900|Glyma.17G148900.1|12352255|GNA
MLSAGFFAVGENAYSFVVWYSEPYGQTRNTTVSHVWSSNTISLSSSMLLFLNNTGNLVLR
QTESIGVVLWLSFDFPTDTLLPQQVFTRHAKLVFSRSKTNKSLGFYTLFFDNKNILHLLL
YDGPEVSGL*

>Glyma.17G211200|Glyma.17G211200.1|34877467|GNA/protein kinase
MGRQWSLFSAMDTLLLCILLSSEVVLTSYQNVGKVYPGIEGSQMNWIDRYGILLESYNGE
FGFGLVTTANDSTLFLLAIVHMHTPKLVWVANRELPVSNSDKFVFDEKGNVILHKGESVV
WSTYTSGKGVSSMELKDTGNLVLLGNDSRVIWQSFSHPTDTLLPMQDFIEGMKLVSEPGP
NNLTYVLEIESGSVILSTGLQTPQPYWSMKKDSRKKIVNKNGDVVASATLDANSWRFYDE
TKSLLWELDFAEESDANATWIAVLGSDGFITFSNLLSGGSIVASPTRIPQDSCSTPEPCD
PYNICSGEKKCTCPSVLSSRPNCKPGFVSPCNSKSTIELVKADDRLNYFALGFVPPSSKT
DLIGCKTSCSANCSCLAMFFNSSSGNCFLFDRIGSFEKSDKDSGLVSYIKVVSSEGDTRD
SGSSKMQTIVVVIIVIVTLFVISGMLFVAHRCFRKKEDLLESPQEDSEDDSFLESLTGMP
IRYSYTDLETATSNFSVRLGEGGFGSVYKGVLPDGTQLAVKKLEGIGQGKKEFRVEVSII
GSIHHHHLVRLKGFCAEGSHRVLAYEYMANGSLDKWIFNKNKEEFVLDWDTRYNIALGTA
KGLAYLHEDCDSKIIHCDIKPENVLLDDNFRVKVSDFGLAKLMTREQSHVFTTLRGTRGY
LAPEWITNCSISEKSDVYSYGMVLLEIIGGRKNYDPSETSEKSHFPSFAFKMVEEGNVRE
ILDSKVETYENDERVHIAVNVALWCIQEDMSLRPSMTKVVQMLEGLCTVHKPPTCSVLGS
RFYSTSEVGTSSGPSDCNSEANLSAVRLSGPR*

>Glyma.18G037600|Glyma.18G037600.1|2995717|GNA/PAN/protein kinase
MLYSSSKPESNSNSTIITSAILQDNGNFVLQEINQDGSVKNILWQSFDYPTNMLLPGMKL
GFDRKTGQNWSITSWRSGKSPLSGSFSLGLDHKTKEMVMWWREKIVWSSGQWSNGNFANL
KSSLYEKDFVFEYYSDEDETYVKYVPVYGYIIMGSLGIIYGSSGASYSCSDNKYFLSGCS
MPSAHKCTDVDSLYLGSSESRYGVMAGKGFIFDAKEKLSHFDCWMKCLNNCSCEAYSYVN
ADATGCEIWSKGTANFSDTNNLITGSRQIYFIRSGKAEKRKKQKELLTDIGRSTAISIAY
GERKEQRKDGNTSDETYIFDFQTILEATANFSSTHKIGEGGFGPVYKGKLSNGQEIAIKR
LSKSSGQGLIEFKNEAMLIVKLQHTSLVRLLGFCIDREERILVYEYMPNKSLNLYLFDSN
KRNMLEWKIRCQIIEGVAQGLVYLHQYSRLKVIHRDLKASNILLDNELNPKISDFGTARI
FELAESEEQTNRIVGTYGYMSPEYAMRGVISTKIDVYSFGVLLLEIVSGKKNSDDYPLNL
VVYAWKLWNEGEALNLTDTLLDGSCPPIQVLRYIHIGLLCTQDQAKERPTMVQVVSFLSN
EIAELPLPKQPGFCSSESMEEIEQPKSCSNEITMSLTSEQRKDGRTSDDMHIFNFQTILE
ATAHFSSTNKIREGGFGPVYKGKLLIGQEVAIKRLSKRSGQGLIEFKNEAMLIVELQHTN
LVSLFIFTRNRDSIIPMHF*

>Glyma.18G171500|Glyma.18G171500.1|40396636|GNA/S-locus glycoprotein/protein
kinase
LLLSFKCSSSLLSLNKGSFLSVEKHAEDVIVSQNQMFCAGFSQVRENAFSFDIWFNESHT
HNNSNTVVWIANREQPVNGKLSKISLLNSGKVVLVNAGQIITWSSNTAPHAPVELHLQDD
GNLVLRELQGKICGKFRFHNRYSSSRQTNLSSGFYKLLFDDDNVLRPVYDGSDVSSTYWP
HPWLRSWEAGRFNYNSSRVAVLDSLGNFNSSDSYAFSTDDHGRVMPRRLTLDSDGNVRVY
SRNEALKKWYVSWHFIFDTCTILGICGANSSSNYDPQKQRRCSCLPGYRVKNHSDWSYGD
DGDEGYSCYTKILLLNGRRSQRFKGTIYLRLPKNKNFSREESVSADDHVCSVKLPRDNVR
KPANPLELICFFMIWSFLIWNRQKSGADQQGLHLAEVGIRKFSHFELKEAIKGFSQEIGG
GAGGVVYKDILSDQRHAAIERLYDTKQGEGEFLAEVSIIGRLNLMNLIEMWGYCAEGKHR
LLPKVADFGLSKLLNKNNLNNNSSFSMIRGTRGYMAPEWIYTLPITSKVDVSSFGIVLLE
MITGKSPTMDIETVDGTEPHDGRLVTWVREKKRRTSWIEQILDPSIEPSCDVNKMEILAT
VAFDCVEEDKDVRPTMKQVIEMLQSYESDA*

>Glyma.18G210600|Glyma.18G210600.1|49690288|GNA
MLLLLLLPLMRLFPFLLLLLLLVLLFNYGFSESGVGAGYQLMVAVPVEYEVDFKGRAFLVET
NQTAPNFRVALSIEAINGKYSCSLEVFLGDVKVWDSGHYSRFYITEKCLLELTMDGDLRL
KGPKERVGWKTGTSGQGVKRLEIQRTGNLVLIDALNNIKWQSFNFPTNVMLRGQQLDVAT
RLTSSQSNSSLFYYSFEIEDKKVALYLNYGKLRYSYWGFQPTMNRSITYIKLSSRGLVLF
DVKYKKIAQIPSEGIHPLRFLALNNETGKLGLYYYSPEKGKFEASFQALNSTCDLPIACR
PYGICTFSNSCSCIQLLTNEDKGGADCGWAISGGFCNGKEAEMLELDNVSSVLKNVTKVV
NITKKACENLCFQDCKCAALYFGNASTDEAECYLYKLVLGLKQVEKGTGFSYMVKVPKG
TVKNHERHNVKRWVFVVAGGVDGLIILLLVGGFGYWLVKRRSHALHSRASTT*

>Glyma.19G141400|Glyma.19G141400.1|40269449|GNA/S-locus glycoprotein/protein
kinase
MLEQECLFRPPFLLCILVGFLLHPVVSTVIPLNSKLSMVDNNCWVSSNGDFSFGFFNISD
EPNQFSAGIRFNSKSIPYDQQTVVRVAGAHDKVSNMSYFQLTPEGELILRGTGNRAVASA
TLRDNGNLVLIDTEQNIIWQSFDTPSDTLLPGQSLSVYETLRAMTKNPMSSTYTLYMNPS
SQLQLQWDSHIIYWTSESPSSASNLTAFLTAGGALQLQDPSLKAVWSVFGEGHNDYVNYR
FLRLDVDGNLCLYSWIEASQSWRSVWQAVEDQCKVFATCGQRGVCVFTASGSTDCRCPFE

VTESNQCLVPYDQECESGSNMLTYKNTYLYGIYPPDDSVVISTLQQCEQLCLNDTQCTVA
TFSNNGRPQCSIKKTKYVTGHADPSLSSISFIKRCSGPFAVNPGLTKSPPPKLPPRLCVP
CLMGALGIIFIIFRRKNSTMQNVALAFTSPNPKGLNVFSFSEIKSLTRDLKDRIGPNMFK
GKKGSSRLRSAVMKLGNIHHKNLVKLEGCCCEFNLRFLVYEYAKNGSLHKYIGDGTLCKR
LTWRKRVEICSSVAKAICYMHTGCREFGSHGNLKCENVMLDENSVAKVCEYGFAIEDGEA
TYCGFSAEKDVGDFGKLVLTLLTGCQDHEQLCEWAYTEWMEGRAVNVVDKRIDGVVNAEE
LERALRISFWWCLQMDERRRPSMEEVVRVLDGTLNVDPPPQPYYETSSVMC*

>Glyma.19G192000|Glyma.19G192000.1|44928945|GNA/S-locus glycoprotein/PAN
MFNMLQTHHLQFLAILFLYTLTCSETATTSIPQELHIGFSVTPESWTTPFQAVLSDHSGN
FSLGFLRVNQNQLALAVLHVASSEPFWVANPSHAPSWSDNTRLFFNGSLVLSDPETLVLW
STATNGDRVVLLNSSNLQVHHNGIPLWESFHFPKNTLVQDQNFTSNMTLLSSNGIYSMRL
GNDFMGLYENHVQLYWKRTPLGAKAEVKEGQGPIYARVNPEGYLGMYQTTDEKPTDVQKF
NTFQQTSSFLFVRLEPDGNLKGYYWDGSTWQLNYQAITEACDLPRSCGSYGLCTPGGSGC
SCLENRTRFEPDGCFKDVGGESSGDLCSSEGIGGSKSSYWVLRRTGVEAPHKELVRHLTT
SSWADCEGLCQNNCSCWGALYSNATGFCYMLDYPIQTMVGTGDGSKVGYFKVKKEERGKN
RVWIRVGVVVTVLVGVGVIIIGTGFCVTRWRKRRGVKEEEWGSPGPYKNLGSASFRFIEM
SNAHDARGDACAQS*

>Glyma.20G164200|Glyma.20G164200.1|40172633|GNA/S-locus glycoprotein/protein kinase
MANADSSLICYFWVILALGLCCFSGCISGQIGLGSRLLAREAQTWVSENGTFALGFTPAE
TDNRLFVIGIWFARLPGDRTLVWSPNRDNPVSQEAALELDTTGNLVLMDGHMTMWTSNTS
GADVQTAIMAETGNFILHNSNNHSVWQSFSQPSDTLLPNQLLTVSSELTSSKSSSQGGYY
SLKMLQQPTSLSLALTYNLPETYQASDESYTNYSYWQGPDISNVTGEVIAVLDQAGSFGI
VYGDSSDGAVYVYKNDGDDAGLSSAVHQSAPLTVLRRLTLEKNGNLRLYRWDEVNGSRQW
VPQWAAVSNPCDIAGVCGNGVCNLDRSKTKATCTCLPGTSKVGRDGQCYENSSLVGNCNG
KHENLTSQFRISAVQQTNYYFSEFSVITNYSDISNVSKCGDACLSDCDCVASVYGLNEER
PFCWVLRSLSFGGFEDTSSTLFVKVRANGSWTSEGQAGGSNSSSDGMGSAKEKAVIIPTV
LSMVVLIVLLSLLLYFSVHRKRTLKREMESSLILSGAPMSFTYRNLQIRTCNFSQLLGTG
GFGSVYKGSLGDGTLVAVKKLDRVLPHGEKEFITEVNTIGSMHHMNLVRLCGYCSEGSHR
LLVYEFMKNGSLDKWIFPSYQGRDRLLDWTTRFNIAIATAQGIAYFHEQCRDRIIHCDIK
PENILVDENFCPKVSDFGLAKLMGREHSHVVTMVRGTRGYLAPEWVSNRPITVKADVYSY
GMLLLEIIGGRRNLDMSFGAEDFFYPGWAYKEMTNGSIIKVADRRLNGAVDEEELTRALK
VAFWCIQDEVSMRPTMGEVVRLLEDSIDINMPPMPQTVVELIEEGLDHVYKAMKREYNQS
SSFTLTSHLTSQATCSNSTMSPR*

>Glyma.20G173600|Glyma.20G173600.1|41121090|GNA/S-locus glycoprotein/protein kinase
MKLQTHQLLLSFVLFSAVIISTISGTTITISPGTTLYASNTTQSWSSPNDTFSLHFLPLH
PPTFPPSFTAAVVHSGGAPAVWSAGNGAAVDSAASFQFLPAGNLVLVNGSGSTVWDSGTS
NMGVSSATLHDNGNLVLSNATSSVWSSFDNPTDTIVSFQNFTVGMVLRSGSFSFSVLSSG
NLTLKWSDSVPYWDQGLNFSMSVMNLSSPVLGVEPKGVLQLFYPNLSAPVVVAYSSDYGE
GSDVLRVLKLDGDGNLRVYSSKRGSGTVSSTWVAVEDQCEVFGYCGHNGVCSYNDSSSSP
ICGCPSQNFEMVNPSDSRKGCRRKVRLEDCVGKVAMLQLDHAQFLTYPPQFLINPEVFFI
GISACSGNCLASNSCFASTSLSDGSGLCYIKTSNFISGYQNPALPSTSYIKVCGPVAPNL
APSLENAHWRLHGWVALVVLSTLLCFLVFQGGLWLWCCRNRQRFGGFAAQYTLLEYASGA
PVHFSYKELQRSTKGFKEKLGDGGFGAVYKGTLFNQTVVAVKQLEGIEQGEKQFRMEVST
ISSTHHLNLVRLIGFCSEGQHRLLVYEFMKNGSLDNFLFVDEEQQSGKLLNWGYRFNIAL
GAAKGLTYLHEECRNCIVHCDVKPENILLDENYNAKVSDFGLAKLLRPVDCRHRTLTSVR
GTRGYLAPEWLANLPITSKSDVYSYGMVLLEIVSGRRNFEVSEETRRRKFSVWAYEEFEK
GNIMGVIDRRLVNQEINLEQVKRVLMACFWCIQEQPSHRPTMSKVVQMLEGVIDIERPPA
PKINSNAAPISTIATSSAPNYSSSSSLFTFEASPLALAIS*

>Glyma.20G246600|Glyma.20G246600.1|47659069|GNA/protein kinase
LPYVSATNVSIGETLVAGNGGKRWLSPSEDFAFGFHQLDNDLYLLAISYQNIPRDSFIWY
ANGDNPAPKGSKLELNQYTGLVLKSPQGVELWTSQLISGTISYGLMNDTGNFQLLDENSQ
VLWDSFSNPTDTLVPTQIMEVKGTLSSRQKEANFSRGRFQFRLLPDGNAVLNPINLPTNY
TYDAHYISATYDSTNTTNSGFQVIFDNSGLATINFDGTFTISNYPKNPASNPSWTVMKTL
PDNICMNLLGNTGGSGVCGFNSICTLKADQRPKCSCPEGYSPLDSRDEYGSCKPNLELGC
GSSGQSLQGDLYFMKEMANTDWPVSDYELYKPYNSEDCKTSCLQDCLCAVSIFRDDSCYK
KKLPLSNGRRDRAVGASAFIKLMKNGVSLSPPNPFIEEKKYKKDQDTLITVISVLLGGSV
FFNLVSAVWVGFYFYYNKKSSTNKTATESNLCSFTFAELVQATDNFKEELGRGSCGIVYK
GTTNLATIAVKKLDKVLKDCDKEFKTEVNVIGQTHHKSLVRLLGYCDEEQHRILVYEFLS
NGTLANFLFGDFKPNWNQRVQIAFGIARGLVYLHEECCTQIIHCDIKPQNILLDEQYNAR
ISDFGLSKLLKINESHTETGIRGTKGYVAPDWFRSAPITTKVDVYSFGVLLLEIICCRRN
VDGEVGNEEKAILTDWAYDCYRAGRIDILLENDDEAIDDTNRLERFVMVAIWCLQEDPSL
RPPMKKVMLMLEGIAPVTIPPSPSPYTSVSVSCG*

>Glyma.U042800|Glyma.U042800.1|49659|GNA/S-locus glycoprotein/protein kinase

MSIIVYILFSPSLIVFIAAETSSITLSQSLSYGKTLVSPSGIFELGFCNLGNPNKIYLGI
WYKNIPLQNIVWVANGGNPIKDSFSILKLDSSGNLVLTHNNTVVWSTSSPEKAQNPVAEL
LDSGNLVIRDENEDKEDTYLWQSFDYPSNTMLSGMKVGWDIKRNLSTCLIAWKSDNDPTQ
GDLSWGITLHPYPDIYMMKGTKKYHRFGPWNGLRFSGMPLMKPNNPIYHYEFVSNQEVVY
YRWSVKQTSSISKVVLNQSTLELPENGQSLYIRLPASEIDKPKKNENIERQLEDLDVPLF
HLLTITTATNNFSLNNKIGQGGFGPVYKGKLVDGREIAVKRLSSSSGQGITEFITEVKLI
AKLQHRNLVRLLGCCFRGQEKLLVYEYMVNGSLDTFIFGHHNESGNPESNIMLDVEITKS
AIR*

Hevein

>Glyma.02G042500|Glyma.02G042500.1|3995112|Hevein/GH19
MKNMKLCSVMLCLSLAFLLGATAEQCGTQAGGALCPNRLCCSKFGWCGDTDSYCGEGCQS
QCKSATPSTPTPTTPSSGGDISRLISSSLFDQMLKYRNDGRCSGHGFYRYDAFIAAAGSF
NGFGTTGDDNTRKKEIAAFLAQTSHETTGGWASAPDGPYAWGYCFINEQNQATYCDGGNW
PCAAGKKYYGRGPIQLTHNYNYGQAGKALGLDLINNPDLVATDATVSFKTALWFWMTAQG
NKPSSHDVITGRWTPSSADSSAGRAPGYGVITNIINGGLECGHGQDNRVQDRIGFYRRYC
QMMGISPGDNLDCNNQRPFA*

>Glyma.12G049200|Glyma.12G049200.1|3533167|Hevein/GH19
MMMMGNNVHSIGVIGVMVSGLVLMMVSKGVSVRAQNCGCEAELCCSKYGYCGSGDDYCGK
GCKEGPCYGTATPNDDVSVADIVTSEFFNAIIDQAEDHCAGKNFYSRDAFLDALIAYDQF
AKTGSVDDSKREIAAAFAHFTYQSRHFCYIEEIEGASKDYCDKTNRHYPCAHNKGYYGRG
PIQLSWNFNYGPAGENNGFDGLNAPETVASDPVISFKTALWYWTQNVSPVMKHGFGATIR
AINGHLECDGANPETVQARVNYYTEYCSQLDVAPGDNLTC*

>Glyma.13G346700|Glyma.13G346700.1|43705650|Hevein/GH19
MIGKKFLCVVVAVAFVMITKVPQNVSAQNCGCAEGLCCSQHGYCGNGEEYCGTGCKQGPC
YSSTPSTNNVNVADIVTPQFFSGIIDQADSGCAGKNFYSRDAFLNALNSYNDFGRLGSQD
DSKREIAAAFAHFTHETGHFCHIEEINGASQDYCDENTISQYPCLSNRGYYGRGPIQLTW
NFNYGPAGQSNDFDGLNAPETVGNDPVISFKTALWYWMQHVRPVINQGFGATIRAINGQL
ECDGANPSTVQARVNYYTDYCRQFGVATGDNLTC*

>Glyma.16G119200|Glyma.16G119200.1|26625538|Hevein/GH19
MGNMKLCPLMLCLLLAFLLGAAAQNCGTQVGGVICPNGLCCSQYGWCGNTEAHCGRGCQS
QCTPGSTPTPTTPSGGDISNTISRSQFEEMLKHRNDAACPGRNFYTYDAFIAAARSFNGF
GTTGDITTRRREIAAFFGQTSHETTGGWASAPDGPYAWGYCFINERNQADYCTSGTRWPC
APGKKYYGRGPIQLTHNYNYGLAGEQLNLNLLNDPDLVSRDPIVAFRTAIWFWMTAQGNK
PSSHSVIIGTWNPSSADWQAGRVPGYGVITNIINGGLECGRGPDSRVQSRIGFYERYCQI
FGVSPGNNLDCNNQRPF*

>Glyma.19G245400|Glyma.19G245400.1|49238744|Hevein/Barwin
MGKAWVGLVVLLCLIVTAIAEQCGRQAGGQTCPNNLCCSQYGWCGNTEEYCSPSKNCQSN
CWGGGGGGGGGGGGESASNVRATYHYYEPEQHGWDLNAVSAYCSTWDASKPYSWRSKYGW
TAFCGPVGPRGRDSCGKCLRVTNTGTGANTIVRIVDQCSNGGLDLDVGVFNRIDTDGRGY
QQGHLIVNYQFVDCGNELDLTKPLLSILDAP*

>Glyma.19G245500|Glyma.19G245500.1|49241284|Hevein/Barwin
MEKVGVRLVVLLCFIVTAAMAQNCGRQAGGQTCGNNLCCSQYGWCGNSEDHCSPSKNCQS
TCWGSGGGGGGGESASNVRATYHNYLPEQHGWDLNAVSAYCSTWDAAKPYSWRSKYGWTA
FCGPVGPRGRDACGKCLRVTNTGTGANIIVRIVDQCSNGGLDLDVGVFNRIDTDGRGYQQ
GHLIVNYQFVNCGD*

Jacalin

>Glyma.02G227500|Glyma.02G227500.1|41437909|Jacalin/Jacalin/Jacalin
MSFEDSSKKHQSVGPWGGNGGSRWDDGIYSGVRQLVIVHGTGIDSIQIEYDKKGSSIWSE
KHGGSGGRKTDKVKLDCPNEFLTKIHGYYGSSNQRGPNFVRSLSFESNKKTYGPFGVELG
TYFSVPLTGAKIVGFHGRCGWYVDAIGVYLKSSKQPNPSKTLAHSQSSITNISENFGYSV
IQGTLNENYDIVLALKQKDDFNKPSAKNVSGKISFVKESNNIEHKEKMAHVEKSSPKVGD
VVTHGPWGGIGGYVFDDGTYTGIRQINLSRNVGIVWIRVFYDYHGETIWGSKQGGTGGYK
NDKIVFDFPYEALTHISGYYGPLMYMGPSVIRSLTFHTTKRKYGPYGEEQGTYFTTKVKE
GKIVGIHGRKGLFLDAFGVHVVEGKVIVPVATPPKEITSRESSIGEIGSAQWPTKLVLAK
PSAAEEVSCGVVKEPAPCGPGPWGGDGGRSWDDGVFSGIKQIYLTKVSEGICSIQIEYDR
NRQSVWSVKHGGSGGDTMHRIQLEYPNEVLTCISGYYGPIAKDEQHIIMKSLTFNTSRGS
MVHLVKK*

>Glyma.11G173800|Glyma.11G173800.1|18905184|Jacalin/Jacalin/Jacalin

MSFEEKPVSVGPWGGNGGYRWDDGVYSTVRQLVIVHGEGIDSIQIEYDKQGSSIWSLKYG
GSGGYKIDKIKLDYPEEFLTSVDGYYGSLNQWGPIFIRSLSFESNKKLYGPFGVEQGTYF
SLPMTGGKIIGFHGRYGWHLDAIGINVKSSQQQKPSKALSFSQNYMTNTNDNASYSVIQG
SVGQGYDIVLALKQKDDFGKSPTVKISSFKEPNNVEPKEKVVFVEKVPPMVEGVVTYGPW
GGTGGYVFDDGPYTGVRQIDMSRNVGIVWIRALYDLDGEPVWGYKHGGAGGFKHEKIIFD
FPYEVLTHISGYYGSLMYMGPAVIRSLTFHTTKRPYGPFGDEYGTYFTTKLREGKVVGIH
GRSGLFLDSLGVHAIEGKVIVPVATAPSMEIISKEPNISEIDNPQRPVVAKPAPIEQASR
CVIKEPAPCGPGPWGGDGGRPWDDGVFSGIKQIYMTKAPEGICSIQIEYDRYKQSVWSVK
HGGNGGNTMHRIKLEYPNEVLSCISGYYGSITADEQPIIIKSLTFHTSRGQYGPFGDEVG
KYFTSTTTEGKVVGLHGRSSMYLDAIGVHMQHWLGGIQKTSKLSFFKLF*

>Glyma.13G352300|Glyma.13G352300.1|44119294|Jacalin
MEGKSRKKNVIVGPWGGNGGNSWDDGIFTGVREIKLVYGHCIDSIQVVYDRNGKPFTAEK
HGGVGGNKTAEIKLQFPDEFLVSVSGHYCPVVRGGTPVIRSLTFKSNHRTFGPYGVEEGT
LFTFSIDGGCVVGFKGRGDWYLDAIAFTLCNTRSKSLFQKVQRSLLWLTTTAPKSTSSKD
G*

>Glyma.15G022500|Glyma.15G022500.1|1756942|F-box/Jacalin
MISGSKRKRSLGDNLDCVREAARLKETPGEKDSDEMDTSDDGSVDRISQFPDHVIHHILS
HLRNVNDAIRTSVLSKRWRELWYSYSVLIFDERKFAAKIGHEDSSNKGMMFRDYVSNSLL
TSNAKNLQIRKLVLHMTSFDLLEDAPCLELWLNIAIYRNIKELDLHVGIKNGECYTLPQT
VFSSKTLTGIRLSGCKLGTCNNIKLPYLQKLYLRKIPLVENFIQNLISCCHSVEDLRIIK
CSGLKHLHVSNLIRLKRAEIHHCIQLKKVEISAPNLDTFWYCGKKTSPCKVSLEGCTSLK
RLTLEHPQVTRDFCENQFSNFPLLEKLDLSMSNNKSRFIIISNPHLEKFTLKGCKKLGIV
LVEAPNLLSFECKGETMPWVEIHPFGLTQAKLSFVPKSEPRVVGYGDKIWIRMKSFIQKF
NRERFKLVLYSNKSIVIHEDLNNVILPPVPDLGCEIINSSACIDDALNSLLRKLHPVTLS
IISPTDSKFPKLVYEMMKNKDKDPICCIYSTSKNKCWRHLLKDVNFEDLNDVKFEDIEEG
KSRKRSIILGPWGGNGGNSWDDGTFTGVREIKLVYGHCIDSIQVVYDRNGKPLTAKKHGG
VGGNKTAEIKLQFPDEFLVSVSGHYCPVVRGGTPVILSLTFKSNRKTFGPYGVEEGTPFT
FSIDGGCVVGFKGRSDWYLDAIAFTLCNTRSKSLLQKVQRGLFWLTSTAPKSSSSKDG*

>Glyma.18G061800|Glyma.18G061800.1|5612390|Jacalin/Jacalin/Jacalin
MSFEEKPVSVGPWGGNGGYHWDDGVYSTVRQLVIVHGEGIDSIQIEYDKQGSSIWSLKYG
GSGGYKIDKIKLDYPDEFLTSIDGYYGSLSQWGPIFIRSLSFESNKKLYGPFGVEQGTYF
SLPMTGGKIVGFHGRYGWHLDAIGVNLKSSQQPKPSKALSYSQNNMTNTTDNGRYSVIQG
SVGQDYDIVLALKQKDDFGKSPTVKISSFKEPNNIEPKEKIVFVEKAPSMVEGVVTYGPW
GGTGGYVFDDGPYTGVRQIDLSRNVGIVWIRALYDLDGEPVWGYKHGGAGGFKHEKIIFD
FPYEVLTHISGYYGSLMYMGPAVIRSLTFHTTKRSYGPFGDEYGTYFTTKLREGKVVGIH
GRSGLFLDSLGVHAIEGKVIVPVATSPSMEIISREPSISEIDNPQWLVVAKPAPVEQASR
CVIKEPAPCGPGPWGGDGGRPWDDGVFSGIKQIYLTKAPEGICSIQIEYDRYKQSVWSVK
HGGNGGNTMHRIKLEYPNEVLTCISGYYGSVTADEQPIIIKSLTFHTSRGQYGPFGDEVG
KYFTSTTTEGKVVGLHGRSSMYLDAIGVHMQHWLGGIQKTSKLSFFKLF*

Legume

>Glyma.01G020600|Glyma.01G020600.1|2067151|Legume
MASHAAQNPKSVFLMTFLLLITSAKSDSFSFNLPRFEPDALNILLDGSAKTTGGVLQLTK
KDKRGNPTQHSVGLSAFYAALHLSDAKTGRVANFATEFSFVVNTKGAPLHGDGFTFYLAS
LDFDFPDNSSGGFLGLFNKKTAFNTSLNQVVAVEFDSFANEWDPNFPESDSPHIGIDINS
IRSVATAPWPLDIQPQGSIGKARISYQSSTKILSVSVAYPNSPVKLNATVLSYPVNLGAV
LPERVLFGFSAATGDLVETHDILSWSFNSFL*

>Glyma.01G020700|Glyma.01G020700.1|2069065|Legume
MAFPNSKPNLLQSLSPLIKFFIPFLLLLQHHSVKSQQQPPSPMSAYETVGIDFSFFDKDD
PNVLLIGNASVSGGALRLTNTDQLGKPVPHSVGRVVHITPIHLWNKNNGHLADFTSDFSF
VVNPKGSALRGDGFAFFLTSANLNFLIPKNSSGGYLGLFKPETALDPSKNQIVAIEFDSF
TNDWDPNSPNQSPHVGIDVDSIKSVATVPWPSELEPDNAVAHASLNYNSEDKRLSVFVGY
PDNRNATVSAIVDLRNVLPEWISVGFSASTGDLVETHDILNWSFEAAL*

>Glyma.01G099900|Glyma.01G099900.1|33275414|Legume/protein kinase
MTLRGVAEMESNGIVKLTDDSSRFMKNGMLHHAFTIASFENLKALPSPYLGLLNSSGNGN
SSNHLFAIEFDTTQDFELGDTDDNHLSRPKKKHTSVIIGVSVSGLLALCAILFGIYIYSY
QELKKTTKGFKDKELLGQGGFGSVYKGTLPNSNTQLLLKRISHDSKQGLRKFVSEIASIG
PLHHWNLVRLLGWCLRRGDLLLVYDFMENGSLDKHLFDEPETILSWEQRFKVIKDVASAL
LYLHEGYEPVVIHRDVKANNVLLDGDFGLARLYEHGANPSTTRVVGTFGYIAPEVPRTGK
STPSSDVFAFGALLLEVACGLRPVDPKAMPKDVVLVDCVWDKYRQGRILYVVDPKLNGAF
NERVEVLMVLKLGILCSNGAPTFRPSMRQVVRFLEGEVGLPDELRKPGEVGYQEGFDEFL
NSLEPSSFYHMSTSSYDIKTDMDARFPFIDTSLYTLHARGETR*

>Glyma.01G101000|Glyma.01G101000.1|33518924|Legume/protein kinase
MATKLNMLLKLLPLLVFLLIPVSSQPNQLFYAGFKGLGSNNMTLDGVAEIEPNGVLKLTN
DSSKVMGHAFYPTPFRFKNSSGGKAFSFSSSFALAIVPEFPKLGGHGLAFTIAPSKDLKA
HPSQYLGILDSSNIGNFSNHLFAVEFDTAKDFEFGDIDDNHVGIDINSLASNASASAGYY
TGDDDSSKQNLTLQSRVPILAWVDYDAAKSVVHVTISASSTKPKRPLLSYHVDLSPILKE
SMYVGFSASTGLLASSHYILGWSFKINGPAPPLDLSSLPQLPGPKKKHTSLIIGVSVSVV
VLALCAVLFGIYMYRRYKNADVIEAWELEIGPHRYSYQELKKATKGFKDKELLGQGGFGS
VYKGTLPNSNTQVAVKRISHDSNQGLREFVSEIASIGRLRHRNLVQLLGWCRRLGDLLLV
YDFMENGSLDKYLFNEPETILSWEQRFKVIKDVASALLYLHEGYEQVVIHRDVKASNVLL
DGELNGRLGDFGLARLYEHGTNPSTTRVVGTLGYLAPEVPRTGKATPSSDVFAFGALLLE
VACGLRPLEPKAMPEDMVLVDCVWNKFKQGRILNMVDPKLNGVFNEREMLMVLKLGLLCS
NGSPTARPSMRQVVRFLEGEVGVPDELRKPGEGGYQEGFDEFLHSLESSSFDQMNTGSYG
RNRDMDSSFPSLTGTSLFSPHGKGQTM*

>Glyma.01G109200|Glyma.01G109200.1|37229455|Legume
MAPFPTSHYYFRAFTFSILFLKTLAFDPIPLFYYAGFGKDLKFTPNVALFGNAKVLNEGS
GIHFSGSGSSGDTGRVMYKKPIKLFQGKPRQLVSFSTYFAFSVSLEDGGGLAFVMAPKGS
EGDMFYQSSYGLNSRKFEVVGVEFSASKGGRKGVSSSCHVNMNIGGFVAKKSNTSIGEKL
HVWIDYEASSKRLEVRLSQHGKSRPSYPLLWQSIDLSNVLKEKEMLVGFSSVKGNDSQAC
FLYSWSFVLRNFPHSMHSEPLDPKVFVKNTESPVVKQRSDCFLRVLAAMIFGTGCGALTA
FIVLYLWTIFGNNKRAVVPEESVVQPVDVEYRKNVRIVVDKTIEDGKK*

>Glyma.01G155600|Glyma.01G155600.1|49302837|Legume/protein kinase
MFPLQCSNHPMCAFSAAVTTILLLFPAATSQAQILKKETYFFGPFNQSYFTTFAVLPSAA
INLGALQVTPDSTGNVSLANQSGRIFFSTPFTLWDDENLNGKLVSFNTSFLINVFRPQNN
PPGEGIAFLIAPSSSTVPNNSHGQFLGLTNAATDGNATNKFIAVELDTVKQDFDPDDNHI
GLDINSVRSNVSVSLTPLGFEIAPNVTRFHVLWVDYDGDRKEIDVYIAEQPDKDVPIVAK
PAKPVLSSPLDLKQVLNKVSYFGFSASTGDNVELNCVLRWNITIEVFPKKNGNGKAYKIG
LSVGLTLLVLIVAGVVGFRVYWIRKKKRENESQILGTLKSLPGTPREFRYQELKKATNNF
DDKHKLGQGGYGVVYRGTLLPKENLQVAVKMFSRDKMKSTDDFLAELTIINRLRHKNLVR
LLGWCHRNGVLLLVYDYMPNGSLDNHIFCEEGSSTTPLSWPLRYKIITGVASALNYLHNE
YDQKVVHRDLKASNIMLDSNFNARLGDFGLARALENDKTSYAEMEGVHGTMGYIAPECFH
TGRATRESDVYGFGAVLLEVVCGQRPWTKNEGYECLVDWVWHLHREQRILDAVNPRLGND
CVVEEAERVLKLGLACSHPIASERPKMQTIVQILSGSVHVPHLPPFKPAFVWPAMDLSSL
ASDLTTQTTTTEYTPMSSDTHSMHVQFSDSSSLV*

>Glyma.02G012600|Glyma.02G012600.1|1123507|Legume
MATSKLKTQNVVVSLSLTLTLVLVLLTSKANSAETVSFSWNKFVPKQPNMILQGDAIVTS
SGKLQLNKVDENGTPKPSSLGRALYSTPIHIWDKETGSVASFAASFNFTFYAPDTKRLAD
GLAFFLAPIDTKPQTHAGYLGLFNENESGDQVVAVEFDTFRNSWDPPNPHIGINVNSIRS
IKTTSWDLANNKVAKVLITYDASTSLLVASLVYPSQRTSNILSDVVDLKTSLPEWVRIGF
SAATGLDIPGESHDVLSWSFASNLPHASSNIDPLDLTSFVLHEAI*

>Glyma.02G042900|Glyma.02G042900.1|4015752|Legume/protein kinase
MLATTEHFHYLKTCLLLSIIFMILRIVQPLSFNITNFSNPESASRIQYTGVAKIENGSIV
LNPLINNGVGRAIYGQPLRLKNSSKGNVTDFSTRFSFTIDARNKTNYGDGLAFYMAPLAF
DYQTPPNSSDGFRLGLYGGSQDNIVAVEFDTCVNEFDPPMQHVGINNNSVASLEYKKFDI
ESNIGKMGHALITYNASAKLLAVSWFFEGTSSGFTPNDSLSHQIDLMEILPKWVTVGFSG
ATGSSKEENVIHSWEFSPNLDLNSTNQEANNENFIIITKYKVQVKVVVVAVICSNIVVLV
VISIITWLIIKKRRTEDGFDLDKLAFMPRRFGYKELVAATNGFADDRRLGEGGYGQVYKG
FLSDLGRVVAVKRIFSDVEDSEEIFANEVKIISRLIHRNLVQFIGWCHERGESLLVFEYM
TNGSLDTHIFGDNSRRTLTWGVRYKIALGVARALRYLHEDAEQCVLHRDIKSANVLLDAD
FNTKISDFGIAKLVDPRLRTQKTRVVGTYGYLAPEYINQGRVSKESDMYGFGVVVLEIAS
GRKTYNHDVPLVNRVWKHYVEGNILNVADKDLKMDFDAVEMTCLLTVGLWCTLQDHKKRP
KAEQVINVLKQEVPLPNIDRV*

>Glyma.02G043000|Glyma.02G043000.1|4018144|Legume/protein kinase
MAATLPHTRRHDQGLESLLRTTSAPVGLIFQLTVSPAPRGYRIPPNPAVGTLGLFNATTN
VYIPNNHVHAVEFDTFNGTIDPPFQHVGIDDNSLKSVAVAEFDIDRNLGNKCNALINYTA
SSKTLFVSWSFNNSNSNTSLSYKIDLMDILPEWVDVGFSAATGQYTQRNVIHSWEFSSST
ASKKHNNNVLLIVVVTCSTVLVVVVVAVSVAVWAMITKKRKATQVKFDLDRATLPRRFDY
KELVVATKGFADDVRLGRGSSGQVYKGFLSGLGRVVAVKRIFTNFENSERVFINEVRIIS
RLILMHRNLVQFIGWCHEQGEFLLVFEFMPNGSLDTHLFGEKKTLAWDIRYKVALGVVLA
FRYHHEDAEQSVLHRDIKSANVLLDMDFSTKLGDFGMAKMEGPRLRTQRTGVVGTYGKES
DIYSFGVVALEIACGRRIYQDGEFHVPLVNWVWQLYVEGNVLGVVDERLNNEFDVDEITS
MIVVGLWCTNPNDKERPKAAQVIKVLQLEAPLPVLPLDMHNASHPSLVTDAQSTYNSSYS
VPFTNSFVSVGR*

>Glyma.02G156800|Glyma.02G156800.1|16787594|Legume
MKVLCIIFEFKQIKAMATSNFSIVLSVSLAFFLVLLTKAHSTDTVSFTFNKFNPVQPNIM

LQKDASISSSGVLQLTKVGSNGVPTSGSLGRALYAAPIQIWDSETGKVASWATSFKFNIF
APNKSNSADGLAFFLAPVGSQPQSDDGFLGLFNSPLKDKSLQTVAIEFDTFSNKKWDPAN
RHIGIDVNSIKSVKTASWGLSNGQVAEILVTYNAATSLLVASLIHPSKKTSYILSDTVNL
KSNLPEWVSVGFSATTGLHEGSVETHDVISWSFASKLSDGSSNDALDLPSFVLNEAI*

>Glyma.02G172200|Glyma.02G172200.1|27235922|Legume/protein kinase
MHLPWLVKYQLASTIFIIITLTKVTCFYFNFPTFQKDNESELLLSKNSQIYFDAIQVTPD
IRGPIQDYSGRAFYKKPYKLWNKKKNQIASFNTTFVLNIKPETTPGGEGLAFILTSDTTL
PQNSSGEWLGIVNATSNGTSQAGILAVEFDTRKSFSQDGPDNHVGVNINSINSIQQVPLI
NTGVNVSSGINVTFKIQYLNDTITVFGSMTGFEESMETLLVSPPLNLSSYLHEVVYLGFS
ASTSNYTELNCVRSWEFSGVDIADDDNKSLLWVYITVPIVIVIVIIGGMVIFLLCWQRKR
HMERPEDAYPRIEDQIQYSSMAPKKFKLREITKATGGFSPQNKLGEGGFGTVYKGLLENK
EVAVKRVSKNSRQGKQEFVAEVTTIGSLHHRNLVKLTGWCYEKRELLLVYEFMPKGSLDK
YLFGDKNFGNNTLEEGYSLTLNWETRHSVIHGVAQALDYLHNGCEKRVLHRDIKASNIML
DSDYNAKLGDFGLARTIQQRNETHHSTKEIAGTPGYMAPETFLTGRATVETDVYAFGVLV
LEVVCGRRPGSVYAQDDYKNSIVYWVWDLYGKGKVVGAVDAKLKKEEIKEEEVECVLVLG
LACCHPNPHHRPSMRTVLQVLNGEATPPEVPKERPVFMWPAMPPSFKEAEDSSLVQGTLA
PFTEITGR*

>Glyma.02G221900|Glyma.02G221900.1|40932876|Legume/protein kinase
MIGISFVLSHAAMASLSNILLMFCLLNLLAFPVSVHSAGEVPINVTKHFSFYNFSFSNNP
RLVHDMKLLGSAKFSNEKGALQIPNESEEDIRHQAGRGIYSFPIRLLDPSTKTPASFQTT
FSFQMNNSTASEQAAYGGSGLTFIIVPDEFTVGRPGPWLGMLNDACENDYKAVAVEFDTR
KNPEFGDLNDNHVGINLGTIVSTKVINVSDVGLSLNDGSVHRAWITYDGPQRRMDIRLGR
ANQEDYDYPPKPLFSESMDLSPFLNEYMFVGFSASTGNHTQIHNILSWNFTSTSQAFLRL
PSSETCQGKILLENSTASTEVPPTSHKSSKNEPPRSFLIFVAAVALALALFLGFYFISKH
RRNAAKLNTSVEAELHMPRPPNKPRRFAFSQLSSSTRSFSEIELLGSDNRGEYYRGKLSN
GSQVAVKRFSAQFLSTHGSDKKRLLKEIKGVSHVRHPNLLLVRGWCQDNHEIMVVYDFVP
NGSLDKWLFGAGVLPWTRRFKVIKDVADGLSFLHTKQLAHKNLKCSSVFLDVNFRAVLGD
FGFVLMGAESKHFESQVCQGADVFEFGVLVLEVIAGRVRDEKEEGNPEERNLLDYAWNLH
QIDEKVKLVDRRMGSLINLEQAIRVLEIGLLCTLNENKGRPSMEQVVEFLLNMDKPIPEL
PRTRPVALFPYNSANTGLCNAYSCTF*

>Glyma.02G241500|Glyma.02G241500.1|42993453|Legume/protein kinase
MLLSIFCFFNSVTAATEFDFGTLTLGSLKLLGDAHLNNNTVSLTRDLAVPTSSAGRALYS
RPVRFRQPGNRFSASFTTFFSFSVTNLNPSSIGGGLAFVLSPDDDTIGDAGGFLGLGGGG
GFIAVEFDTLMDVEFKDINGNHVGVDLNSVVSSEVGDLANVGVDLKSGDLINAWIEFDGS
SKGLSVWVSYSNLKPKDPVLTMNLDVDKYLNDFMYVGFSASTQGSTEIHRIEWWSFGSSF
AAAEAAAPPPPPASAPPPSLAPSHSEEKESISKSSCHNGCVKRVNKFDSLGSEIIKMPKQ
FSYKELKSATKCFNANRIIGHGAFGTVYKGVLPENGDIVAVKRCSHSSQGKNEFLSELSI
IGSLRHRNLVRLQGWCHEKGEILLVYDLMPNGSLDKALFEARTPLPWAHRRKILLGVASA
LAYLHQECENQVIHRDIKTSNIMLDEGFNARLGDFGLARQTEHDKSPDATVAAGTMGYLA
PEYLLTGKATEKTDVFSYGAVVLEVASGRRPIEKDANGGGKGGISCNLVESVWSLHREGR
LLMAADPRLGGEFDDGEMRRVLLVGLACSHPDPLTRPTMRGVVQMLVGEAEVPLVPRTKP
STGFSTSHSHLLLSLQDSVSDCDGIITISTSTSENSFNLGDIV*

>Glyma.03G051100|Glyma.03G051100.1|6684296|Legume/protein kinase
MAPKNLFGLLIFLILLVTCTAFSFQFHGFHNSERNLTREGDSNVTPQGILQLTKRENNIV
GHAFYNKPIKILEKTNSSVPQTKFSSFSTCFVFSIVSPNSGLGGFGLAFTIAPTTQFPEA
EGGHFLGLFNNSNDMNTSNHILVVEFDTVNGYKDNTDTVGNHVGVNINGMQSKIAEPAAY
FEEGMDAKKEEFSMEKEDAVCAWIEYDGETEILNVTIAPLKVSKPSKPLISQAIHDIKFV
MKETMFFGFSASTGKRKASSHYILGWSVSVNGGIAPPLNFSLLPKPPPKEKDASSFPWVK
VAVAMLSALTFTLLCLLFIVTRYKRYMMFETLEDWELDCPHRFRYRDLHIATKGFIESQL
IGVGGFGAVYKGVLPSTGTEVAVKRIMRSPMQGMREFAAEIESLGRLRHKNLVNLQGWCK
HKNDLILIYDYIPNGSLDSLLFNDNIALDWDQRFNIIKGVAAGLLYLHEEWEQVVIHRDV
KSSNILIDGEFNARLGDFGLARLYSHDQVSHTTSVVGTIGYIAPELTRTGKASASSDVYA
FGVLLLEVVAGTRPVGSSGQFLLVDWVLENCQLGQILEVVDPKLGSAYDEEEMELVLKLG
LLCSQYKAEYRPSMKQVARYLNFDDSLPDISDWRYYDSQSSTNSLSFLEAMSTGKIASSY
SLSSIGSRSTLPIKTGR*

>Glyma.03G068600|Glyma.03G068600.1|14238074|Legume/protein kinase
MAINILNPQKAQASPSNLSIFEVLDFPHLLMATNLNMLLKFLSLLVFLLIPVSSQQNQLF
YAGFKGLKSNNMTLDGVAEIEPNGVLKLTNDSSKVMGHAFYPTPFRFKNSSGGGNKAFSF
SSSFALAIVPEFPKLGGHGLAFAIAPTKELKAHPSQYLGLLDSTGIGNFSNHLFAVEFDT
AKDFEFGDIDDNHVGIDINSLSSIASASAGYYSGDEDSTKQNVTLQSGVPILAWVDYDAA
QSVVHVTISASSTKPKRPLLSYHVDLSPIFEDLMYVGFSASTGMLASSHYILGWSFKING
PALPLDLSSLPQLPGPKKKHTSLIIGVSASVVFLVLCAVLLGIYMYRRYKNADVIEAWEL
EIGPHRYSYQELKKATKGFKDKGLLGQGGFGSVYKGTLPNSNTQVAVKRISHDSNQGLRE
FVSEIASIGRLRHRNLVQLLGWCRRRGDLLLVYDFMENGSLDKYLFDEPEIVLSWEQRFK
VIKDVASALLYLHEGYEQVVIHRDVKASNVLLDGELNGRLGDFGLARLYEHGTNPSTTRV

VGTLGYLAPEVPRTGKATPSSDVFAFGALLLEVACGLRPLEPKAMPEDMVLVDCVWNKFK
QGSILDLVDPKLNGVFNEREMLMVLKLGLLCSNSSPTARPSMRQVVRFLEGEVGVPDELK
KPGEGGYQEGFDEFLHSLESSSFDQMSTGSYGRTRDMDSSFPSLTDTSLFSPHGKGQTM*

>Glyma.03G069300|Glyma.03G069300.1|14502541|Legume/protein kinase
MANLNMLLQQFLHVLLFLLIPVSSQPNQLFYDGFLGSNIMSLRGVAEIESNGILKLTDDS
SRVVGQAFYPTGLRFKNSSDGYYTGGDDNSTKQNLTLTSGEPIIAWVDYDASQSVVNVTI
SESSTKPKRPLLSHHVDLSPIFEDLMFVGFSASTGLLASSHYILGWSFKINGPAPPLELS
SLPQLPGPKKKHTSLITGVSISGFLALCGFLFGIYMYRRYKNADVIEAWELEIGPHRYSY
QELKKATKGFKDKELLGQGGFGSVYKGTLPNSNTQVAVKRISHDSKQGLREFVSEIASIG
RLRHRNLVPLLGWCRRRGDLLLVYDFMENGSLDKYLFDGPKTILSWEQRFKVIKDVASAL
LYLHEGYEQVVIHRDVKASNVLLDGGLNGRLGDFGLARLYEHGANPSTTRVVGTFGYMAP
EVPRTGKSTPNSDVFAFGALLLEVACGLRPLEPKALPEDVVLVDCVWNKYKQGRILDLVD
PKLNGAFNEREVLMVLKLGILCSNAAPAARPSMRQVVRFLDGEVGLPDELRKPEEVGYQE
GFDEFMNSLEPSSFYQMSTSSYAIKTDMDARFPFIDTSLYTLHARGETR*

>Glyma.03G074300|Glyma.03G074300.1|18511826|Legume
MAPFPTSHYFRAFTFSILFLKTLAFDPIPLFSYAGFGKDLKFKPNVALFGNAKVLNEGSG
IHFSGFGSSRGTGRIMYKKPIKLSQGKPRQLVSFSTYFAFSVSLEDGGGLAFVMAPKGSQ
GNVFYQSSSSGLNDGKFEVVGVEFSASKSGRKGVSSSCDVNMNIGGSVVAKKSNTSIGEK
LHVWIDYEASSRRLEVRLSQHGKARPSYPLMWHSIDLSNVLKENEMFVGFSSVKGNNDSQ
ACDLYSWSFVLRNFPHSMHSEPLDPKVFVKNTESPVAKQRSDCFLSVLAAMIFGTGCGAL
TAFIVLYLWTIFGNNKRAVVPEESLVEPVDVDYRKKVKIVVDKTIEDGKK*

>Glyma.05G041300|Glyma.05G041300.1|3710746|Legume/protein kinase
MSPSNSPVAAETLFSGTVFLILLHLSLFLTPTLSLDFLFNSFAGVTNLTLIKDARVDASV
IRMNNDSNQYSYGRAFYPIKIPMTKTNSSISSFSTSFVFSILPQISTSPGFGLAFVLCNT
TNPPGALASQYFGLFTNATSPSVFPLVAVEFDTGRNPEFNDIDDNHIGIDLNNIESINAT
TAGYFNSSGAFVPVRMRTGQNIHAWIDFNGENLEFNVTVAPVGVSRPTKPSLSYQNPAIA
DYVSADMYVGFSASKTNWIEAQRVLAWSFSDSGPAKELNTTNLPVFQLESSSSSISGGAI
AGIVVGCFVFVLICASGFYLWWRMNKAKEEEDEIEDWELEYWPHRFSYEELSSATGEFRK
EMLLGSGGFGRVYRGTLPNHTQIAVKCVNHDSKQGLREFMAEISSMGRLQHKNLVQMRGW
CRKGNELMLVYDYMPNGSLNKWVFDKSEKLLGWEQRRRILVDVAEGLNYLHHGWDQVVIH
RDIKSSNILLDADMRGRLGDFGLAKLYTHGEVPNTTRVVGTLGYLAPELATVAAPTSASD
VYSFGVVLLEVACGRRPIETSVAEEEVVLIDWVRELYAKGCAREAADAWIRGEYDEGDVE
MVLKLGLACCHPDPQRRPTMKEVVALLLGEEPQEAPGKVLSDLVRGGKDMDETAPLQPSP
PPV*

>Glyma.06G292100|Glyma.06G292100.1|48095366|Legume/protein kinase
MSTSTFLISLLVFITNLTLLLLLTSVSCTEFIYNTNFNSTNTLLHGNATIESSILTLTNS
STFSVGRAFYPFKIPTKPSNSSTPLPFSASFIFSIAPFKDLLPGHGFVFILTPSAGTTGV
NSAQHLGLFNYTNNGDPNNHVFGVEFDVFDNQEFNDINDNHVGVDINSLSSFASHDAGFW
GGSDNDEFEDLKLNDGENYQVWIEYLDSRVNVTMAPAGQKRPQRPLISEIVDLSEVLLDE
MFVGFCGATGQLVESHKILAWSFSNSNFSIGDALVTTNLPSFVLSKESILRSTGFIVGII
VGVLFVLSAAVVIFVFFLRRKRSKRKDEEIEDWELEYWPHRVSYEDIYSATKGFSDQHVI
GFGGNGKVYKGLLQGVQVAVKRIPCDSEHGMREFLSEISSLGRLKHRNVVPMRGWCKKDR
SLILIYDYMDNGSLDKRIFDDDENTIFGWEKRIKVLKDVAHGVLYLHEGWEVKVLHRDIK
SSNVLLDKGMNARLGDFGLARMHNHEQIAHTSQVIGTVGFMAPELIHTGRASTQTDVFSF
GVLILEVVCGRRPNEENKPLVAWLWRLKQRGEECSALDERLKKRGECNIDEVKRVLHLGL
LCTHHDPHVRPSMREVVKEGR*

>Glyma.07G135400|Glyma.07G135400.1|16041248|Legume/protein kinase
MSLKVVTVVFLLATIVVASDYTSFTYNGFQSSHLYLDGSAEFTTNGMVKLTNHTKQQKGH
AFFPSPIVFKNTTNGSVFSFSTTFVFAIRSEFPNLSGHGIAFVVSPTKEVPHSLPSQYLG
LFDDTNNGNNSNHVFGVELDTILNTEFGDINDNHVGIDVNELKSVKSASAGYYSDGGFKN
LSLISGYPMQVWVEYDGLKKQIDVTLAPINVGKPERPLLSLNKDLSRILNSSMYVGFTSS
TGSILSSHYVLGWSFKVNGKAQQLAISELPMLPRLVGKQESKVLIVGLPLILLILILMVA
LAVVHAIKRKKFVELLEDWEQDYGPHRFKYKDLSLATKGFREKELLGSGGFGRVYKGVMP
ISKIEVAVKKVSHESRQGMREFVAEIASIGRLRHRNLVPLLGYCRRKGELLLVYDYMPNG
SLDKYLYNKPRVTLNWSQRFRITKGVASGLFYLHEEWEQVVLHRDIKASNVLLDAELNGR
LGDFGLSRLYEHGTDPHTTHVVGTLGYLAPEHTRTGKATTSSDVFAFGAFMLEVVCGRRP
IEQGRESGSEILVDWVYNCWKKGEILEARDPNLGANYRPDEVELVLKLALLCSHSEPLAR
PSMRQVVQYLEKDVPLPDLSMLSLSSIGLTFGLHEDFQDCPMSYPSSMDRPISHTSSIAE
SLLSGGR*

>Glyma.07G135600|Glyma.07G135600.1|16050761|Legume/protein kinase
MATLLKILSLLVLLLIPVSCQVDQVLYSGFKDVGASNLTMNGVTTIERNGILKLTNESSR
SIGHAFYPSPFQLKNSTSGKALSFSSSFAFAIVPEYPKLGGHGLAFTIATSKDLKALPNQ
YLGLLNSSDNGNFSNHIFAVEFDTVQDFEFGDINDNHVGIDINSMQSNTSANVSLVGLTL
KSGKPILAWVDYDSRLNLISVALSPNSSKPKTPLLTFNVDLSPVFHDTMYVGFSASTGLL

ASSHYILGWSFKINGPAPPLDLSSLPQLPKPKKKQTSLIIGVSVSVVVIVLLAISIGIYF
YRKIKNADVIEAWELEIGPHRYSYQELKKATRGFKDKELLGQGGFGRVYKGTLPNSKIQV
AVKRVSHESKQGLREFVSEIASIGRLRHRNLVQLLGWCRRQGDLLLVYDFMANGSLDKYL
FDEPKIILNWEHRFKIIKGVASALMYLHEGYEQVVIHRDVKASNVLLDFELNGRLGDFGL
ARLYEHGANPSTTRVVGTLGYLAPELPRTGKATTSSDVFAFGALLLEVVCGRRPIEPKAL
PEEMVLVDWVWEKYKQGRILDVVDPKLNGHFDEKEVMVVLKLGLMCSNDVPAARPSMRQV
VRYLDGEVEVPEDLKKPGAVSHHEGFEEFLHSLASSSFDKMSSGSNFGNRDMDSSFLSFS
NSPHSLLHGSGTN*

>Glyma.07G143000|Glyma.07G143000.1|17040669|Legume
MPTSIMAPFSTSHHFTAFTFLILFLKTQAFDPLSSFSFTDFQKDPNFKSSVGLYGNAKVV
YNGSEVLLSGNGGGRVMYKKPFKLVHGEARELVSFSTYFGFSMSLDGEKNGLAFVMVPSG
IEGEVFGNSSYGFSFGLKEREFKVIGVQFSAYGRNGGSGSCIVSINVGSSVPVKTINASS
VIMGLKSEGKLHAWIDYEASSKRLEVRLNQFGQSRPVDPLLWHSMDLSNVWGTEEMFAGF
STVKGNNTSQSCFLYSWSFIVRHFPHWMHSEPLDPKFLAKKTETPTVKYYRSFCLLRVLA
AMIFGAGCGALTAFIVLYLWTIFGNKRPVVPEEYAMQPVDFDYKKVNIVVDKTT*

>Glyma.07G154100|Glyma.07G154100.1|18942248|Legume/protein kinase
MASPTLVFVFLLFLSLLPQVIASKAVIFEGFDDENSELNLEGSSIIKTSRLLKLTNRSTN
IVGHAFYATPFQMLNNTNQPYAYSFSTNFVFSIVSPSSGSGGFGLAFTIAPSTQFPGAEA
GHYLGLVNSTNDGNESNHIFAVEFDTMNGYKDDSDTEGNHVGVNINGMDSNITEPAAYIK
EGTDKVKEDFRMAKVDAVQAWIEYDGENKTLNVTIAPLSKPRPSKPIIKNHIIDLYNVME
ESMYVGFSASTGQETSSHYLLGWSFAVNGVAPQLKISNLPNPPPKEKEPTSFPWVNIAIG
VLSASTFCLLCILFCITCYRRYYMDFEVLEDWEMDCPHRFRYKDLHLATKGFIESHLIGV
GGFGAVYKGVLPSTGAEVAVKRIVRSPFHGMREFAAEIESLGRLRHKNLVNLQGWCNKKN
DLLLVYDFIPNGSLDYVLYKPNNNNFVLNWGQRFNILKGISAGLLYLHEEWEQVVIHRDV
KTSNILIDAHLNARLGDFGLARLYNHGQLSHTTSVVGTIGYIAPELTRTGKASTSTDVYA
FGVVLLEVATGKRPLDSDQFFLVEWVIEKYHLGQILEVVDPKLDSLYDEEEIELVLKLGL
LCTQHRADYRPTMKQVTRYLNFDEPLPDIVDWGHGVSGSSRLSSGFLEVTYSMGTVETLG
YLSSISMSTKSIDAGRQQIII*

>Glyma.07G183900|Glyma.07G183900.1|35144343|Legume/protein kinase
MQLPPSKGYLHNSISTPSNINSLVMAASRYCKKSYVHASFHAITLTLLLLAIPHAASLSF
NYQQLGDTGIALNFSGKARRDNDVINLTRSEPDSYGRVTYYELLHLWDKNSEKVTDFTTH
FSFTINTPNKTHHGDGITFFLAHPDFPQSDIDGSGIGLASREQLKNLNFAKDYPFVAVEF
DTFVNDWDPKYDHVGIDVNSINTTDTTEWFTSMDERGYDADVSYDSGSNRLSVTFTGYKD
DKKIKQHLFSVVNLSDVLPEWVEIGFSSATGDFYEEHTLSSWSFNSSLGPKPQKGGSKTG
LVIGLSVGLGAGVLFVILGVTFLVRWILRNRGVEEVSLFDHTMDNDFERMSLPKKFSYEE
LARATNNFASENKIGQGGFGAVYRGFMRELNAHVAIKKVSRGSRQGVKEYASEVKIITQL
RHKNLVRLFGWCHENNDLLLVYEFMENGSLDSYLFKGKGLLTWKVRYDIARGLASALLYL
HEEWEECVLHRDIKSSNVMLDSNFNAKLGDFGLARLMDHAIGSKTTGLAGTIGYLPPEAA
TRGKASRESDVYSFGVVTLEIACGRKVIEPNLNEEQIYLVDWVWEHYGMGALLKASDASL
YGHFDEKEMERLMIVGLWCTHSDFLLRPTIRQAVQVLNFEAPLPILTSFSSMSSRTPASA
NNQHVSSNSSSSLLTESLQSSTTIDLISPAAAYLHTY*

>Glyma.07G184000|Glyma.07G184000.1|35149746|Legume/protein kinase
MHVQLSNLNFPHHLLVMLLSIFFLLIIPYASSLSFNFTSFDPNDKSIVFEGSANPVAPTI
QLTRNQMDKGMIGSIGRATYYQPMQLWDKATGNLTDFTTHFSFVIDSQNRSKYGDGIAFF
LAPAGSKIPNATKGASMGLTLDNQQLNSTDNSFVAVEFDIYQNGWDPPHEHVGIDINSMR
SASNVTWLADIKEGKLNEAWISYNSSSLNLSVVFTGFNNDTDHTIQQQHLSAIVDLRLHL
PELVSFGFSAATGNATAIHSLYSWDFTSTLAAQENITKGADPVARSPTSNIAPSQKKKNK
TGLAVGLSIGGFVCGLGLISIVLWKKWKKGTEEEEHDFEEFMGEDFGRGVETRKYSYAEL
AQAANGFKDEQKLGQGGFGGVYRGYLKDIKSHVAIKRVSEDSDQGIKEFASEIRTINRLR
HRNLVHLIGWCHERKKLLLVYEYMPNGSLDTHLFKKQSLLKWAVRYNIARGLASALLYLH
EEWEQCVVHRDIKSSNIMLDSEFNAKLGDFGLARFVDHAKGAQTTALAGTMGYMAPECAT
LGRASKESDVYSCGVVALEIACGRKPINLKAQENEINIVQWVWELFGGGRILDAADPRLE
GDFEEEQIKCLMIVGLWCAHPDHNNRASIRQAIQVLNFEAPLPNLPSSLPVPTYLDGPLH
SSIAPFSITASVEGHSQIRSSTANTNSSGFTTTSDDASPSVSLLYSR*

>Glyma.08G065500|Glyma.08G065500.1|5051641|Legume/protein kinase
MAWWRNSLQNPTLLFCYATRTIFILQIIPLANSLSFDYPNFKNGDVKWEGDASILKGAIQ
VTSNTMDQNNNYSVGRVTSYKKMLLWDMNTGKLADFTTKFSFVVFSGKSYYGDGMAFFLA
DPNLPLLKNIREGGGLGLVDGKQVLNSTQPFVAVEFDTFHNKWDPQGGTHVGLNFNSMRS
NITKQWLTDIQIWNVYNCSIEYNSSTLNLSVSFTTYNNVSKPVEEYISYKVDLRDYLPGK
VILGFSAATGKLYEVHTLRSWSFNSSLQSDENTNEIKPVAAPPTSNPDSENEHKIGLWVG
IGIGVGLVLGLLGLICALLWKRSREKKGELVFDLNMADEFPKGTGPKSFCYNELVSATNK
FAEKLGQGGFGGVYKGYLKDLKSYVAIKRISKESRQGMKEYVTEVKVISQLRHRNLVQLI
GWCHRKNDFLLIYEFMPNGSLDSHLYGVKSFLTWTVRYNIALGLASALLYLQEEWEQCVI
HRDIKSSNIMLDSCFNAKLGDFGLARLVDHEKGSQTTRIAGTRGYIAPEYFTSGKATKES
DIYSFGVVLLEIASGRKPVELEAEEGQITVVEWVWKLYGLGRFLEAADPKLCGEFDENQM

ERLVIVGLWCVHPDYSFRPSIRQVIQVLKFESALPILPEMMPVPTYLPPTIKALFSSVSS
SFWGRS*

>Glyma.08G065600|Glyma.08G065600.1|5055963|Legume/protein kinase
MQSYTSSVGRVTYPEQINLWDDSSNEPKDFTTNFSFVVSSNQSSLYGDEKWWTNVTQGEV
CNCSIVYNSRNNILKVSFTENKLGGGDATQIIQHLSYHVNITDQLSKSVTVGISAATGEY
TEEHTLFSWSFSTSTSSPTPSKGHSKKGRINTILLEGTGIGTEVWPRWFGGFHKGYFKGL
NSYAAMKRISAGSAQSLKEYAEEVTIISQLRHMNLVKLAGWCHKKNDLFLIYEYMPNGSL
DSCLFGGEKFLPWKVRYNVALGLASAWLYLQEECEKFVFHREIKSSNIMVDSNFSAKLGD
FGLARQVDHEKGSQSSVGESDTFSFGVVLLEVATGRKAIHHKDMEGGVSLVEWAWEHHGL
RNLLAAADPNLCAEFDVQQTECLLVVGLCKTRPSIRQVIKVLNFEAPFPILPPQIPVLNN
LPPTTNMLVFTASP*

>Glyma.08G065700|Glyma.08G065700.1|5063560|Legume/protein kinase
MVSAAPKAMVVPLSNLNFPYHHFLVMLLSIFFLLIIPYASALSFNFTSFDPNDKSIIYEG
SANPVAPTIQLTRNQMDKNMIGSIGRATYCQPMHLWDKATGNLTDFTIHFSFVIDSRNRS
KYGDGMAFFLAPAGLKIPNATKGGSLGLTLDNQRLNSTDNPFVAVEFDIYKNPYDPPGEH
VGIDINSLRSVANVTWLADIKEVKLNEAWISYNSSSLNLSVVLNVFNNDTDHTIQQQYLS
AKVDRRLYLPELVTFGFSAATGNATAIHSVNSWILAQLWQHKKT*

>Glyma.08G065800|Glyma.08G065800.1|5066539|Legume/protein kinase
MYSQLSNLSFSHHFLVMLLSIFFIFIIPCAFPLSFNITSFDPNGKSIIYEGSANPVTPVI
ELTGNVRDITGRATYFQPMHLWDKATGNLTDFTTHFSFVIDSRNQSAYEDGMAFFLAPAG
LKFPYVSRGGALGLTLEDQRLNSTDPFVAVEFDIYENPDDPPGEHVGIDINSLRSVANVT
WLADIKQGKLNEVWISYNSSSFNLSVVFTGFNNDTILRQHLSAITDLRLHLPEFVTVGFS
AATGIDTAIHSVNSWDFSSTLAAQENITKGADTVARYPATSNIAPSQKKKNKTGLAVGLS
IGGFVLIGGLGLISIGLWKKWKKGSVEEDLVFEEYMGEDFGRGAGPRKYSYAELTEAANG
FKDEHKLGQGGFGGVYKGYLKDIKSHVAIKRVSEGSDQGIKEFASEVNIISRLRHRNLVH
LIGWCHAGKKLLLVYEYMPNGSLDIHLFKKQSLLKWTVRYNIARGLASALLYLHEEWEQC
VVHRDIKSSNIMLDSEFNAKLGDFGLARFVDHAKSAQTTALAGTMGYMAPECATSGRASK
ESDVYSFGVVALEIACGRKPINHRAQENEINIVEWVWGLYGEGRILEAADQRLEGEFEEE
QIKCLMIVGLWCAHPDHNNRPSMRQAIQVLNFEAPLPNLPSSLPVPTYLEGPLHSFIAPF
SITSSEEGQSQITGSSSNTNSTGFTTKSDDASPSVSLLYSR*

>Glyma.08G065900|Glyma.08G065900.1|5074504|Legume/protein kinase
MVSAAPNAMYAQLSNLSFSHHFLVMLLSIFFIFIIPCAFPLSFNITSFDPNGKSIIYEGS
ANPVTPVIELTGNVRDSTGRATYFQPMHLWDKATGNLTDFTTHFSFVIDSRNRSGYGDGM
AFFLAPAGLKFPYVSRGGALGLTLENQRLNSTDPFVAVEFDIYKNFYDPPGEHVGIDINS
LRSVANVTWLADIKQGKLNEVWISYNSSSFNLSVVFTGFNNDTILRQHLSAIIDLRLHLP
EFVTVGFSAATGSSTAIHSVNSWDFSSTLAAQENITKGADTVARSPATSNIAPSQKKKNK
TGLAVGLSIGGFVLIGGLGLISICLWKKWKKGSVEEVHVFEEYMGKDFGRGGGPRKYSYA
ELTQAANGFKDEHKLGQGGFGGVYKGYLKDIKSHVAIKRVSESSDQGIKEFASEVNIISR
LRHRNLVHLIGWCHAGKKLLLVYEYMPNGSLDIHLFKKQSLLKWTVRYNIARGLASALLY
LHEEWEQCVVHRDIKSSNIMLDSEFNAKLGDFGLARFVDHAKSAQTTALAGTMGYMAPEC
ATSGRASKESDVYSFGVVALEIACGRKPINHRAQENEINIVEWVWGLYGEGRILEAADQR
LEGEFEEEQIKCLMIVGLWCAHPDHNNRPSMRQAIQVLNFEAPLPNLPSSLPVPTYLEGP
LHSFIAPFSITSSEEGQSQITGSSSNTNSTGFTTKSDDASPSVSLLYSR*

>Glyma.08G066000|Glyma.08G066000.1|5079926|Legume/protein kinase
MSLKFVTCIIFPSTPPPTQARNINYMQAIYVQFHFHRTNISSPDMAASPYSASLAFNYQQ
LGDAGNATLSISGDVYHEQEVLQLTRYETFSYGRVIYHKQLHLWDKNSGKVADFTTHFSF
TINARNNTNYADGMTFFLAHPSFPELDPRDGVGIGLLSRTQLLNPNFTKEYPFVAVEFDT
YVNPEWDPKYHHVGIQVNSFVTSVSDTTQWFTSMDQRGYDADISYDSASNRLSVSFTGYK
DNVKIKQNLSSVVNLKDKLPDWVEFGVSAATGMYYEEHTLSSWSFNSSFVFDKHKGGSKK
GLAVGMGIGGFVLIGGTGLISLGLWKKWKKVDEEENHIVEEYMGEDFERGAGPRKYSYAE
LAHAANGFKDEHKLGQGGFGGVYKGYLKDIKSHVAIKKVSEGSDQGIKEFASEVIIISRL
RHRNLVNLIGWCHERKKLLLVYEYMSNGSLDIHLFKKQSILQWAVRYNIARGLASALLYL
HEEWEQCVVHRDIKPSNIMLDSEFNAKLGDFGLARFVDHAKSAQTTALAGTMGYMAPECT
LGYRPASKESDVYSFGVVALEIACGRIPINHRAQENEISIVQWVWGLYGEGRILEAADQR
LEGKFEEEQIKCLMIVGLWCAHPDHNNRPSMRQAIQVLNFEAPLPNLPSSLPVPTYLEGP
LHSYIAPFSITASEEGQSQIISFSSNTNSIGFTTKSDDASPSVSLLYSR*

>Glyma.08G066100|Glyma.08G066100.1|5084470|Legume/protein kinase
MAASRYCKKTYVRAYFLHVTLIFLLLVIPRAAASLAFNYQQLGDTGNALKTSGDVYPDQD
VLLLTRYEPDSYGRVTYYENLHLWDKNSGKVTDFTTHFSFTINTPNKTHHGDGITFFLAH
PDFPQSGIDGSGIGLASREQLKNLNYAKDYPFVAVEFDTFVNDWDPKYDHVGIDVNSINT
TDTTEWFTSMDERGYDADISYDSASNRLSVTLTGYKDSVKIKQHLFSVVNLSDVLPEWVE
IGFSSATGFFYEEHTLSSWSFNSSLDKEQQKGGSKIGLVIGLSVGLGAGLSVLIVIWGVT
FLVRWMLKNRGLEEVSLFDHAMDNDFERMSLPKKFSYEELARATNNFARENKIGEGGFGA
VYRGLIRELNIHVAIKKVSRRSSQGVKEYASEVKIISQLRHKNLVQLLGWCHQNNDLLLV

YEFMENGSLDSYLFKGKGLLAWKVRYDIARGLASALLYLHEEWEECVLHRDIKSSNVMLD
SNFDAKLGDFGLARLMDHAIGSKTTVLAGTIGYLPPEAVTRGKASRESDVFSFGVAALEI
ACGRKAIEPNVNEEQLYLVDWVWELHGMVDLLKASDPSLYGHFDEKEMERLMIVGLWCTY
TDFHLRPTIRQVVQVLNFEAPLPTLSPQVPSFSYNSSFSSMPSRTSAFANNQCVSSTSSS
LGTGSSQSNTTCEVIISPAAAH*

>Glyma.08G066200|Glyma.08G066200.1|5090942|Legume/protein kinase
MHVQLSNLNFSYHLVVMLLSIFFLLIIPYASSLSFNFPSFDPNDNRIIYNRSANAVAPNI
QLTTNQADKGMNGSIGRATYYQPMHLWDKATGTLTDFSTNFSFVINSRGQSVYGDGIAFF
LAPAGSMVPNSTLGGTMGLTLDNQILNSTDNPFVAVEFDIFGNDWDPPGEHVGIDINSLR
SVANATWLADIKGGKVNQALISYNSTSLNLSVAFTGFKNGTALLHHLSVIVDLKLYLPEF
VTVGFSAATGNLTAIHTLNSWDFNSTSIIAPSQKKKDKKALAVGLGVGGFVLIAGLGLIS
IRLWKKTSEEEDHDFEEYIDEDFERGAGPQKYSYAELAQAANGFKDEHKLGQGGFGGVYK
GYLKDLKSHVAIKKVSEGSDQGIKEFASEVRIISRLRHRNLVNLIGWCHAGKKLLLVYEY
MSNGSLDIHLFKKQSILQWAVRYNIARGLASALLYLHEEWEQCVVHRDIKPSNIMLDSEF
NAKLGDFGLARFVDHAKSAQTTALAGTMGYMAPECTLGYRPASKESDVYSFGVVALEIAC
GRKPINHRAQENEISIVQWVWGLYGEGRILEAADQRLEGKFEEEQIKCLMIVGLWCAHPD
HSNRPSIRQAIQVLNFEAPLPNLPSSLPVPTYLEHPLHSSILPFSINASEEGQSQITGCS
SNTNSSGFTTTSDDASPSVSLM*

>Glyma.08G075200|Glyma.08G075200.1|5745983|Legume/protein kinase
MAMHAFGILLGVLYSLLSFVSSDINFVKYGFKQAGLKMDGASYVRPNGILTLINDSPKIL
GHAFYPSPLPFKSSKNKSIVATFSTTFVFSIVPKYPELGAQGFAFVLISTNKPKGCLMNQ
YLGLPNVTSSLEFSTRFLAIEFDGIQNLDLHDMNDNHVGIDISSLISNISRPVAYYLSDH
SKNISFSLKSGKPIQAWVDYNEGEMLMNVTVSPFGMPKPYFPLISFPIDLSLVLNDYMYA
GFSASNGLLVAEHNIHGWGFKIGEAGQELDKSAVPLIGSSTSTSSKVVHKKDFAVGITLT
SATLFILTVIGAFHVLRRLRNGDEILEDWELEFASHKFKYSELHSATGKFGDSNLIGYGG
FGKVYRGVIASTGLEVAVKRVAPDSRQGIREFVSEITSMAQLKHRNLVQLHGWCRKKDEL
LIVYNYVPNGSLDKLLFENEHQKKKLLTWDQRYTIITGVAQGLLYLHEECELQVVHRDVK
PSNVLIDEDLQPKLGDFGLARTYEHGINPQTTNVVGTLGYMAPELTKTGKARTSTDVYGY
GILILEVACGRKPIEPQKNPEELVLVDWVRELHHQGKISRAIDPSLDEYDKDEARLVLSL
GLFCAHPNPDYRPSMRRIVQFLLGETSLPPLPPDIHCEDPTAIRKCPNNFADDSGSSSSM
VSSSKY*

>Glyma.08G274600|Glyma.08G274600.1|36369863|Legume
MAIIKNTRAQSKTPISILMLIISFLGLVHNVKSVSFSFPSFGSYTNDITLQGEAYVNSEG
AIKLTPLSPNNVGRASYAAPLHLWDAKTGKLAGFNTTFSFVVAPSGPGLFGDGIAFFLAP
FTSNLPNNSSGGFLGLFSPNSALNVYKNQIVAVEFDSFSGNPWDPPSAHVGIDVNSIASV
TTRKWETGNSFEVAYATVNYEPIGKSLNVLVTYPGSSLNTTSLSFVIDLRTVLPEWIRVG
FSGATGQLVETHKIYSWTFASSFY*

>Glyma.08G274700|Glyma.08G274700.1|36373519|Legume
MAKTQTPFPALMICLFFVLLLNNVKSDSISFSFSNFEPGQNFDIGFLGDARPVDGAIQLT
RRDNNGPYGTPNIRQHSVGRAVYIPPVRLWDKTTGKLADFETDFSFVVDFAASQIHADGL
SFFIIPFDADPRIPKNSSGGYLGLFSPETAFNAYKNQIVAVEFDSFGNEWDPKPVPVAPH
IGIDVNSLESVETIDWPINSLPLGSVGKASISYDSNAKQLSVTVGYDSNHPPIFVGLKQI
IDLRGVLPEWVRIGFSGATGEKVETHDILSWSFTSRI*

>Glyma.08G274900|Glyma.08G274900.1|36399447|Legume
MVLSNSKPFFLLLVQFLMLHRNWDDTSFNFPNFSGPYPNTVLTFQGDARIIRGVIDPTNF
VKNAEIVPSAGRATYALPVRLWDSKSGKVASFTTTFSFKISNGPNTGDGIAFFLAPFGSN
MPRDSAGGYLGLFSRDTALRNTNKNHIVAVEFDMHQNEWDPAATPHIGIDVNSISSVATV
RWEIEELGVPTVSATVSYDSKTQIFGMALNDGTVVAYEIDLRTVLPEFVSVGFSGATGVL
IEDHEILSWTFSSSFD*

>Glyma.08G275000|Glyma.08G275000.1|36402052|Legume
MAKSKTQTPFPTLLVFLCYVLLLNNVKKSNSLSFNFSNFVSGPNFDIGFLGDARPLDGAI
QLTRRDNNGPYGTANIRQHSVGRAVYIPPVRLWDKTTGKLADFETDFSFVVDYYSAGSQI
HADGLSFFIIPFGADPRIPKNSSGGYLGLFSPETAFNAYKNQIVAVEFDSFRNEWDPEPV
PVAPHIGIDINSLESVETTDWPINSVPQGAVGKAIISYDSNAKKLYVAVGYDTQPPTIVA
LSQTIDLRVVLPEWVRIGFSGATGDMVETHDILSWSFTSHI*

>Glyma.08G275100|Glyma.08G275100.1|36421619|Legume
MAKTQNSFHILMVSLCFVLLINNVKSDSLSLRFPNFAPRQNSNIGFLGDERPLDVYIPQV
HLWDKTTGKLANLETSFSFVVDYYSAGFEIHIDGLSFFIIPFDADPSIPKNSSGGYLGLF
SPETTFNPNRGYSFGNEWDPKPVPVALHIGIDINTLESVETVGWPINYVPHGSVGQASIR
YYADVKELSVVVGYFNTQPATIVRVLQSIDLRAVLPESVRIGFSGATGDKVETHDILSWS
FNSRI*

>Glyma.08G275400|Glyma.08G275400.1|36550819|Legume/protein kinase
MHIVRYSLASPPPSLFFSFSSAMAFFTYASNSNQCPKQTLLFIFTIFTLFHTLFTTVECL
SFNFSTFQPNSNNLIDFKGDAFSSRGVLQLTKNQIDDKITFSVGRASYNQQVRLWDRRTK
KLTDFTTHFSFVMKAVDPKRFGDGLAFFIAPFDSVIPNNSAGGYLGLFSNESAFNMKKNQ
LVAVEFDSFENEWDPSSDHVGIDVNSIQSVTNVSWKSSIKNGSVANAWIWYNSTTKNLSV
FLTYADNPTFNGNSSLSYVIDLRDVLPELVRIGFSAATGSWIEVHNILSWSFSSNLDGDN
RKKVKVGLVVGLSVGLGCCLVCVVGLLWFTFWRRKNKGKEENLGVDASIDDEFERGTGPK
RFTYRELSNATNNFAEEGKLGEGGFGGVYKGLVVNSNLEVAVKRVSKGSKQGKKEYVSEV
RVISRLRHRNLVQLIGWCHEQGELLLVYEYMPNGSLDSHIFGNRVMLSWVVRHKVALGLA
SALLYLHEEWEQCVVHRDIKSSNVMLDANFNAKLGDFGLARLVDHELGSQTTVLAGTMGY
LAPECVTTGKSSKESDVYSFGVVALEITCGRKPVEVREEPSKVRLVEWVWSLYGKGKLLE
AADKKLNWEFEEQQMECLMIVGLWCCHPDHTMRPSIRQVISVLNLEAPLPSLPSKLPVPM
YYAPPMDMCKFSYTSSGVTSTTKESSPYSSMSAGSGKSLL*

>Glyma.09G110500|Glyma.09G110500.1|21870064|Legume/protein kinase
MHLPWLVKYQLASTIFIIITLTKVTCFYFNFLTFQKENESDLLLSKNSLIYLDAIQVTPD
IRGRIHDYSGRAFYNKPYKLWSKKKNQIASFNTTFVLNITPETTPGGEGLAFILTSDTTL
PQNSDGEWLGIVNATSNGTSQAGILAVEFDTRKSFTEDGPDNHVGININSINSIQQVPLI
NTGVNVSSGINVTFKIQYMNDMITVFGSMTGFEESMKTLLVSPPLNLSNYLQEVVYLGFS
ASTSNYTELNCVRSWEFSGVDIADDDNKSLLWVYITVPLVIVIIIIGGLAIFFLYWQRKR
HMEMPEDAYPRIEDQIQYSSMAPKKFKLMEITKATGGFSPQNKLGEGGFGTVYKGLLDNK
EVAVKRVSKNSRQGKQEFVAEVTTIGSLHHRNLVKLTGWCYEKRELLLVYEFMPKGSLDK
YLFGDKTFGNNTLEEGCSSTLTWETRHSVIHGVAQALDYLHNGCEKRVLHRDIKASNIML
DSDYNAKLGDFGLARTIQQRNETHHSTKEIAGTPGYMAPETFLTSRATVETDVYAFGVLV
LEVVCGRKPGSVYAQDDYKNSIVYWVWDLYGKGEVVGVVDARLKKEEIKEEEVECVVVLG
LACCHPNPHHRPSMRTVLQVLNGEAPPPEVPKERPVFMWPAMPPSFKEAEDNSLIQGTLT
PFTEITGR*

>Glyma.09G110700|Glyma.09G110700.1|21937240|Legume/protein kinase
MQLPLLVKYQLAAIIFIIISLTKVTCLSFNFSTFERKDEDHLLLNNNSKIFSSAIQVTPD
TRAQSIHNYSGRAFYNKPYKLWSQKKNQTASFNTTFVLNIDPQTTPAGGEGLAFILTSDT
NLPENSSGEWLGIVNATSNGTSQAGILAVEFDTRNSFSQDGPDNHVGININSINSIKQAP
LINTRVNLSSGEHVKIHIQYFNDTLSVFGAMDGASEESMETLLVSPPLNLSNYLQEEVYL
GFSASTSNYTQLNCVRSWEFSGVDIADDDNKSLLWVYITVPIVIVIIIIGGMVVFFLYWQ
RKRHMEMPEDAYPRIEDQIQYSSMAPKKFELRKITKATGEFSPQNKLGEGGFGTVYKGLL
DNKEVAVKRVSKNSRQGKQEFVAEVTTIGSLHHRNLVKLTGWCYEKRELLLVYEFMPKGS
LDKYLFGDKIFGNNTLEEGCSSTLTWETRHSVIHGVAQALDYLHNGCEKRVLHRDIKASN
IMLDSDYNAKLGDFGLARTIQQRNETHHSTKEIAGTPGYMAPETFLTGRATVETDVYAFG
VLVLEVVCGRRPGSVYAQDDYKNSIVYWVWDLYGKEKVVGAVDARLKKEEIKEEEVECVL
VLGLACCHPNPHHRPSMRTVLQVLNGEAPPPEVPMERPVFMWPAMPPSFKEAEDNSLIQG
TLTHFTEFTGR*

>Glyma.09G150400|Glyma.09G150400.1|37120770|Legume
MAIWKTNKSLSLPLMAFTMATMFLMLLNRVNSADSLSFSFNNFSEDQEDLILQGDATTGA
SSENDKNVLQLTKLDDSGKPEFGSVGRVLYFAPVHLWKSSQLVSTFETTFTFKISSASPD
SVPADGLAFFIASPGTTPGAGGQDLGLFPHLTSLKNSSSSHHRKVTRITGVKDLASEPLV
AVEFDTFINTDIGDPEYQHIGIDINSITSVTTTKWDWQNGKTVTAQISYNSASKRLTVVA
SYPDSTPVSLYYDIDLFTILPEWVRVGFSASTGGAAEANTLLSWSFSSSLQTNQIQKEDM
HGIVM*

>Glyma.09G201400|Glyma.09G201400.1|42571183|Legume
MAFSNSKPNLLQSLSPLIKFLIPFLLLQHSVNSQQPPSPMSAYETVGFGFGFFDKDDPNV
FLLGNASVSGGALRLTNTDQLGKPVPHSVGRALHVTPIHLWNKNNGELADFSSGFSFVVN
PKGSTLRGDGFAFFLAPANLNFPKNSSGGYLGLFNPETALDPSKNQIVAIEFDSFTNDWD
PNSPNQSPHVGIDVDSIKSVATVPWPSELEPDNAVAHASLNYNSESKSLSVFVGYPDNRN
ATVSTIVDLRNVLPEWIRVGFSASTGDLVETHDILNWSFEAAL*

>Glyma.09G201500|Glyma.09G201500.1|42573522|Legume
MANHAAQNPLSVFLTTFLLLITSAKSDLFSFNIPRFEPGALNILLDGSAKTTGGVLQLTK
KDKSGNPTQHSVGLSAFYAALHLSDAKTGRVANFATEFSFVVNTKGAPLHGDGFTFYLAS
LDFDFPDNSSGGFFGLFNKKTAFNTSLNQVVAVEFDSFANEWDPNFPQSDSPHIGIDINS
IRSVATAPWPLDIQPQGSIGKARISYQSSSKILSVSVAYPNSPVNLNATVLSYPVNLGAV
LPEWVLFGFTASTGDLVETHDILSWSFNSFL*

>Glyma.09G201600|Glyma.09G201600.1|42577045|Legume
MANHAAQNPISVFLMTFLLLITSSKSDSFSFNFPSFEPGVRNILVGDDAKTTGGVLQLTK
KDQSGNPTQHSVGLSAYFGPLHLSDRRTGRVADFATEFSFVVNTKGAPLHGDGFTFFLAS
IDYEFPDKSSGGFLGLFNKKTAFNTSLNQVVAVEFDSFANEWDPNFPESDSPHIGIDINS
IRSVATAPWPLDIQPQGSIGKAQISYQSSSKILSVSVDYPNSPVKLKPTVLSYPVNLGAV
LSEWVLIGFSGATGDLVETHDILSWSFNSFL*

>Glyma.10G013100|Glyma.10G013100.1|1176602|Legume
MATSKFHTQKPLFVVLSVVVVLLTMTKVNSTKPFLSPGTSSCRTNRTLILQGDALVTSSR
KSLGRALYSTPIHIWDSEIGSVASFAASFNFTVYASDIANLADGLAFFLAPIDTQPQTRG
GYLGLYNSTDTQQHPSNSSWGLANDQVTNVLITYDASTNLLVASLVHPSQRSSYILSDVL
DLKVALPEWVRIGFSATTGLNVASETHDVHSWSFSSNLPFGSSNTNPSDFAIFI*

>Glyma.10G108900|Glyma.10G108900.1|25055887|Legume
MATSNFSIVLSLSLALFLMLLTKANSTNTVSFTTSKFSPRQQNLILQGDAAISPSGVLRL
TKVDSYGVPTSRSLGRALYAAPIQIWDSETGKVASWATSFKFNVFSPDKTADGLAFFLAP
VGSKPQYKAGFLGLFNSDSKNMSLQTVAVEFDTYYNHGRRHIGIDVNSIKSVKTAPWGFA
NGQVAQILITYNADTSLLVASLVHPSRKTSYILSETVSLKSNLPEWVNVGFSATTGANKG
FAETHDVFSWSFASKLSDGSTSDTLDLASFLLNEAI*

>Glyma.10G113600|Glyma.10G113600.1|27715217|Legume
MATSNFSIVLSLSLAFFLVLLTKANSTNTVSFTVSKFSPRQQNLIFQGDAAISPSGVLRL
TKVDSIDVPTTGSLGRALYATPIQIWDSETGKVASWATSFKFKVFSPNKTADGLAFFLAP
VGSKPQSKGGFLGLFNSDSKNKSVQTVAVEFDTYYNAKWDPANRHIGIDVNSIKSVKTAS
WGLANGQIAQILITYDADTSLLVASLIHPSRKTSYILSETVSLKSNLPEWVNIGFSATTG
LNKGFVETHDVFSWSFASKLSDGSTSDTLDLPSFLLNEAI*

>Glyma.10G227000|Glyma.10G227000.1|45753863|Legume/protein kinase
MLCLFLLLLLLLFTFPSLVLTLPFFPSDNLTLYGDAFFTRNAITLTTQHSKCSSSSIGRA
FFIYPVRFLDPQTNSTASFSCRFSFSILSSPSCPSADGLAFLIASSTHFPTLSSGYMGLP
SSSFSSFFAVEFDTAFHPFLGDINDNHVAVDVNSLASSFASVDAASRGVDLKSGKIITAW
VEYRHAMRMVRVWIGYSSTRPPTPILATQIDLSERLEDFMHVGFTASNGEGSSVHLVHHW
QFKTFGYDDDSRSMDVVEEGDCFLCYEGDSTGKREGSSMSNKDDIERRKKIGEMALGLAG
LTAFVVSGLAAMVVVCVFLTKNKACIRKKNKEEQGQSCRFQTSKVPTRLSLSDIKSATMG
FNRDRLVGEGASAKVYKGYLPFGGDVAVKRFERDNGLDCLHNPFATEFATMVGYLRHKNL
VQLKGWCCEGNELVLVYEFLPNGSLNKVLHRNFNSSIVLSWQQRLNIVLGVASALTYLHE
ECERQIIHRDVKTCNIMLDADFTAKLGDFGLAEVYEHSSSTRDATIPAGTMGYLAPEYVY
SGVPTVKTDVYSFGVVVLEVATGRKPVEDDGTVVVDFVWGLWGKRKLIEAADPRLMGKFD
EKEMERMLLVGLLCVHPDYEKRPRVREATRILKKEAPLPLLPTSKPRVRIRPICPDDDTS
EAQSVVADWLSTDDAPYLTPRSQFY*

>Glyma.10G235600|Glyma.10G235600.1|46486132|Legume
MLFMLPNKVNSAHSVSFTFNKLGGDQKDLIFQGDATSNNNVLQLTKLDNKGNPVSGSQLT
LLQLLEFPSSLHDSTIPPHSGGRLLGLFPDSNALKNSSSSNNETAIDFKASSDKVVAVEF
DTYHNWDSWDPYYKHIGIDVNSIRSKATAQRNWQNGKIATAHISYNSASKRLTVVAFYPA
TKAVTLSHDIELNKVLPEWVRVGISASTGAHKQKNTILSWSFTSSLKNNGVQKKEDMYIA
SVA*

>Glyma.11G089100|Glyma.11G089100.1|6750992|Legume/protein kinase
MFPLQCSNHPMCAFSAVTAILLLFPAATSQAQILKKETYFFGPFNQSDFTTLTVLPSAAI
NLGALQVTPDSTGNVSLANHSGRIFFNNPFTLWDNDDNLNGKLVSFNTSFLINVFRPQNN
PPGEGITFLITASTTVPNNSHGQFLGLTNAATDGNATNKFVAVELDTVKQDFDPDDNHIG
LDINSVRSNVSVSLTPLGFEIAPNVTRFHVLWVDYDGDRKEIDVYIAEQPDKDAPIVAKP
AKPVLSSPLDLKQVVNKVSYFGFSASTGDNVELNCVLRWNITIEVFPKKNGIGKALKIGL
SVGLTMVVLIVAGVVGWVCWLKKKKRGNESQILGTLKSLPGTPREFRYQELKKATNKFDE
KHKLGQGGYGVVYRGTLPKENLEVAVKMFSRDKMKSTDDFLAELTIINRLRHKNLVRLLG
WCHRNGVLLLVYDYMPNGSLDNHIFCEEGSSTTPLSWPLRYKIITGVASALNYLHNEYDQ
KVVHRDLKASNIMLDSDFNARLGDFGLARALENDKTSYAEMEGVHGTMGYIAPECFHTGR
ATRESDVYGFGAVLLEVVCGQRPWTKNEGYECLVDWVWHLHREQRILDAVDPRLGNGCVV
EEAERVLKLGLACSHPIASERPKMQTIVQIISGSVNVPHVPPFKPAFVWPAMDLSSPASD
LTTPTTTTEYTPMSSDTHSMHVQFSDSNSLI*

>Glyma.11G213100|Glyma.11G213100.1|30598525|Legume/protein kinase
MSPLQTPFFITFFFFLCCLNASSSIFATTQFDFATLTMSTLKLLGDAHLNNNTVSLTGDP
AVPNSAAGRALYSAPVRFRQPGTPSPASFSTFFSFSVTNLNPSSVGGGLAFVISPDSSAV
GDPGGFLGLQTAAGGTFLAVEFDTLMDVEFSDVNGNHVGLDLNSVVSTQVSDLGTIGVDL
KSGDSVNAWIEYDGNAKGLRVWVSYSNLRPKDPILKVDLDVGMYVDDFMYVGFSGSTQGS
TEVHSVEWWSFNSSFDSAAAPAAATSVQKERKSSKKSTVGAVAGVVTAGAFVLALFAGAL
IWLYSNKVKYYVKKLDHSIESEIIRMPKEFSYKELKLATKGFSANRVIGHGAFGTVYKGV
LPESGDIVAVKRCNHSGQGKNEFLSELSIIGSLRHRNLVHLQGWCHEKGEILLVYDLMPN
GSLDKALYESRMALSWPHRLKILLGVSSVLAYLHHECENQVIHRDIKTSNIMLDEGFNAR
LGDFGLARQTEHDKSPDATVAAGTMGYLAPEYVLTGRATEKTDVFSYGAVVLEVASGRRP
IEKDDDAAAGNGKVGISSNLVEWVWSLHQDGKLLTAADPRLEGEFEEGEMRKVLLIGLAC
SHPDSMARPTMRCVVQMLLGEAEVPIVPRAKPSTSYSTSQLLMNLQDSDTDCKNGMITIS
TSSSENSSNGKDIV*

>Glyma.11G220900|Glyma.11G220900.1|31613010|Legume/protein kinase

MMVKTKTLSLVLHLCVMTIMVTNLAKSQQEEFFFNGFGGAASSNITLNGGAVIEHRGILR
LTNDTQRVIGHAFYPTPIQFKHRNKNATKVFSFSTAFAFAIIPQHPKLGGHGFAFTISRS
RSLEDAYPSQYLGLLNPNDVGNFSNHLFAVEFDTVQDFEFGDINGNHVGINLNNLASNKS
VEAAFFTSTNNKQKLNLKSGEVTQAWVDYDSLKNNLEVRLSTTSSKPTSPILSYKVDLSQ
IIQDSMYVGFSSSTGLLSSSHYILGWSFKINGDAKTLSLKNLPSLSASSKPQKRLIFALS
LSLIIPTVLAATALACYYFLLRKMRNSEVIEAWEMEVVGPHRFPYKELHKATKGFKDKNL
IGFGGFGRVYKGVLPKSNIEVAVKRVSNESKQGMQEFVSEISTIGRLRHRNLVQLLGWCR
KQNDLLLVYDFMRNGSLDKYLFEQPKRILSWEQRFKIIKGVASGLVYLHEEWEQTVIHRD
VKAGNVLLDNQMNGRLGDFGLAKLYEHGSNPSTTRVVGTLGYLAPELTRTGKPTTSSDVY
AFGALVLEVLCGRRPIEVKALPEELVLVEWVWERWRVGNVLAVVDPRLGGVFDEEEALLV
VKVGLSCSAEAPEERPSMRQVVRYLEREVAPPEVLVEGKKEGGGDGEFKGYAHSYSTASF
FDVESESASSLSLSGGR*

>Glyma.11G225900|Glyma.11G225900.1|32114932|Legume
MRGRMRIEEQRDRDRDKERGIVVLQSKKHSGMVAFSFFILCSLILLVQPSSSSTQQPPNL
DPDNIYLLGDAHVVSAAADNGDSHVRLTRPTPSSSGILRRREPLAFSDPTSLSTEFSFSV
SGHGHGLLLVLAAAGNVSNYVGVEFDTSKDDNAGDPNANHVSIDVGSHVSVAIANVSDLN
LVLNNGEKLQAWVDYEASSKVLEVRLSKWGEQKPSDPIVSHDIDFSKIWGKNPVIAALSS
SNGAHSVQVVSVYSWRVSLKKVSNGLHSLPADPHSNNNNNKFEDEHKKSVCPLTVLAWVI
FGTGCVALVTFVVLFMWVIFFQKGEEESLVKIPDHPSSDVRYERIDVAVDKNAHDDQS*

>Glyma.12G114100|Glyma.12G114100.1|11273065|Legume/protein kinase
MSTLLISLLLFIISNLTLLLLTSVSCTEFIYNTNFNSTNTLLHGNATIESSILTLTNRST
FSVGRAFYPFKILTKPSNSSSTPLPFSTSFIFSITPFKDLLPGHGFVFILTPSAGTTGVN
SAQHLGLFNYTNNGDPNNHVFGVEFDVFDNQEFNDINDNHVGVDINSLSSFASHDAGFWG
GGDNDEFEDLKLNDGENYQVWIEYLDSRVNVTMAPAGQKRPQRPLISEIVDLSEVLLDEM
YVGFCGATGQLVESHKILAWSFSNTNFSIGDALVTTNLPSFVHSKESILRSTGFIVGIII
GVLFVIGGAVVIFVLFLRRKRSKRKDEEQEEIEDWELEYWPHRVSYEDIYAATKGFSDQH
VIGFGGNGKVYKGLLQGVQVAVKRIPCDSEHGMREFLSEISSLGRLKHKNVVPLRGWCKK
QRSLILIYDYMDNGSLDKRIFDGDENTIFGWEKRIKVLKDVAHGILYLHEGWEVKVLHRD
IKSSNVLLDKGMNARLGDFGLARMHHHGQIAHTSQVIGTVGFMAPELIHTGRASTQTDVF
SFGVLILEVVCGRRPNEENRPLVTWLWSLKERGEECSALDERLKRRGECSIDEVKRVLHL
GLLCTHHDPHVRPSMRQVVKVLEGESLDMSLLDKINSAAGYVGSFVNRFHPTIEDIYSSN
CSFTTLTMRADE*

>Glyma.12G205100|Glyma.12G205100.1|36532632|Legume/protein kinase
MEKTISNVNLSGVLMDETYVGFTAATGRIIDSAKILAWSFSDSNFSIGDALVTENLPSFV
HHKKWFPGAQAFAVGVTSIVFVLIISCGYVAFFVLRRRKTQEEVEDWELEYWPHRIGFHE
IDAATRGFSEENVVAVGGTGKVYKGVLHGVEVAVKRIPQEREEGMREFLAEVSSLGRMKH
RNLVGLRGWCKKEKGNLILVYDFMSNGSLDKWIFECEEGMMLTWEERIQVLKNVATGILY
LHEGWEVKVLHRDIKANNVLLDKDMNARLGDFGLARMHDHQGQVVSTTRVIGTVGYIAPE
VIQRGTASTLSDVFGFGILVLEVICGRRPIEEHKPGLIEWLMSLMVQGQLHSAVDERLKA
KGGYTIEEAERLLHLGLLCSHTDPSIRPTMRQVVKILEVEIDSIESDEDNMEMSLLGKIR
SATTWSRAECALPYSGYPSFDEVKMFSFNSRTSRSGSSTFLGSESEITRENI*

>Glyma.13G238700|Glyma.13G238700.1|34901577|Legume/protein kinase
MIRHKHTHHLPSILLSILFLFNSTCAIDFVFNGFNSSEVLLFGNATVDSRILTLTHQQRF
SVGRALYNKKIPTKKPNSSRVYPFSTSFIFAMAPFEDTLPGHGLVFIFTPVTGIQGTSSA
QHLGLFNLTNNGNSSNHVFGVEFDVFQNQEFDDIDANHVGIDINSLKSYVSHDAGYWPDG
ADKSFKELTLNSGENYQVWIDYEDSWINVTMAPVGMKRPSRPLLNVSLNLSQVFEDEMFV
GFTSATGQLVESHKILGWSFSNEKFSLSDELITTGLPSFVLPKDSIFKSKGFVAGFTVGV
FFVICLLVLLALFLIQRKREKERKRMEMEDWELEYWPHRMTYEEIEAATKGFSEENVIGV
GGNGKVYKGVLRGGVEVAVKRISHENDGLREFLAEVSSLGRLKQRNLVGLRGWCKKDVGN
FLLIYDYMENGSLDKRVFDCDESKMLSYEDRIRILKDVAFAVLYLHEGWEDKVVHRDIKA
SNVLLDKDMNGRLGDFGLARMHSHGQVASTTKLVGTVGYMAPEVFKTGRASTQTDVYMFG
ILILEVLCGRRPLEEGKPPLVEWIWQLMVQGQVECALDERLRAKGEFNVQEMERVMHLGL
LCAYPEPKTRPTMRQVVNVLEGKNEVEDSEIENMDTYLLQQLKSRDILSEYSQYFSYTSH
PTFQDIRLSSSMSLTWSESVVEGR*

>Glyma.13G255500|Glyma.13G255500.1|36119175|Legume/protein kinase
MATSHVGLHATSHLFCYARGIVFFLMITFVNPLSFHYQGFEYNDARIEGDATLSHSEIQL
TATTRYQSNAYSVGRVTSFKLLQLWDMSSGKLTDFTTEFSFVIYSNETSFGDGFAFFFAD
PKLPLSNQIQQGGGLGLVDGNRLLKPTKYPFVAVEFDTHQNSWDPPGTHVGINFNSMRSN
ITVPWSIDIRQMKVYYCAIEYNASTHNLNVSFTGNQINGKPIKSYISCNVNLRDYLPERV
IFGFSAATGFMFEMNTLLSWSFRSSLPSDEKVSNQIPPMAAPPIQNPSPSPFPTANISPK
QEGNKGLLKGIEAGIGIAASFLILGLVCIFIWKRAKLKKEDSVFDLSMDDEFQKGIGPKR
FCYKELASATNNFAEAQKIGQGGFGGVYKGYLKKLNSNVAIKRISRESRQGIKEYAAEVK
IISQLRHRNLVQLIGWCHMKKDLLLIYEFMQNGSLDSHLYRGKSILTWQMRYNIAMDLAL
AVLYLHEEWEQCVLHRDIKSSNVMLDLSFNAKLGDFGLARLVDHEKGSQTTILAGTVGYI
APEYCTTGKARKESDIYSFGVVLLELASGRKPIDLNAKEGQITIFEWVWELYRLGKLLEV

VDSKLGGAFDEEQMEHLVIVGLWCANPDYTSRPSVRQVIQVLTFEAPLPVLPQKMPEPYH
HSPTMSTIFASVSSLSLATC*

>Glyma.13G296200|Glyma.13G296200.1|39540769|Legume/protein kinase
MSPRTLFFLLSTLQFLSFVSTTEFVYNRNFNSTNVKLYGNATIENSVLKLTNQTFFSIGR
AFYPHKIPMKPPNSSSSTLLPFATSFIFSVAPCENFPVAHGFAFVVTPVMSANGALSGNY
LGLFNRSTSGNSSNHVFAVEFDDFRNEEFNEENDNHVGVDLNSMISVYSEPAGFWGGREG
EELEDLKLSDGRNYQVWIEFENSVINVTMAPAGRKKPHRPLISKPMNLSWVLLDEMYVGF
SGATGRMVDNCRILAWSFSNSNFSIGDVLSTKHLPLYVHPKRLVFRSNGFIIGVTFGVFF
VGGFCALVVFFILFRNRRGEKQENFEDWELEYWPHRISYREICDATSGFSEEKVIGIGTS
GKVYKGLLKGVEVAVKSINHETRHGMREFLAEISSLGRMKHRNLVGFRGWSKRKGGKLIL
VYDYMVNESLDKRIFECEETMLLSWEERIRVLQNVADGILYLHEGWDVEVLHRDIKACNV
LLDKDMNARLGDFGLARLHHQENVADTRVIGTLGYMAPELVRIGRPSTACDVYSFGVLVL
EVVCGRRPIIADQPPLIDWLFSHMENGELSCAIDERLKGQSGYNAEEAERLLHLGLLCVS
TDPGVRPTMRQVVKTLEGIKCTECNEDCIHLALLGKINSAASWSKSSTSSANVNYPTFDE
ILQTKFYSTASLSISCPSPQLEPEFVSEGR*

>Glyma.13G296300|Glyma.13G296300.1|39544249|Legume/protein kinase
MSPLKLLIFLHTVTIFSSASTTEFVYNTNFNSTNIILYGNASVQTSILTLTNQSFFSIGR
AFYPHKIPTKLANSSTFLPFATSFIFSIVPIKNFITGHGFVFLFTPSRGVNGTTSAEYIG
LFNRSNEGNPQNHVLGVEFDPVKNEEEFNDISDNHVGIDINSLCSSTSHEAGYWGGKGDK
EFKVLDIKNGENYQVWIEFMHSQLNITMARAGQKKPRVPLISSSVNLSGVLMDEIYVGFT
AATGRIIDSAKILAWSFSNSNFSIGDALVTKNLPSFVHHKRWFSGARALAVGVTSIVCVL
IIGWGYVAFFILRRRKSQEEVEDWELEYWPHRIGFHEIDAATRRFSEENVIAVGGNGKVY
KGVLHGVEVAVKRIPQEREEGMREFLAEVSSLGRMTHRNLVGLRGWCKKERGNLILVYDF
MTNGSLDKRIFECEERLMLTWEERIQVLKNVAAGILYLHEGWEVKVLHRDIKANNVLLDK
DMNARLGDFGLARMHDHQGQVVSTTRVIGTVGYIAPEVIRSGTASTMSDVFGFGILVLEV
VCGRRPIEEHKPGLIEWLMSLMMQGQLHSAVDERLKAKGGYTIEEAERLLYLGLLCSNSD
PGIRPTMRQAVKILEVEIDSTESDEENIEMSFLGKIKSAAMWSRAECALPYRGYPSFDEV
KMFSFNSRTSGSGSSTFPGSESEITRENR*

>Glyma.14G013300|Glyma.14G013300.1|998854|Legume/protein kinase
MDFTFFFFFILTLLLWEVAVADNVSFDFPSFTLNNITLLGDSSLRNNGVVRLTNAAPTSS
TGAVVYSQPVSLFHASFSTTFSFSIHNLNPTSSGDGLAFFLSPNTTLSLSGPLGLPTATG
FVAIEFDTRLDARFDDPNENHVGFDVDSMKSLVTGDPILDGIDLKSGNTIAAWIDYNTQY
TLLNVFLSYSRSSKPLLPLLSVKFDLSHHLRDPVYVGFSASTQGSIELHHIKNWTFHSKT
ITTTLHHPHNVSVVGISRSGATKKRDKRVVGIVAGSVSFFVAFTIFLGYVFVRRWKIGGR
KEREKDKFQKSGFVAYPREFHYKELKSATREFHPSRIVGHGSFGTVYKAFFISSGTIAAV
KRSRHSHEGKTEFLAELNTIAGLRHKNLVQLQGWCVEKGELLLVYDFMPNGSLDKMLYKE
PERGKLLSWSHRQNIALGLASVLVYLHQECEQRVIHRDIKAGNILLDGNFNPRLGDFGLA
KLMDHDKSPVSTLTAGTMGYLAPEYLQYGKATDKTDVFSYGVVVLEVACGRRPIEREGSK
MLNLIDWVWGLHSEGKVIEAADKRLNGEFEEEEMRKLLILGLSCANPDSAERPSMRRVLQ
ILNNEAAPLAVPKVKPTLTFSSDLPLPLTIEDIVSEADQESMCEIKID*

>Glyma.14G100300|Glyma.14G100300.1|9818448|Legume/protein kinase
MQHVGINNNSLVSLNYSRFNIESNIGKMGHALITYNASSKLLVASWFFEGTTSGFMPKTS
VSYQIDLGEILPEWVTVGFSGATGLSNEENVIHSWEFTSTMNSTRSDVNKESYIITKYKF
QVKVVVVEVTCSILFVLVVIGVSWLIVIKKRRSGDGFDLDKASMPRRYCYNELIAATNGF
ADDRRLGEGGYGQVYKGFLTDLGRVVAVKRIFSDVEDSEEIFTNEVKIISCLIHRNLVQF
IGWCQEQGELLLVFEYMTNGSLDTHLFGSRRTLTWDVRYKIALGVARALQYLHEDAEQCV
LHRDIKSGNVLLDTAFNTKVSDFGMAKLVDPRLRTQKTRVVGTYGYLAPEYVKEGRASKE
SDMYGFGVVALEIACGRRTYKDGEYNHVPLTNWVWKQYVDGNVLNAVDEGLKRDYDVNEM
KCLLTVGLWCTLQDHKKRPKAEQVINVLKQEASLPNLFTDMRA*

>Glyma.14G100400|Glyma.14G100400.1|9839656|Legume/protein kinase
MVITVILLVLAFPSSLKTAESLNFNITNFNDPESAKNMAYQGDGKANNGSIELNIGGYLF
RIGRALYGQPLRLWDSSSGNDESATYGDGFAFYIAPRGYQIPPNGAGGTFALFNVEFDTF
NGTIDSPMQHVGIDDNSLESVASAKFDIDKNLGKKCNALITYTASNKTLFVSWSFNGTAT
PHSNSSLSRRIDLMEILPEWVDVGFSASTGKLTERNLIHSWEFSSTLNSSTASNNNSSDS
SGAKHGNRLSSVAVVVVVVCAIVLVATTVNVATWVIIMKKKRRKGDYDNDESGPTSAKFD
LDRATIPRRFDYKELVAATKGFADDTRLRRGGSGQVYKGVLSHLGRVVAVKRIFTNFESS
ERVFINEVRIISRLIHRNLVQFVGWYKVALGVALALRYLHEDAEQSVLHRDIKSANVLLD
TDFSTKLGDFGMAKLVDPRLRTQRIGVVGTYGYLAPEYINEGRASKESDIYSFGVVALEI
ASGRRTYQDGEFHVPLMNWVWQLYMEGKVLDVVDERLNKEFDVDQMTSLIIVGLWCTNPD
DKERPKAAHVIKVLQLEESLPVLPLDMHDRSPPSLITNTHARPTYYSSQSLPFTNSLGSV
GR*

>Glyma.14G100600|Glyma.14G100600.1|9884437|Legume/protein kinase
MVLTIFLLVLAIPSLKTAESLSFNITNFHDPDSAKNMAYQGDGKVNKNGSIELNIVTYIS
RVGRAFYGQPLHLWDSSSDVLTNFSTRFTFTIERATNDTIGDGFAFYLAPLGYQIPANAV

GGTLGLFNATTNTYIPHNHVVAVEFDTFNGTIDPPFQHVGIDDNSLKSVAVAEFDIYKNL
GKECNALITYTASTKTLFVSWSFNGTATPRSNSSLSYKIDLMDILPEWVVVGFSAATGQY
TERNIIHSWEFSSTLNSFTASRHGNEKHNVLLIVVVTCSTVLVVVAASFAAWVTITKRRK
GKVDNDNDELGATPVMFDLDRATIPRRIDYKELVAATKGFAADARLGRGSSGQVYKGVLS
NLGRVVAVKRIFTNSENSERVFINEVRIISRLIHRNLVQFIGWCHEQGEFLLVFEFMPNG
SLDTHLFGEKKSLAWDIRYKVALGVALALRYLHEDAEQSVLHRDIKSANVLLDTDFSTKL
GDFGMAKLVDPRLRTQRTGLVGTYGYLAPEYINHGRASKESDIYSFGVVALEIACGRRTY
QNGEFHVPLVNWVWQKYVEGNVLDVVDERLNKEYDVDEITSLIVVGLWCTNPNDRERPRA
AQVIKVLQLEASSLPVLPFDMHDGPPPPSLVTHAQSSYNSARSVPFTNSFVNVGR*

>Glyma.14G100700|Glyma.14G100700.1|9887705|Legume/protein kinase
MLATLEYFHCFKTSLLLLIFMILPIVQPLSFNITNFSDPESASLIKNEGIAKIENGTIVL
NSLINSGVGRAIYSEPLSLKNDSNGNVTDFSTRFSFTIKVLNKTNYGDGFAFYIAPLAFD
YQIPPNSSGFLLGLYGDTQNNLVAVEFDTYVNEFDPPMKHVGINNNSVASLDYKKFDIDS
NIGKMGHTLITYNASAKLLAVSWLFDGTSSGFTPNNSLSHQIDLGEILPKWVTVGFSGAT
GSSKEENVIHSWEFSPNLDLNSTNPEANNENVIIITKYKVQVKVVVVAVICSIIVVLVVV
SISWLIIKKRRTKDDFHLDKEPRRFGYNELVAATNGFADDRRLGEGGTGEVYKGFLSDLG
REVAVKRIFSDVEDSEEIFTNEVKIISRLIHRNLVQLMGWCHEQGKLLLVFEYMVNGSLD
THLFGSRRTLTWGVRYNIALGMARALRYLHEDAVQCVLHKDIKSGNVLLDTDFNIKVSDF
GMAKLVDPRLRTQKTKLEGTYGYLAPEYVKEGRVSKESDMYGFGVVVLEIACGRKTYQDG
EHNHVPLVNWVWKHYVEENILNVADKGLNMGFDVDEMTCLLTVGLWCTLQDYKKRPKAEQ
VINVLKQEVPLPKLST*

>Glyma.14G100800|Glyma.14G100800.1|9954693|Legume/protein kinase
MATSEHFHYFKTSLLLPILKFPLVQPLSFNITNFSDTESASLVEYAGVAKTENGTVVLNP
LINGEDGRVTYVQPLRLKNSSSGDVTDFSTRFSFTIDAPNKTMYADGFAFYVAPLTFAYQ
DPPNSGGLRLGLYDDNKPQNSFIAVEFDTFVNEFDPSGQHVGINNNSIASLWNKGHALIT
YNASAKLLSVSWFFEGTSSGFTPNTSLSHQIDLAETLPEWVAVGFSGSTGSYKEKNVIHS
WEFSSSLELNSTHPEDSTHREVNKESDITKCKFQVKVVVVAVTCSIIFVVLVLSVSWFII
KKRRTKDGFGNLDHMPRRFAYKELVAATNEFADDRRLGEGGYGQVYRGFLSDLGRVVAVK
RIFSDVEDSEKIFTNEVKIISRLMHRNLVQFMGWCHEQGELLLVFEYMLNGSLDTHLFGS
RRTLTWGVRYKIALGVVRALQYLHEDAVQCVLHRDIKSGNVLLDTDFNTKVSDFGMAKLV
DPRLRTQKTKLVGTYGYLAPEYVKEGRASKESDMYGFGVLALEIACGMRTYQDRENNHVP
LTNWVWKQYEVGNVLSAADKGLNDDYDVNEMTCLLTVGLWCTLHDHKKRPKAEQVINVLK
QGAPLPNLFTDMRA*

>Glyma.14G189100|Glyma.14G189100.1|45369316|Legume/protein kinase
MIGISFVLCHAAMASLSNILLMFCLFNLLAYPVSVHSAGEVPINVTKHFSFYNFSFSNNP
RLVHDVKLLGSAKFSNEKGALQIPNESEDIRHQAGRGIYSFPIRLLDPSTKTPASFQTTF
SFQMNNSTASEQAAYGGSGLTFIIVPDEFTVGRSGPWLGMLNDACENDYKAVAVEFDTRK
NPEFGDPNDNHVGINLGTIVSTKVINVSDVGLSLKDGSVYRAWITYDGPQRRMDIRLGKA
NQEDYPSKPMFSESMDLSPYLNEYMFVGFSASTGNHTQIHNLLSWNFTSTSQAFLHLPSS
ESCQGKILLENSTAATEPTNSQKSSKNEPPRSFLIFVAAVALALALFLGFYFISKHRRNA
AKLNTSVETELHMPRPPNKPRRFAFSQLSTATRSFSEIELLGSDNRGEYYRGKLSGGSQV
AVKRFSAQFLSTHGSDKKRLLKEIKGISHVRHPNLLPVRGWCQDNHEIMVAYDFVPNGSL
DKWLFGAGVLPWTRRFKVIKDVADGLSFLHTKQLAHKNLKCSSVFLDVNFRAVLGDFGFV
LMGAESKHFESQVCQGADVFEFGVLVLEVIAGRVRDEKEEGNPEERNLLGYAWNLHQIDE
KVKLVDKRMGSLINLEQAIRALEIGLLCTLNENKGRPSMEQVVDFLLNMDKPIPELPRTR
PVALFPYNSANTGLCNAYSCTF*

>Glyma.14G211200|Glyma.14G211200.1|47611571|Legume/protein kinase
MSLLCFPSILSIFCFFSFVTAATEFDFGTLTLGSLKLLGDAHLNNATVSLTRDLAVPTSS
AGRALYSRPVRFRQPGNRFPASFTTFFSFSVTNLNPSSIGGGLAFVLSPDDDTIGDAGGF
LGLSAAADGGGFIAVEFDTLMDVEFKDINGNHVGVDLNSVVSSEVGDLANVGVDLKSGDL
INAWIEFDGSSKGLSVWVSYSNLKPKDPVLTMNLDVDKYLNDFMYVGFSASTQGSTEIHR
IEWWSFGSSFAAAAAVAPPPPAVSLMNPTENSVKFAPPPSLAPSHSEEKESKSKSSCHNG
LCKQNMGAVAGVVTAGAFVLALFAGALIWFYSKKFKRVKKFDSLGSEIIRMPKQFSYKEL
NSATKCFNANRIIGHGAFGTVYKGVLPENGDIVAVKRCSHCSQGKNEFLSELSIIGSLRH
RNLVRLQGWCHEKGEILLVYDLMPNGSLDKALFEARTPLPWAHRGKILLGVASALAYLHQ
ECENQVIHRDIKTSNIMLDEGFNARLGDFGLARQTEHDKSPDATVAAGTMGYLAPEYLLT
GKATEKTDVFSYGAVVLEVASGRRPIEKDANGGGKGGISCNLVEWVWSLHREARLLMAAD
PRLEGEFDEGEMRKMLLVGLACSHPDPLTRPTMRGVVQILVGEAEVPLVPRTKPSTGFST
SHSHLLLLSLQDSVSDCDGIITISTSTSENSFNLGDIV*

>Glyma.15G059200|Glyma.15G059200.1|4568948|Legume/protein kinase
MATYQYCICKCSHLLPYLGITIAILSFLIPHAAPLDFSFQQFLNKESTLNFEGDVSYDNG
LLQLTQLKKDSVGRVTYYKPLHLWVKDSRKLTDFTSNFSFIINQPNKTHIGDGITFFLAS
PKFPLPVPPDGSGIGLVSGQQMADPNYINEHPFVAVEFDTFWNHFDPQYDHVGINIKTIK
SPFTTEWFSINDGRVHDAQISYNSSTCNLSIIFTGYEDNVTVKQHYSQVIDLREVLPDWV
EFGFSSATGLLSEIHTLCSWSFSANLDLKVHKDESKTRMVIGLSIGGGVLVVGIGLAWLL

KLKMKTRGKEDDLDLIMDSDFERGTGPKRFSYNELVRTTNNFANELKLGEGGFGGVYKGF
IRELGVEEYASEVKIISKLRHRNLVQLLGWCHKKNDLLLIYELMPNGSLDSHLFGGKSLL
TWAARYNIAGGLASALLYLHEEWEQCVLHRDLKSSNVMLDSNFNAKLGDFGLARLVDHGK
GSQTTVLAGTMGYMAPESATRGKASRESDVYSFGVVVLEIACGRKPIELRASEEQIVMVE
WVWELYGMGNLLEAADSRLCGDFDEQAMERLMIVGLWCAHPDYSARPTIREAMHVLNFEA
HLPSLPSKMPKATYIVPSITASASSNPPSSSADAFGPNKTELLSSGSYTGSPESKTSAAL
LHSIEY*

>Glyma.15G074600|Glyma.15G074600.1|5716534|Legume/protein kinase
MIRHKHTHPLLLLILLLFNSACAIDFVFNGFNSSEVLLFGNATIDSRILTLTHQQSFSVG
RALYKEKIPAKKPNSSYVYPFSISFIFAMAPFEDTLPGHGLVFIFTPITGIHGTSSAQHL
GLFNLTNNGNSSNHVFGVEFDVFQNQEFDDINANHVGIDINSLKSYVSHDAGYWPDGGDK
SFKELALNSGENYQVWIDYEDSWVNVTMAPVGMKRPSRPLFNVSLNLSQVFEDEMFVGFT
SATGQLVESHKILGWSFSNENFSLSDELITIGLPSFVLPKDSIFKSKGLVAGFTVGVFFV
VCLLVLLALFLIQRKRVKERKRLEMEDWELEYWPHRMAYEEIEAATKGFSEENVIGVGGN
GKVYKGVLRGGVEVAVKRISHENDGLREFLAEVSSLGRLKQRNLVGLRGWCKKDVGNFLL
IYDYMENRSLDKWVFDCDESKMLSYEDRIRILKDVAFAVLYLHEGWEAKVVHRDIKASNV
LLDKDMNGRLGDFGLARMHSHDQVASTTKLVGTVGYMAPEVIKTGRASTQTDVYMFGILI
LEVLCGRRPLEEGKSPLVEWIWQLMVQGQVECALDERLRAKGDFNVQEMERVMHLGLLCA
YPEPKARPTMRQVVNVLEGKNEVDDSEIENMDTYLLQQLKSRDILSEYSQYFSYTSHPTF
QDIRHSSSMSLTWSEVEGR*

>Glyma.16G119700|Glyma.16G119700.1|26751676|Legume/legume
MILPIVQPLSFNITNFSDPEIASRIQCTGIAKIENGNIVLNPLINNGVERAIYGQPLRLK
NSSNGNTPLAFDYQTPPNFSGFSLGLYGGTLDNIVAVEFDTYINEYDQPMHRVGINNNSV
ASLEYKKFRIESNIGKMGHASAFTPNDSLSHQIDLREILPKWVTVEFSGATRSSKEENVI
HSWEFSTNLDLNINDN*

>Glyma.16G119800|Glyma.16G119800.1|26753687|Legume/protein kinase
MVITIFLLVLAIPSPLIKTAESLSFNITNFHGAKSMAYEGDGKVNKNGSIELNIVTYLFR
VGRAFYKQPLHLWDSSSGVVNDFSTRFTFTIARATNDTIGDGFAFYLAPRGYRIPPNAAG
GTLGLFNATTNAYIPHNHVFAVEFDTFNSTIDPPFQHVGVDDNSLKSVAVAEFDIDKNLG
NKCNALINYTASSKILFVSWSFNNSNSTNSSLSYKIDLMDILPEWVDVGFSAATGQYTQR
NVIHSWEFSSSTASKNHNNVLLVVVVTCSTVLVVVVVVVSVAAWVMITKKRKGKVDNDNN
GERGATPVKFDLDRATLPRRFDYKELVVATKGFADDARLGRGSSGQVYKGVLSDLGRVIA
VKRIFTSFENSERVFINEVRIISRLIHRNLVQFIGWCHEQGEFLLVFEFMPNGSLDTHLF
GEKKTLAWDIRYKVALGVVLALRYLHEDAEQSVLHRDIKSANVLLDMDFSTKLGDFGMAK
LVDPRLRTQRTGVVGTYGYLAPEYINGGRASKESDIYSFGVVALEIACGRRIYQDGEFHV
PLVNWVWQLYVEGNVLGAVDERLNNEFEVDEITRLIVMGLWCTNPNDKERPKAAQVIKVL
QLEAPLPVLPLDMHNAYPPSLVTHGQPTYNSSCSVPFTNSFVSVGR*

>Glyma.17G085000|Glyma.17G085000.1|6561542|Legume/protein kinase
MSHSKTPAAGETLFSGTAFLILLHLFLFLTPALSLDFLFNSFAGVTNLTLIKDARVDASV
IRMNNDSNQYSYGRAFYPVKIPMLKTNTSNNSSSISSFSTSFVFSILPQISTSPGFGLAF
VLSNTTDPPGAIASQYFGLFTNATSPSVFPLVAVEFDTGRNPEFNDIDDNHIGIDLNNIE
SINATTAGYFNSSGAFVPVRMRTGQNIHAWIDFDGENLEFNVTVAPIGVSRPTKPTLRYQ
NPAIADYVSSNMYVGFSASKTNWIEAQRVLAWSFSDSGPARELNTTNLPVFELESSSSSL
SNGAIAGIVIGSFIFVLICASGFYLWWRMNKANEEEDEIEDWELEYWPHRFSYEELSYAT
GEFRKEMLLGSGGFGRVYKGTLPNNTEIAVKCVNHDSKQGLREFMAEISSMGRLQHKNLV
QMRGWCRKGNELLLVYDYMPNGSLNKWVFDKSDKVLGWEQRRRILVDVAEGLNYLHHGWD
QVVIHRDIKSSNILLDADMRGRLGDFGLAKLYTHGEVPNTTRVVGTLGYLAPELATVAAP
TSATDVYSFGVVLLEVACGRRPIETSVAEEEVVLIDWVRELYAKGCAREAADLRIRGEYD
EGDVEMVLKLGLACCHPDPQRRPTMKEVVALLLGEDPPEAPGKVLSDLVRGGEDSDEAAP
LQPSPPPV*

>Glyma.17G150600|Glyma.17G150600.1|12482498|Legume/protein kinase
MQYTQQPLLFIFLLILGATSFMAMDFTFFFFFFILTLLLWEVAVADNVSFDFPSFTLNNI
TLLGDSSLRNNGVVRLTNAAPTSSTGAVVYSQPVSLFHASFSTTFSFSIHNLNPTSSGDG
LAFFLSPNTTLSLSEPLGLPTATGFVAIEFDTRSDDPNENHVGFDVDSMKSLVTGDPILH
GIDLKSGNTIAALIDYNTQYTLLNVFLSYSRFSKPLLPLLSVKFDLSHHLRDPVYVGFSA
STQGSIELHHIKNWTFHAKTMTTTLHHPHNVSVVEISRSGATKKRDKRVVGIVVDSVSFF
VAFTIFLGYVFVRRWKIGGRKEREKDKFQKSGFVAYPREFHYKELKSATREFHPIRIVGH
GSFGAVYKAFFISSGTIAAVKRSRHSHEGKTEFLDELNTIAGLRHKNLVQLQGWCVEKGE
LLLVYDFMPNGSLDKMLYKEPERGKLLSWSHRQNIALGLASVLVYLHQECEQRVIHRDIK
AGNILLDGNFNPRLGDFGLAKLMDHDKGPVSTLTAGTMGYLAPEYLQYGKATDKTDVFSY
GVVVLGVACGRRPIEREGSKMLNLIDWVWRLHSEGKVIKAADKRLNGEFEEEEMRKLLIL
GLSCANPDSAERPSMRRVLQILNNEAAPLAVPKVKPTLTFSSDLPLPLTIEDIVSEADQE
SMCEIKID*

>Glyma.17G218500|Glyma.17G218500.1|36908440|Legume/protein kinase

MLGTPHTLILLLFLFLITTFSHSLVFNITNFDDPAAATAISYEGDGRTTNGSIDLNKVSY
LFRVGRAIYSKPLHLWDRSSDLAIDFVTRFTFSIEKLNLTEVAYGDGFAFYLAPLGYRIP
PNSGGGTFGLFNATTNSNLPENHVVAVEFDTFIGSTDPPTKHVGVDDNSLTSAAFGNFDI
DDNLGKKCYTLITYAASTQTLFVSWSFKAKPASTNHNDNSSSFSYQIDLKKILPEWVNIG
FSASTGLSTERNTIYSWEFSSSLNGSPADFENVKLKHQSSKLALILAVLCPLVLLFVLAS
LVAVFLIRKKRRSHDDCMLYEVGDDELGPTSVKFDLDKGTIPRRFEYKELVDATNGFSDD
RRLGQGASGQVYKGVLSYLGRVVAVKRIFADFENSERVFTNEVRIISRLIHKNLVQFIGW
CHEEGEFLLVFEYMPNGSLDSHLFGNKRVLEWHLRYKIVLGVVNALHYLHEDAEQCVLHR
DIKSANVLLDTEFNTKVGDFGMAKLVDPRLRTQRTGVVGTYGYLAPEYVNVGRASRESDI
YSFGVVSLEMASGRRTYQDGEFHVSLMNWVWQLYVEGEIMRAADEKLNNEFEVDQMRSLL
VVGLWCTNPNDKERPKAAQVIKVLNLEAPLPVLPLDMYERAPPMEIIRMPHHPSGKNHSG
MSTPITSSLVSVGR*

>Glyma.17G224200|Glyma.17G224200.1|37717367|Legume/protein kinase
MLPLKTCLLLLPIFMILLPIVQPLSFNITNFSNTESASPIEYAGVAKTENGTVVLNPLIN
GGVGRAICVQPLRLKKSSNEDVTDFSTRFSFSINAPNKTNYADGFAFYVAPLALAYQIPP
SSGGLRLGLYDDSKPQNSFVAVEFDPYVNEFDPPVQHVGINNNSIASLDYKKFDIERNIG
KMGHALITYNASAKLLSVSWFFDGTSSDANSLSHQIDLGEIIMSDWVAVGFSGSTGTTKE
ENVIHSWEFSSSLDLSSTDPEVNNENDDDNKITKYKVQVKVVVVVAVVCSIIVVIVVISV
TWLIIKKRRSGDGFGLDRAAIPRRFGYKELVAATNGFADDRRLGEGGYGQVYKGFLSDLG
RVVAVKRIFSDVEDYEEIFTNEVKIISRLMHRNLVQFMGWCHEQGEVLLVFEYMVNGSLD
THLFGSRRTLAWGVRYKVVLGVARALRYLHEDAVQCVLHRDIKSGNVLLDTDFNAKVSDF
GMAKLVDPRLRTQKTKVVGTYGYLAPEYVKEGRASKESDMYGFGVLALEIASGIRTYRDG
ENNHVPLTIWVWKHYEDGNVLNVADKGLNGDYDVNEMTCLLTVGLWCTLQEHKKRPNAEQ

VISVLKQEKPLPVLSAI*

>Glyma.17G224300|Glyma.17G224300.1|37732104|Reverse transcriptase/Legume/protein kinase
MRLEENKVDQCIYLKVSGSKFIFLLLYVDDILLAFNNLGMLHETKDMATRSFDMKDLGKP
LESLTFFILPSKFREFHQEVTTIVVYEDVGDFSDNEDLMPSEITFYRDVCGEEIFEGKMV
SSDECFSVQIHIPEDYYIMAVSPQAPQNCYSSHFLRLRNLPKPKEKRVFKVQRLAIDGSS
NSIFDEWIEHYGSVLNLGYACRSNEVCAFSVIEVRYVKGEWLNNWKDNKKEAEKKRSFEV
VLEMFRALSNKGRKKQPQLRLLEMVITVFLLVLAIPSPLKTAESLNFNITNFANSESAKN
MLYVGDGAVNKNGSIELNIVDYDFRVGRALYGQPLRLWDSSSGVVTDFSTRFTFTIDRGN
NKSASYADGFAFYIAPHGYQIPPNAAGGTFALFNVTSNPFIPRNHVLAVEFDTFNGTIDP
PFQHVGIDDNSLKSVATAKFDIDKNLGKKCNALVNYNASNRTLFVSWSFNGAATPNSKNS
SVSYQIDDLMDILPEWVDVGFSASTGDLTERNIIHSWEFSSTLNSSTVSNNNSSDNNGAK
DRNGLSSVAVVAVAACAIVLVAAAANFAAWVIIMKKRRGKGDYYDNDESGHTSAKFDLDR
ETIPRRFDYKELVVATNGFADDTRLGRGGSGQVYKGVLSHLGRVVAVKRIFTNSENSERV
FINEVRIISRLIHRNLVQFVGWCHEQGEFLLVFEFMPNGSLDSHLFGDKKTLPWDVRYKV
ALGVALAIRYLHEDAEQSVLHRDIKSANVLLDTDFSTKLGDFGMAKLLDPRLRTQRTGVV
GTYGYLAPEYINGGRASKESDIYSFGVVALEIACGRRTYKDGEFLVPLVNWMWKLYVEGK
VLDAVDERLNKEFDVDEMTSLIVVGLWCTNPNNKERPTATQVIKVLQLEAPLPTLPLDMH
DGPPLSLNTYTHAQPPYDSLQSVPFTNSLQSVGR*

>Glyma.17G224500|Glyma.17G224500.1|37772129|Legume/protein kinase
MLLLATTSCIPCLKTFLLLHILILPIIVQPLSFNITNFNDTESTNLIFGGESRIIIQNGT
IVLNSDIGNGVGRATYGQPLCFKNSSNGHVTDFSTRFSFTIDVSNKTFFGDGFAFYVAPH
PYQIPLNSGGGRLGLYDDNAPAPHSNIVAVEFDTYVNRYVDPNMRHVGINNNSAMSLAYD
RFDIESNIGKMGHALITYNASAKLLSVSWFFEGTSSGFTPNANSLSYRIDLGEALPEWVT
IGFSGATGSLKEENVIHSWEFSSTMNSMHSEVSNENDDRIMVKYKFQVKAVVVAVMTTCV
FFVLVIIGVYWLIIKKRRSEDGYDLDRETIPRRFYYKELVAATNGFADDRKLGRGGSGQV
YKGILSYLGRDVAVKRIFTNLENSERVFINEVRIISRLIHRNLVQFIGWCHEEGEFVLVF
EYMPNGSLDTISLGRKKLWRGMYKVALSVTLALRYLHEDAEQCVLHRDIKSANVLLDTNF
NTKLGDFGMAKLVDPRLRTQRTRVVGTYGYLAPEYINGGRASKESDIYSFGVLALEIACG
RRTYKDGEFHVPLVKWVWQQYVGGNVLNVVDERLNKEFNVNEMTSLIIVGLWCTNPNDKE
RPKAAQVIKVLQLEAPLPVLPLDMHDRPPPSLAMHAQQPTNYSSQSLPFTNSIISVGR*

>Glyma.17G224600|Glyma.17G224600.1|37777526|Legume/protein kinase
MLATSACFHYLETFLLLCTLMILPIVKPQPPLSFNITNFNDSTGIASLIGCVGVARIGNG
TIVLTNSLNNNNDNNSSFDFGRAIYGQPMRLKNTSNGHVVVTDFSTRFSFSSFSIDGSTE
SDFGEGFAFYMAPIAYHIPLGSGGSRLGIYGDKVHDPTNIVAVEFDTFQNVGFDPPLNQH
VGINNNSVVSLAYARLDIEGNIGNMGHVLITYNASAKLLAVSWFFEGRNSSSSAPEASLS
HQIDLGEILPEWVTVGFSGGNGNSKGKNVIHSWEFSSNMDLKSTRNPEVINKGSDDITKC
KFQVKVVVVAVTCSIIIFVVMVISASWFIINKRRTGDGFGLDHRAAIPRRFSYNELVAAT
NGFADDGRLGEGGTGQVYKGILGDLGRVVAVKRIFSDVEDSERMFTNEVNIISRLIHRNL
VQFLGWCHEQGELLLVFEYLTNGSLDTHIFGNRRTLTWDVRYKIALGVARALRYLHEDAE
QCVLHRDIKSANILLDTDFNTKVSDFGIAKLVDPRLRTQKTKVVGTYGYLAPEYLNEGRA
SKESDMYGFGVVVLEIACGRKTYQDAEHNHVPLVNWVWKHYVEGNILNVADKGLNMDFDV

DEMTCLLTVGLWCTLHNHKKRPHAEQVISVLKQEATLPKLST*

>Glyma.18G031400|Glyma.18G031400.1|2408367|Legume
MVAFSLFILCSLILLVQPSSSSTQQPPNLDPDSIYLLGDAHVVTAADADSHVRLTRPAPS
SSGILRRREPLAFADPTSLSTEFSFSVTGHGHGLLLVLAAGGNLSNYVGVEFDTSKDDNV
GDPNANHVGIDVGSHVSVAVANVSDVHLVLNNGEKLNAWVDYEASSKVLEVRLSKWGAQK
PSDPIVSHDIDFSKIWGANPVIAGISSSNGAHSVQVVSVYSWKLSLKKVSVSNGLHSLPA
DPHGNNNNKFEDEHKKLCPLTVLAGVIFGTGCVAMVTFVVLFMWVIFFQKGEEESLVKIP
NHPSSDVRYERIDVAVDKNAHGDES*

>Glyma.18G043500|Glyma.18G043500.1|3711823|Legume/protein kinase
MSPLQTSFFVFFLCFLNASSSIFATTQFDFGTLTMSTLKLLGDAHLNNNTVSLTGDPAVP
NSAAGRALYSAPVRFRQPGTPSPASFSTFFSFSVTNLNPSSVGGGLAFVISPDSSAVGDP
GGFLGLQTAGGGNFLAVEFDTLMDVEFSDINGNHVGLDLNSVVSTQVSDLGGIGVDLKSG
DSVNAWIEYDGNAKGLRVWVSYSNVRPKDPILKVDLDVGMYVNDFMYVGFSGSTQGSTEV
HSVEWWSFNSSFDSAAAPAASSAPSATSEQKESRSSRKSTVGAVAGVVTAGAFVLALFAG
ALIWVYSKKVKYVKKLDHSIESEIIRMPKEFSYKELKLATKGFSANRVIGHGAFGTVYKG
VLPESGDIVAVKRCNHSGQGKNEFLSELSIIGSLRHRNLVHLQGWCHEKGEILLVYDLMP
NGSLDKALHESRMPLSWPHRLKILLGVSSVLAYLHHECENQVIHRDIKTSNIMLDEGFIA
RLGDFGLARQTEHDKSPDATVAAGTMGYLAPEYVLTGRATEKTDVFSYGAVVLEVASGRR
PIEKDAPAAGNGKVGISSNLVEWVWSLHQEGKLLTAADPRLEGEFEEGEMRKVLLVGLAC
SHPDSMARPTMRGVVQMLLGEAEVPIVPRAKPSTSYSTSQLLMNLQDSESECNNGMITIS
TSSSESSFNGRDSLMEV*

>Glyma.18G076200|Glyma.18G076200.1|7212910|Legume/protein kinase
MKITRSTYDLIIPNSRRNILNVFLDIVLGALRAKPKNRTGKGMLIVPIRAGQTGRGDSDL
TTPNSTLTNIPTTFTDLNTQNNNIPITSWLDYHAHTKKLNVFLNYSSVPSSKPQNPILSV
DLDLSHYFKDNLYVGFSGSTLGSTELVQVMSWSFEFESFQKPGSNLHPDNGSRTPASVAV
SDIPSPSNSTEGNSYNRGKRFFFGVAVAIAGPAFFCVVLVVLGYVSFLKWRGVRKLQKSF
GTVGCCPKEFGYKEVKLATKGFHPSRVIGKGSFGTVYKALFESSGTIAAVKRSRQYSHEG
RTEFLAELSVIAGLRHKNLVQLLGWCVEKGELLLVYEFMPNGSLDKVLYQECESGNNSNN
VLSWNHRVNIAVGLASVLSYLHQECEQRVIHRDIKTGNILLDGSMNPRLGDFGLAKLMDH
DKSPVSTLTAGTMGYLAPEYLQCGMANEKTDVFSYGVVVLEVACGRRPIEREGQKMVNLV
DWVWGLHSQGTIIEAADKRLNGDFREGEMKRLLLLGLSCANPDSAQRPSMRRVLQILNNN
QGVALVVPKEKPTLTFSSGLPLSLDEIVSDAEEELDSGQVVCEIKID*

>Glyma.18G150100|Glyma.18G150100.1|27332702|Legume
MAIKNSRAQTQTLFSILVLIISFLALVHNVKSVSFSFPSFGSYTNDITLQGDAYVNSEGA
IKLTPVAPNSVGRASYAAPVHLWDAKTGKLAGFNTTFSFVVMPNVPGLFGDGIAFFLAPF
NSNIPNNSSGGFLGLFSPNYALNVYKNQIVAVELDSFSGNPWDPPSAHVGIDVNSIASVA
TRKWETGNAVNGFVAYANLNYEPVGKSLNVLVTYPGSKVNATSLSFVIDLRTVLPEWVTV
GFSGATGQLVEIHKIFSWTFTSSFY*

>Glyma.18G150200|Glyma.18G150200.1|27449066|Legume
MAFFTSASYSNQTQKQTSLLIFIIIFTLFHTLFYTAVSVSFNFSTFQPNSNNLIDFDGDAF
SSNGVLLLTKNQLDGSITFSVGRASYDQPVRLWDRRTNKLTDFTTHFSFVMKAVDPSRFG
DGLAFFIAPFDSSIPNNSAGGYLGLFSNESAFNTKKNQLVAVEFDSFQNTWDPSSDHVGI
NVNSIQSVATVAWKSSIKNGSVADAWIWYNSTTKSLSVFLTYAHNQTFSGNSSLSYAIDL
RDVLPEFVRIGFSAATGSWIEIHNILSWSFNSNLLS*

>Glyma.18G150300|Glyma.18G150300.1|27618064|Legume/protein kinase
MVFFASVSNSNQCRKQTLLFIFTIFTLFHTLFTTVVSLSFNSSTFQLNSNNLIDFKGDAF
SSNGVLQLTKNQIDDKITFSVGRASYNQPVRLWDGRTKKLTDFTTHFSFVMKAIDPSRFG
DGLAFFLAPFDSVLPNNSAGGYLGLFSNESAFNTKKNQLVAVEFDSFKNEWDPSSDHVGI
NVNSIQSVTNVTWKSSIKNGSVANAWIWYNSTTKNLSVFLTYANNPTFNGNSSLWYVIDL
RDVLPEFVRIGFSAATGSWIEIHNILSWSFSSSLDEGSRKKVKVGLVVGLSVGLGCLVCV
VGLLWFTFWRRKNKGKEDNLGVDASIDDEFERGTGPKRFTYPELSNATNNFAEEGKLGEG
GFGGVYKGIVVHSNLEVAVKRVSKGSKQGKKEYVSEVRVISRLRHRNLVQLIGWCHEQGE
LLLVYEYMPNGSLDSHLFGNRVMLSWVVRHKVALGLASALLYLHEEWEQCVVHRDIKSSN
VMLDANFNAKLGDFGLARLVDHELGSQTTVLAGTMGYLAPECVTTGKSSKESDVYSFGVV
ALEITCGRKPVEVREEPSKVRLVEWVWSLYGKGKLLEAADQKLNWEFEEQQMECLMIVGL
WCCHPDHTMRPSIRQVISVLNFEAPLPSLPSKLPVPMYYAPPMDMCKFSYTSSGVTSTTK
ASSPYSSISEGSGKSLLKH*

>Glyma.18G185400|Glyma.18G185400.1|44566780|Legume/protein kinase
MSLKVVTVVFLLATIVVASDDTSFTYNGFQSSYLYLDGSAEFTTNGMLKLTNHTKQQKGH
AFFPSPIVFKNTTSGSVFSFSTTFVFAIRSEFPNLSGHGIVFVVSPTKGVPHSLPSQYLG
LFDDTNNGNNSNHIFGVELDTILNTEFGDINDNHVGVDVNELKSVKSAAAGYYSDEGFKN
LSLISGYPMQVWVEYDGLKKQIDVTLAPINVGKPEGPLLSLSKDLSPILNSSMYVGFSSS
TGSILSSHYVLGWSFKVNGKAQQLAISELPMLPRLGGKEESKVLIVGLPLILLSLILMVA

LAVVHVIKRKKFTELLEDWEQDYGPHRFKYKDLSLATKGFREKELLGSGGFGRVYKGVMP
ISKIEVAVKKVSRESRQGMREFVAEIVSIGCLRHRNLVPLLGYCRRKGELLLVYDYMPNG
SLDKYLYNKPRVTLNWSQRFKITKGVASGLFYLHEEWEQVVVHRDIKASNVLLDAELNGR
LGDFGLSRLYEHGTDPHTTHVVGTLGYLAPEHTRTGKATTSSDVFAFGAFMLEVVCGRRP
IEKGGESGSEILVDWVYNCWKKGEILESMDPNLGANYRPDEVELVLKLALLCSHSEPLAR
PSMRQVVQYLEKDVPLPDLCMLSLSSNGLTFGLHEDFQDCPMSYPSSMNRPISHTSSIVE
SLLSGGR*

>Glyma.18G185500|Glyma.18G185500.1|44588776|Legume/protein kinase
MATLLKILSLTVLLLIPVSCQVDQLLYAGFKDVGASNLTMNGVAKIEHNGILKLTNDSSR
LMGHAFYPSPFQLKNSTSGKVLSFSSSFALAIVPEYPKLGGHGLAFTIATSKDLKALPSQ
YLGLLNSSDNGNISNHIFAVEFDTVQDFEFGDINDNHVGIDINSMQSNASANVSLVGLTL
KSGKPILAWVDYDSQLNLISVALSPNSSKPKTPLLTFNVDLSPVFHDIMYVGFSASTGLL
ASSHYILGWSFKINGPAPPLDLSSLPQLPQPKKKQTSLIIGVSVSVFVIVLLAISIGIYF
YRKIKNADVIEAWELEIGPHRYSYQELKKATRGFKDKELLGQGGFGRVYKGTLPNSKIQV
AVKRVSHESKQGLREFVSEIASIGRLRHRNLVQLLGWCRRRGDLLLVYDFMANGSLDKYL
FDEPKIILNWEHRFKIIKGVASALLYLHEGYEQVVIHRDVKASNVLLDFELNGRLGDFGL
ARLYEHGANPSTTRVVGTLGYLAPELPRTGKATTSSDVFAFGALLLEVACGRRPIEPKAL
PEELVLVDWVWEKYKQGRILDLVDPKLNVYFDEKEVIVVLKLGLMCSNDVPVTRPSMRQV
VRYLDGEVEVPEDLKKPGDISHHEGFDEFLHSLASSSFDKMSSGSNFGNRDMESSFLSFA
NSPHSLLHGRGETR*

>Glyma.18G193900|Glyma.18G193900.1|46708118|Legume
MPTSIMAPFSTSPHFTAFTFLILFLKTQAFDPLSFFSFTDFEKDPNFKSSVGLYGNAKVV
NNGSEVLLSGNGGRVIYKKPVKLVHGGARELVSFSTYFGFSMSLDSEKSGLAFVMVPSGV
EGEVFGNSSYGLSFGLKEREFKVVGVQFSAYGRNGGSGSCIVSINVGSSVPVKTINASSV
IMGLKSEGKLHAWIDYEASSKRLEVRLNQYGQSRPVDPLLWHSMDLSNVWGTEEMFAGFS
TVKENNTSQTCFLYSWSFIVRHFPHWMHSEPLNPKVLAKKTETPAVKSRSDCLLRVLAAM
IFGAGCGALTAFIVLYLWTIFGNKRPVVPEEYAMQPVDFEYKKVSIVVDKTTIIDAKE*

>Glyma.18G205000|Glyma.18G205000.1|48865940|Legume/protein kinase
MPIPLMASPTLLVFLLFLSLLHQVASKAVIFEGFDENSELNLQGSSIIKTSRLLKLTNRS
TNIVGHAFYATPFQMLNKNNTNPPLQPYAYSFSTNFVFSIVSPISGSGGFGLAFTIAPST
QFPGAEAGHYLGLVNSANDGNDSNHIFAVEFDTVNGYKDDSDTEGNHVGVNINGMDSIIT
EPAAYIEEGTDNVKEDFRMAKVDAVQVWIEYDGEKKTLNVTIAPLPLPRPSKPIIMNHNI
DLYNVMEESMYVGFSASTGQETSSHYLLGWSFVVNGVAPLLNISNLPKPPPKEKEPTSFP
WVNVAIGILSGLTFCLLCILFCLTCYRRYMDFEVLEDWEMDCPHRFRYKDLHIATKGFIE
SQLIGVGGFGAVYKGVLPSTGAEVAVKRIVRSPFHGMREFAAEIESLGKLRHKNLVNLQG
WCKKKNDLLLVYDFIPNGSLDYVLYKPNNNNNFVLNWGQRFNILKDISAGLLYLHEEWEQ
VVIHRDVKTSNILIDAHLNARLGDFGLARLYNHGQVSHTTSVVGTIGYIAPELTRTGKAC
ANTDVYSFGVVLLEVATGKRPLDSDQFFLVEWVIENYHLGQILEVVDPKLDSLYDEEEVE
LVLKLGLLCTQHRADYRPSMKQVTRYLNFDDPLPDIADWGHDVSGSSRLSEGFLEVTSSM
GTVEALGYLSSISMSTKSIDAGR*

>Glyma.20G072500|Glyma.20G072500.1|25665985|Legume/protein kinase
MFVIILLLLSNLLIQTQSGASTVVIDPNTFSFVTFTPESCTNGELLCMGSATAGNGYLSL
TPEPQQSGNSSSSLSNTTNNVGRVLYPLPVNVWPAIISTTFTVRITPFSENSTGSGDGMA
LVFAQDNRPSPNASYGSYLGMFDQSTQGGGFQQIAVEMDTFMNEFDLDGNHIGIVTTSIT
NPLASESLNSSGIDLKSGRDIEVKIDYDGWSKMIFVSVGYSESQLKSVLNHSINLPDIVP
SSIYVGFTASTGNTFPESHQVLNWVFTSVPLPILSAEHTKVGTIKTILVVVAVCLFPFIW
IAASLRRRYMRAKKKGDIESLSKKAADIAEVFTYKQLSRATCNFSQENLLGRGAFGSVYK
GIILDSGKTVAVKKISATSKQGEREFLAEICTIGRLRHKNLVKLQGGASEGIIFSWQGQF
ELANQATRILQGLASALLYLHEECGNPFVHRDVKPNNVMLDSNHDAHLGDFGLARLLKNE
GSVTTNLNGTLGYLAPELSFTGRATPESDVYSFGMVVLEVTCGKRLNWLKQGNSFVDSVW
NLHSQNALLECVDQRLENKFDEEEAKRALMVGLACMHPDSLFRPRMRKVVNIFQNPNEPL
MELPGVRPTGVYVSVSSSTSASNSSSRGELQIQSSTKSSDGKFNYV*

>Glyma.20G158700|Glyma.20G158700.1|39726429|Legume
MGLWNTTNAVSCRFHKFGDDQKNLVFQGDATSSSRGIELTKLDGGGKPVGGSVGRVLYSS
PVHLWESSTVVASFETDFTFSISSDSTTPGDGLAFFIAPFDTKIPPNSGGSNLGLFPSDN
VVAVEFDTYPNRDKGDPDYRHIGIDVNSIVSKATARWEWQNGKIATVHISYNSASKRLTV
AAFYPGTQTVTLSHDIELNKVLPEWVRVGLSASTGQQKQTNTIHSWSLAFNNSIG*

>Glyma.20G165300|Glyma.20G165300.1|40273684|Legume/protein kinase
MLCLFFFLFFTHNAITLTTQHSTCSSSSIGRAFFIYPVRFLDPQTNSTVSFSCHFSFSIL
SSPSCPSRDGLSFLIASSTPFSTLSSGYMGLPSPQRSSLSFFAVEFDTAFYPSLGDINDN
HVAVDVNSLASFASTNAAMVRVWAGYSSTRPPTPILAAQIDLSERLEDFMHVGFTASNGE
GSSVHLVHHWQFKTFGYDSPSMYVVEEGDCFLCYEGDSTEEEKDWGNGSWVGRVDSVCGV
WFGGNDCGTSKMPTRLSLSDIKSATMGFNRDRLVGEGASAKVYKGFLPFRGDVAVKRFER
ENDLDCLHNPFATEFATIVSYLRNKNLVQLKGWCCEGNELVLVYEFLPNGSLNKVLHRNF

NSSIVLSWQQRVNIVLGVASALTYLHEECERQIIHRDGQWGILPLNMFILVLEVATGRKP
VEDGGTVVADFVWGLWEKRKLIEAADPRLMGKFDELEMESSPLPLLPTSKPRVRSRPICP
DDDTSDAQSVVADLPSTDEAPYLTPRNQFY*

>Glyma.U032400|Glyma.U032400.1|226169|Legume/protein kinase
MIQPQSGASTVVDPNTFSFVTFTPESCTNGELLCMGSATAGNGYLSLTPEPQQSGNLSLS
LSNTTNSVGRVLYPHPVKVWPAIISTTFTVRITPFSENSTNAGDGMALVFAQDNRPSPNG
SYLGMFDQSTQGGGFQKIALEMDTFMNEFDLDGNHIGIVTTSITNPLASESLNSSGIDLK
SGRDIEVRVDYDGWSKMIFVSVGYTESQLKSVLNHSINLPDIVPSSIYVGFTASTGNNTF
PESHQVLNWVFTSVPLPILSVELTKVGTIKTILVVVMVCLFPCIWIAASLRRTYVRAKKK
GDIESLTKKAADIPKVFTYKQLSRATCKFSQENLLGKGAFGSVYRGIILDSGKTVAVKKI
SATSKQGEREFLAEICTIGRLRHKNLVKLQGWCSEGENLLLVYDYMQNGSLDHFIGKGSL
NWQTRHKILTGLASALLYLHEECGNPFVHRDVKPNNVMLDSNHNAHLGDFGLARLLKNEG
SVTTNLNGTLGYLAPELSFTGRATPESDVYSFGMVVLEVICGKRLNWLKQGNSFVDSVWN
LHAQNALLECVDQRLENKFDEEEAKRALMVGLACLHPDSMFRPRMRKAVNIFQSPNEPLM
ELPGVRPTGVYVSVSSSSSLASNSSSRGELQIQLSRKSSDGKFNYV*

LysM

>Glyma.01G027100|Glyma.01G027100.1|2841569|LysM/protein kinase
MASSLVALLSLLLTLLATSCLSATVFSLQVSIKTTYLEPFKCSPKITTCNASLYHISYGN
NIDDIATFYSVSTSQIKPIMRTTEQDYLITVPCSCNDTNGLGGYFYDTTYKVKSNDTFVN
INNFVYSGQAWPINGELDQNEELTIHLPCGCSEKSDSQIVVTYTVQRNDTPVSIAALLNA
TLDDMVSMNEVLAQNPSFIDVTWVLYVPRELNGLPLSKGKDKKQKLEIIIGILAGVTLLS
IITLIILSVVLRRSRANKTAKNDPSVVSKRSITNRTISIKNRDFHTEYIEDATTFESERP
VIYALEEIEDATNNFDETRRIGVGGYGTVYFGMLEEKEVAVKKMRSNKSKEFYAELKALC
RIHHINIVELLGYASGDDHLYLVYEFVPNGSLCEHLHDPLLKGHQPLSWCARIQIALDAA
KGLEYIHDYTKARYVHRDIKTSNILLDEKLRAKVADFGLAKLVERTNDEELIATRLVGTP
GYLPPESVKELQVTIKTDVFAFGVVLAELITGKRALFRDNQEASNMKSLTSVVGQIFKDD
DPETVLADAIDGNLQRSYPMEDVYKMAELAHWCLCEDPNVRPEMREIVVALSQIVMSSTE
WEASLGGDREVFSGVLDGR*

>Glyma.01G179000|Glyma.01G179000.1|51506809|LysM/protein kinase
MYKKWCIIAFISLQTTMDLFPFIPIIIFTLLIHNFSLILGQQPYIGLGTVACPRRGNKNS
IRGYTCNGANHSCQSYLTFRSQPIYNSVKTISTLLGSDPSQLAKINSVSMNDTFETNKLV
IVPVNCSCAGEYYQTNTSYEFHNSETYFLIANNTFEGLTTCQALENQNHNPANIYPGRRL
LVPLRCACPTKNQTEKGIRYLLSYLVNWGDSVSFISEKFGVNFMTTLEANTLTLTQATIY
PFTTILVPLHDKPSSSQTVSPTRRTPPPSPPSSDHSSNKTWVYVVVGVVVGAIALISVLC
AVIFFTRYRKNRKKDDSVVVGSKSFEAIEEKPEVKVNEKLSEIISGIAQSFKVYNFEELQ
RATDNFSPSSWIKGSVYRGVINGDLAAIKRIEGDVSKEIEILNKINHSNVIRLSGVSFHE
GGWYLVYEYAANGDLSEWIYFHNVNGKFLSWTQRMQIALDVATGLDYLHSFTSPPHIHKD
INSSNILLDGDFRGKVTNLSLARCLEGGDDQLPATRHIVGTRGYMAPEYLENGLVSTKLD
VYAFGVLMLEMVTGKEVAAILTEDETKLSHVLSGILGEESGKEMLKEFVDPSLGENCPLE
LAMFVIEMIDNCIKTDPASRPSVHEIVQSMSRTLKSSLSWERSMNVPRN*

>Glyma.02G000400|Glyma.02G000400.1|40099|LysM/protein kinase
MTTSSLKTMHQFFSAVFLFLLLPLCSNAQTARQANNTGFTCNFTRTCTSYAFYRATAPNF
TDLASIGDLFSVSRLMISTPSNISSSSLNTPLLPNTPLFVPLTCSCNPVNASFGSLSYAN
ISYTINPGDTFFLVSTIKFQNLTTFPSVEVVNPTLLATNLSIGQDTIFPIFCKCPPNSQG
TNYMISYVVQPEDNMSSIASTFGAEEQSIIDANGGETTLHDYDTIFVPVARLPALSQPAV
VPHAPPPVIGSNNDDRTGTVRGLGVGLGIVGLLLILVSGVWVYREVVVMKGVVRDDEEKN
VYLGGKAEGKNLDVKLMANVSDCLDKYRVFGIDELVEATDGFDQSCLIQGSVYKGEIDGH
VFAIKKMKWNAYEELKILQKVNHGNLVKLEGFCIDPEEANCYLVYEYVENGSLYSWLHEG
KKEKLSWKIRLRIAIDIANGLQYIHEHTRPRVVHKDIKSSNILLDSNMRAKIANFGLAKS
GMNAITMHIVGTQGYIAPEYLADGVVSTKMDVFAFGVVLLELISGKEVINEEGNLLWASA
IKTFEVDNEQEKTRRLKEWLDKDILRETFSMESLMGALTVAIACLHRDPSKRPSIMDIVY
ALSKSEDMGFDISDDGIGSPRVIAR*

>Glyma.02G059700|Glyma.02G059700.1|5377614|LysM/LysM/protein kinase
MNLFPMITTTIFTLIMLCSMSVVVHVQGKQPYVGLATTACGQTGNSNSMRGYTCNGVNPS
CQAYLTFRAQPLYNTVPSISALLGSDSSQLSVANSVSEDGTFETNKLVIVPINCSCSGNN
NNQYYQFNTSYEVERGDSYFVIANNTFEGLSTCQALQDQNNIPEGDLMPGNELIVPLRCA
CPSKNQTEQGVKYLLSYLVASNHIVWLIGERFGVSSETIVEANTLSSQQPIIHPFTTLLV
PLQDEPSSNQTSEPSPPPSTPPPPPLSSSSGRSSKTWVYAVVGVVGAIALISSVLCAIVF
RTRYLKGGNKRKDDSLIVSDSFVAVAIEKPQEKKLEEEESENLAEIISGISESFKVYRYE
ELQSATNGFSPSCCIKGSVYRGFINGDLAAIKKIDGDVSKEIELLSKVNHSNVIRLSGVC
FNGGYWYLVYEYAANGYLSDWINIKGKFLSWTQRIQIALDVATGLDYLHSFTSPPHVHKD
LKSGNILLDSDFRAKISNFRLARSVEREGSEGDQYVMTRHIVGTRGYMAPEYLENGLVST

KLDVYAFGVLMLEMLTGKDVADVYAEGNIANLFDVLSAVLDEEGEHLRLSEFMDPSLKGN
YPMELAVFVARMIETCIKKDPASRPDMHEIVSSLSKALDSSLRWETSMERKFRFYKGFLN
*

>Glyma.02G269600|Glyma.02G269600.1|45381938|LysM/LysM/protein kinase
MATIQCATTTLILLLLLLLIIPRSNSQQEYVNNKQLDCNNEYNSTKGNLCNSLPSCTSYL
TFKSSPPEYTTPAAISFLLNSTPALIAAANNITDVQTLPADTLVTVPVNCSCSGPYYQHN
ASYTIKVQGETYFSIANNTYQALTTCQALELQNTVGMRDLLKGQNLHVPLRCACPTQKQR
EAGFKYLLTYLVSQGESVSAIGDIFGVDEQSILDANELSTSSVIFYFTPISVPLKTEPPV
TIPRAAIPPEDSPSPPLPPAPAGDGDSDSSKKWVIVGIVVGVVVLLILGAALFYLSKAFS
GSTTTKATIPTTQSWSLSSEGVRYAIESLSVYKFEELQKATGFFGEENKIKGSVYRASFK
GDYAAVKILKGDVSGEINLLRRINHFNIIRLSGFCVYKGDTYLVYEFAENDSLEDWLHSG
SKKYENSTSLSWVQRVHIAHDVADALNYLHNYTSPPHVHKNLKSGNVLLDGNFRAKVSNL
GLARAVEDHGDDGGFQLTRHVVGTHGYMAPEYIENGLITPKMDVFAFGVVLLELLSGREA
VVGGDQNGSGEKMLSATVNHVLEGENVREKLRGFMDPNLRDEYPLELAYSMAELAKLCVA
RDLNARPQISEAFMILSKIQSSTLDWDPSDELERSRSVGQISDSSR*

>Glyma.03G193700|Glyma.03G193700.1|40454287|LysM
MAEKISWCCVLFMAIILVLSCCESSTNEFRVQMLMQRNINNNNNKKACDEIYVVREGETL
QTISEKCGDPYIVEENPHIQDPDDVFPGLVIKINPFTNR*

>Glyma.04G250300|Glyma.04G250300.1|51681851|LysM/LysM
MPNPKHIILQSFVFLCLLLQASSKSTIEPCSNSDSCNALLGYTLYTDLKVSEVASLFQID
PISLLTANAIDISYPDVEHHILPSKLFLKIPISCSCVDGIRKSVATHYKTRPSDTLSSIA
DAVYAGLVSSDQLREANSISDPSVLDVGQNLVVPLPCTCFNGSDNSLPAIYLSYVVRPVD
TLAAIAARYFTTLTDLMNVNAMGSTAINDGDILAVPIPACASNFPKSASDFGLLVPNGSY
AITAGHCVQCSCGPKNLDLYCMPASLAVSCSSMQCRGSNLMLGNVTVQQTSGGCNVTSCN
YDGIVNGSIVTTLSPSLQPRCPGLQEFPPLVAPPTTVARDTVFAPAPAPLFDGAGPASPK
SSLVPSAGLPGGFSPANGPISGISSGASVACSLVKPLPTLTNALVLLLVKLMIPVAL*

>Glyma.05G133700|Glyma.05G133700.1|32660003|LysM/LysM
MFAGKGAVWFSRAVALAAIAVVACVGLTQAQPEARFNCNSANIPTCRALISYSHPNTTTL
GDIQKLFNVKHILDIVGANNLPSNATKTYAVGPNEVVKVPFPCRCSNNTGLSDRVPLYRI
KKGDTLYYIATTTFAGLMKWPQIQVANNIANANNITTGDMLYIPLPCSCDEVGGKSVVHY
AHLVAPQSTVEGIAEEFGTTQQILLNLNGISDPKNLQAGQILDVPLQACSSNVKNDSLDY
PLLVPNATYAYTAHECVKCKCDSSNNFILQCEPSQLKPTNWSVCPSMECSANVLIGKTIS
SDSCNRTTCAYTGYRFHNISAEAVTENTCAVPPTPSGSGGSTGSDSGASRSTLQGLFWSN
LFIVIHFVLFLVYVL*

>Glyma.06G112500|Glyma.06G112500.1|9126878|LysM/LysM
MPNPNPKHLFHATLFLSILLQALSKSTIEPCSNSDSCNALLGYTLYTDLKVSEVASLFQI
DPIALLTANAIDISYPDVEHHILPSKLFLKVPISCSCVDGIRKSVATHYKTRPSDTLSSI
ADAVYAGLVSSDQLREANSLSDPSVLDVGQNLVVPLPCTCFNGSDNSLPAIYLSYVVRPV
DTLAAVAARYFTTLTDLMNVNAMGSTAINDGDILAVPIPACASNFPKSASDFGMLVPNGS
YAITAGHCVQCSCGPKNLDLYCMPASLAVSCSSMQCRGSNLMLGNVTVQQTSGGCNVTAC
NYDGIVNGSIVTTLSPSLQPQCPGLQEFPPLVAPPTTVARDTVFAPAPAPLFDGDGPASP
KSSLVPSTGLPGGFSPANGPISGISSGASAACSLVKPFPALTYALVLLLVKLMIPVAL*

>Glyma.07G015300|Glyma.07G015300.1|1208761|LysM/protein kinase
MMVLVQKPHWGLLLLFLLFQLHLHCSTCYPTEPMNCTDTSRVCTSFMAFKRGPNHTLALI
ESMFDVLPGDITVEGNGWGYMFIRKNCSCAAGIKKYVSNTTFTVKSNEGLVYDMVMDAYD
GLAFLPNTTRMARNGAVVSLRLFCGCSSGLWNYLVSYVMRDGDSVESLASRFGVSMDSIE
SVNGIGNPDNVTVGSLYYIPLDSVPGDSYPLNNAAPTVPVLSPSFDNFSADQVNHKAHVP
YGWIVGGLGVALVLIILTVILCVCLRSSNCFADTRTHEKDAEGKISHKFHILRNPSFFCG
SGRYICGKHVDQKQTDGESSNHTIMVPKASTLWPDVFDMDKPVVFTYEEIFSTTDGFSDS
SLLGHGTYGSVYYSLLRDQEVAIKRMTATKTKEFMLEMKVLCKVHHANLVELIGYAASHE
ELFLVYEYAQKGSLKSHLHDPQNKGHSPLSWIMRVQIAIDAARGLEYIHEHTKTHYVHRD
IKTSNILLDASFRAKISDFGLAKLVGKANEGEISTTKVVGTYGYLAPEYLSDGLATTKND
VYAFGVVLFEIISGKEAIIRSEGTMSKNADRRSLASIMLGALRNSPDSMSMSSLREYIDP
NMMDLYPHDCVFKLAMLAKQCVDEDPILRPDMRQVVISLSQILLSSVEWEATLAGNSQVF
SGLVQGR*

>Glyma.07G079600|Glyma.07G079600.1|7237783|LysM
MMASNYPRKGLALTSSKAIANAASWYCAFVLVALALLSMFKDSSMLIPSQYASGQLLSRP
CEEIYVVGEGETLHTISDKCGDPFIVENNPHIHDPDDVFPGLVIKITPSLRT*

>Glyma.07G190500|Glyma.07G190500.1|35821203|LysM
MAKSTITSFALIFSSLLIMVLIAESGSTPTVADPICNTVHGVEEAETCTSIVQSFNLDER
HFLDINPNINCNLIFVGQWVCVDGKVI*

>Glyma.08G041000|Glyma.08G041000.1|3249248|LysM/LysM
MRNYMQFLNPQILCFLFFSALLLCLVETKSTIEPCNSSNSCPSLLSYLLPWDSKLSEIAT
RFNVNFFDILASNSLFPITPSSAHQILSAKSQVKIPISCSCVDGIRRSMSTIYTVHAADT
LASISEGYGGLVSAEQIKIVNAINATNPLTYRGTLVIPLPCTCFDNVNNGGNAIYMSYVV
QRRESLGSIATKFGTTVSDLETVNGFGEATVDPGDILSIPIAACSSATLNWYDESMIVPN
GSYTLTATNCIKCTCEPTDITLQCVPSGLDVPCYNLRCKGSNLIIGDQCVDLSQTACNVS
QCVYRGHRGGKILSSMKKSSYLECPDSLCHSGPSCWPSSSPYPEDPFGMSPKPSPSLPLP
VSKAALRTLASSGRLGPFLFNVLQLFLLKFIIYFLI*

>Glyma.08G058700|Glyma.08G058700.1|4490689|LysM
MMKFSSISFALILSFLLITMFIAESRPTPTVADPVCSIIHGVEEGETCFTITQRFILQER
QFLEINPNINCNTIFVGQWVCVNGKVN*

>Glyma.08G200800|Glyma.08G200800.1|16246816|LysM/protein kinase
MMLLLQKPHWGLLLLFLLFQLHSSTCYPTEPMNCTDTSRVCTSFMAFKPGPNHTLALIQS
MFDVLPGDITVEGTGWGYMFIRKNCSCAAGIKNYVSNTTFTVKSNEGLLYDMVMDAYDGL
AFLPNTTRMARNGAVVSLTLFCGCSSGLWNYLVSYVMRDGDSVESLASRFGVSMDSIESV
NGIGNPDNVTVGSLYYIPLDSVPGDPYPLNNAAPPVPVPSPSFDNFSADQVNHKAHVPYG
WIVGGLGVALVLIILTVILCVCLRSSNCFADTRTHEKDAEGKVSHKFHILRNPSFFCGSG
RYICGKHVDKKQTDGESSNHTITIPKASTLGPDVFDMDKPVVFTYEEIFSTTDGFSDTSL
LGHGTYGSVYYSLLRDQEVAIKRMTATKTKEFMSEMKVLCKVHHANLVELIGYAASHEEL
FLVYEYAQKGSLKSHLHDPQNKGHSPLSWIMRVQIALDAARGLEYIHEHTKTHYVHRDIK
TSNILLDASFRAKISDFGLAKLVGKANEGEISTTKVVGTYGYLAPEYLSDGLATTKSDVY
AFGVVLFEIISGKDAIIRSEGTMSKNPDRRSLASIMLGVLRNSPDSMSMSSLREYIDPNM
MDLYPHDCVFKLAMLAKQCVDEDPILRPDMRQVVISLSQILLSSVEWEATLAGNSQVFSG
LVQGR*

>Glyma.08G283300|Glyma.08G283300.1|38947605|LysM/protein kinase
MISFTNLLSLLFPLFTTSFVRVFASEVSIKTTNLSPLNCSSKIRTCNASLYHISQNLTIE
QIASFYSVISSQITPIMHGIKQDYLIRVPCSCKNTSGLSGYFYDTTYKVRPNDTFANISN
LIFSGQAWPVNHTLQPNETLAIHIPCGCSESKSQVVVTYTVQPNDTPMMIANLLNSTLAD
MQNMNKVLAPNIEFIDVGWVLFVPKESKGLLLLPSATKKKHNKWTTIIIGILGGMTLLSI
VTTIILILRRNKVDKISIEDSRLISGRSIANKTISSKYSLHKEFVEDLISFESERPLIYN
LEDIEEATNNFDESRKIGSGGYGSVYFGILGNKEVAVKKMRSNKSKEFYAELKVLCKIHH
INIVELLGYANGEDYLYLVYEYVPNGSLSDHLHNPLLKGNQPLSWSARVQIALDAAKGLE
YIHDYTKARYVHRDIKTSNILLDNKFRAKVGDFGLAKLVDRTDDENFIATRLVGTPGYLP
PESLKELQVTPKTDVFAFGVVLSELLTGKRALFRESHEDIKMKSLITVVNEIFQDDDPET
ALEDAIDKNLEASYPMEDVYKMTEIAEWCLQEDPMERPEMRDIIGALSQIVMSSTEWEAS
LCGNSQVFSGLFSGR*

>Glyma.09G067200|Glyma.09G067200.1|6652058|EEIG1/LysM
MDFFGLQSFGFEYEKMMLSRMEAGKKGGGGSSNQKKLLKDVETMNKALYLDRNSSRSSIP
SANSRSKFTGKPQLPDPRSKSKASNDHNGENAQKDKKSIWNWRPLRALSHIRNKRFNCSF
YLQVHLIEGLPPSFDDAGLAVYWKRRDGVLVTQPAKVVQCVAEFEEKLTYTCSVYGSRSG
PHHSAKYEAKHFLLYASLLSVPEMDLGKHRVDLTRLLPLTLEELEEEKSSGKWTTSFRLM
GLAKGATMNVSFGYTVVGDNASATRDSLPKALSSRQNSFSLTPTKFDVKPRQFDGSSTMR
RATSLQYSPQASDEVKDLHEVLPLTKSALASSITSYIELDEEKLCSPLDDKTELDSFTEN
LGPIKPDAYASDLGKERLEEHATKDESTCDKPELYVFQEKLETVKPDGYFLPDFGNKNPE
QCHDNEFFVVDKGIELSSNERVKLEESIIKAPDDASMVDTVCTLGISGIQISSEDSVKHD
FLDEANDSSKDQGVVEEFASIKAPEDASTVDTSCTLGISGRQVSSEDSVEHDFLDEANGL
DTNELLMQELESALNSVSNLERVALESPKTTEAKSEHKMTKSHSLDDVTASVATEFLSML
GLDHSPMGLSSESEPESPRELLLRQFEKEALNGGFSSLFDFDMNYDSEAAGGYDASASSE
QWNFSEGVKSSSFLQDLLEEPPVESQDVRSKQRAQMLEDLETEALMRQWGLNENAFHHSP
PKDFAGFGSPIHLPPEEPPTLPPLDDGLGPFLQTKDGGFLRTMSPSIFKNSKSCGSLIMQ
VSNPVVVPAEMGSGIMEVLQCLASVGIEKLSMQAKELMPLEDITGKTMQQIAWEAMPSLE
GAERQCHLRHDPITVPDSAGVQRDLKGMPSKQKSGKFSSRTVANQTGSEFVSVEDLAPLA
MDKIEALSMEGLRIQSGMSEEEAPSNIIAQSIGDISALQGKGVDISGSLGLDGAAGLQLM
DVKDGGDGVDGIMSLSLTLDEWMKLDSGEIDDIDNISEHTSKLLAAHHANSFDFIRGSSK
GEKRRGKSRCGLLGNNFTVALMVQLRDPMRNYEPVGTPMLALIQVEREFMLPKQRIFNSV
SEIRKNYYEDDESNIVAKLKTKDTEKEEKSSEEEGGIPQFRITEVHVAGLKPEPQKKKLW
GTSSQQQSGSRWLLANGMGKSNNKLSLMKSKAASKSNAPVTTKGQPGDSLWSISSRIDGA
RGKWKELAALNPHIRNPNVILPN*

>Glyma.09G148200|Glyma.09G148200.1|36715062|LysM
MYSTKSIADSASWCCALALLCLILLGSIRDNHVPEEEDPAAAATTRGRSNLFERPCDEIY
VVGEGETLHTISDKCGDPFIVERNPHIHDPDDVFPGLVIKITPTPTYTNTKKLFKR*

>Glyma.09G148300|Glyma.09G148300.1|36733007|LysM
MRGLEPVSWFLLIVLALRLVPNIEECSSPTIDVEKHTLEEPCSEFYEVREGETLYSIAEK
CRDPQIWLWNPHVEDPDDVYPGVIVRLNLFND*

>Glyma.09G165100|Glyma.09G165100.1|38915737|LysM
MSPSNGLQNGGGGGGNGVSYIEHQVSKLDTLAGVAIKYGVEVADIKRMNGLATDLQMFAL
KTLKIPLPGRHPPSPSPGPHEEPTKSGDASSERKPLRIGQSAMKEPLQSLRLKPPQPNIS
PAMSILQKFYGLKSSNSRDTLNGTEMAVYSSSTSDHSNGEWLPKALPISDLPSASNDYPR
STNLVYDLLTGDDEYVPLAEIGDAGAEKSDEKSVRRRQKAEVDNGASTPEKIMKEGNGNG
SNGFSSTGKTLAMRPKSASRAALFPESESGWLDSIPVGLGESIFTDGFSGVRKSSSASSL
REQEKNNSAAAWPPAIWGLKPDLQAAISKPIFDGLPIPISGRRSKAALD*

>Glyma.09G253200|Glyma.09G253200.1|47309778|LysM/protein kinase
MELFHNLKILSTILLLCMFPHSLKCQQAYLNGTVYDCSDNPSVPKGYLCNGLQKSCTSFL
LFRSKPPYDSPEKIAYLLGSEASTIASINMISRNDKIPSNKSIIVPVFCSCSGNIYQHNT
PYTASKNDTYYELVKETFQGLTTCQAMMGRNYYAPVNIVIGAELTVPKLCACPTENQTAR
GITSLLVYLVNYGDTIKSIGRAYGVDEQSVLEANKLAEPQSSNRSMDLFALTPILVPLIG
KSCKENPDKFYCRCYQAPDGILKGPFCGESDGQKFPAKLVAGLGVGIGAGFLCLFLLGYK
SYQYIQKKRESILKEKLFRQNGGYLLQEKLSYGNGEMAKLFTAEELQRATDNYNRSRFLG
QGGYGTVYKGMLPDGTIVAVKKSKEIERNQIKTFVNEVVILSQINHRNIVKLLGCCLETE
TPILVYEFIPNETLSHHIHRRDNEPSLSWVSRLRIACEVAGAVTYMHFSASIPIFHRDIK
PTNILLDSNYSAKVSDFGTSRSVPLDKTHLTTAVGGTFGYIDPEYFQSSQFSDKSDVYSF
GVVLVELITGRKPISFLYEDEGQNLVAQFISLMKKNQVSEIFDARVLKDARKDDILAVAN
LAMRCLRLNGKKRPTMKEVSAELEALRKAQSSLQMSHDHEHTTSNIVQECTEESISLSLH
LESTSF*

>Glyma.09G263500|Glyma.09G263500.1|48114125|LysM
MAFNSKRAPISSSKAIADAASWCCACFLVSLLLLCIFRDISALHDDQGNLMMRSSHVLSK
PSCDEIYVVGEGETLHTISDKCGDPFIVEKNPHIHDPDDVFPGLVLKITRSQTT*

>Glyma.09G282400|Glyma.09G282400.1|49794964|LysM
MERENWREHINSNSNGNYYDRFVCDYDNGERSPAKPSSPPRLGYIEHHVSKFDTLAGVAI
KYGVEVADIRKMNSLVTDHQMFALKTLHIPLPARHPPSPYLSNGSSTPGHGNSDHSPPNQ
AHHDLLDSFQSLRIKSSERKVSPAMNSLQGYYGLKGTPSPSEDGPFPRNLPMSDRPLSHH
RKSRSLVNVILEEIMEKSDDALAAETREVGSNKWNDKLGLRHQKSVADFIRIPELLLRED
NSSSGVLPSRTGKGLALRQKAANRTTATTDSEPIGLNPAALGMGDASLIDGSSGVRKSSS
TSCLQDQDNSGSSSIWPTKMWNLKPDLQALSTAAIGKPIFDGFPKPITGRKNKAALD*

>Glyma.10G070600|Glyma.10G070600.1|7093624|LysM
MACGCSSTTLAEKISWNCSVFVALMLVLSSCESNTSDFTSQMMLHGSVTGNISNNNNKAC
DEIYVVREGETLQTISEKCGDPYIVEENPHIHDPDDVFPGLVIKINPFTNRG*

>Glyma.10G154000|Glyma.10G154000.1|38910538|F-box/LysM
MGCCCDEDDGDIFRHLMNSNFPSSSSTSATTTSSCTVISPMNSHFSALSSTDILRLIFDN
LPIPDLARASCVCRLWNSVASQRDMVTRAFVAPWKLNDVVGNPLSGSFWRDNSLAKFAVS
HHITRGDTVASLAVKYSVQVMDIKRLNNMMSDHGIYSRERLLIPISNPDILINRTCFIEL
DVYAKREVAVLYPNDLPDRRTAYVSNRISSEESNKKVLESLKRSMHVDNETAQYYWSVAN
GDPRAAFAQFSADLKWDWQAGHS*

>Glyma.11G063200|Glyma.11G063200.1|4784434|LysM/protein kinase
MHLFPFIPIIIFTLLIHNFSLILGQQPYIGLGTVACPRRGNKNSIRGYTCNGANHSCQSY
LTFRSQPIYNSVKTISTLLGSDPSQLAKINSVSMNDTFETNKLVIVPVNCSCSGEYYQTN
TSYVFQNSETYLLIANNTFEGLTTCQALENQNHNPANIYPGRRLLVPLRCACPTKNQTKK
GIRYLLSYLVNWGDSVSFISEKFGVNFMSTLEANTLTLTQAMIYPFTTILVPLHDKPSSS
QTVSPTQRISPPPSPPSSDHSSNKTWVYVVVGVVVGAIALTSVLCAVIFFKRYRKNRNKD
DSLVAVPKSFEAIEEKPQVKVNEKLSENISGIAQSFKVYNFEELQRATDNFSPSSWIKGS
VYRGVINGDLAAIKKIEGDVSKEIEILNKINHTNVIRLSGVSFHEGRWYLVYVYATNGDL
SEWIYFNNVDGKFLSWTQRMQIALDVATGLDYLHSFTSPPHIHKDINSSNILLDGDFRGK
VANLSLARCLEGGDDQFPTTRHIVGTRGYMAPEYLENGLVSTKLDVYAFGVLMLEMVTGK
EVAAILTEDETKLSHVLSGIPGERSGKEWLKEFVDPSLGENCPLELAMFVIEMIDDCIKT
DPASRPSVHEIVQSLSRTVNSSLSWERSMNVPRN*

>Glyma.11G100600|Glyma.11G100600.1|7621257|LysM
MQIQTRSMYDYNHSQMMMSSSASSPPTLGYIQHPISKLDTLAGIAIKYGVEVADIKKMNG
LVTDSQMFALKSIGIPLNGKHPPPIITGYDDNTDNSPAADNAKSLRRKSSEQKLSPVMSC
LRSHYGTKPTMKKSVSEIFSMVEYEKRASKCSENGSFYKKSPMSPQHHSHHKKSHSLANE
TLDDIMEVVKARRSDSDRSGTLIRRSYKSEANLQRIPELLLKQDCNNSNGSFSFSARSAK
GLAQRQKSGSRIALTAYSNHVV*

>Glyma.11G210200|Glyma.11G210200.1|30234588|LysM
MACGCSSTAMAEKISWNCAVFVALMLVLSSCESNTSDFTTQMMFHESVSNSNNKACDEIY
VVREGETLQTISEKCGDPYIVEENPHIHDPDDVFPGLVIKINPFTNRA*

>Glyma.13G080700|Glyma.13G080700.1|18732462|LysM/LysM
MRCKSTIEPCSNYDSCNALLGYTLYTDLKASEVASLFQIDPIALLTANAIDISYPDVEHH
ILPSKLFLKVPITRSCVDGIRKSMSTHYRTRPSDTLSSIANSIYGGLVSPDQLREANSIG
DDPSVLDVGLNLVVPLPCTCFNESDNSLPSIYLSYVVQPIDTLAAIAARYFTTFTDLMNV
NDMGTTAISDGDILVVPIPACASNFPKYASDYGLLVPNGSYTILLLVTVLYCMPSSLAVS
CSSMRCKNSNLMLGNVTVQRSSSGCNVTSCNYDGFVSGTTITSLSPSLQPRCPGLQRFHP
LIAPPTSVIRESEFAFTPSLSPSQSSQSQETGLTAPKSSVMPATRSFPGFSPANGPVSRI
ASGASATPSLANPMLVLRFAFMLLLVKLLIPLAL*

>Glyma.13G354400|Glyma.13G354400.1|44281122|LysM/protein kinase
MNLIQNPSLSLLLHLYFFLLCLHCTSSYPTAMNCTDTSRVCTSFLAFKPHQNQTLAVIQS
MFDVLPGEITVEGNGWDYIFIRKNCSCAAGMKKYVSNTTLTVKSNGGFEHDLVMEAYDRL
ALLPNTTTRWAREGGIISLSLFCSCSSGLWNYLMSYVIRDGDSVESLASRFGVSMDSIET
VNGIDNPTVGSLVYIPLNSVPGESYHLMNDTPPAPTPSPSVNNFSADQVNQKAHVPHEWI
IGGLGVGLALIILTIIVCVALRSPNCLVEAGNNAKDSSGKISNKFYVFGNPSLFCGCVKP
VDQKQTDGESSSHQITGTKTSTLIPDMLDMDKPVVFSYEEIFSSTDGFSDSNLLGHRTYG
SVYYGLLGDQEVAIKRMTSTKTKEFMSEVKVLCKVHHANLVELIGYAVSHDEFFLIYEFA
QKGSLSSHLHDPQSKGHSPLSWITRVQIALDAARGLEYIHEHTKTRYVHQDIKTSNILLD
ASFRAKISDFGLAKLVGKTNEGETAATKVVNAYGYLAPEYLSNGLATTKSDVYAFGVVLF
EIISGKEAIIQTQGPEKRSLASIMLAVLRNSPDTVSMSSTRNLVDPIMMDMYPHDCVYKM
AMLAKQCVDQDPVLRPDMKQVVISLSQTLLSSVEWEATLAGNSQVFSGLVQGR*

>Glyma.13G358900|Glyma.13G358900.1|44635509|LysM/protein kinase
MEKEGNVLWYTDTVELNSSSVARGMALVERVQVRIPLLLLLTLTPTAVAVGFRCWERTTC
PALLGYKSPVPTTIGAIQRLFNVTGVADILAANNLPEWTLPNNFTVEENRLLRIPLECDC
SGPGLYGEPSNPLVYTVRKGDNISYLATTVFSGLVASTDIQHGNNVTNLRTGQELRVPLP
CSCGKVNGLDVLHFGLTLEYGSTTLEQIAHEYHVSSQTITTSNQPHLMFGRALDIPLPVC
SSMVRKDSLDHPLLVPNGSYVYTANGCVKCYCDATKNWKLTCEPSKLRPTNWSTCPSMKC
DSSISSNMYIGDTTYSSSHNLAICAYAGYGTQTIFTTLTAVYIPPGLVHAHHTKSRWWAW
LIVIAGVFVVLIFGYLCCIIWRKCKIEADRKKKQKELLLEIGVSSVACIVYHKTKRHRKR
SKVNYEMQIFSFPIIAAATGNFSVANKLGQGGFGPVYKGVLPDGQEIAIKRLSSRSGQGL
VEFKNEAELVAKLQHTNLVRLSGLCIQNEENILIYEYLPNKSLDFHLFDSKRREKIVWEK
RFNIIEGIAHGLIYLHHFSRLKVIHRDLKAGNILLDYEMNPKISDFGMAVILDSEVVEVK
TKRVVGTYGYMSPEYVIKGIISTKTDVFSYGVLVLEIVSGKKNNSRYQADYPLNLIGFAW
QLWNEGKGVELIDSSMLESCRTAEVLRCTQVALLCVQANAADRPSMLEVYSMLANETLFL
PVPKQPAYFTDACANEKNALVGNGKSYSTNEVTISMMDAR*

>Glyma.14G077700|Glyma.14G077700.1|6572516|LysM/protein kinase
MNHHVVFLFTLTFLLLSFNAKAQQNYSGNSILSCKNDDKMGPSPSFLYTCNGFNKTCMSF
LIFKSKPPFNSITTISNLTSSNPEELARINDVTVLKVFPTGKEVIVPLNCSCLTREYYQA
ETKYVLGQSPTYFTVANDTFEGLTTCDTLMRANSYGELDLLPGMELHVPLRCACPTWHQI
TNGTKYLLTYSVNWGDSIKNIAARFNVAAGNVVDANGFSTQTQTIFPFTTVLIPLPSEPV
SSMAIIVNGPPAVSPLPVCSSEKCNSRRKLYIVIATTGGSMLVLCVVLFGGFLCRKRSAR
FIKRGEQSEKAKKLSSEDIRGKIAIIEHHSKVYKFEEIEEATENFGSKNRIKGSVFRGVF
GKEKNILAVKKMRGDASMEVNLLERINHFNLIKLQGYCENDGFPYLVYEFMENGSLREWL
SRNRSKEHQSLAWRILIALDVANGLQYLHNFTEPCYVHRNINSGNILLNRDLRAKIANFA
LVEESESKITSGCAASHVVKSRGYTAPEYLEAGMVTTKMDVFAFGVVLLELITGKDSVTL
HDGREVMLHAIIVNLIGKENLEEKVSLFIDPCLTVTGNSEIVCAPQLVKLGLACLIQEPA
ERPTMVEVVSSLLKIYTSYMEQIIPPSISNSPSMER*

>Glyma.15G019800|Glyma.15G019800.1|1540655|LysM/protein kinase
MNLIQNLPLRLLPHLYIFLLHLYCTSSYPTPMNCTDTTRVCTSFLAFKPQPNQTLAVIQS
MFDVLPGEITVEGNGWDYIFIRKNCSCAAGMKKYVSNTTLTVKSNGGFEHDLVMDAYDGL
ALLPNTTTRWAREGGVISLSLFCSCSSGLWNYLMSYVIRDGDSVESLASRFGVSMDNIET
VNAIDNPDSLTVGSLYYIPLNSVPGELYHLKNDTPSAPIPSPSVDNFSVFSAADHVTQKA
HVPHEWIVGGLGIGLALIILTIIVWVALRSPNCLVEARNNAKDSAGKISKKFYVFGNPSL
FCGCGKPVDQHQTYGESSSHQITVTKASTLMPDMLDMDKPVVFSYEETFSSTDGFSDSNL
LGRRTYGSVYHGLLRDQEVAIKRLTTTKTKEFMSEIKVLCKVHHANLVELIGYAVSHDEF
FLIYEFAQRGSLSSHLHDPQSKGYSPLSWITRVQIALDAARGLEYIHEHTKTRYVHQDIK
TSNIFLDASFRAKISDFGLAKLVGETNEGEIAATKVVNAYGYLAPEYLSNGLATTKSDVY
AFGVVLFEIISGKEAIIQTQGPEKRSLASIMLAVLRNSPDTVSMSSTRNLVDPIMMDLYP
HDCVYKMAMLAKQCVDEDPVLRPDMKQVVIFLSQILLSSVEWEATLAGNSQVFSGLVQGR
*

>Glyma.15G173100|Glyma.15G173100.1|15871521|EEIG1/LysM
MEAGKKGGGGSSNQKKLLKDVETMNKALYLDRTSSRSSIPSANSRSKFTGKPQLPDPKSK
SKASGDNNSENVQKDKKSIWNWRPLRALSHIRNKRFNCSFYLQVHLIEGLPPSFDDASLA
VYWKRRDGVLVTQPAKVVQRVAEFEEKLTYTCSVYGSRSGPHHSAKYEAKHFLLYASLLS
VPEMDLGKHRVDLTRLLPLTLEELEEEKSSGKWTTSFRLTGVAKGAAMNVSFGYTVVGDN
ASATRDSLPKALTSRQHSFAPTPTKLDVKPRQFDGSSKMRRATSLQYSSQAADEVKDLHE

VLPLTKSALASSIDVLYTKLDEEKACSPLDDEAELDSFNLGPIKPDAYASDLGKERLEEH
ATKDENTCPVDDKPEPYVFQEKLETVKPDGYSLPDFENENPEHCLDNDFFVVDKGIELSS
NESVKLEESIIKAPDDASTVDSASTLGISGIQISSEDSVKHDFLDDANDSSKDQAVVEEF
ASIKAPEDASTVDASCTLGISASPSTPRELLLRQFEKEALNGGFSSLFDFDMNYDNEADG
GYDASAASEQWNFSEGVKSSSFLQDDLQEEHPVESQDVRSKQRAQMLEDLETEALMREWG
LNEKAFHHSPPKDFAGFGSPIHLPPEEPPTLPPLDDGLGPFLQTKDGGFLRSMNPSIFKN
SKSGGSLIMQVSNPVVVPAEMGSGIMEVLQCLASVGIEKLSMQAKELMPLEDITGKTMQQ
IAWEAMPVLEGAERQCHLQHDPIAWPDSAYVQRDLKGMPSKQKSGKFSSRTVANQTGSEF
VSVEDLAPLAMDKIEALSMEGLRIQSGMSEEEAPSNIIAQSIGDISALQGKGVDVSGSLG
LDGAAGLQLMDVKDSDDGGDGVDGIMSLSLTLDEWMKLDSGEIDDIDNISEHTSKLLAAH
HANSFDFIRGSSKGEKRRGKSRRCGLLGNNFTVALMVQLRDPLRNYEPVGTPMLALIQVE
REFMLPKQRIFDSVSEIRKNYDEDDESEIVAKVEMKDTEKEEKSSEEDEGIPQFRITEVH
VAGLKPEPQKKKLWGTSSQQQSGSRWLLANGMGKSNNKLSLMKSKAASKSNAPVTTKGQP
GGDSLWSISSRIDGARGKWKELAALNPHIRNPNVIIPN*

>Glyma.15G212300|Glyma.15G212300.1|33098200|LysM/protein kinase
MRAGFKYLLTYLVSQGESVSSIVDIFGIDEQNILEANELSATSIIFYFTPISVPLKTEPP
VGIQRATTPPEDSPLPPPRPAPAEDRDSDSSKKWVIVGIVVGVVVLLILSAALFLLRFYQ
LRQVEHPSLPPPKAFSGSATMKATIPMMHSWSVSSEGVRYAIKSLSVFEFEELQKATGFF
GEENKIKGSVYRASFKGDYAAVEVLKGDTYLVYRFAENDSLEDWLHSVNKKYENSVPLSW
VQRVHIAHDVADALNYLHNYTSPPHVHKNLKSGSVLLDRNFRAKFSNFGLARAVEDQGGD
GGIQLTKHVVGTQGYMPPEYIENCLITPKMDVFAFGGVLLELLSGNVKEKLGGFMDPDLR
YEYPLELAYSMAEHAKRCVARPQISEVFMILSNIQYSTLDWDPSDELEWSRSVSQISDRR
*

>Glyma.16G199900|Glyma.16G199900.1|36101914|LysM
MYSTKSIADSASWCCALALLCLILLGSIRENHVPEEDEATARGRSNLFERPCDEIYVVGE
GETLHTISDKCGDPFIVERNPHIHDPDDVFPGLVIKITPTPTYTNTKKLFKR*

>Glyma.16G200000|Glyma.16G200000.1|36107537|LysM
MEECSSPAIDVEKHTLEEPCKEFYEVRQGETLYSIAEKCRDPHIWLWNPHVEDPDDVYPG
VIVRLKPVR*

>Glyma.16G213400|Glyma.16G213400.1|37145525|LysM
MSPSNGLHNGGGGGSGGGGGNGVSYIEHQVSKLDTLAGVAIKYGVEVADIKRMNGLATDL
QMFALKTLKIPLPGRHPPSPAPGPHDEPAKSGEASIERKPLRIGQSAMKEPLQSLGLKPP
QPNISPAMSILQKFYGLKSSNSRDTLNGTEMAVYLSSNSDHSSGEWLPKASPILDLPSAS
NDYPRSTNLVYDLLTGDDEYVPLAEIGDTGVEKSDEKSVRRRQKAEVDNGTSTPEKIMKE
GNGNGSNGLSSNGKTLAMRPKSASRAALFPESESGWLDSIPVGLGESIFTDGFSGVRKSS
SASSLREQEKNNSAAAWPPAIWSLKPDLQAAISKPIFDGLPIPISGRRSKAALD*

>Glyma.17G051700|Glyma.17G051700.1|3922350|LysM
MSHLEYQCFLLQVERVFVPPKQKIYRHVSEAGKNNHECEIVAKVEMKANKEEKNSEEEAI
HQFRITEVHVAGLKTEPLKKKFWGTSRRQQQQQSGSRWLIANGMGKNNKNSLVKSKVVSK
SSAPITTANVQPGDTLWSISPRIYGTGTRWKELKELNPHIRNPNIKIPNKTSTWSARFNF
LL*

>Glyma.18G228600|Glyma.18G228600.1|51766615|LysM
MAFNSKRAPISSSKALADAASWCCACLLVSLLLLCIFRDSSALHDDQGNLMIRSSHVLSK
PCDEIYVVGEGETLHTISDKCGDPFIVEKNPHIHDPDDVFPGLVIKITPS*

>Glyma.18G239600|Glyma.18G239600.1|52837347|LysM/protein kinase
MELFHNLNIFSTILLLCMFPHSLKCQQAYLNGTVYDCSDNPSAPKGYLCNGLQKSCTSFL
LFRSKPPYDSPGIIAYLLGSEASTIASINRISRNDKIPSNKSIIVPVFCSCSGNIYQHNT
PYTASKNDTYYELVKETFQGLTTCQAMMGQNYYASINIAIGAELTVPMLCACPTENQTAR
GVTSLLVHLVNYGDTIKSIGRAYGVDEQSVLEANKLAVSQSKNSSMDLLALTPIIVPLIG
KSCKENPDKFYCRCYQAPDGSSKGPFCDESDGQKFPAKLVAGLGVGIGAGFLCLFLLGYK
SYQYIQKKRETILKEKLFRQNGGYLLQEKLSSYGNGEMAKLFTAEELQRATDNYNRSRFL
GQGGYGTVYKGMLLDGTIVAVKKSKEIERNQIQTFVNEVVVLSQINHRNIVKLLGCCLET
ETPILVYEFIPNGTLSHHIHRRDNEPSPSWISRLRIACEVAGAVAYMHFAASISIFHRDI
KPTNILLDSNYSAKVSDFGTSRSVPLDKTHLTTAVGGTFGYIDPEYFQSSQFSDKSDVYS
FGVVLVELITGRKPISFLYEDEGQNLIAQFISLMKENQVFEILDASLLKEARKDDILAIA
NLAMRCLRLNGKKRPTMKEVSTELEALRKAQSSLQMNHDHEHTTSDIVQECTEESMSLPL
HLEFTSF*
>Glyma.18G239700|Glyma.18G239700.1|52842315|LysM/LysM/protein kinase
TVYDCSENPSAPKGYLCNGLQKSCTSFLVFRFKSPHDNPVSLAYLLGSEASTIASINKIS
RDDKIPSNKSIIVPVFCSCSGNIYQHNTPYSATKNDTYFKLVTETYLGLTTCQALMGQNY
YASDGIRVGSELTVPVVCACPTENQTERGITSLLVYSVKNGDTIKSIGEVYGVDEQSMLE
ANGLPVPTSTENSIIIYALTPILVPLRGKSCKEDPDSFYCTCSQGMLAVVDLTFTGFHCN
ESEGNNFPAKLVASLGVGIGAGFLCLFLLGYKLYQYIQKKRKSNRKEKLFRQNDGYLLQE

KLSFYGNREMAKLFTAEELQRATDNYNRSRFLGQGGQGTVYKGMLLDGTIVAVKRSKKIE
RNQIETFVNEVVILSQINHRNIVKLLGCCLETEAPIIIYEFIPNRTFSHHIHGRQNEPSL
LWDTSIPIFHRDIKPTNILLDSNYSAKVSDFGTSRSVPLDKTHLTTDVGGTFGYIDPEYF
QSGQFSDKSDVYSFGVVLVELITGRKPISFLYKHEGQNLIAEFISSVRQNQVYEILDARV
LKEGRKDDILAAANLAMRCLRLNGKKRPTVKEVSA*

>Glyma.19G088300|Glyma.19G088300.1|31430957|LysM/protein kinase
MGTVEMIIAFHMKALVFFLWLFVPSLGKDLLSCETTSPDASGYHCIENVSQNQCETFALF
LTNSYYSSLSNLTSYLGLNKFVIAQANGFSADTEFLSQDQPLLVPIHCKCIGGFSQAELT
KTTVKGESFYGIAQSLEGLTTCKAIRDNNPGVSPWNLDDKVRLVVPLRCSCPFSSQVRPQ
PKLLLSYPVSEGDTISNLASKFNITKEAIVYANNISSQGLRTRSSLAPFTSILIPLNGKP
IIGPLVKPKEPDSGNQTTSIPVTSPHKKSPMWKTELCIGLAGVALGVCIAFAAAFFFIRL
KHKKEEENSCKEGDLELQYLNQSVRTTSTSDKKVSFEGSQDALDVKIVDALPRKLLLDTY
TIEDVRKATEDFSSSNHIEGSVYHGRLNGKNMAIKGTKAEVVSKIDLGLFHDALHHHPNI
LRLLGTSMLEGEQQEESFLVFEYAKNGSLKDWLHGGLAIKNQFIASCYCFLTWSQRLRIC
LDVAGALQYMHHVMNPSYVHRNVKSRNIFLDEEFGAKIGNFGMAGCVENDTEDPQFYSTN
PASWSLGYLAPEYVHQGVISPSVDIFAYGVVLLEVLSGQTPISRPNEKGEGSIWLTDKIR
SILVSENVNELRDWIDSALGENYSFDAAVTLANIARACVEEDSSLRPSAREIVEKLSRLV
EELPQGENDMLMCESSSKPLVKAVENNVE*

>Glyma.19G193600|Glyma.19G193600.1|45125361|LysM
MAEKIYWCCVVVFVELVLVLSGCESSTNEFSVPMLMQMNINKACDEIYVVREGETLQTIS
NKCGDPYIVEENPHIQDPDDVFPGLVIKINPFTNR*

>Glyma.20G003100|Glyma.20G003100.1|288005|LysM
MERENWREHINSNGNYYDDDGVERSPAKPSSLSSSPPRLAYIDHRVSKFDTLAGVAIKYG
VEVADIRKMNNLVTDHQMFALKTLHIPLPGRHPPSPCLSNGSSTPGHGNYDHSPPSQAHH
DLLDSFESLRIKSSERKVSPAMNSLQGYYGLKGTPSPSEDGPFPRNLPMSDRRLSRHRKS
RSLVNVILEEIMQKSDDAPAAETREINSNKWNDKLGQGHQKSVADFTRIPELLLREDNSS
SGVLSSRIGKGLALRQKAANRTAATIDSEPIGLNPAVLGMGDTSLNDGSYGVRKSSSTSC
LQDQDTSGSSSIWPTSMWSLKPDLQALSSAAIGKPIFDGLPKPITGRKNKAALD*

>Glyma.20G154900|Glyma.20G154900.1|39375162|LysM
MYIYIYRVEACLFNSICDNMNMGSSAADSASWYCAMVLLATILLGSIRESTVAEEGEPIK
GNNLLQRPCDEIYVVGEGETLHTISDKCNDPFIVERNPHIHDPDDVFPGLVIKITPIHQY
*

>Glyma.20G234300|Glyma.20G234300.1|46690568|F-box/LysM
MKVYPLKDLLFSRNLTPLPMGCCCDEDDGDIFRHLMNSNFPSSSSSTSSSCTVISPMNSH
FSALSSTDILRLIFENLPIIDLARASCVCRLWCSVASQRDMVTRAFVAPWKLNDVVGNPL
SGSFWRDNSLAKFAISHRITRGDTVASLAVKYSVQVMDIKRLNNMMSDHGIYSRERLLIP
ISNPDILINRRCFIELDVYAKREVAVLYPNDVPDRRTTYVSNRISSEESNKKVLESLKRS
MHVDNETAQYYWSVANGDPRAAFAEFSADLKWDWQAGHS*

Nictaba

>Glyma.03G189500|Glyma.03G189500.1|40038404|F-box/Nictaba
MGASLSNLGSNGSAAAPGLGDIPENCVARVFLHLTPPEICNLARLNRAFRGAASADSVWQ
TKLPRNYQDLLDLMPPERHRNLSKKDIFALLSRAVPFDDGNKEVWLDRVTGRVCMSISAK
AMSITGIDDRRYWTWVPTEESRFNTVAYLQQIWWFEVDGEFSFPFPADIYTLSFRLHLGR
FSKRLGRRVCSYEHTHGWDIKPVRFELSTMDGQQASSECYLDETEPDDLHGNHKRGHWVD
YKVGEFIVSGSEPTTKVRFSMKQIDCTHSKGGLCVDSVFIIPRDLRERKRSGILK*

>Glyma.03G233800|Glyma.03G233800.1|43456534|F-box/Nictaba
MSHMASSCSFNIESLPHDCVSEILSHTSPLEACMVSLVSPTLRSCANSDTVWRSFLPSDY
EDIVSSAVNPFILSFSSYKQLFHALCHPLLIDQGNKSFKLEKSSGKKSYIISARELSIAW
SSDPMMWSWKPIPESRFPEAAELRTVSWLEIEGKIRTRILTPNTSYSAYLIMNVSHRAYG
LDFAPSEVSVTVGKNVRRGKAYLGHKDENKRKMETLFYGNRTEVLRNAAFDEGIASPSKR
EDGWMEIELGEFFNGEGDEEIKMCLREVGYQLKGGLVLEGIQLRPKP*

>Glyma.03G233900|Glyma.03G233900.1|43459075|F-box/Nictaba
MELLPYDCFAHILSFTSTQDVCRSSLVSSIVQSMADSDAVWEKFLPLNHQEIVSRLVPPS
LLCSSKKELFVKLCKPRPIDDGNKMLSIEKTTGKICYLLSARQLSITWGNSSMYWSWKPI
KGPRSRQRQKNLFIAFSNHRTVVVEKGAESNAEGVGIR*

>Glyma.03G253900|Glyma.03G253900.1|44924165|F-box/Nictaba
MDNKSRNKVMVLEAEEEVGGGEFEHLPEGCIANIVSFTTPPDACVLSLVSSSFRSASVTD
FVWERFLPSDYQAIISQSSKPSTLTNYSSKKDLYLHLCHNPLLIDAGKKSFALDKLNGKI
CYMLSARSLSIVWGDTPRYWRWTSVPAARFSEVAELVSVCWLEIKGGIKSGTLSEKTLYG

AYLVFKQRSGGAYGFYNQPVEVSVEGRRRTVYLEEAETPRRPREQIVPGIFSRVRSRFLD
SFDAAPPPPPPNAKGGGEYPKERSDGWMEVELGDFFNVGGEKEKEKEVEIGVYEVKSGGW
KAGILVQGIEIRPKHKN*

>Glyma.05G049400|Glyma.05G049400.1|4412561|F-box/Nictaba
MRVHSVKSKSDTAVWRKQRKAQNQVPLLTYKLTTFSFRHIKSTIISHFHFNVTLMGLRFS
SSEAAASESASPSSSLGELPESCVAQIMTYMDPPQICKLATLNRAFRGASSADFVWESKL
PPNYDILLRRIFADFPSHLGKRGIYARLCRLNSLDDGTKKVWLDRGMGKLCLCVSAKGLS
ITGIDDRRYWNHIPTDESRFSSVAYLQQIWWFQVDGEVEFPFPAGKYSVFFRIHLGRAGK
RFGRRVCNTEHVHGWDKKPVRFQLWTSDGQYVASQCFLNGPGKWIFYHAGDFVVEDGNAS
TKVKFSMTQIDCTHTKGGLCLDSVLVYPSEFRKVKAFLNYS*

>Glyma.06G221100|Glyma.06G221100.1|26785124|Nictaba
MPFKKPHHTSDKNYITGDDGGKFEIQPRGLNIVWGNDSRYWKIPEQGPAELIQVSWLEVS
GVVNLPGVKKYRVEFEVRVKDDGFGWSGTDVLVMAKIGKTGKYTYKVTKLNPGETLNIPK
STDPLEIQVNKQSEDLHFGLYEVWSGKWKGGLEIVRALIKPLT*

>Glyma.06G270800|Glyma.06G270800.1|46003360|F-box/Nictaba
MDTITKVLPEECVATIISLTSPKDACQLSLVSPSFKEIADSDAVWANFLPSDCEDIIDQS
STPTLNLLSKKQIYAYLCDYHVLFDNGNMTLSLEKATGKKCIIVSAKGFKISWGDKPCYW
YWESTPESRFSEVAMLKYLWWLEILGSLEAKFLSANTTYGVYFIFNFENHGSEFIYLNQN
SQPRTYGDLVVCEGNINGYRKRVCLDPPGEEVHEREDGWMEVEMGEFFSGDHEDNLVDFK
LRDINSQLTHFLVVEGVEFRPKNM*

>Glyma.06G271000|Glyma.06G271000.1|46016701|F-box/Nictaba
MDTITKVLPEECVATIISLTSAKDACQLSPVSPSLKAIADSDAVWANFLPSDCEDIIDQS
STPTLNLLSKKQIYAYLCDYHVLFDNGNMTLSLEKATGKKCIMVSAKGFKISSGDKPCYW
YWESTPKSRFYEVAMLKYMWWLEILGSLEAKFLSANTNYGVYFIFNFENHGSEFIYLNQY
FQPRTYGDLVVCEGNINGYRKRVCLDPPGEEVHEREDGWMEVEMGEFFSEDHEDNLVGFK
LWDMNSQLTRFLVVEGVEFRPKNM*

>Glyma.07G222500|Glyma.07G222500.1|39833031|Nictaba
MGGSRLSKPKSGSASGSTNCFMLYARALSITWGDTPEYWIWVQQKETSGTIVELAKLKRV
CWLEVHGKFDTRKLSLGILYQVSFLIMLEESSQGWEVPINVRFVLPGGKRQQHKVNLNEK
LRESWMEILVGEFVASEKDAGEMEISMYEYEGGMWKTGLVIQGVVIKPKN*

>Glyma.10G150400|Glyma.10G150400.1|38573748|F-box/Nictaba
MDTLPEDCVSKILSYTSPPDACRFSMVSSTLRSSADSDLLWRTFFPSDYSDIVSRALNPL
SLNSSSSYKHLFYALCHPLLLDGGNMSFKLDKSSGKKSYILSARQLSITWSNDPLYWSWR
PVPESRFKEVAELRTVSWLEIQGKIGTRILTPNTSYVVYLIMKTSHREYGLDSVACEVSI
AVDNKVKQSGRVYLCQNEKDENNLKKESIGIPMRREDGWMEIEMGEFFCGEADEEVLMSL
MEVGYQLKGGLIVEGVEIRPKIIV*

>Glyma.10G169300|Glyma.10G169300.1|40354794|F-box/Nictaba
MKSQDLPEGCVAHILSYICTPEDIVRLSLVSKAFYSAADYDTVWDRFIPSDFSSTISPLS
SSNSKKDLYFTLSDRPTIIDQGRKVRTLFLLACSDVFYGIPEMKCVVYVASLKFLQSFQL
EKRTAKKCYMLSARDISITWAPTQGEASQYWEWKSLPESRFQEVARLYAVCWFNITGQIK
TRVLSPNTQYAAFLVFQMIDASGFHHHPAMLSVSNVGGSRTSKYVCLDPNLEDNDLDDRF
RGLQRPNVRKDKWLEIEMGEFFNSGLEEDEIYMNVRETSDMWKHGFILEGIEVRPKHV*

>Glyma.10G169600|Glyma.10G169600.1|40364491|F-box/Nictaba
MEFQGLPEGCIASILSRTTPADVCRFSVVSKIFRSAAESDAVWKRFLPSDYHSIISQSPS
PLNYPSKKELYLALSDRPIIIDQGKKSFQLEKKSGKKCYMLAARALSIIWGDTEQYWNWT
TDTNSRFPEVAELRDVCWLEIRGVLNTLVLSPNTQYAAYLVFKMIDARGFHNRPVELSVN
VFGGHGSTKIVCLDPNEELPHRRVEGLQRPNARSDGWLEIEMGEFFNTGLDDEVQMSVVE
TKGGNWKSGLFIEGIEVKPKEEN*

>Glyma.14G073300|Glyma.14G073300.1|6158677|F-box/Nictaba
MELQDLPEGCIAKILSYTTPVDVCRLSLVSKAFRSAAESDTVWDCFLLSDFTSIIPISST
SKKDLYFTLSDHPTIIHQGRKSVQLDKRTGKKCCMLSARNLTIIWGDTVQHWEWTSLPES
RFQEVAMLQAVCWFDISGSINTLTLSSNTHYATFLVFKMINASGFHYHPTVLSVGVLGGN
SNTKYVCLDPNLKGNHRLQELQFPKVRSDGWLEIEMGEFFNSGQEEKQVQMKVMETTSHI
WKCGFILEGIEIRPKHV*

>Glyma.17G251700|Glyma.17G251700.1|40625907|F-box/Nictaba
MELQDLPEGCIAKILSYTTPVDACRLSVSIAFRSAAESDTVWDCFLLSDFTSFIPPSSTS
KNDLYFTLSDLPTIMDQGRKSVQLAKGPGRSVTCFPLEIWPLYGVILFDIGSGQACQSPT
RFQEVAMLRAVCWFDISGSINTLTLSSNTHYSASLVFKMINPSGFHYHPTVLSVGIFGGN
SNTKYVCLDPNLKDNHRFQELQCPKVRSDGWLEI*

>Glyma.19G231000|Glyma.19G231000.1|48171303|F-box/Nictaba
MNHMASCSFNIESLPHDCVSEILSHTSPLVACIVSLVSPSLCSCANSDTVWRSFLPSDYE
DIVSRAVNPFTLSFSSYKQLFYSLCHPLLIDQGNKSFNLEKSSGKKSYIISARELSIAWS
SDPMMWSWKPIPESRFAEAAELRTVSWLEVEGKIRTRILTPNTSYLAYLIMNVSHRAYGL
DFAPSEVSVMVGNKVHRGKAYLGDKDENKREMEALFYGNRTEVLRNAAFQEGIPFPSKRE
DGWMEIELGEFFSGEGDEEIKMSLREVGYQLKGGLVLEGIQVRPKTS*

>Glyma.20G020900|Glyma.20G020900.1|2139622|Nictaba/Nictaba
MGASQSQEELQSQRQFQQHQHPNELTRRESLLLSPAASNNTKQFSNSTKVVDNVTAANST
NTNCFMLNARALSITWAENPDYWTWVQDKDESGTMIELPNLKMVCWLEVHGKFDTRKLSL
GILYQVSFIVMLKDSAQGWEVPINVRLVLPGGKKQQHKENLNEKLRECWIEIPVGEFVAS
EKDVGNIEISMYEYEGGKWKTGLIIQGIAIKPKNETNQSQHHQQPHELTRRESWLMSPAG
NSKYFKNSTKAIDNVTSANNTNSFMVYARSLSITWAENPNYWKWVQHKEASGTMTELAKL
KMVCWLEVHGKFDARKLLPGIPYQVLFIVMLKDSAQGWEVPINFRLVLPGGKKQEHKENL
NKLRESWIHIPVGEFVASEKDVGNIEISMYEYEGGMWKTGLIIQGIVIKPKN*

>Glyma.20G021000|Glyma.20G021000.1|2146166|Nictaba
MGGCLSSKPKAVDNGSASGNINCFMLYARALSITWGETPEYWIWVQQKEASGTIIELAKL
KKVCWLEVRGKFDIRKLLPGILYQVSFLIMLEDSSEGWEVPINVRLVLPGGKRQQHKVNL
NEKLRENWMEILVGEFVASEKDGGEMDISMYEYEGGMWKSGLVIQGVVIKPKN*

>Glyma.20G021200|Glyma.20G021200.1|2154903|Nictaba
MGGCLPSKPNPEDNGSASSSTNRFMLYARALSITWGDTPEYWTWVQQKEEASGTIVELAK
LKSVCWLEVHGKFDTRKLSAGILYQVFFLIMLEESSQGWEVPINVGFVLPGGKRQQHKVN
LNEKSRESWLEILVGEFVASKKDVGEMKISLYEYGGMWKTGLVIQGVVIKPKN*

>Glyma.20G021500|Glyma.20G021500.1|2178687|Nictaba
MGASESHEKLQSQQPQPQSQHVQHQQPQPQLHEPTRRRESSLLPPDQGNTKASKDRTKAV
VDNNATMMPVKSLKGLPIPHNYEHILKSNADSPVDKSLLDKLYAGVFLDHKTKKYWVEKK
SNGNCFMLYARALSITWAENPNYWKWVQHKEESGSMIELAKLKMVCWLEVNGKFGTGMLS
PGILYQVSFIVMLKDSAQGWELPINVRLVLPGGKKQQHKENLMEKSRESWIEILVGEFVA
SEKDVGEMEISMYEHEGGMWKTGLVIEGVAIKPKN*

>Glyma.20G220100|Glyma.20G220100.1|45545826|F-box/Nictaba
MTQSVVVDFNNLPEGCIANILSFTSPRDVCRLSLLSSTFRSAAQSDAVWNKFLPSDFHTI
LSQSSSLSLPSKKDLFLYLCQKPLLIDDGKKSFQLDKVYGKKCYMLSARNLFIVWGDTPR
YWRWTSLPDARFSEVAELRSVCWLEIRGWINTGMLSPETLYGAYLVFKPNPSGFYGFDYQ
LVEVSIGIAGGENRKRNVFLDAERGRRLRYQIVPRRAGTGIFNRARFLAPVEAPPVEDND
SLDLQHPKERADEWLEVELGEFFNDGQEDKELEMGVYEIKSGDWKGGLLVQGIEIRPKRT
PIN*

>Glyma.20G220200|Glyma.20G220200.1|45549852|F-box/Nictaba
MKFEDLAEGCMAKILSYTTRADVCRLSLVSKAFHSAAEANTVWDCFLPSDLSSIISPSSS
VPPFRSIKDLYLYLSDRPTIIDQGIKSFQLEKRTGNKCYMLSARDLSIIWGDTTHYWEWT
TLPESSRFEEVARLRAVCWFDITGRMNTRVLSPNTNYAAFLVFKMIDAGGFHYDPAVLSV
GILGGNSSTKNVCLDPNLVDNRLDDRFHGLQRPTVRSDGWLEIEMGEFFNPGLEEDELQI
KVSETTSNWWKRGFILEGIEVRPKHV*

>Glyma.20G220300|Glyma.20G220300.1|45552204|F-box/Nictaba
MGIFFSPYGKQESSGTTTINLLPEGCISYILSYTTPVDACRLSLVSKAFRSAAQSDTLWD
RFITSALSSLVSPSSFPSSHSKKHLYFALCDRPIIIHNGTKSIQLDKRTGKRCYTLSTRV
HLIDSEWGLAPLQWEHIRLPNSRFQQFGVLVSAPEAWFDISGRIKALSLSPRTEYAAFLL
FKMVYATPTEVHYHPWVLGYLVITLPDSSSVRTIDVCLLKNLQQHSCVRRSDGWLEFELV
GFFDLGLEDDQVQIIKVTDTRPNSNWKHGFILEGIEIRPKHVY*

Ricin B

>Glyma.05G173300|Glyma.05G173300.1|36277504|GH 5/Ricin
MEMGRWWSSTLVFTVLSAPILIIALLSSTFEEVDHDNTVPVTGLLHTDSRWILDQDGRRV
KLACVNWVSHLEAVVAEGLSKKPVDVISKGIKSMGFNCVRLTWPTLLVTNDSLASLTVRR
SFQSLGLLESIAGVQTNNPSIIDLSLIQAFQAVVKSLGDNDVMVILDNHVTQPGWCCGNT
DGNGFFGDKFFDPNQWILGLTKMATLFKGVTAVVGISLRNELRGSRQNVNDWYKYMVKGA
EAAHAANPDVLVILSGLNFDTDLSFLRDRPVSLTFKGKLVFEVHRYGFTDGGAWADGNPN
QVCGKVTANIKKTSGFLVDQGWPLFVSEFGGDLRGTNVNDNRYLNCFLALVAELDLDWAY
WTLVGSYYFREGVIGMEEFYGLLTWDWNQVRSTSFLNRISALQIPFRGPGIIEGNPHKLI
FHPLTGLCVISKSQLTSLTLAACSSSDAWTYTPQKTLLVNNTDFCIHAEEERKPATLSMT
CSDPNSKWEMISDSNMHLSSKLSDGSNLCLDVDDNNIIVTNACKCLSKDKTCDPGSQWFK
LIDSGRRSISTTSTLSMLNSPDLLWKSLSSI*

>Glyma.05G173400|Glyma.05G173400.1|36283079|GH 5/Ricin
MILLKKHEMVFNFVSALITTILLSGAIVEVKGLPLHTDSRWIVNEDGQRVKLACVNWVSH
LEAVVAEGLSKKPVDVISNGIKSMGFNCVRLTWPIVLVTNDSLASLTVRSSFQNLALLES
IAGVQTNNPSIIDLPLIQAFQAVVKSLGDNDVMVILDNHITQPGWCCSNSDGNGFFGDKF
FDPNQWILGLTKMASLFNGVTNVVGMSLRNELRGPKQNVNDWYKYMVKGAEAIHAANPDV
LVILSGLNFDKDLSFIQNRPVSLTFKGKLVYEAHWYAFTDGQAWVNGNPNQVCGQVAGNM
MRTSGFLVNQGWPLFISEFGGDLRGTNVNDNRYLNCFLAVAAELDLDWALWTLVGSYYFR
QGVIGMEEFYGILSWDWTQVRNTTFLNRISALQLPFRGPGITRGNPYKLIFHPLTGLCVI
RKSLLDPLTLGPCYLSDGWKYTPQKILSIKGTYFCIQAENEGMPAKLGIICSDPNSRWEM
ISDSKLHLSSKLSDDSNVCLDVDDNNNIVTNACKCLSRDRTCDPSSQWFKLIDSGRRSML
TTSTSSMLNSSDLYGNH*

>Glyma.05G174200|Glyma.05G174200.1|36323855|GH 27/Ricin
MMKCFSVSLSSLWVLLALCSLSVLSQNISQSGLQQASLPPRGWNSYDSFCWTISEEEFLQ
SAEIVSQRLKAHGYQFVVVDYLWYRKKVTGAYPDSLGFDVIDEWGRMLPDPGRWPSSIGG
KGFSDVANRVHSLGLKFGIHVMRGISTQAVNANTPILDTTKGGAYQESGRVWRAKDIAMP
ERACAWMPHGFMSVNTKLGAGRAFLKSLYEQYAAWGVDLVKHDCVFGDDLDLNEISYVSE
VLSELNRPIVYSLSPGTSVTPAMAKDVSGLVNMYRITGDDWDLWEDVKAHFDVTRDFSTA
NMIGGKGLKGNSWPDLDMLPFGWLTDPGSNEGPHRFSKLTLEEKRTQMTLWSLAKSPLMY
GGDVRKIDATTYELITNPTLLEINYFSSNNMEFPYVTSSINLKHPGGKKRRPKKGIKASF
THSLGLTGCSESKASGWSIESLNQDLERICWKNGLENKHQATFCVHKRELQFRLDGVSMY
PADYRGKHQLVATDRMKFCLDASPKRKVTSREFRRGTFSPCRWDSNQIWELNSNGTMVNS
YSGLCATVEYVEANVNSGGIRSWIATGRTGEIYLAFFNLSEQKTEIYAKTSYLAKVLPDK
SITSCKGKEVWSGTDVITTQGTISMNVEIHGCALFLLNCN*

>Glyma.08G060700|Glyma.08G060700.1|4677376|GH 5/Ricin
MVRKSSILLACLLAIFTSCCNSLPLSVHKRWIIDDATGKRVKLHCAHWVAHATPMLAEGL
DKSPMNDIAANIAKAGFNCVRLSYATYMFTRYANNTVRDIFHTHDIPGIVSAIENYNPRV
LNMTHLQAYEAVVDALGDHGVMVLIDNHVSLAKWCCANDDQNGFFGDRHFNTSEWLQGLA
FIAHHFKGKPNVFAMDLRNELRGSRQNHHDWYKYMTQGANTIHDINPDFIVIISGLAFDN
DLSFLKKKPLDLNFPHKIVYESHIYSVSGDTHRWRVQPVNWICNATIQLLHQQSSFLLSG
KNPAPLLVSEFGYDMTGGSFADNMYLPCIVSYFASVDLDWSLWAFQGSYYYRQGKVGLGE
SYAVMDDDWKSYRDPNFTQKFELLQRMVQDPTSNVSKSNIIFHPLTGYCAHVNNSKELVM
GDCKSNSLWSYEGDGSPIRLMNSAKCLKAVGERLPPSLSEDCLSPQSSWKTVSMTGLHLA
TFDKDGDLLCLEKDSNSSKIVTSKCICISDDDSSCLDNPQSQWFKLVSTNV*

>Glyma.08G130600|Glyma.08G130600.1|10025431|GH 5/Ricin
MGRWRPSTLVFTILSAPIIIIIALLSSGGTTTVEDVDNDHTMAVTGLLHTDSRWILNQGG
QRVKLACVNWVSHLEVAVAEGLSKKPVDAISKGIKSMGFNCVRLTWPTLLATNDSLASLS
VRRSFQSLGLLESVAGVQTNNPSIIDLPLIQAFQAVVKSLGDNDVMVILDNHLTNPGWCC
GYSDGNGFFGDKFFNPDQWIFGLTKMATLFNGVTNVVGMSLRNELRGPKQNVNDWYKYMV
KGAEAVHAANPDVLVILSGINFDTSLSFIRDRPVSLTFKGKLVFEVHRYGFTDGGAWADG
NPNQVCGKVTADIKQTSTFLVDQGWPLFVSEFGGDLRGTNVNDNRYLNCFLALVAELDLD
WAYWTLVGSYYFREGVIGMEEFYGLLTWDWTQVRSTSFLNRISALQIPFRGPGIIEGSAY
KLIFHPLTGLCVISKSQLTSLTLGPCSSSDAWTYTPQKTLLINNTNFCIHAEQEGKPATL
SITCSDANSKWEMISDSNMHLSSKLSDGSNLCLDVDDNNIIVTTACKCLNQDKTCDPASQ
WFKLIDSGRRSISTTSTLSMLNSPDILWQPLSSI*

>Glyma.08G131400|Glyma.08G131400.1|10070505|GH 27/Ricin
MMKCFSVSLASLWVLLALFSLSVSSQNISESGLQQASLPPRGWNSYDSFCWTISEDEFLQ
SAEMVSQRLKAHGYQFVVVDYLWYRKKVKGAYPDSLGFDVIDEWGRMIPDPGRWPSSIGG
KGFSEVANKVHSLGLKFGIHVMRGISTQAVNANTPILDITKGGAYQESGRVWHAKDIAMP
ERACAWMPHGFMSVNTKLGAGRAFLKSLYEQYAAWGVDLVKHDCVFGDDLDLNEISYVSE
VLSVLNRPIVYSLSPGTSVTPAMAKDVSGLVNMYRITGDDWDKWEDVKAHFDVTRDFSTA
NMIGGKGLKGNSWPDLDMLPFGWLTDPGSNEGPHRFSKLTLEEKRTQMTLWSLAKSPLMY
GGDVRRIDPTTYELITNPTLLEINYFSSNNMEFPYITSSINLKHPGGKERRLKKGIHSLG
LTSCSESKARGWSIESLNQDLERICWKKGLENKHQAPFCVHKRELQFRLDGVSMYQEDYR
GKHQLVATDRMKFCLDASPRRKVTSKEFKRGTFSPCRWDSNQIWELNSNGTMVNSYSGLC
ATVEYIEANVNSGGIRSWIATGRTGEIYLAFFNLSEQKTEIYAKTSYLAKVLPDKSITSC
KGEEVWSGTDVITTQGTISMNVEIHGCALFVLNCN*

>Glyma.11G179700|Glyma.11G179700.1|24537774|GH 5/Ricin
MIPLITVRVIVLLLLGVTLQITKPVVVKGFPLHTSERWIVDESGKRVKLACVNWVSHLDA
VVAEGLSQQPLDEISKRIKTMGFNCVRLTWPLFLVTNDSIASLTLRNSFQNLGLIQSLNG
VQAINPSIIDLPLIKAYQAVVKSLGENDVMVILDNHVSQPGWCCSNLDGNGFFGDQYFDP
DLWIMGLTKMATIFKGVTNVVAMSLRNELRGPRQNVNVWYRYMPKGAEAVHAANPDVLVI
LSGLNFDTNLSFIRNEAVKLSFNGKLVFEVHWYSFSDGQAWTLGNPNQVCGQVTENVMRR
AGFLLDQGWPLFVSEFGVDLRGTSVNDNRYLNCFMALVAQLDLDWALWTLGGNYYIRQGD
VGMEEYFGILNSDWIQVRNTSFLQRISAIQLPFKGPGLSEAKPYKVIFHPLTGLCILRNS
PVEPLMRLGPCSNSDAWEYTDQKILSIKGTYFCLQAEEEGKQAKLGNACSGSNSRWEMIS

DSKMHLSTQTNNASGVCLDVDTNNIIVTNICQCLSKDNTCDPATQWFKLVDSTRKSRHYL
RIPS*

>Glyma.11G243600|Glyma.11G243600.1|33743871|GH 27/Ricin
MKCFSLSSISLFILLFLCSESVSSQNVSESEQQQASIPPRGWNSYDSFCWTISEEEFLQS
AEIVSQRLHDHGYEYVVVDYLWYRRKVEGAYHDSLGFDVIDEWGRMVPDPGRWPSSENGK
GFTEVANQVHSMGLKFGIHVMRGISTQAVNANTPILDTTMGGAYQESGRVWYAKDIAIPE
RACAWMSHGFMSVNTKLGAGKAFLRSLYEQYAAWGVDFVKHDCIFGDDFDLNEISYVSEV
LKEFDRPIVYSLSPGTSATPAMAKDVSGLVNMYRITGDDWDTWGDVKAHFDITRDFSNAN
MIGAKGLMGNSWPDLDMLPFGWLTDPGSNEGPHRYSYLNLEEKKTQMTLWSMAKSPLMYG
GDVRKIDPSTYDVITNPTLLEINSFSSNNMEFPYITSVNSEDQDLGRPMRRSSMEIKTSY
THSLGLTSCTESKASGWASESLNQYLERICWKRSLGNKHLAPFCVHKRELYFPFDEASMY
QEYHQRKHHLVATNRIKFCLDASPKRKLTSKEFKRGTFSPCSWDSNQMWELNPNGTLVNS
YSGLCATVESSEDTINSGGLHSWIATGRKGEVYVAFFNLSEQKRVISAKTSDLAKVLPGR
DFSSCQGSEVWSGDAIEITQGTLSTAVEVHGSALIVLNCNESPLLSPKIERKKENK*

>Glyma.18G012900|Glyma.18G012900.1|914862|GH 5/Ricin
MGWSFPLSPLVTTITIVLLLCVTLQTKVVKAFPLYTQNRWIVDGNATRVKLACVNWVSHL
EYMVAEGLGERPLDGIAKEIKSMGFNCVRLTWPIYLITNDSLATLTVRQSFNNLGLPQAI
SALQVNNPSLIDLPLIKAYQDVVKGLGDKGLMVILDNHVSKPQWCCSNDDGNGFFGDQYF
DPDLWIKGLTKMATLFKGVTNVVAMSLRNELRGPRQNANDWFKYMPKGAEAVHGANPDVL
VIMSGLNYDLDLSFLRKQQVKLSFSRKLVFELHWYSFSDGDSWTTENPNQVCGKVTGRVM
RSAGYLLEQGYPLVLSEFGWDLRGTNQNDNSYFNCLLPLAAQLDFDWAYWTLAGSYYLRE
GTVGLIEVYGILTQNTTLPTTTFLLQRISAIQLPYRGPGLSEVEAHKVIFHPLTGLCISG
KLEPLKLGPCSNSEGWEYTAQKVLSVKGRNSTCLQAEGEGKEAKLGNECSVWEIVSDSKL
HLSSKINNASDVCLDVDSNNNIVTNACKCLSGDKTCDPASQWFKLVDSTRKST*

>Glyma.18G013500|Glyma.18G013500.1|951791|GH 27/Ricin
MKCFSLSSISLFILLFLCSESYHLVQMACFACLLSECIIIPQMTLWSMAKSPLMHGGDVR
KIDPSTYDVITNPTLLEINSFSSNNMEACEPSIFFGMEEIIVNSEDQDLGKQMRRSSKEI
KTTYTHSLGLTSCTESKASGLASGSLNQYLERICWKRSLGNKHLAPFCVHKRELYFPFGE
VGMYQEYHHYHLVATNRIKFCLDASPKHKLTSKEFKRGTFSPCRWDSNQMWGLNPNGTLV
KSYSGLCATVESSEDTINSGGLHSWIATGRKGMLLTKLQINLKVVLKVLNVHQLNWFNLS
SSSGRSSIQSNEHF*

Table S2. Contribution of tandem and segmental duplication events to the expansion of the lectin families.

	Percentage			
	**tandem**	**segmental**	**both**	**other**
CRA	0.0	66.7	0.00	33.3
EUL	66.7	0.0	0.00	33.3
GNA	48.2	20.5	21.08	10.2
Hevein	33.3	50.0	0.00	16.7
Jacalin	0.0	100.0	0.00	0.0
Legume	37.2	41.5	12.77	8.5
LysM	2.1	70.2	10.64	17.0
Nictaba	45.5	36.4	13.64	4.5
Ricin B	10.0	40.0	30.00	20.0
